# Radiosynthesis, Preclinical, and Clinical Positron Emission Tomography Studies of Carbon-11 Labeled Endogenous and Natural Exogenous Compounds

**DOI:** 10.1021/acs.chemrev.2c00398

**Published:** 2022-11-18

**Authors:** Antonio Shegani, Steven Kealey, Federico Luzi, Filippo Basagni, Joana do Mar Machado, Sevban Doğan Ekici, Alessandra Ferocino, Antony D. Gee, Salvatore Bongarzone

**Affiliations:** † School of Biomedical Engineering & Imaging Sciences, King’s College London, King’s Health Partners, St Thomas’ Hospital, London SE1 7EH, United Kingdom; ‡ Department of Pharmacy and Biotechnology, Alma Mater Studiorum−University of Bologna, via Belmeloro 6, 40126 Bologna, Italy; § Institute of Organic Synthesis and Photoreactivity, Italian National Research Council, via Piero Gobetti 101, 40129 Bologna, Italy

## Abstract

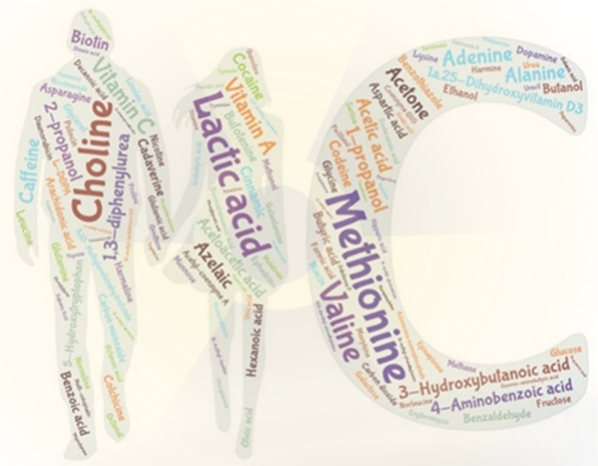

The presence of positron emission tomography (PET) centers at most major hospitals worldwide, along with the improvement of PET scanner sensitivity and the introduction of total body PET systems, has increased the interest in the PET tracer development using the short-lived radionuclides carbon-11. In the last few decades, methodological improvements and fully automated modules have allowed the development of carbon-11 tracers for clinical use. Radiolabeling natural compounds with carbon-11 by substituting one of the backbone carbons with the radionuclide has provided important information on the biochemistry of the authentic compounds and increased the understanding of their *in vivo* behavior in healthy and diseased states. The number of endogenous and natural compounds essential for human life is staggering, ranging from simple alcohols to vitamins and peptides. This review collates all the carbon-11 radiolabeled endogenous and natural exogenous compounds synthesised to date, including essential information on their radiochemistry methodologies and preclinical and clinical studies in healthy subjects.

## 1 Introduction

### 1.1 Background

Living biological systems are composed of myriad molecules providing a physical and functional scaffold for life to thrive and propagate. The molecules exist in numerous forms: messenger and transport molecules, sources of fuel, structural building blocks, or more complex molecular arrangements such as membranes, proteins, and DNA. In probing the complexity and function of living systems, the study of the molecules that create this ecosystem is crucial to enable our understanding of normal function and disease in humans and other organisms.

Complex biological systems are composed of molecules created by the organism itself (endogenous compounds) and molecules not synthesized *in situ* but acquired from its environment (exogenous compounds). Exogenous compounds can be natural products already existing in the natural world but not synthesized by the organism *per se* (*e.g.*, “essential” amino acids) or compounds synthesized in a laboratory (*e.g.*, therapeutics).

Many techniques have been used to study the role of endogenous and natural exogenous compounds in humans, including the use of radiolabeled compounds. Over the last 50 years, the *in vivo* imaging of radiolabeled compounds in humans has been made possible by the technique called positron emission tomography (PET) using compounds radiolabeled with biogenic radionuclides such as Carbon-11.

PET is widely considered the most sensitive technique for noninvasively studying physiology, metabolism, and molecular pathways in the living human being. Over the last few decades, tremendous advances have been made in radiosynthetic/radioanalytical chemistry and instrumentation, including improved detector sensitivity/responsivity, radiolabeling techniques, signal processing, and instrumentation. These technological advances have significantly increased the number of carbon-11 labeled compounds available for PET studies, providing valuable tools for understanding human physiology in health and disease. This comprehensive review aims to compile a library of the most important carbon-11 labeled endogenous and natural exogenous compounds used to date, including synthetic methods and *in vivo* distribution and kinetics, as an essential reference for new and established researchers and practitioners in the field.

### 1.2 Scope of the Review

This review describes the synthetic methods and routes developed for radiolabeled endogenous and natural exogenous compounds, focusing on the synthetic methods applied to carbon-11 chemistry and their *in vivo* kinetics in preclinical and clinical studies. The compounds have been divided into 11 categories: alcohols, alkaloids, amino acids, enzyme cofactors and vitamins, endogenous gases, fatty acids, hormones and neurotransmitters, nucleotides, peptides, sugars, and miscellaneous compounds with a (1) general introduction, (2) radiosynthesis, (3) preclinical, and (4) clinical studies in healthy subjects described for each radiopharmaceutical compound. Each section contains a selection of synthetic schemes and images from *in vivo* PET scanning as appropriate. This review covers all relevant publications up to May 2022.

### 1.3 Carbon-11

In 1934, Lauritsen *et al*. observed the formation of a radionuclide with a half-life of 20 min following the deuteron irradiation on boron oxide.^1^ This radionuclide was subsequently identified as carbon-11 (^11^C), formed by the ^10^B(d,n)^11^C nuclear reaction and isolated in the form of carbon-11 dioxide ([^11^C]CO_2_) and carbon-11 monoxide ([^11^C]CO).^2^ In 1939, the formation of carbon-11 by the bombardment of nitrogen-14 gas with high-energy cyclotron-produced protons was achieved by Barkas.^3^ To date, the ^14^N(p,a)^11^C remains the most common method of producing ^11^C.

Carbon-11, with a physical half-life of 20.4 min, decays to stable boron-11. Decay occurs primarily (99.8%) by positron emission, with the emitted positron having mean energy of 0.386 MeV and a mean range of 1.2 mm in water.^4^ The utility of ^11^C as a radiolabel for biological applications is evident as the ubiquity of carbon in biomolecules allows the substitution of a stable carbon-12, with ^11^C producing a corresponding radioactive analogue (isotopologue) with chemical and physiological properties indistinguishable from the carbon-12 isotopologue. The first chemical manipulation with ^11^C was reported by Long in 1939, in which [^11^C]CO_2_ was used to produce the radioactive endogenous compound potassium [^11^C]oxalate.^5^ In the same year, Ruben *et al*. reported the first biological application of ^11^C in their study of [^11^C]CO_2_ uptake during photosynthesis.^6^ The first human experiments with ^11^C were performed in 1945 by Tobias *et al*. studying the [^11^C]CO uptake and bodily distribution following inhalation of the gas.^7^

The emergence of medical cyclotrons in the 1960s and PET scanners in the late 1970s has driven ^11^C radiochemistry research to meet the demands for new or improved tracers for an expanding array of biological targets.^8^ Short-lived positron-emitting carbon-11 has thus been widely employed to study the fate of labeled molecules in biological systems, most notably using the *in vivo* medical imaging technique PET. As carbon is present in almost all biologically active molecules, methods developed for specifically labeling the same molecule in different positions enable the study of the divergence of metabolic pathways represented by different radiolabeled metabolites generated. For example, serial studies using β- and carboxy labeled [^11^C]DOPA in the brains of the same subjects have enabled the differentiation of [^11^C]dopamine formation from [^11^C]CO_2_ formation; each labeled compound having a distinct kinetic profile corresponding to the enzymatic cleavage of radiotracers after being acted upon by DOPA decarboxylase (see section 8.1). The following sections summarize the extensive efforts of generations of radiochemists to label endogenous and natural products to understand their function in mammalian systems, health, and disease.

## 2 Alcohols

Simple alcohols are ubiquitous in nature. Methanol is usually present in human body fluids in trace amounts derived from dietary sources, some normal metabolic processes, and by the action of colonic bacteria on pectin.^9^ Small amounts of ethanol are endogenously produced by gut microflora through anaerobic fermentation.^10^ However, most ethanol detected in biofluids and tissues likely comes from consuming alcoholic beverages.^11,12^ Propanol exists as two different isomers, 1-propanol and 2-propanol, which are also considered byproducts of bacterial fermentation processes.^13^ Butanol is produced in small amounts by gut microbial fermentation through the butanoate metabolic pathway and can be detected in blood and urine samples.^14^ Butanol has similar effects as ethanol when ingested and is considered a central nervous system (CNS) depressant. Carbon-11 alcohols have been mainly used to study their distribution in the body and measure the blood-brain barrier (BBB) permeability and cerebral blood flow (CBF) (Table 1).

**Table 1:** Carbon-11 Labeled Alcohols

compds	radiolabeling position	preclinical and clinical studies	synthon	molar activity (*A*_m_)^15^ GBq/μmol	RCY^15^	total time (min)	ref
methanol	*1*-	monkeys^16^	[^11^C]CO_2_	nr^a^	95%	nr	(17)
ethanol	*1*-	rats,^18,19^ rabbits,^18^ dogs,^18,20^ cats,^21^ monkeys,^16,22^ humans^22^	[^11^C]CO_2_	nr	nr	60	(21)
*1*-propanol	*1*-	nr	[^11^C]CO_2_	6.5	nr	40	(23)
*2*-propanol	*2*-	monkeys^16^	[^11^C]CO_2_	nr	nr	2^b^	(16)
butanol	*1*-	rats,^24−28^ dogs,^25,29,30^ monkeys,^16,31^ humans^31−33^	[^11^C]CO	nr	71%	65	(34)
			[^11^C]CO_2_	nr	73%	37	(35)

a nr: not reported.

b Reaction time.

### 2.1 Methanol

#### 2.1.1 Radiosynthesis

**Figure 1:**
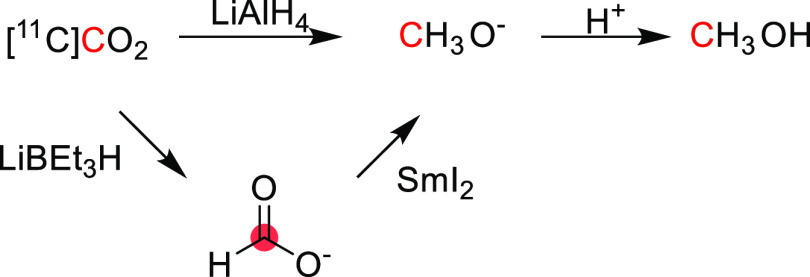
Radiosynthesis of [^11^C]methanol using [^11^C]CO_2_. ^11^C radionuclide position is highlighted in red.

The synthesis of [^11^C]methanol was first reported in 1972 by Christman *et al.* as a precursor in the synthesis of [^11^C]formaldehyde for the enzymatic labeling of [*methyl*-^11^C]thymidine.^36^ In this process, [^11^C]CO_2_ is cryogenically trapped and then released into a solution of LiAlH_4_ in tetrahydrofuran (THF), where it is reduced to [^11^C]methoxide. Subsequent hydrolysis forms [^11^C]methanol, which can be distilled into a second vessel if required (Figure 1). This approach is regularly used in the “wet method” of [^11^C]CH_3_I production, where [^11^C]methanol is subsequently iodinated.^37^ Roeda and Crouzel,^38^ however, report that non-negligible quantities of radioactivity remained in the reduction vial when distilling out [^11^C]methanol and identified this as [^11^C]formate arising from incomplete reduction.^39^ The same group reported that the reducing agent SmI_2_ could improve the yield of [^11^C]methanol through enhanced reduction of [^11^C]formate.^38^ This could be performed either by treating the lithium hydride-reduced [^11^C]CO_2_ mixture with SmI_2_ (88% radiochemical yield, RCY) or by making [^11^C]formate intentionally using LiBEt_3_H, followed by SmI_2_ reduction (90–100% RCY) (Figure 1). [^11^C]Methanol production has also been reported using an alumina column impregnated with ethereal LiAlH_4_.^17^ This method allows for direct trapping and reduction of [^11^C]CO_2_ from the irradiated target gas, followed by hydrolysis on the column, with a reported RCY of >95%.

#### 2.1.2 Preclinical Studies

In 1976, Raichle *et al*. performed a scintigraphy study to measure the BBB permeability of [^11^C]methanol in rhesus monkeys following intra-arterial injection.^16^ This allowed the estimation of the fraction of labeled [^11^C]methanol extracted by the brain during a single capillary transit and the brain capillary surface area.^16^

### 2.2 Ethanol

#### 2.2.1 Radiosynthesis

**Figure 2:**
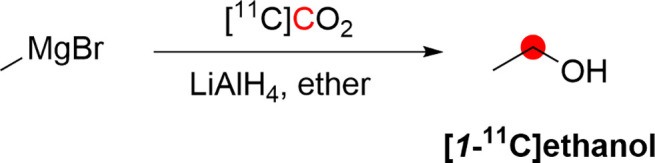
Synthesis of [*1*-^11^C]ethanol using [^11^C]CO_2_. ^11^C radionuclide position is highlighted in red.

[*1*-^11^C]Ethanol was synthesized by DeGraza *et al*. in 1974 by reaction of CH_3_MgBr with [^11^C]CO_2_ followed by reduction using LiAlH_4_.^21^ In total, the researchers produced 37 MBq of [*1*-^11^C]ethanol from 370 MBq of [^11^C]CO_2_ trapped in a NaOH solution within 60 min (Figure 2).^21^

#### 2.2.2 Preclinical Studies

**Figure 3:**
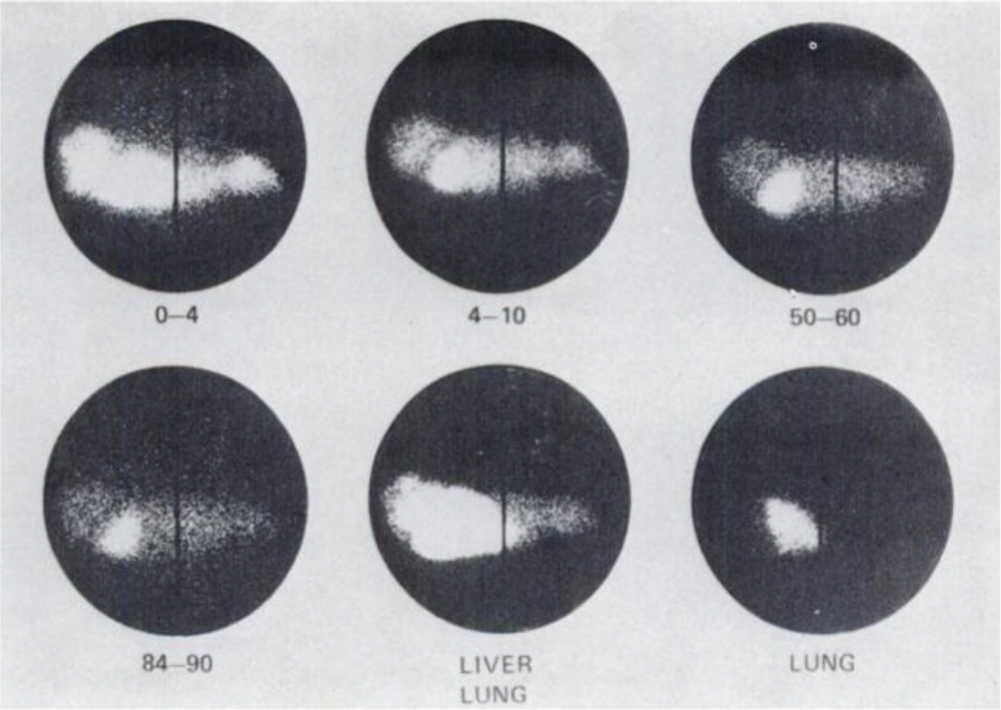
Images of [*1*-^11^C]ethanol uptake in cats’ liver using gamma scan. Reproduced with permission from ref (21). Copyright 1975 Society of Nuclear Medicine. This work is licensed under a Creative Commons Attribution 4.0 International License (https://creativecommons.org/licenses/by/4.0/).

In 1973, Robinson *et al*.^18^ studied [*1*-^11^C]ethanol extraction in the brain of rats, rabbits, and dogs, and Poe *et al*.^20^ in the heart of dogs; however, quantitative results have not been reported. A year later, DeGrazia *et al*. reported *in vivo* study of [*1*-^11^C]ethanol in cats. The results showed a high accumulation of radioactivity in the liver (Figure 3), with the authors concluding that some aspects of ethanol metabolism could be assessed in specific tissues such as the liver.^21^

In 1976, the extraction of [*1*-^11^C]ethanol by the brain was studied *in vivo* in six adult rhesus monkeys (*Macaca mulatta*) by external detection of the time course of these tracers after their internal carotid artery injection. The data demonstrated the feasibility of accurately measuring brain permeability of highly diffusible substances and showed that [*1*-^11^C]ethanol could freely equilibrate within the brain.^16^ Another study in monkeys was performed in 2002 by Gulyas *et al*. to evaluate the contribution of [*1*-^11^C]ethanol to the regional cerebral radioactivity arising from de-esterification of [*ethyl*-^11^C]vinpocetine.^22^ [*1*-^11^C]Ethanol rapidly entered the brain, reaching a maximum between 60 and 120 s after iv injection. After this peak, the radioactivity in the brain rapidly declined (*t*_1/2_ = 250 s).^22^

In 1980, Kleinanen *et al*. reported liver perfusion experiments in male Wistar rats after injection of [*1*-^11^C]ethanol through the portal vein.^19^ The results showed a maximal extraction of about one, indicating that [*1*-^11^C]ethanol distribution is flow-limited in the liver.^19^

#### 2.2.3 Clinical Studies

The distribution of ethanol was followed in a healthy human in a fasting state after the peroral administration of [*1*-^11^C]ethanol. After 10 min, most of the radioactivity was in the stomach, but smaller amounts were observed in the proximal part of the small intestine. After 30 min, intestinal and liver radioactivity concentrations increased. After 40 min, the uptake in the liver was further increased, and activity could be detected throughout the body. During this experiment, a considerable part of the activity was retained in the stomach and liver. The distribution in all the other tissues was nearly uniform, and there was no distinct accumulation in the brain.^40^

**Figure 4:**
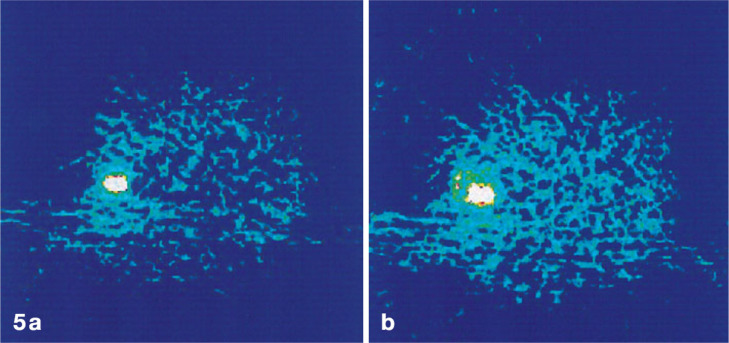
[*1*-^11^C]Ethanol intratumoral administration PET scans after (5a) 5 min and (b) 45 min. Reproduced with permission from ref (42). Copyright 2014 Springer Nature.

Pharmacokinetic imaging following intratumoral injection of [*1*-^11^C]ethanol in eight patients with hepatocellular carcinomas showed that five out of seven tumors demonstrated high uptake shortly after injection, followed by a plateau during the remainder of the 45 min study (Figure 4).^41^ The PET data demonstrated no significant elimination of radioactivity from the tumor and no accumulation in the surrounding liver tissue.^42^

### 2.3 *1*-Propanol and *2*-Propanol

#### 2.3.1 Radiosynthesis

**Figure 5:**
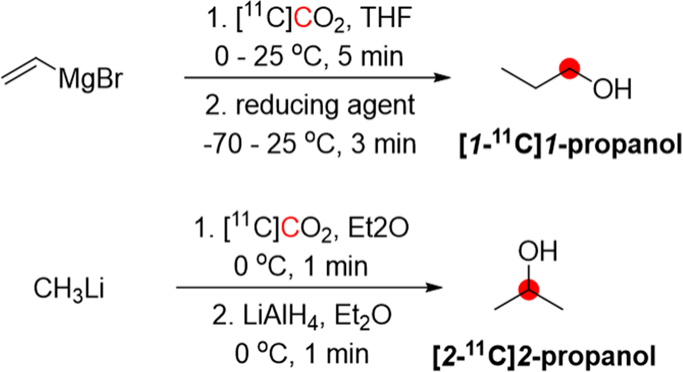
Radiosynthetic schemes of [*1*-^11^C]*1*-propanol and [*2*-^11^C]*2*-propanol. ^11^C radionuclide position is highlighted in red.

[*1*-^11^C]1-Propanol was obtained from the reaction of [^11^C]CO_2_ with vinyl magnesium bromide, followed by reduction with different reducing agents, with the best RCY achieved with AlH_3_. After purification with gas chromatography, RCY was 4–6.5% from [^11^C]CO_2_ in 40 min synthesis time from the end of [^11^C]CO_2_ production (Figure 5).^23^ Using a slightly different method, CH_3_Li was reacted with [^11^C]CO_2_ in ether, followed by reduction with LiAlH_4_, to obtain 185–370 MBq of [*2*-^11^C]*2*-propanol from 370–740 MBq of [^11^C]CO_2_.^16^

#### 2.3.2 Preclinical Studies

In six adult rhesus monkeys [*2*-^11^C]*2*-propanol, injected by the internal carotid artery, was evaluated for its brain permeability by determining the extraction rate of the brain for a single capillary transit.^16^ [*2*-^11^C]*2*-Propanol bolus freely exchanges with the brain, results that are consistent with lipophilicity and establish it as one of the best short-chain alcohols for brain permeability studies.^16^

### 2.4 *1*-Butanol

#### 2.4.1 Radiosynthesis

**Figure 6:**
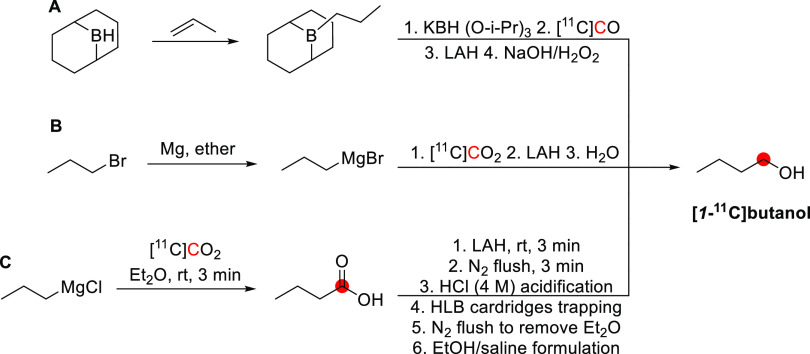
Radiosynthesis of [*1*-^11^C]butanol using [^11^C]CO or [^11^C]CO_2_. ^11^C radionuclide position is highlighted in red.

In 1985, Kothari *et al*. described the synthesis of [*1*-^11^C]butanol *via* two routes: carbonylation of an organoborane (Figure 6A) and carbonation of a Grignard reagent (Figure 6B).^34^ The reaction of [^11^C]CO with *B*-*n*-propyl-*9*-borabicyclo[*4*.*4*.*1*]nonane, followed by oxidation in an alkaline medium, produced [*1*-^11^C]butanol within 65 min with a RCY of 33–71% based on the delivered activity of [^11^C]CO, and radiochemical purity (RCP)^15^ >95%.^34^ The reaction of [^11^C]CO_2_ with *1*-propylmagnesium bromide, followed by lithium aluminum hydride (LAH) reduction, produced [*1*-^11^C]butanol within 27 min with a RCY of 55–74% and RCP of 95–99%.^29,34^ A robotic synthesis of [*1*-^11^C]butanol taking 25 min has also been described with a RCY of 11–15%.^43^

Waterhouse *et al*. described the automated radiosynthesis of [*1*-^11^C]butanol consistent with Good Manufacturing Practice (GMP) guidelines.^35^ Ten batches of [*1*-^11^C]butanol product were produced consecutively and fully compliant with United States Pharmacopeia guidelines within 37 ± 5 min, with a RCY of 73 ± 13%, and RCP of 97.7 ± 1.3% (Figure 6C).^34,35,44,45^

#### 2.4.2 Preclinical Studies

[*1*-^11^C]Butanol has been evaluated in rats,^24−28^ dogs,^25,29,30^ and monkeys.^16,31^ In Sprague-Dawley rats, a measurement of CBF was performed after a bolus intravenous injection of [*1*-^11^C]butanol. To improve the preconditions for external hyperthermia treatment of cancer, Knapp *et al*. measured the effects of a specific calcium antagonist and 5-hydroxytryptamine (5-HT) on tumor-to-muscle blood flow in tumor transplants of rats.^24^ [*1*-^11^C]Butanol appears to be an appropriate radiotracer for assessing blood supply to malignant tumors relative to muscle.^25^

In 1984, a new method for measuring CBF in rats was described by Takagi *et al.*, which is noninvasive to the brain, skull, or large cervical vessels, minimizes blood loss and gives stable blood flow values.^27^ Three years later, the same group developed a quantitative method to measure the water extraction fraction of rat brain after successive intravenous bolus injections of [*1*-^11^C]butanol.^27,28^

A study was performed in dogs to determine whether regional myocardial perfusion can be assessed quantitatively by administering a freely diffusible tracer intravenously at an exponentially increasing rate, with the authors finding that the approach permits accurate measurement of myocardial perfusion and that it should prove helpful in the noninvasive measurement of regional myocardial perfusion *in vivo* by PET.^30^

Another study by Knapp *et al*. showed that more than 80% of the activity was cleared from the blood within 1 min following the administration of [*1*-^11^C]butanol into the aorta of two dogs.^25^ Within 25 min, activity was only observed in the liver, spleen, and kidneys, while muscle and whole body showed constant levels.^25^

In monkeys, preliminary results with [*1*-^11^C]butanol indicate no diffusion limitation, and it is freely diffusible in the rhesus monkey brain after an intracarotid injection.^16,31^

#### 2.4.3 Clinical Studies

Herscovitch *et al*. in 1987 validated the use of [*1*-^11^C]butanol in 17 healthy humans as an alternative freely diffusible tracer for PET to determine the underestimation of CBF that occurs with [^15^O]H_2_O.^31^ Average global CBF was significantly greater when measured with [*1*-^11^C]butanol than with [^15^O]H_2_O.^31^ Quarles *et al*. in 1993 evaluated [*1*-^11^C]butanol in three healthy humans after intravenous administration to further understand the best way to measure regional CBF with PET.^32^ In 2012, Pagani *et al*. studied 13 patients with autism spectrum disorder (ASD) of ordinary intelligence and 10 IQ-, sex-, and age-matched healthy controls who underwent PET/CT (computerized tomography) using [*1*-^11^C]butanol.^33^ Tracer uptake reached a plateau at around one minute before decreasing with time in all patients. ASD patients showed increased blood flow in areas such as the limbic, posterior associative, and cerebellar cortices. Significant CBF differences were found between highly functioning ASD subjects and healthy controls in part of the posterior right hemisphere in limbic, posterior associative, visual, and cerebellar cortices.^33^

## 3 Alkaloids

Of all carbon-11 labeled natural alkaloids, only *N*,*N*-dimethyltryptamine and galantamine are endogenous. *N*,*N*-Dimethyltryptamine is a hallucinogenic compound detected in blood, urine, and cerebrospinal fluid in humans. It interacts with several serotoninergic receptors, mainly 5-HT2A and glutamate, accounting for its hallucinogenic properties. Due to little sign of adverse effects, apart from some cardiovascular and endocrine effects, *N*,*N*-dimethyltryptamine represents an exciting model for potential therapies for psychiatric disorders.^46^ Galantamine is a weak competitive and reversible cholinesterase inhibitor. Also, it is a potent allosteric potentiating ligand of human nicotinic acetylcholine receptors (nAChRs) in some regions of the brain^47^ but does not functionally act as a positive allosteric modulator.^48^ By binding to the allosteric site of the nAChRs, a conformational change occurs, increasing the receptor’s response to acetylcholine.^49,50^ Approximately 75% of a dose of galantamine is metabolized in the liver from CYP2D6 and CYP3A4.^51^

Several natural exogenous alkaloids, important for humans, have been labeled with carbon-11 to evaluate their brain uptake, bodily distribution, and metabolism (Table 2). Each of them has unique functions in the brain related to different receptors, and carbon-11 labeling could potentially be used for brain imaging:

- Bufotenine, and its methoxy analogue, *O*-methylbufotenine, are potent hallucinogenic compounds that act as nonselective serotoninergic ligands.^52^
- Caffeine is the most widely consumed CNS stimulant of the methylxanthine class,^53^ eliminated in the liver *via* cytochrome P450.^54,55^
- Cocaine is a potent addictive stimulant having many short- and long-term effects on users.^56,57^
- Codeine is a selective agonist with a weak affinity for the μ-opioid receptor (MOR).^58^ Codeine is metabolized to morphine and norcodeine in the liver by the cytochrome P450 enzyme CYP2D6^59,60^ and CYP3A4,^61^ respectively.
- Colchicine is a potent inhibitor of cellular mitosis.^62^
- *N*,*N*-*dimethyl*phenethylamine (*N*,*N*-DMPEA) is a trace amine neuromodulator in humans derived from the trace amine phenethylamine.^63,64^ There is evidence that it is a potent agonist of human trace amine-associated receptor 1.^63,65,66^ It is metabolized rapidly by monoamine oxidases (MAO) and most probably by the isoform MAO-B during first-pass metabolism.^63,65^ Thus, labeling *N*,*N*-DMPEA could be helpful for *in vivo* measurement of MAO-B activity in the brain.^67^
- Ephedrine and its derivative *N-methyl-*ephedrine cause the release of norepinephrine from storage vesicles in nerve cells and directly stimulates α- and β-adrenergic receptors.^68^
- Harmine and its derivative, harmaline, belong to the family of β-carbolines. They are known for their anxiolytic, sedation, and psychotomimetic effects acting as potent inhibitors of MAO-A and serotonin antagonists.^69,70^
- Morphine is the most used chronic and acute pain killer as an opioid receptor ligand.^71^
- Nicotine elicits the release of neurotransmitters such as norepinephrine, dopamine, acetylcholine, serotonin, glutamate, and GABA.^72^ It initiates its biological function by activating the nicotinic acetylcholine receptor through binding with ligand-gated ion channels.^73^
- Oxycodone is a highly selective full agonist of the MOR but has a low affinity for the δ-opioid and the κ-opioid receptors.^74^ Oxycodone is metabolized in the liver mainly *via* the cytochrome P450 system by the enzymes CYP3A4 and CYP2D6.^75^
- Papaverine was recently identified as a specific inhibitor of phosphodiesterase 10A (PDE10A). The latter is a phosphodiesterase that converts cyclic adenosine monophosphate (cAMP) to AMP and cyclic guanosine monophosphate (cGMP) to guanosine monophosphate, concentrated in the brain’s striatum.^76,77^
- Physostigmine mimics the binding of acetylcholine to the enzyme acetylcholinesterase (AChE)^78^ as a reversible inhibitor of AChE.^79,80^
- Psilocin is the brain-penetrant metabolite of psilocybin, its *O*-phosphorylated derivative, and acts as an agonist with moderate affinity for serotonin receptors and low affinity for dopamine receptors.^81^
- Quinidine interacts with the sodium channels in the Purkinje fibers of the heart.^82^
- Scopolamine can antagonize muscarinic acetylcholine receptors.^83,84^
- Theophylline is distributed in the extracellular fluid, placenta, mother’s milk, and CNS and is a competitive nonselective phosphodiesterase inhibitor^85−88^ and a nonselective adenosine receptor antagonist (A1, A2 and A3 receptors).^89^

**Table 2:** Carbon-11 Labeled Alkaloids

compd	radiolabeling position	preclinical and clinical studies	synthon	*A*_**m**_ GBq/μmol	RCY	total time (min)	ref
bufotenine	*N*-*methyl*-	rats^90^	[^11^C]CH_3_I	nr^a^	9%	20	(90)

*O*-methylbufotenine	*N*-*methyl*-	rats^90^	[^11^C]CH_3_I	nr	18%	30	(90)

caffeine	*1*-	nr	[^11^C]CH_3_I	114.7	27%	5	(91)
	*3*-	nr	[^11^C]CH_3_I	144.3	64%	5	(91)
	*7*-	mice,^92^ cynomolgus,^93^ monkeys^93^	[^11^C]CH_3_I	247.9	68%	5	(91)

cocaine	*N*-*methyl*-	rats,^94^ baboons,^95−99^ macaques,^100^ monkeys,^101,102^ humans^95,98,99^	[^11^C]CH_3_I	9.25	nr	35	(95)
			[^11^C]CH_3_I	9.25	nr	35	(95)
	*O*-*methyl*-	baboons^103^	[^11^C]CH_3_I	>3.7	nr	nr	(103)

codeine	*N*-*methyl*-	monkeys^104^	[^11^C]CH_3_I	nr	25%	10	(104)

colchicine	*3*-*O*-*methyl*-	monkeys^105^	[^11^C]CH_3_OTf	0.389	0.5%	50	(105,106)
	*10*-*O*-*methyl*-	mice,^107^ rats^107^	[^11^C]CH_3_I	8.88	21%	60	(108)

*N*,*N*-dimethylphenethylamine	*N*-*methyl*-	mice,^67^ rats,^109^ monkeys^109^	[^11^C]CH_3_I	3.7	35%	35	(67,109)
	[*1*-^11^C]phenethyl-	rats,^109^ monkey^109^	[*1*-^11^C]C_6_H_5_CH_2_CH_2_I	3.7	10%	50	(109)

*N*,*N*-dimethyltryptamine	*N*-*methyl*-	rats,^90^ dog^110^	[^11^C]CH_3_I	2.812	90%	50	(110)

ephedrine	*N*-*methyl*-	mice^92^	[^11^C]CH_3_I	nr	11%	45	(92)

*N*-*methyl*-ephedrine	*N*-*methyl*-	mice^92^	[^11^C]CH_3_I	nr	43%	36	(92)

galanthamine	(+)-*N*-*methyl*-	mice,^111^ rats^111^	[^11^C]CH_3_OTf	nr	14.4%	nr	(111)
	(−)-*N*-*methyl*-	mice,^111^ rats^111^	[^11^C]CH_3_OTf	nr	13.7%	nr	(111)

harmine	*O*-*methyl*-	monkeys,^112,113^ baboon,^114^ minipigs,^115^ humans^116−120^	[^11^C]CH_3_I	101.32	51%	35	(121)

harmaline	*O*-*methyl*-	monkeys,^112^ baboons^114^	[^11^C]CH_3_I	87.3	65.9%	40	(112)

morphine	*N*-*methyl*-	rats,^122^ dogs,^123^ monkeys^93,104,124^	[^11^C]CH_3_I	962	50%	45	(125)
			[^11^C]CH_2_O	nr	nr	60	(126)

nicotine	*N*-*methyl*-	mice,^127,128^ rabbits,^127^ monkey,^129,130^ humans^131−133^	[^11^C]CH_2_O	1.1	nr	30	(127)
			[^11^C]CH_3_OTf	648	60.4%	32	(134)
			[^11^C]CH_3_I	3.89	35%	30	(135)

oxycodone	*N*-*methyl*-	rats^136^	[^11^C]CH_3_I	94.7	nr	35	(136)

papaverine	*3*-*O*-*methyl*-	rats and monkeys^137^	[^11^C]CH_3_I	>740	70%	50	(137)

physostigmine	*carbonyl*-	rats,^138^ baboons,^139^ humans^140^	[^11^C]CH_3_NCO	13	19%	52	(141−143)
			[^11^C]COCl_2_	39.6	25%	35	(144)

psilocin	*N*-*methyl*-	nr	[^11^C]CH_3_I	85.1	20%	45	(145)

quinidine	*7*-*O*-*methyl*-	rats^146^	[^11^C]CH_3_I	2.22	60%	55	(147)
			[^11^C]CH_3_OTf	259	65%	45	(146)

scopolamine	*N*-*methyl*-	rats,^148,149^ humans^150,151^	[^11^C]CH_2_O	0.037	43%	40	(152)

theophylline	*6*-	nr	[^11^C]CO_2_	nr	18%	10	(153)

a nr: not reported.

### 3.1 Bufotenine and *o*-Methylbufotenine

#### 3.1.1 Radiosynthesis

**Figure 7:**
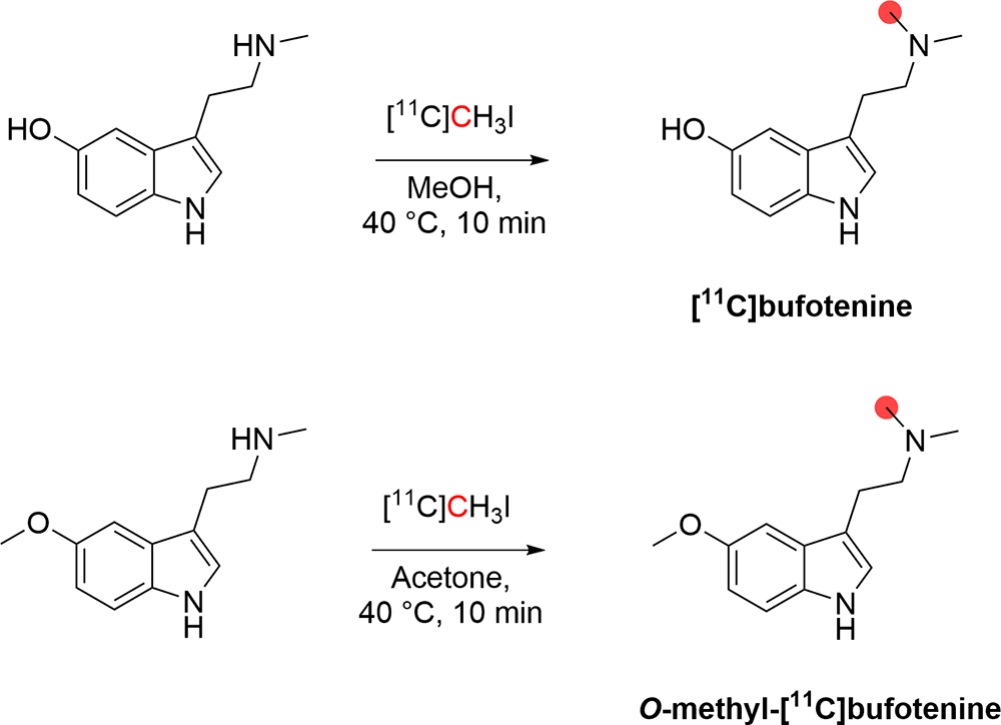
Radiosynthetic schemes of [^11^C]bufotenine and *O*-*methyl*-[^11^C]bufotenine. ^11^C radionuclide position is highlighted in red.

[^11^C]Bufotenine was obtained by *N*-methylation of *5*-hydroxy-*N*-methyltryptamine with [^11^C]CH_3_I (Figure 7). The reaction was conducted in methanol at 40 °C for 10 min and then purified using a solid-phase extraction (SPE) cartridge. In a process lasting 20 min from the end of [^11^C]CH_3_I trapping, 74 MBq of [^11^C]bufotenine was obtained starting from 1628 MBq of [^11^C]CH_3_I, in 9% RCY and 98% RCP. Similarly, *O*-*methyl*-[^11^C]bufotenine was synthesized by the *N*-methylation of *5*-methoxy-*N*-methyltryptamine with [^11^C]CH_3_I (Figure 7). The reaction was conducted in acetone at 40 °C for 10 min, and the purification was achieved using a reversed-phase SPE cartridge. In a process lasting 30 min from the end of [^11^C]CH_3_I trapping, 55.5 MBq of *O*-*methyl*-[^11^C]bufotenine was obtained starting from 1036 MBq of [^11^C]CH_3_I, with 18% RCY and 92% RCP.^90^

#### 3.1.2 Preclinical Studies

The tissue distribution of [^11^C]bufotenine and *O*-*methyl*-[^11^C]bufotenine was evaluated in healthy Wistar rats. After iv injection of [^11^C]bufotenine and *O*-*methyl*-[^11^C]bufotenine, most of the radioactivity was accumulated in the liver, kidney, lung, and small intestine, paired with a fast clearance from the blood for *O*-*methyl*-[^11^C]bufotenine and slower clearance for [^11^C]bufotenine. The brain-to-blood ratio of [^11^C]bufotenine was low, but this increased with time, while *O*-*methyl*-[^11^C]bufotenine showed relatively high accumulation in the brain at 5 min post injection (p.i.) and the radioactivity retained over time. The liver registered a significant accumulation of radioactivity for both radiotracers.^90^

### 3.2 Caffeine

#### 3.2.1 Radiosynthesis

**Figure 8:**
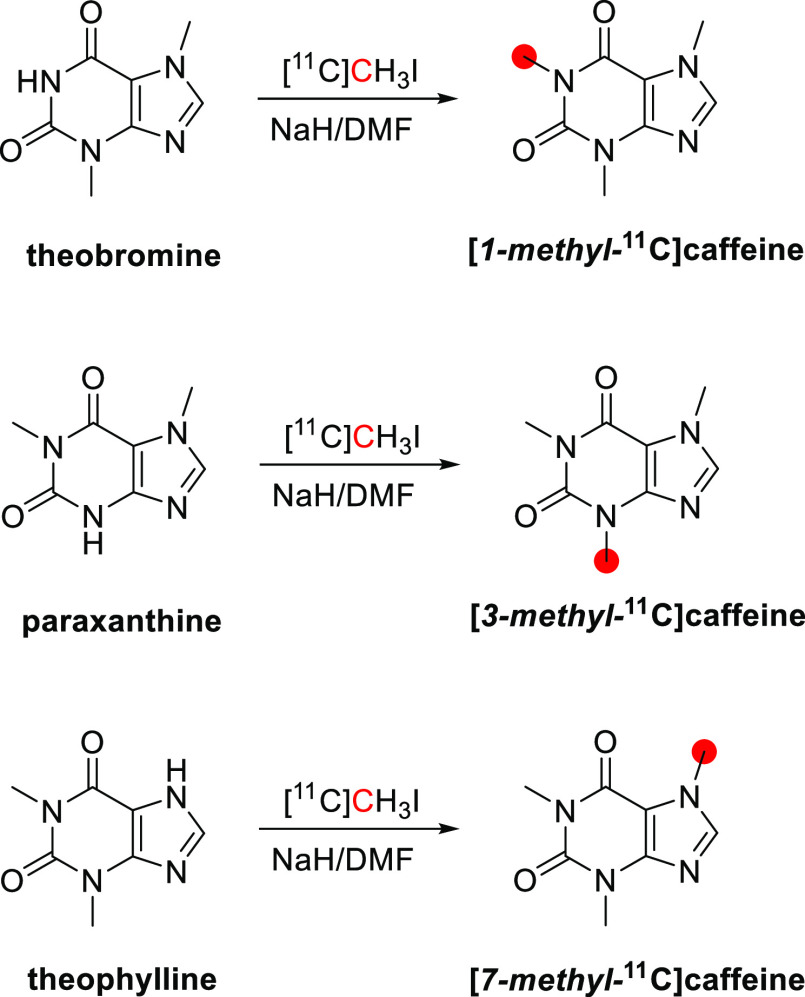
Radiosynthesis of [*1*-*methyl*-^11^C]caffeine, [*3*-*methyl*-^11^C]caffeine, and [*7*-*methyl*-^11^C]caffeine from theobromine, paraxanthine, and theophylline with [^11^C]CH_3_I. ^11^C radionuclide position is highlighted in red.

[*1*-*Methyl*-^11^C]caffeine was initially prepared by reaction of theobromine with [^11^C]CH_3_I by Maziere *et al*. in 1974.^154^ Sodium carbonate was added to a solution of theobromine in methanol, and the mixture was subsequently heated in the presence of [^11^C]CH_3_I for 10 min (Figure 8). The overall time for synthesis, purification, and sterilization was about 30 min with a RCY of 10-20%.^154,155^

[*7*-*Methyl*-^11^C]caffeine was initially prepared in 1978 by Saji *et al.*, by reaction of theophylline with [^11^C]CH_3_I in a 44 min process, with a RCY of 40%.^92,156^ A method using NaH in dimethyl sulfoxide (DMSO) to promote proton abstraction at N-7 was reported by Denutte *et al*. in 1982 (Figure 8).^157^ Following reversed-phase high-pressure liquid chromatography (HPLC), [*7*-*methyl*-^11^C]caffeine was obtained in 90% RCY, with an *A*_m_ of 2.22 GBq/μmol in a 40 min process.

In 1992, Funaki *et al*. prepared [*1*-*methyl*-^11^C]caffeine, [*3*-*methyl*-^11^C]caffeine, and [*7*-*methyl*-^11^C]caffeine from theobromine, paraxanthine, and theophylline with [^11^C]CH_3_I within a solution of NaOH in dimethylformamide (DMF) (Figure 8).^91^ The final products were isolated by HPLC with a RCY of 27%, 64%, and 68% and *A*_m_ of 114.7, 144.3, and 247.9 GBq/μmol, respectively.^91^

#### 3.2.2 Preclinical Studies

**Figure 9:**
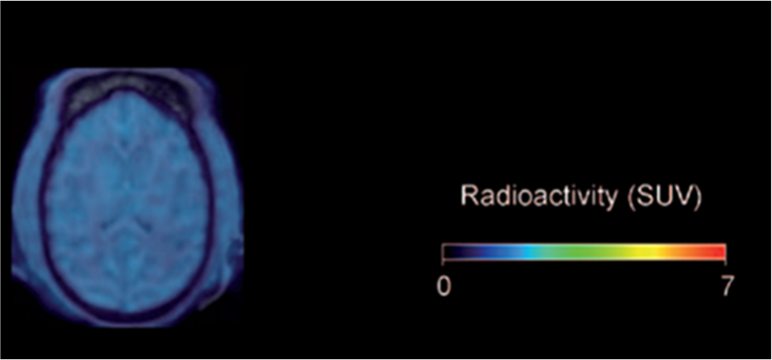
[*7*-*Methyl*-^11^C]caffeine summed retention in rhesus monkey brain (between 3 and 93 min p.i.). Reproduced with permission from ref (93). Copyright 2015 Oxford University Press.

After iv administration of [*7*-*methyl*-^11^C]caffeine in mice, the biodistribution showed high uptake in the liver, kidney, blood, and brain at 5 min p.i.^92^ In 2015, Schou *et al*. examined [*7*-*methyl*-^11^C]caffeine in cynomolgus and rhesus monkeys, supporting a preferential distribution to the brain (Figure 9).^93^ The metabolism of [*7*-*methyl*-^11^C]caffeine was assessed with the PET measurements to generate an arterial input function corrected for radiometabolites. The partition coefficients between the brain and plasma obtained in rhesus monkeys suggested that the passage across the BBB can be characterized as passive diffusion.^93^

### 3.3 Cocaine

#### 3.3.1 Radiosynthesis

**Figure 10:**
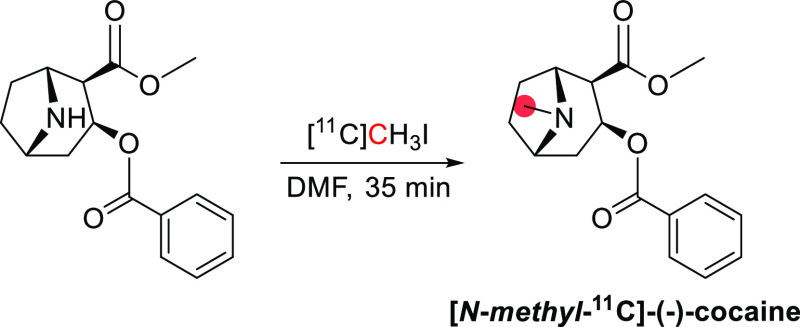
Synthesis of [^11^C]cocaine. ^11^C radionuclide position is highlighted in red.

Carbon-11 cocaine has been radiolabeled in two different positions: the *N*-*methyl*-and *O*-*methyl*-positions.^95,103^ (−)-[*N*-*methyl*-^11^C]cocaine was synthesized from norcocaine at the *N*-*methyl*-position by using [^11^C]CH_3_I (*A*_m_: 9.25 GBq/μmol, RCP > 98% in 35 min) (Figure 10).^95^ In 1990, (+)-cocaine was synthesized from (*+*)-norcocaine and [^11^C]CH_3_I with the same method. Radiosynthesis of (−)-[*O*-*methyl*-^11^C]Cocaine was achieved in 1994 using [^11^C]CH_3_I and benzoylecgonine with a *A*_m_ > 3.7 GBq/μmol.^103^

#### 3.3.2 Preclinical Studies

**Figure 11:**
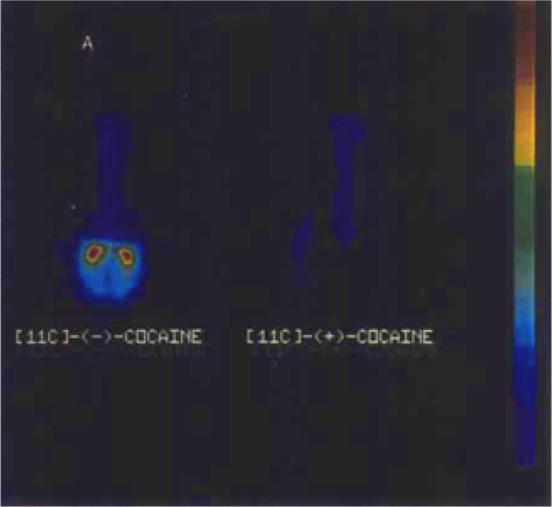
(−)-[^11^C]-Cocaine and (+)-[^11^C]-cocaine PET images in baboon brain (14 to 24 min p.i.). Reproduced with permission from ref (96). Copyright 2006 John Wiley and Sons.

(−)-[*N*-*Methyl*-^11^C]cocaine was studied in baboons to compare ^11^C-labeled cocaine uptake and metabolism under different conditions.^95^ In 1990, Gatley *et al.* labeled the less biologically active (*+*)-cocaine and compared its biodistribution and metabolism to (−)-cocaine in baboon plasma and brain by *in vitro* and *in vivo* studies.^96^ There was no brain uptake of (*+*)-cocaine due to its rapid metabolism in plasma, in which it is primarily debenzylated to give the (*+*)-ecgonine methyl ester within 30 s p.i. (Figure 11).

**Figure 12:**
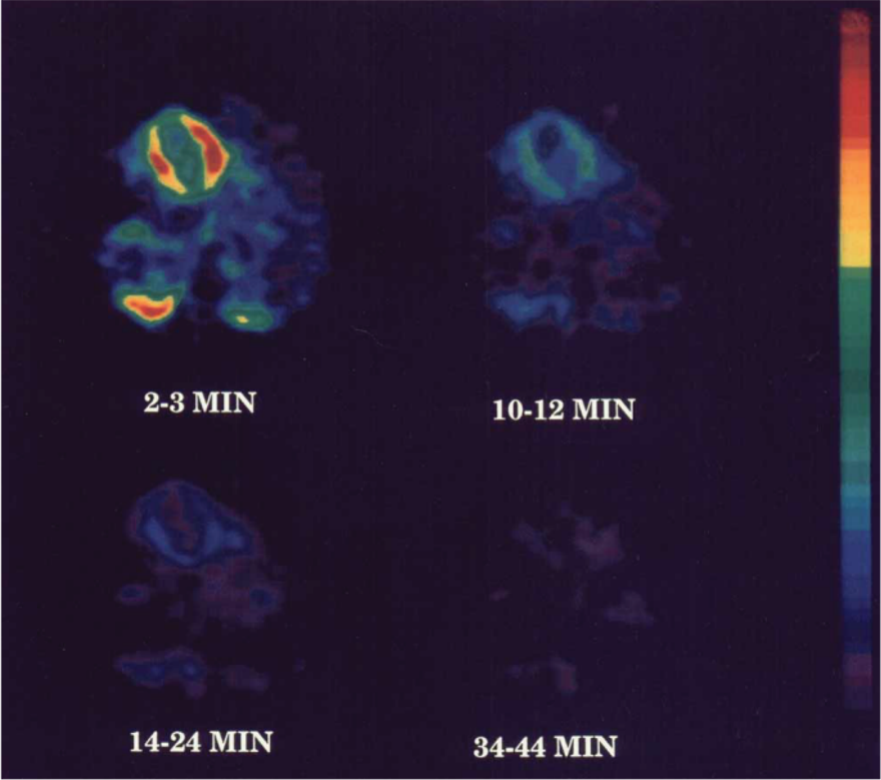
[*O*-*Methyl*-^11^C]cocaine pharmacokinetics in baboon heart over time. Reproduced with permission from ref (159). Copyright 2004 John Wiley and Sons.

Biodistribution and kinetic studies with (−)-[*N*-*methyl*-^11^C]cocaine using PET in seven adult female baboon brains after iv injection showed peak uptake in the thalamus at 2-5 min.^97^ The shortest clearance half-time was 7.9 ± 1.9 min in the cerebellum. (−)-[*N*-*methyl*-^11^C]cocaine has also been studied in the baboon,^103^ in male ddY mice,^158^ in the baboon brain,^98,99^ and (−)-[*O*-*methyl*-^11^C]cocaine has been studied in the baboon brain to probe the contribution of radiometabolites to the brain images (Figure 12).^103^

**Figure 13:**
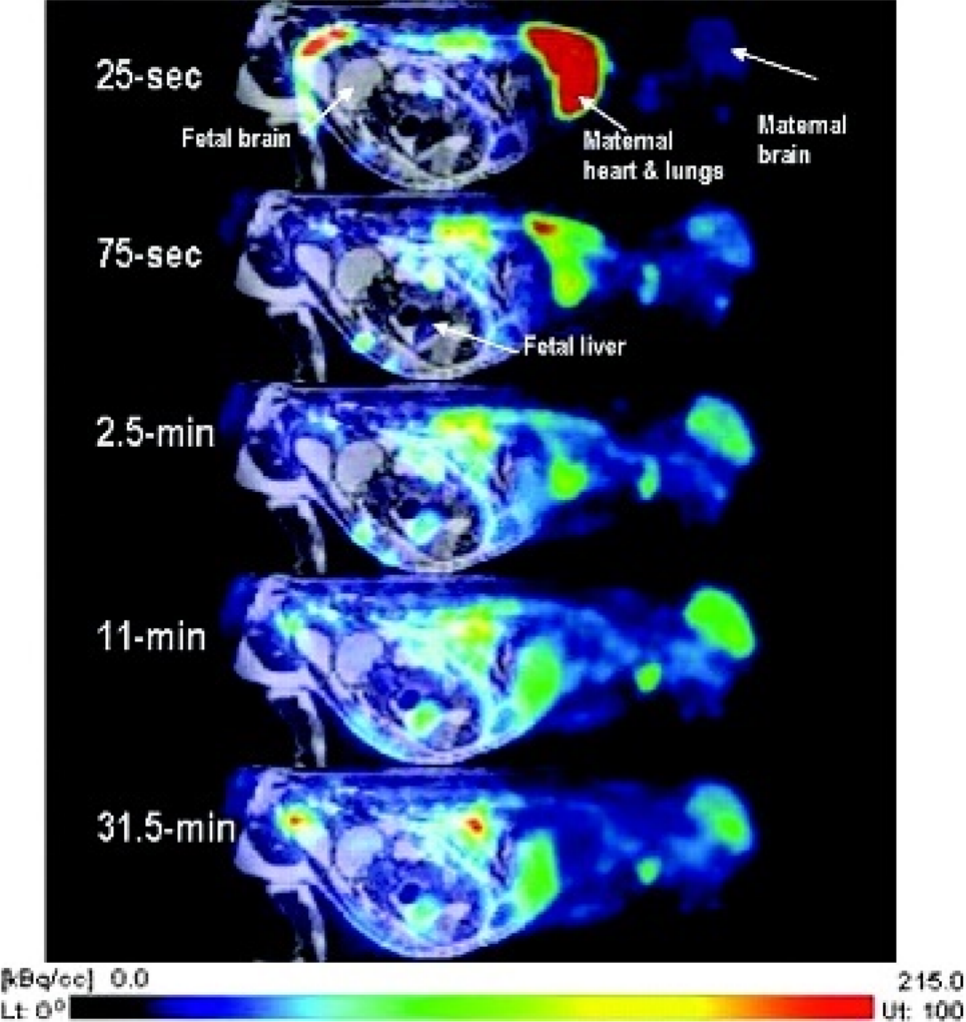
[*N*-*Methyl*-^11^C]cocaine pharmacokinetics of third-trimester pregnant macaques. Reproduced with permission from ref (100). Copyright 2005 Society of Nuclear Medicine. This work is licensed under a Creative Commons Attribution 4.0 International License (https://creativecommons.org/licenses/by/4.0/).

In 2005, Benveniste *et al*. performed biodistribution studies in six pregnant female bonnet macaques (*Macaca radiata*) directly after administration of [*N*-*methyl*-^11^C]cocaine *via* the saphenous vein.^100^ Significant accumulation was observed in the fetal liver at 5.5 min p.i., while fetal brain uptake was slower than maternal brain uptake. [*N*-*Methyl*-^11^C]cocaine uptake in the maternal heart was 15 times higher than in the fetal heart at 4.5 min p.i. Peak uptake in maternal kidneys occurred at 1–2 min, with half activity remaining at 15 min (Figure 13).^100^

In 2008, Kimmel *et al*. performed PET imaging studies of [^11^C]cocaine (without clarifying the label position) in awake rhesus monkeys to measure drug uptake in the brain following iv administration.^101^ Results showed that uptake in putamen was higher than in the cerebellum, with a peak at 9.5 min and dropping markedly by 40–50 min.

In 2009, Du *et al.* performed biodistribution studies of (−)-[*N*-*methyl*-^11^C]cocaine in six female Sprague-Dawley rat brains to study the effects of commonly used anesthetics.^94^

**Figure 14:**
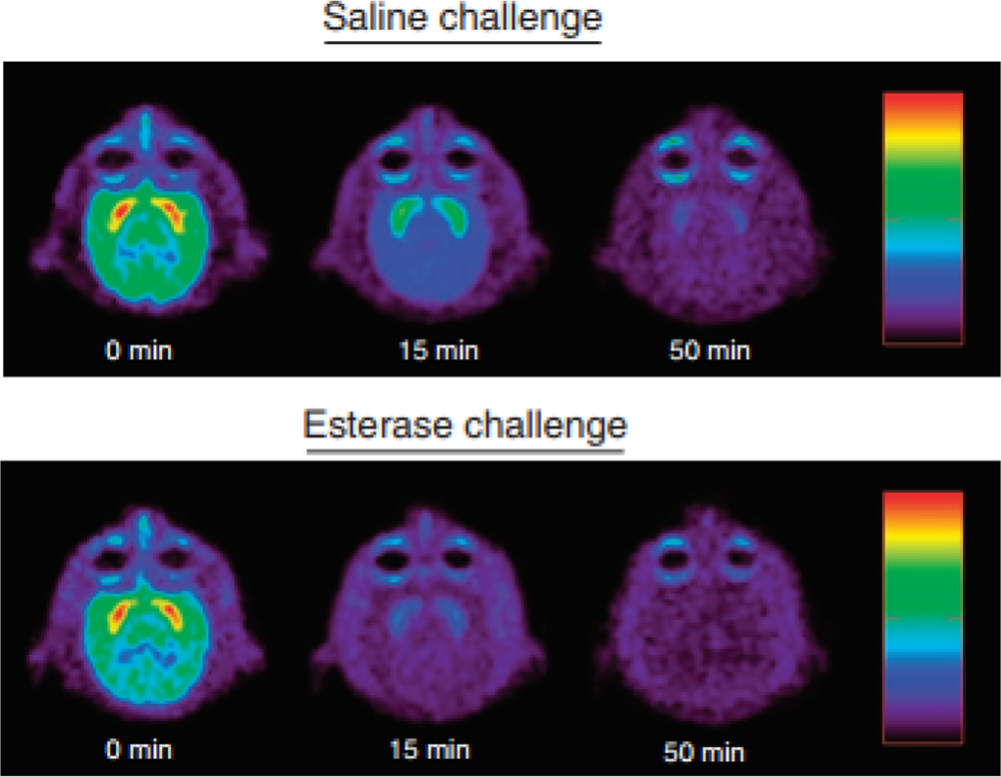
[*N*-*Methyl*-^11^C]cocaine pharmacokinetics in a rhesus monkey’s brain. Reproduced with permission from ref (102). Copyright 2014 Springer Nature. This work is licensed under a Creative Commons Attribution-NonCommercial-NoDerivs 3.0 Unported License (http://creativecommons.org/licenses/by-nc-nd/3.0/).

In 2014, Howell *et al*. used PET to study effects of cocaine esterase administration on [^11^C]cocaine uptake in three rhesus monkeys’ brains, finding that cocaine can be eliminated rapidly (Figure 14).^102^

#### 3.3.3 Clinical Studies

**Figure 15:**
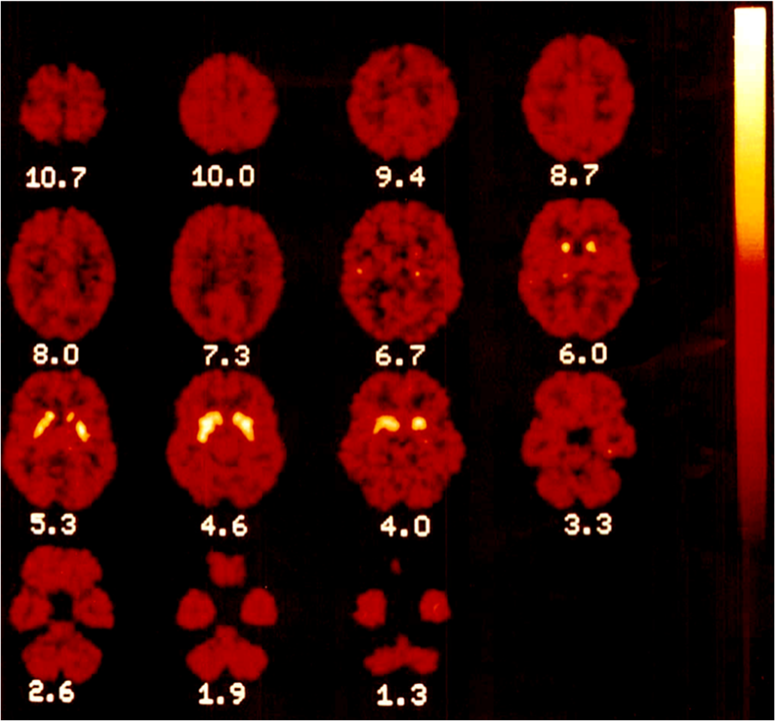
[*N*-*Methyl*-^11^C]cocaine regional distribution at 15 min p.i. in healthy human brains. Reproduced with permission from ref (95). Copyright 2004 John Wiley and Sons.

(−)-[*N*-*Methyl*-^11^C]Cocaine was administered intravenously *via* the saphenous vein to measure cocaine binding in the brain of six healthy male volunteers in two tracer doses within 2–3 h time periods.^95^ Highest uptake in the human brain was observed in the corpus striatum (4–10 min p.i.) (Figure 15).

**Figure 16:**
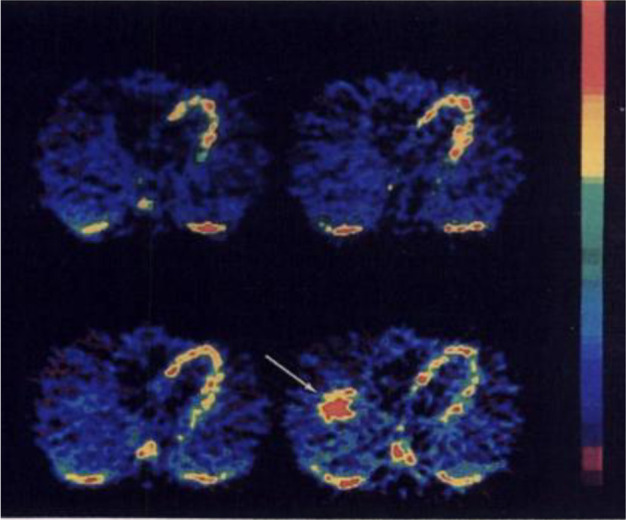
(−)-[*N*-*Methyl*-^11^C]cocaine thoracic distribution showed *via* four contiguous axial planes of the heart (2–10 min p.i.). The arrow highlights liver uptake in the bottom-right image. Reproduced with permission from ref (160). Copyright 1992 Society for Nuclear Medicine. This work is licensed under a Creative Commons Attribution 4.0 International License (https://creativecommons.org/licenses/by/4.0/).

Kinetic studies were performed on 14 healthy male volunteers by iv administration of (−)-[*N*-*methyl*-^11^C]cocaine (Figure 16).^160^ The highest accumulation was observed in the human heart, kidneys, adrenals, and liver. Another clinical study investigated if cocaine uptake in the human brain and heart (7 healthy humans) is influenced by the presence of alcohol.^161^ A cocaine uptake study showed a difference in the uptake values of (−)-[*N*-*methyl*-^11^C]cocaine between the brains of healthy volunteers (20 males) and detoxified cocaine abusers (12 males) but no difference between dopamine transporter availability.^162^ The same group has performed many [*N*-*methyl*-^11^C]cocaine studies in the human brain,^163−169^ published in a review.^170^

### 3.4 Codeine

#### 3.4.1 Radiosynthesis

**Figure 17:**
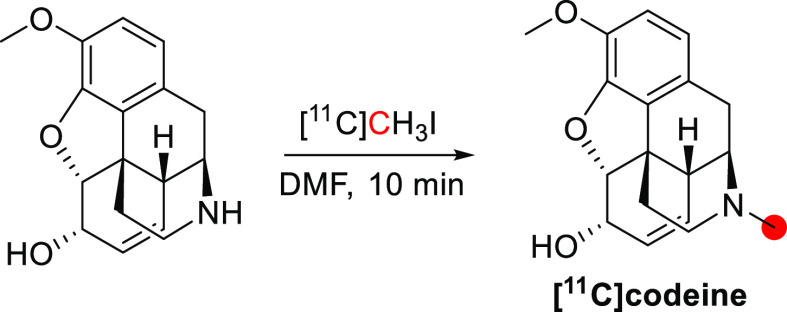
Synthesis of [^11^C]codeine using [^11^C]CH_3_I. ^11^C radionuclide position is highlighted in red.

[*N*-*Methyl*-^11^C]codeine was synthesized using [^11^C]CH_3_I in DMF with a RCY of 15–25% within 5–10 min from [^11^C]CH_3_I addition (Figure 17).^104^

#### 3.4.2 Preclinical Studies

Two rhesus monkeys (*Macaca mulatta*) were imaged after overnight fasting. The radioactivity uptake in the brain from [^11^C]codeine peaked within 5 min. The radioactivity was about 20% higher in gray matter compared to white, probably reflecting higher blood flow in the gray matter. The uptake of codeine radioactivity in extracranial soft tissue was slow, and the normalized uptake was 1.5 and 1.6 in two monkeys.^104^

### 3.5 Colchicine

#### 3.5.1 Radiosynthesis

**Figure 18:**
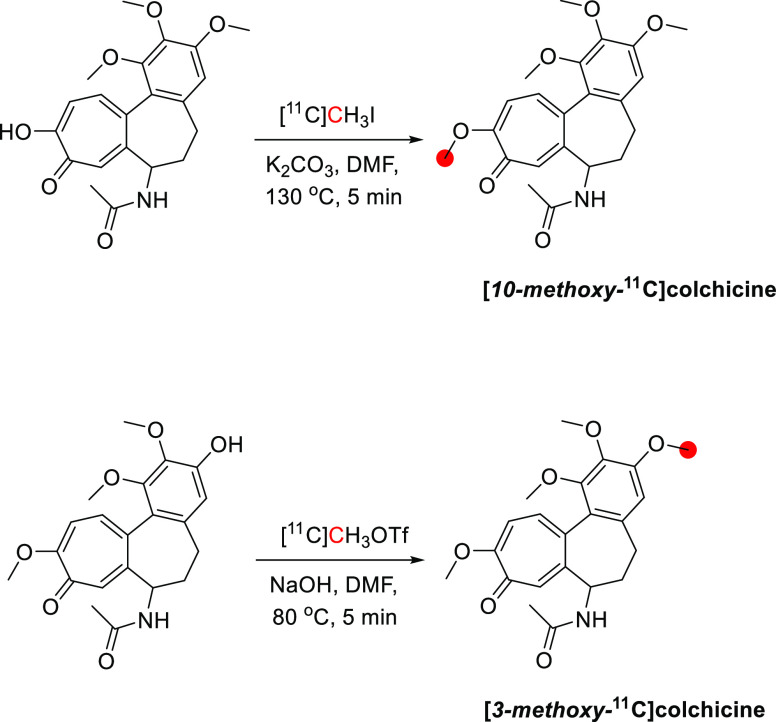
Synthesis of [*10*-*methoxy*-^11^C]colchicine and [*3*-*methoxy*-^11^C]colchicine using [^11^C]CH_3_I and [^11^C]CH_3_OTf. ^11^C radionuclide position is highlighted in red.

[*10*-*Methoxy*-^11^C]colchicine was synthesized by *O*-[^11^C]methylation of 10-desmethyl-colchicine with [^11^C]CH_3_I, with a RCY of 21% and *A*_m_ of 8.88 GBq/μmol in a process lasting 60 min (Figure 18).^108^ [3-*Methoxy*-^11^C]colchicine was radiolabeled by *O*-[^11^C]methylation at the 4-position using [^11^C]CH_3_OTf, with a non-decay correct (ndc) RCY of 0.5%, RCP >99% and *A*_m_ of 0.389 GBq/μmol (Figure 18).^105,106^

#### 3.5.2 Preclinical Studies

[^11^C]Colchicine was evaluated in mice,^105,107^ rats,^105,107^ and rhesus monkey.^105^ [*10*-*Methoxy*-^11^C]colchicine was evaluated in rodents to quantify P-glycoprotein (P-gp) mediated transport after retroorbital injection. Relatively high uptake was observed in the chest area, liver, kidney, and spleen, with low uptake in the brain. Also, the results suggest that [*10*-*methoxy*-^11^C]colchicine can image P-gp mediated transport in tumors (BE (2)-C cell line).^107^

**Figure 19:**
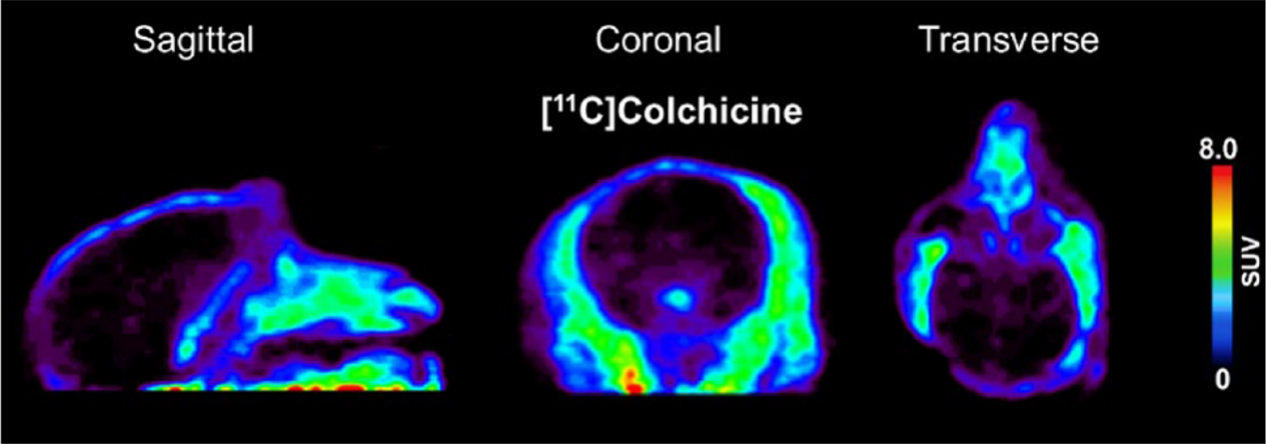
Sagittal, coronal, and transverse planes of [*3*-*methoxy*-^11^C]colchicine summed retention in rhesus monkey brain (between 6 and 60 min p.i.). Reproduced with permission from ref (105). Copyright 2021 Frontiers. This work is licensed under a Creative Commons Attribution 4.0 International License (https://creativecommons.org/licenses/by/4.0/).

In the rhesus monkey, [*3*-*methoxy*-^11^C]colchicine was administered intravenously into the hindlimb as a bolus over 1 min, and the brain was imaged for 90 min (Figure 19).^105^ The lack of brain uptake in non-human primates by [*3*-*methoxy*-^11^C]colchicine was expected as colchicine is a known P-gp substrate in rodents.^107^ Labeling in different positions (*3*-*methoxy*- *vs10*-*methoxy*-) showed no difference in the compound distribution.^105^

### 3.6 *N*,*N*-Dimethylphenethylamine

#### 3.6.1 Radiosynthesis

**Figure 20:**
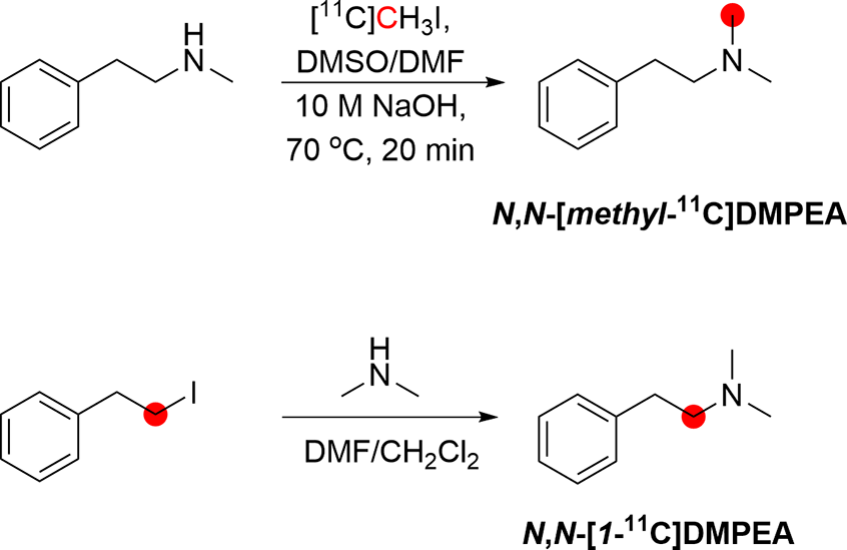
Synthesis of *N*,*N*-[*methy*l-^11^C]DMPEA and *N*,*N*-[*1*-^11^C]DMPEA using [^11^C]CH_3_I and [*1*-^11^C]phenethyl iodide, respectively. ^11^C radionuclide position is highlighted in red.

*N*,*N*-DMPEA has been labeled with carbon-11 in the methyl position^67,109,171^ and the phenethyl position.^109^*N*,*N*-[*Methyl*-^11^C]DMPEA was first synthesized by Inoue *et al*.^171^ in 1984 and further optimized by Shinoth *et al*.^67^ in 1987 and Halldin *et al*.^109^ in 1989 by reaction of *N*-methylphenethylamine with [^11^C]CH_3_I, with a total preparation time of 35 min from EOB (Figure 20). *N*,*N*-[*Methyl*-^11^C]DMPEA was isolated by semipreparative HPLC within 35 min with a RCY of 30–35%, RCP > 99%, and *A*_m_ of 3.7 GBq/μmol.

*N*,*N*-[*1*-^11^C]DMPEA was prepared by *N*-alkylation of dimethylamine with [*1*-^11^C]phenethyl iodide followed by HPLC purification, with a RCY of 10%, RCP >99%, and *A*_m_ of 0.37–3.7 GBq/μmol, in a 50 min process (Figure 20).^109^

#### 3.6.2 Preclinical Studies

*N*,*N*-[*Methyl*-^11^C]DMPEA was studied in mice,^67^ rats,^109^ and rhesus monkeys,^109^ while *N*,*N*-[*1*-^11^C]DMPEA in rats^109^ and rhesus monkeys.^109^ Male C3H mice were injected *via* the tail vein with *N*,*N*-[*methyl*-^11^C]DMPEA, which was transported into most organs with the highest uptake in the kidney and was cleared rapidly from the blood. A slightly lower uptake was observed in the brain and the lung. In the brain, the uptake reaches its peak 1 min after injection, followed by a rapid clearance at 1–5 min, and the activity in the blood decreases to lower levels than that seen in the brain.^67^ In a biodistribution study in rats, Halldin *et al*. found that the radioactivity concentration in the brain was significantly lower for *N*,*N*-[*1*-^11^C]DMPEA than *N*,*N*-[*methyl*-^11^C]DMPEA at 45 min post iv injection.^109^

*N*,*N*-[*Methyl*-^11^C]DMPEA and *N*,*N*-[*1*-^11^C]DMPEA were studied in the rhesus monkey using PET.^109^ Following iv administration, initial rapid uptake was observed in the brain for both tracers. *N*,*N*-[*methyl*-^11^C]DMPEA uptake remained constant over 30 min, while *N*,*N*-[*1*-^11^C]DMPEA cleared from the brain, reflecting the fate of the corresponding radiolabeled metabolites formed.^109^

#### 3.6.3 Clinical Studies

**Figure 21:**
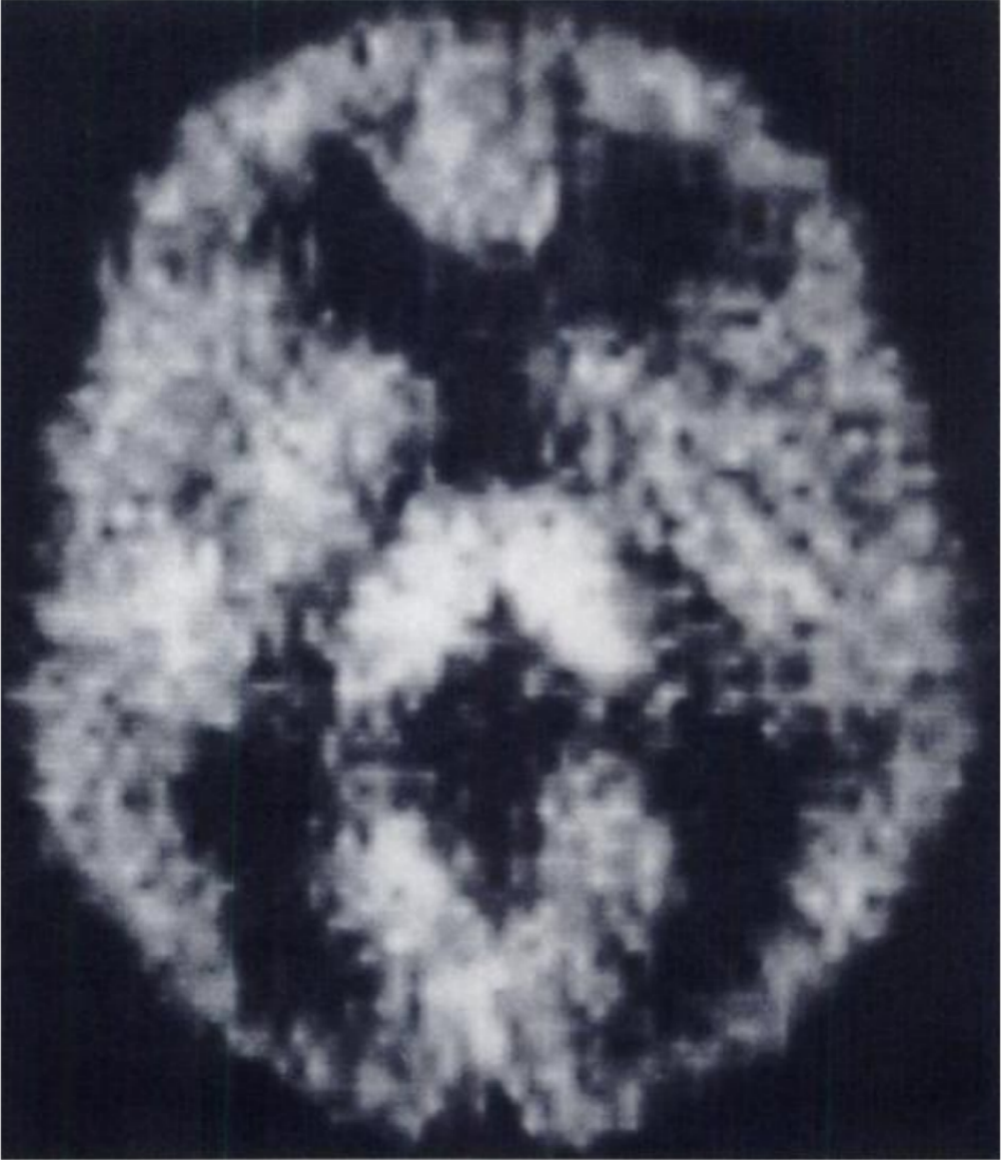
*N*,*N*-[*Methyl*-^11^C]DMPEA summed retention in a healthy volunteer (between 3 and 30 min p.i.). Reproduced with permission from ref (67). Copyright 1987 Society of Nuclear Medicine. This work is licensed under a Creative Commons Attribution 4.0 International License (https://creativecommons.org/licenses/by/4.0/).

*N*,*N*-[*Methyl*-^11^C]DMPEA has been studied in four male volunteers, varying from 48 to 70 years old (Figure 21). A high and rapid accumulation of radioactivity in the brain within 4–6 min was observed, which gradually increased until the end of the experiment. High uptake was observed in the thalamus, basal ganglia, cerebral cortex, and cerebellum, with a moderate uptake in the brain stem. The radioactivity in the blood was much lower than that in the brain.^67^

### 3.7 *N*,*N*-Dimethyltryptamine

#### 3.7.1 Radiosynthesis

**Figure 22:**
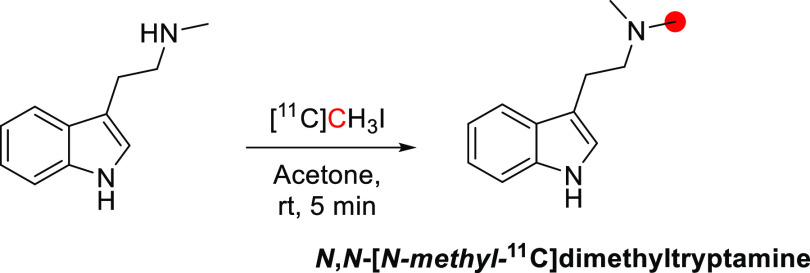
Radiosynthetic scheme for the synthesis of *N*,*N*-[*N*-*methyl*-^11^C]dimethyltryptamine. ^11^C radionuclide position is highlighted in red.

*N*,*N*-[*N*-*Methyl*-^11^C]dimethyltryptamine was achieved by the *N*-methylation of *N*-methyltryptamine with [^11^C]CH_3_I (Figure 22). The reaction was conducted in acetone at room temperature for 5 min, and the purification was achieved by a silica-gel column. *N*,*N*-[*N*-*Methyl*-^11^C]dimethyltryptamine was obtained starting from [^11^C]CH_3_I with 50% of RCY in 35 min from the end of [^11^C]CH_3_I trapping with a RCP of 99%.^90^ Subsequently, the same reaction was conducted at 50–60 °C, obtaining *N*,*N*-[*N*-*methyl*-^11^C]dimethyltryptamine with RCY 80–90% in 50 min. The *A*_m_ was reported to be 0.37–2.81 GBq/μmol.^110^

#### 3.7.2 Preclinical Studies

In healthy Wistar rats, *N*,*N*-[*N*-*methyl*-^11^C]dimethyltryptamine highly accumulated in the kidney, liver, spleen, and brain.^90^ The brain-to-blood ratio increased over time from 2.7 at 5 min to 6.5 at 60 min.^90^*N*,*N*-[*N*-*Methyl*-^11^C]dimethyltryptamine accumulated in the cerebral cortex, caudate-putamen, and amygdaloid nuclei, with traces also in the cerebellum and medulla oblongata.^110^ In dog brain, a displacement study with *O*-methylbufotenine revealed a substantial reduction in posterior cerebral cortex uptake, while a sharp decrease affected basal ganglia and frontal cortex, correlating well with the distribution and density of serotonin receptors.^110^

### 3.8 Ephedrine and *N*-*Methyl*-ephedrine

#### 3.8.1 Radiosynthesis

**Figure 23:**
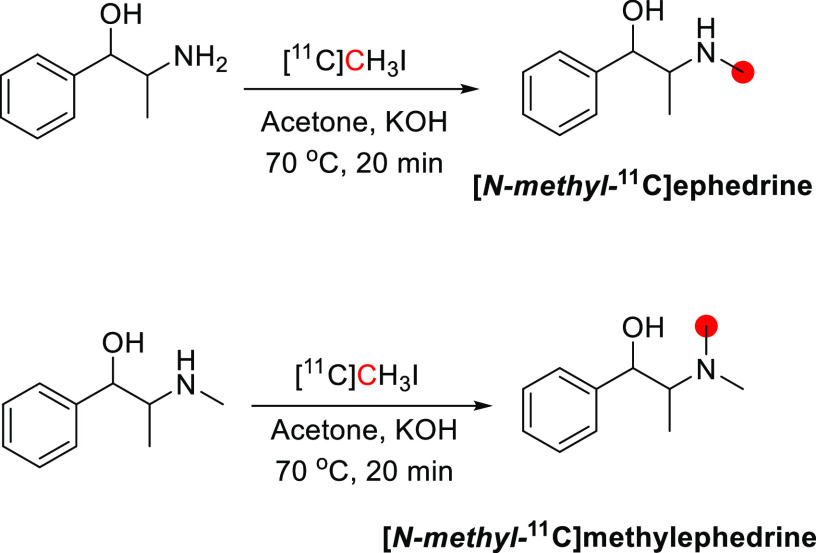
Radiosynthesis of [*N*-*methyl*-^11^C]ephedrine and [*N*-*methyl*-^11^C]methylephedrine using [^11^C]CH_3_I. ^11^C radionuclide position is highlighted in red.

The synthesis of [*N*-*methyl*-^11^C]ephedrine and [*N*-*methyl*-^11^C]methylephedrine were reported by Saji *et al*. in 1978 by the reaction of [^11^C]CH_3_I with norephedrine and ephedrine, respectively, in acetone with KOH at 70 °C for 20 min (Figure 23).^92^ [*N*-*Methyl*-^11^C]ephedrine was prepared in 45 min with a RCY of 11% and [*N*-*methyl*-^11^C]methylephedrine in 36 min with a RCY of 43%.^92^

#### 3.8.2 Preclinical Studies

The biodistribution of [*N*-*methyl*-^11^C]ephedrine and [*N*-*methyl*-^11^C]methylephedrine was evaluated *in vivo* after iv injection in mice.^92^ At 5 min p.i., both tracers showed considerable uptake in the liver and kidney and relatively high in the brain. Tracer uptake decreased over time, with the exception of [*N*-*methyl*-^11^C]methylephedrine in the adrenal glands, which tended to increase with time. [*N*-*Methyl*-^11^C]methylephedrine had a higher initial uptake in the brain compared to [*N*-*methyl*-^11^C]ephedrine, but its washout was quicker. If this difference is due to demethylation in the brain, [*N*-*methyl*-^11^C]methylephedrine may be of interest as a diagnostic agent for healthy brain function; however, further investigations are needed to confirm these observations.^92^

### 3.9 Galanthamine

#### 3.9.1 Radiosynthesis

**Figure 24:**
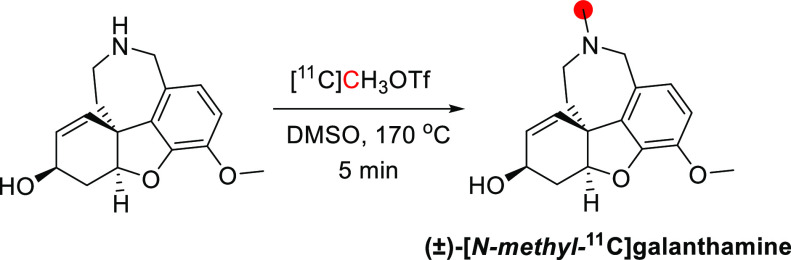
Radiosynthesis of (±)-[*N*-*methyl*-^11^C]galanthamine from norgalanthamine and [^11^C]CH_3_OTf. ^11^C radionuclide position is highlighted in red.

^11^C was incorporated into [*N*-*methyl*-^11^C]galanthamine by *N*-methylation of norgalanthamines with [^11^C]CH_3_OTf (Figure 24). The RCYs of (−)- and (*+*)-[*N*-*methyl*-^11^C]galanthamine were 13.7 and 14.4%, respectively, with a RCP >99%.^111^

#### 3.9.2 Preclinical Studies

**Figure 25:**
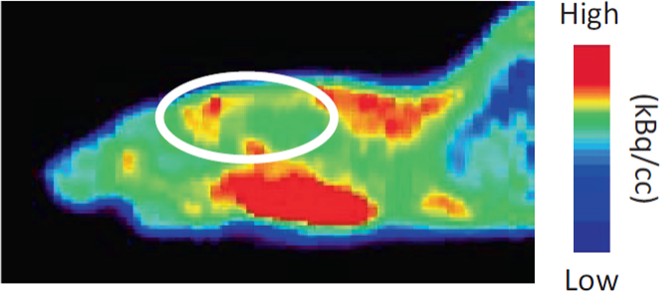
(−)-[*N*-*Methyl*-^11^C]galanthamine PET scan of a ddY mouse (time) with the circle highlighting the brain position. Reproduced with permission from ref (111). Copyright 2014 Elsevier.

Both (−)-[*N*-*methyl*-^11^C]galanthamine and (*+*)-[*N*-*methyl*-^11^C]galanthamine were studied in mice and rats.^111^ In a biodistribution study in male ddY mice, both isomers were found to be brain penetrant, reaching maximum uptake at 10 min p.i. (−)-[*N*-*Methyl*-^11^C]galanthamine showed more significant accumulation in the striatum than in the cerebellum. Pretreatment with donepezil led to a significant decrease in the accumulation of (−)-[*N*-*methyl*-^11^C]galanthamine but did not affect the striatal accumulation of (*+*)-[*N*-*methyl*-^11^C]galanthamine. PET imaging revealed the localization of (−)-[*N*-*methyl*-^11^C]galanthamine in the mouse striatum, which coincided with the localization of AChE in the brain as determined by immunostaining and *in vitro* autoradiography in rat brain tissue (Figure 25). These results indicate that (−)-[*N*-*methyl*-^11^C]galanthamine showed specific binding to AChE and AChR, whereas (*+*)-[*N*-*methyl*-^11^C]-galanthamine accumulation was nonspecific.^111^

### 3.10 Harmine and Harmaline

#### 3.10.1 Radiosynthesis

**Figure 26:**
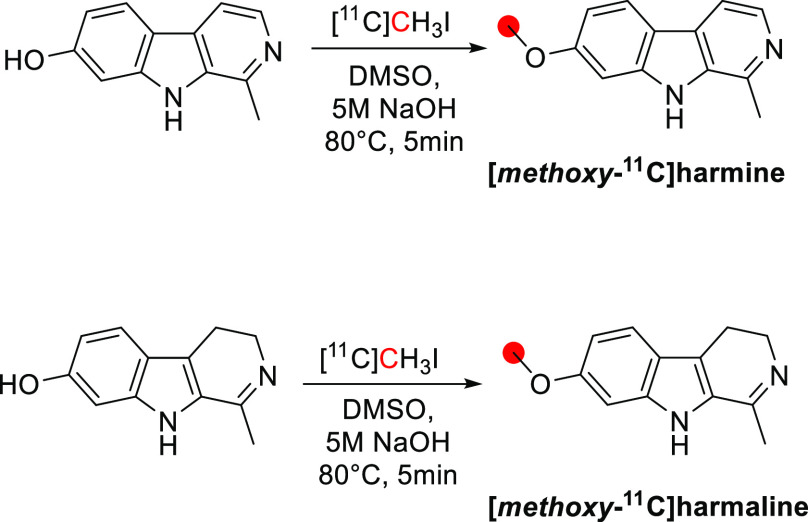
Radiosynthetic schemes of [*methoxy*-^11^C]harmine and [*methoxy*-^11^C]harmaline. ^11^C radionuclide position is highlighted in red.

[*Methoxy*-^11^C]harmine was synthesized by the *O*-methylation of harmol (normethylharmine) with [^11^C]CH_3_I. The reaction was conducted in DMSO with NaOH at 80 °C for 5 min (Figure 26). [*Methoxy*-^11^C]harmaline was synthesized using a similar procedure starting from normethylharmaline. Radiosynthesis, HPLC purification, and formulation were accomplished in 43 and 40 min with RCY of 72.5 ± 3.6% and 65.9 ± 9.7%, respectively. *A*_m_ at the end of synthesis (EOS) was around 18.0–87.3 GBq/μmol, and RCP > 98%.^112^ [*Methoxy*-^11^C]harmine was also prepared in a captive solvent method using an HPLC loop with DMF as the solvent and (C_4_H_9_)_4_NOH in CH_3_OH as a base (Figure 26).^172^ More recently, a fully-automated radiosynthesis was reported, producing [*methoxy*-^11^C]harmine with a RCY of 51 ± 11% and *A*_m_ of 101.32 ± 28.2 GBq/μmol, with a 2 min reaction time and a total process time of 35 min from EOB.^121^

#### 3.10.2 Preclinical Studies

**Figure 27:**
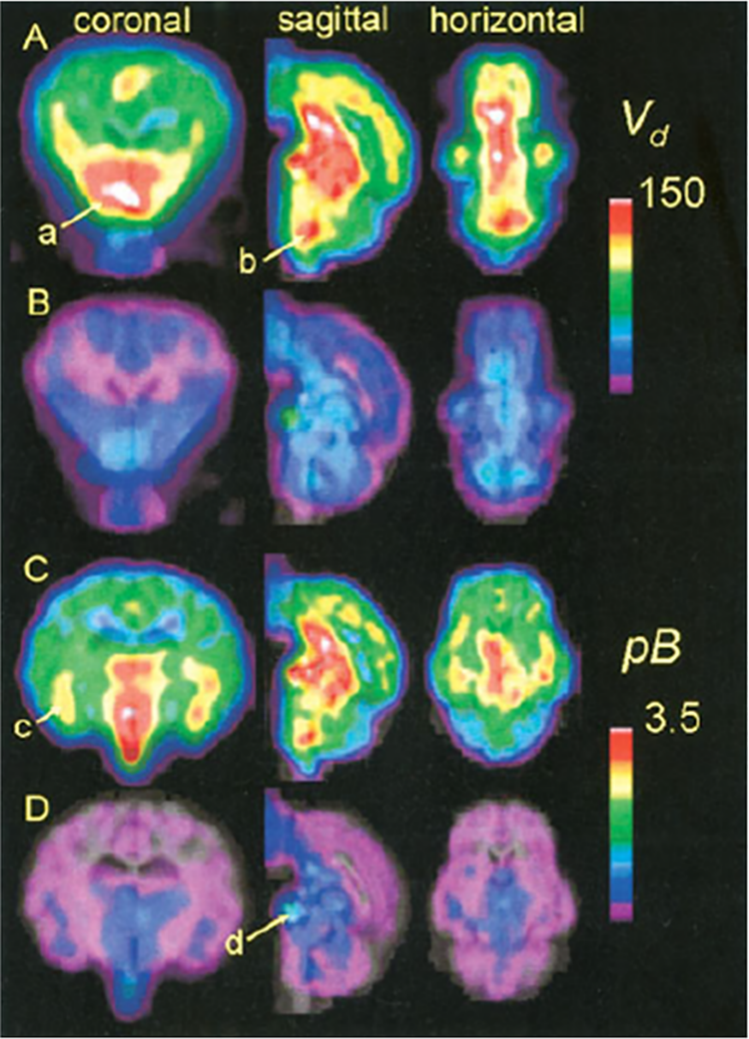
[*Methoxy*-^11^C]harmine means distribution volume (*V*_d_, mL/g, A,B) and binding potential (pB, C,D) in Gottingen minipigs brain with (B,D) and without (A,C) acute pargyline treatment (90 min p.i.). Regions of interest: (a) the ventral forebrain, (b) vicinity of the locus coeruleus, (c) the amygdale and hippocampal formation, and (d) the pituitary gland. Reproduced with permission from ref (115). Copyright 2006 John Wiley and Sons.

PET evaluation in rhesus monkeys revealed a higher brain uptake of [*methoxy*-^11^C]harmine than [*methoxy*-^11^C]harmaline at 54 min p.i. Displacement with MAO-A inhibitors strongly decreased [^11^C]harmine uptake.^112^ Generally, [^11^C]harmine showed rapid uptake in all grey matter regions. After pretreatment with MAO-A inhibitors, its initial brain uptake doubled, followed by a more rapid washout and less differentiation between grey and white matter.^113^ A PET biodistribution study in baboons demonstrated very high uptake of [*methoxy*-^11^C]harmine in the lungs, followed by kidneys, small intestine, and liver. Dosimetry data indicates the tracer elimination *via* the hepatobiliary and renal system, probably due to *O*-demethylation.^114^ In minipigs [*methoxy*-^11^C]harmine was widely retained in all brain regions, directly correlating to MAO-A distribution. High uptake was found in the dorsal striatum, ventral forebrain, and medulla (Figure 27).^115^

#### 3.10.3 Clinical Studies

In healthy humans, [*methoxy*-^11^C]harmine was rapidly metabolized, accounting for <50% of the radioactivity in plasma at 20 min p.i. The regional distribution of [*methoxy*-^11^C]harmine uptake in the brain was consistent with known MAO-A distribution, revealing high radioactivity in the thalamus followed by striatum and cortical regions. Treatment with MAO-A inhibitors such as moclobemide induces enzyme blocking at the peripheral site resulting in increased radioactivity in the brain.^172^ Due to its promising properties as a CNS MAO-A imaging agent, it was further evaluated for binding quantification and measuring the effects of competing substrates in healthy volunteers.^116,117^

**Figure 28:**
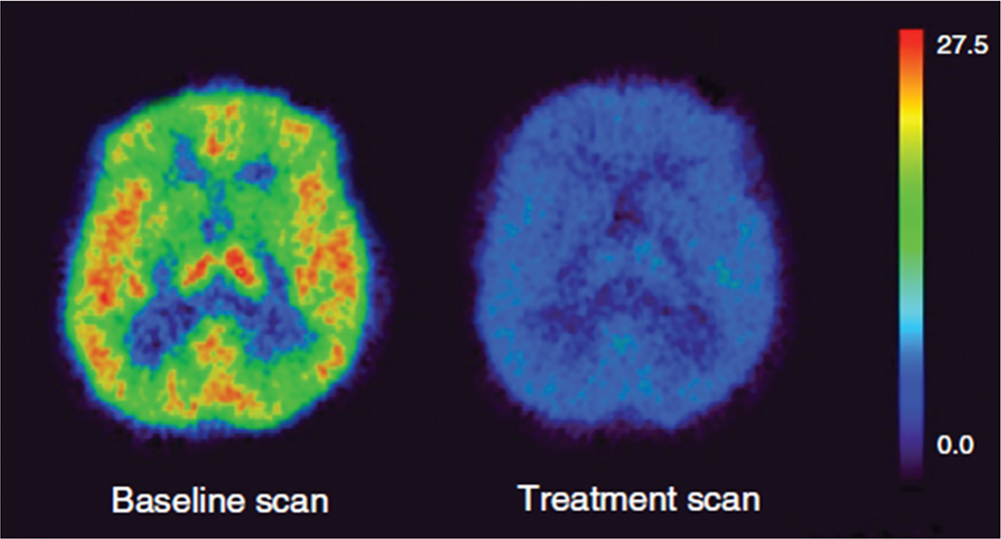
[*Methoxy*-^11^C]harmine PET scans of a depressed patient’s brain at baseline and 6 weeks post moclobemide treatment. Reproduced with permission from ref (118). Copyright 2011 Canadian Medical Association.

[*Methoxy*-^11^C]harmine has been evaluated as a radiotracer to detect MAO-A levels in neuropsychiatric disorders. In people affected by antisocial personality disorder, [*methoxy*-^11^C]harmine PET analyses revealed reduced functional MAO-A levels in all brain regions investigated (*e.g*., orbitofrontal and prefrontal cortex).^173^ In patients with major depressive disorders following antidepressant therapy, [*methoxy*-^11^C]harmine was used to evaluate MAO-A occupancy and correlated with treatment efficacies (Figure 28). [*Methoxy*-^11^C]harmine was successfully used to visualize neuroendocrine gastroenteropancreatic tumors and mood disorders related to perimenopause.^119,120^

### 3.11 Morphine

#### 3.11.1 Radiosynthesis

**Figure 29:**
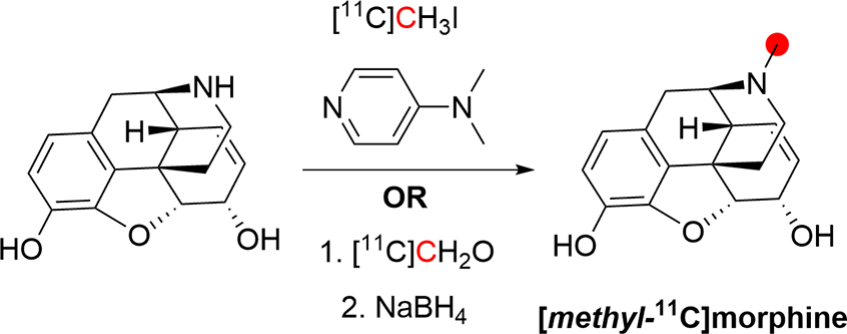
Radio synthesis of [*methyl*-^11^C]morphine using normorphine and [^11^C]CH_3_I or [^11^C]CH_2_O. ^11^C radionuclide position is highlighted in red.

In 1979, the radiolabeling of [*methyl*-^11^C]morphine was reported by two groups using different approaches to achieve ^11^C-methylation at the *N*-*methyl*- position.^122,174^ Kloster *et al.* reported the synthesis *via* reaction of normorphine with [^11^C]CH_3_I with a base in ethanol in 9% RCP in a process lasting 18 min.^122^ Allen and Beaumier^126^ performed a reductive alkylation of normorphine using [^11^C]CH_2_O and NaBH_4_ (Figure 29). In 1982, Långström *et al*. reported the synthesis using [^11^C]CH_3_I in DMF solution.^175^

In 2011, Fan *et al*. published the first automated radiosynthesis of [*methyl*-^11^C]morphine for clinical investigations *via* reaction of normorphine with [^11^C]CH_3_I in DMSO in the presence of NaOH.^125^ Total synthesis time was 45 min with a RCY of 45–50%, RCP >95%, and *A*_m_ of 740–962 GBq/μmol.^125^

#### 3.11.2 Preclinical Studies

Biodistribution studies of [*methyl*-^11^C]morphine in male Wistar rats showed accumulation of activity in the small intestine with very little activity in the brain.^122^ A preclinical PET study in five rhesus monkeys was performed to investigate brain kinetics. No specific localization of [*methyl*-^11^C]morphine was observed in the brain, and uptake was low, reaching a maximum at 30-45 min p.i., followed by slow clearance.^104^

In 1988, Agon *et al*. performed a PET study in adult mongrel dogs using [*methyl*-^11^C]morphine to probe the effects of BBB disruption on tracer distribution.^123^ The ^11^C-labeled morphine was administered at the left femoral vein in two doses; first, 20 min after the injection of Evans blue as a reference for the osmotic opening of BBB, and the second 2 min after saline-mannitol administration.

**Figure 30:**
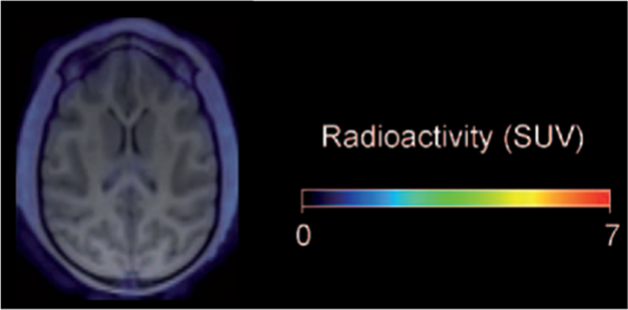
[*Methyl*-^11^C]morphine summed retention in monkey’s brain (between 3 and 93 min). Reproduced with permission from ref (93). Copyright 2015 Oxford University Press.

PET studies of [*methyl*-^11^C]morphine in pregnant rhesus monkeys (120–150 days pregnant) were performed after an iv injection of the tracer. Activity in the placenta reached a maximum within a few minutes, and rapid fetal liver accumulation was observed.^105^ In 1989, [*methyl*-^11^C]morphine was imaged in the spinal canal of nine rhesus monkeys. The tracer was administered at different spinal cord levels at different times, and kinetic measurements were performed up to 120 min post-tracer administration with plasma and cerebrospinal fluid samples.^124^ In 2015, Schou *et al*. studied the brain exposure of [*methyl*-^11^C]morphine after intravenous injection to female cynomolgus and rhesus monkeys.^93^ [*Methyl*-^11^C]morphine was injected at two different dose levels; one at tracer levels (microdose) and one at pharmacological doses; however, the brain uptake was very low (Figure 30).^93^

### 3.12 Nicotine

#### 3.12.1 Radiosynthesis

**Figure 31:**
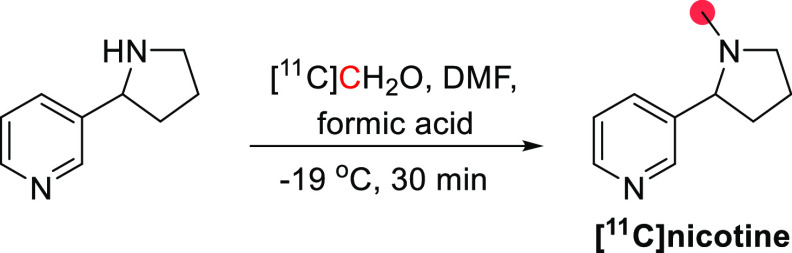
Synthesis of [^11^C]nicotine using [^11^C]CH_2_O. ^11^C radionuclide position is highlighted in red.

The synthesis of [^11^C]nicotine was first reported by Maziere *et al*. in 1976 by reaction of nor-(−)-nicotine with [^11^C]CH_2_O in DMF and formic acid at −19 °C, obtaining 370–555 MBq in 30 min and *A*_m_ of 1.11 GBq/μmol (Figure 31).^127^ In 2017, Garg *et al*. reported the synthesis *via* reaction of *nor*-nicotine biscamsylate with [^11^C]CH_3_OTf and 1,2,2,6,6-pentamethyl piperidine in acetonitrile, obtaining a RCY of ∼30% and *A*_m_ of 0.26 GBq/μmol at EOS.^131^ Xu *et al*. also used [^11^C]CH_3_OTf but with *nor*-nicotine free base in acetone at 45 °C, achieving a RCY of 60.4 ± 4.7% and *A*_m_ of 648 GBq/μmol, with a total synthesis time of 32–36 min.^134^ More recently, Ghosh *et al.* developed an automated loop method to obtain [^11^C]nicotine in a RCY of 19-35% and an *A*_m_ of 3.89 GBq/μmol, with a total process time of <30 min.^135^

#### 3.12.2 Preclinical Studies

An initial study by Maziere *et al*. investigated the distribution of [^11^C]nicotine in mice and rabbits.^127^ In mice, a radioactivity build-up in all organs (except liver and spleen) was observed within 5 min p.i. In the brain, maximum uptake was observed at 5 min, decreasing to almost half at 15 min p.i. In rabbits, rapid radiotracer accumulation in the brain was also observed, suggesting that [^11^C]nicotine instantaneously passes through the BBB. The fast drop in the brain radioactivity was thought to be due to the rapid oxidation of [^11^C]nicotine to [^11^C]cotinine, which has a poor affinity to nicotine targets.^127^ These results are also similar to those of rhesus monkeys obtained by Nordberg *et al.*, where the uptake in the brain peaked within 1–2 min of radiotracer administration and then declined very sharply.^129^ The regional distribution of (*S*)-[^11^C]nicotine in the brain of mice was also studied, showing that the uptake was higher in the cortex, followed by the hippocampus, striatum, hypothalamus, and cerebellum at 5 min after injection.^128^

#### 3.12.3 Clinical Studies

**Figure 32:**
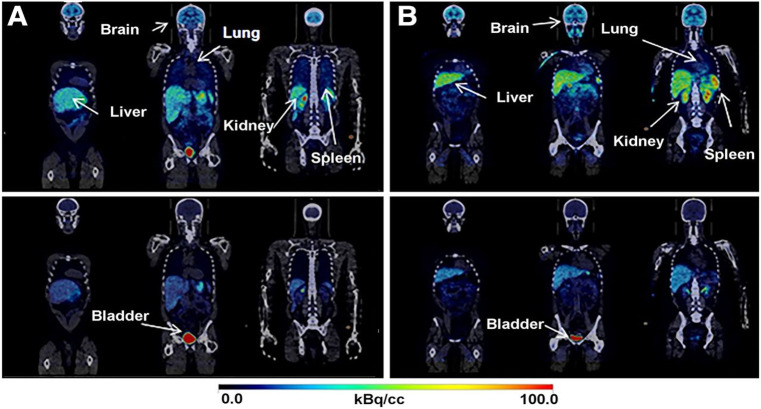
[^11^C]Nicotine whole-body PET images of (A) male and (B) female volunteers (top images recorded 2.8 min p.i.; bottom images recorded 22.6 min p.i.). Reproduced with permission from ref (131). Copyright 2017 Society of Nuclear Medicine and Molecular Imaging. This work is licensed under a Creative Commons Attribution 4.0 International License (https://creativecommons.org/licenses/by/4.0/).

Whole-body human PET scans of healthy nonsmokers showed rapid uptake of [^11^C]nicotine by most significant organs, including the heart, liver, brain, lungs, and muscle, followed by a drop in radioactivity after 22.6 min (Figure 32).^131^ The uptake in the muscle in humans was significantly higher compared to other organs. This can be due to the higher mass of muscle in comparison with the liver and spleen,^131^ similar to the observations mentioned in the rhesus monkey study.^129^

**Figure 33:**
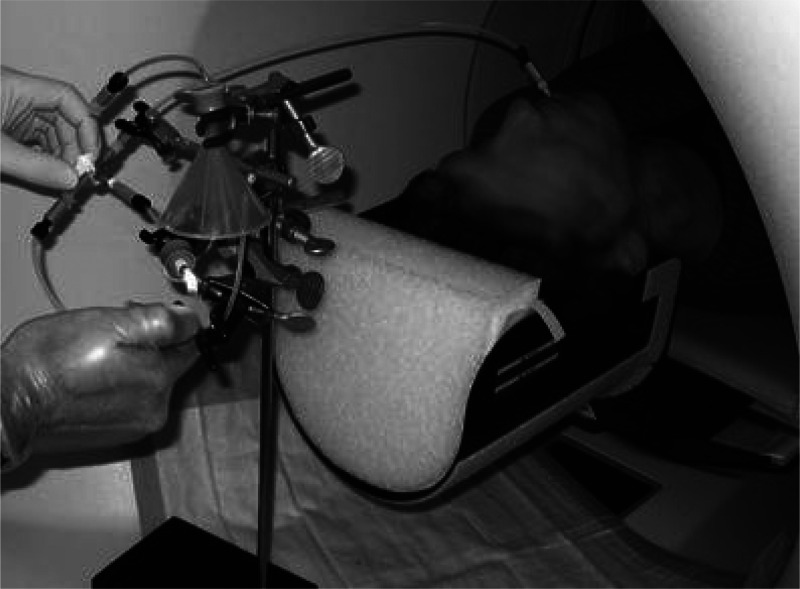
Set-up used for [^11^C]nicotine inhalation through cigarette smoke. Reproduced with permission from ref (176). Copyright 2010 Springer Nature.

Rose *et al*. studied the effect of smoking on the brain of smokers after single puffs of cigarettes formulated with [^11^C]nicotine by investigating the rate of nicotine entry into the brain.^132^ This study showed a rapid uptake of [^11^C]nicotine in the brain at approximately 5 s after inhalation, a possible explanation for the addictiveness of nicotine.^132^ However, another study compared the kinetics of [^11^C]nicotine in dependent and nondependent smokers after a single puff of smoke from a cigarette containing [^11^C]nicotine, showing that dependent smokers have a lower brain nicotine accumulation rate due to the slower nicotine washout from the lungs (Figure 33).^176^

**Figure 34:**
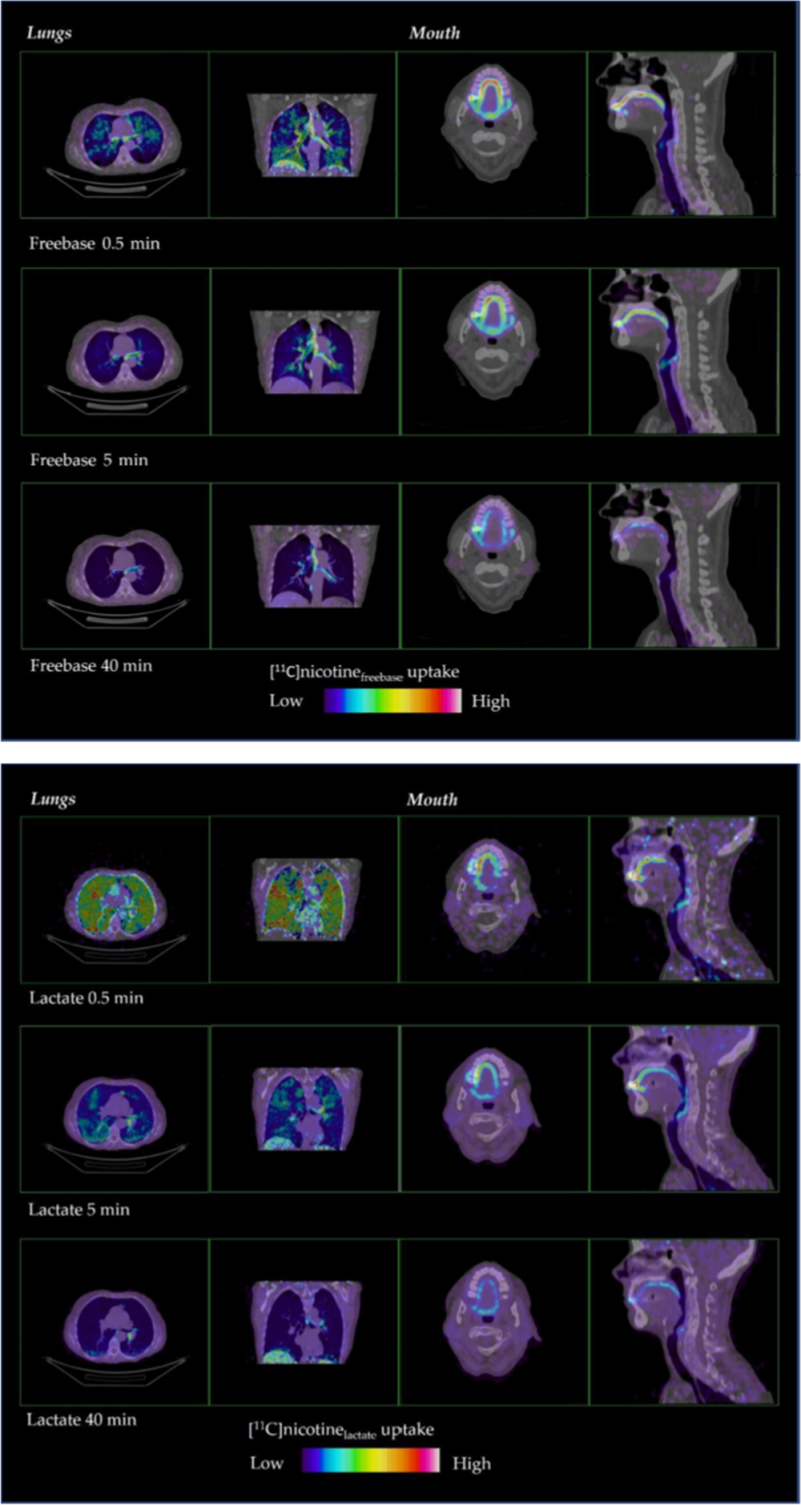
[^11^C]Nicotine_freebase_ (top) and [^11^C]nicotine_lactate_ (bottom) distribution in human lungs and mouth (0.5, 5, and 40 min after inhalation). Reproduced with permission from ref (133). Copyright 2022 MDPI. This work is licensed under a Creative Commons Attribution 4.0 International License (https://creativecommons.org/licenses/by/4.0/).

Recently, Wall *et al*. studied the distribution and accumulation of inhaled [^11^C]nicotine in the respiratory pathways and brain of 15 healthy adult smokers using the *my*blu e-cigarette with two nicotine formulations, freebase, and lactate salt.^133^ Over 30% of the inhaled tracer accumulated in the lung within 15–35 seconds. [^11^C]Nicotine_freebase_ exhibited higher uptake and deposition in the upper respiratory pathways than [^11^C]nicotine_lactate_. For [^11^C]nicotine_lactate_, brain deposition peaked at 4–5%, with an earlier peak and a steeper decline (Figure 34). The authors concluded that e-cigarettes with nicotine lactate formulations might contribute to greater adult smoker acceptance and satisfaction.^133^

### 3.13 Oxycodone

#### 3.13.1 Radiosynthesis

**Figure 35:**
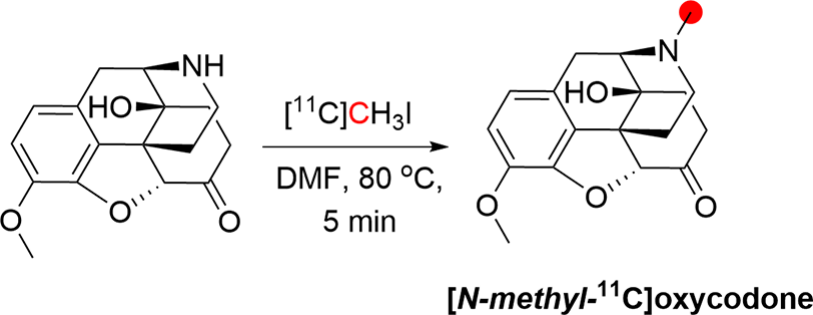
Radiosynthesis of [*N*-*methyl*-^11^C]oxycodone from *nor*-oxycodone and [^11^C]CH_3_I. ^11^C radionuclide position is highlighted in red.

[*N*-*Methyl*-^11^C]oxycodone was synthesized from noroxycodone hydrochloride by alkylation with [^11^C]CH_3_I (Figure 35), purified by semipreparative HPLC, and obtained with a RCP of >99% and *A*_m_ of 94.7 ± 13.2 kBq/μmol oxycodone (after addition of isotopically unmodified oxycodone) at the start of *in vivo* studies. The synthesis time from radionuclide production to the formulated product was approximately 35 min.^136^

#### 3.13.2 Preclinical Studies

**Figure 36:**
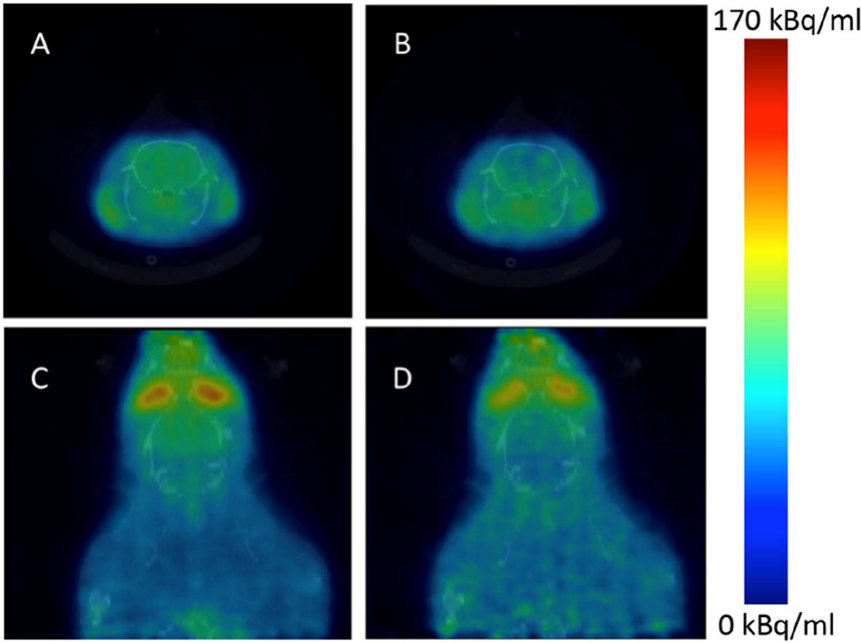
PET/CT scans of rats during a 60 min [*N*-*methyl*-^11^C]oxycodone and oxycodone infusion. parts A and C describe the infusion phase (30-60 min); parts B and D represent the elimination phase (65–120 min). Reproduced with permission from ref (136). Copyright 2017 Elsevier.

The brain pharmacokinetics of [*N*-*methyl*-^11^C]oxycodone have been investigated in Male Sprague-Dawley rats.^136^ Gustafsson *et al*. designed a combined PET and microdialysis analysis, where simultaneous sampling was performed throughout a 60 min infusion of [*N*-*methyl*-^11^C]oxycodone with a therapeutic dose of oxycodone.^136^ This design is a step toward building and verifying a translational concept of brain drug exposure and BBB transport from rodents to humans. [*N*-*Methyl*-^11^C]oxycodone was detected in the harderian glands and brain with increasing concentration during the infusion, followed by elimination immediately after the infusion (Figure 36). However, radioactivity accumulation became more pronounced at later time points, most likely due to the formation of radioactive metabolites, including [^11^C]carbonate, crossing into the brain.^136^

### 3.14 Papaverine

#### 3.14.1 Radiosynthesis

**Figure 37:**
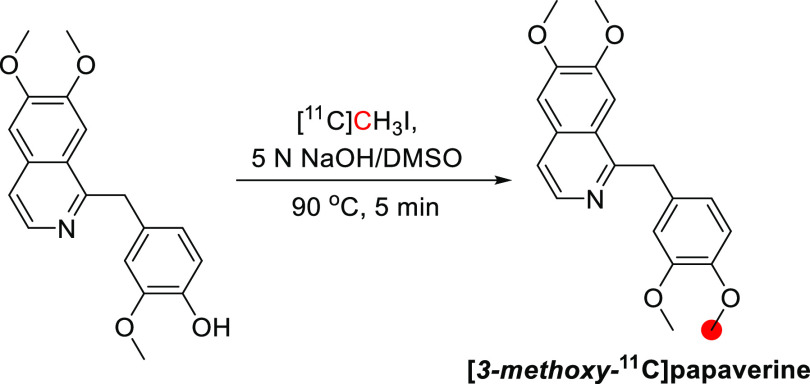
Radiosynthesis of [*3*-*methoxy*-^11^C]papaverine from *1*-(*4*-(benzyloxy)-*3*-methoxybenzyl)-*6*,*7*-dimethoxyisoquinoline and [^11^C]CH_3_I. ^11^C radionuclide position is highlighted in red.

The radiosynthesis of papaverine with ^11^C was achieved by *O*-methylation of the *1*-(*4*-(benzyloxy)-*4*-methoxybenzyl)-*6*,*7*-dimethoxyisoquinoline with [^11^C]CH_3_I (Figure 37). The entire synthetic procedure from the production of [^11^C]CH_3_I to the formulation of the radiotracer for *in vivo* studies was complete within 50–55 min. [*3*-*Methoxy*-^11^C]papaverine was obtained with RCY of ∼70%, *A*_m_ >740 GBq/μmol to EOB, and RCP of 99%.^137^

#### 3.14.2 Preclinical Studies

[*3*-*Methoxy*-^11^C]papaverine has been evaluated in adult male Sprague-Dawley rats and two adult male rhesus macaque monkeys.^137^*In vitro* autoradiography studies of rat and monkey brain sections revealed selective binding [*3*-*methoxy*-^11^C]papaverine to PDE10A enriched regions. The biodistribution in rats at 5 min demonstrated a high accumulation in the striatum, but the washout was rapid. PET imaging studies in rhesus macaques displayed similar initial high uptake in the striatum with very rapid clearance. However, Tu *et al*. suggested that [3-*methoxy*-^11^C]papaverine is not an ideal radioligand for clinical imaging of PDE10A in the CNS, and analogues of papaverine with a higher potency for inhibiting PDE10A and improved pharmacokinetic properties should be investigated for PET imaging.^137^

### 3.15 Physostigmine

#### 3.15.1 Radiosynthesis

**Figure 38:**
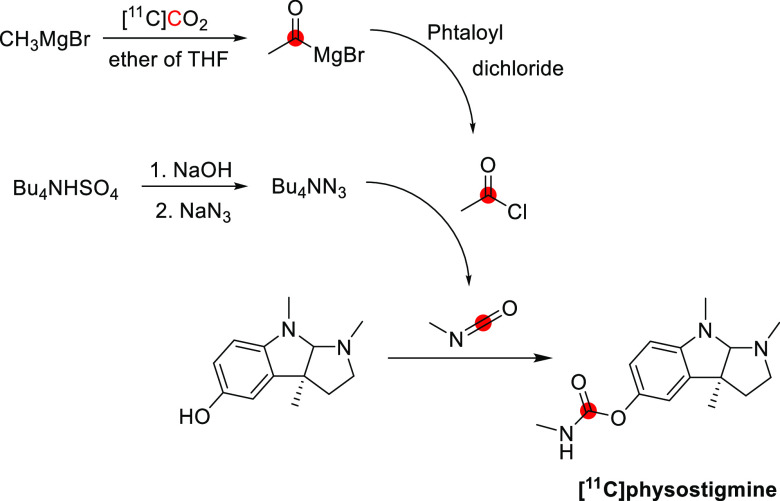
Synthesis of [^11^C]physostigmine by the reaction of [^11^C]methyl isocyanate with eseroline.^177^^11^C radionuclide position is highlighted in red.

[^11^C]Physostigmine has been radiolabeled at the carbonyl position *via* reaction of [^11^C]methyl isocyanate and eseroline, with the purified product obtained within 52 min, with an RCY of 12–19% and *A*_m_ of 11–13 GBq/μmol at EOB.^141−143^ In this process, [^11^C]methyl isocyanate was formed by heating [^11^C]CH_3_COCl with tetrabutylammonium azide in toluene within 10 min, then subsequently distilled into a solution of eseroline for 10 min at 25 °C (Figure 38), and the final product isolated by HPLC. [^11^C]Physostigmine has also been prepared by reaction of [^11^C]COCl_2_ on *N*,*N*-bis(trimethylsilyl)methylamine within 35 min with an RCY of 18–25% and an *A*_m_ of 25.9–39.6 GBq/μmol at EOB.^144^

#### 3.15.2 Preclinical Studies

**Figure 39:**
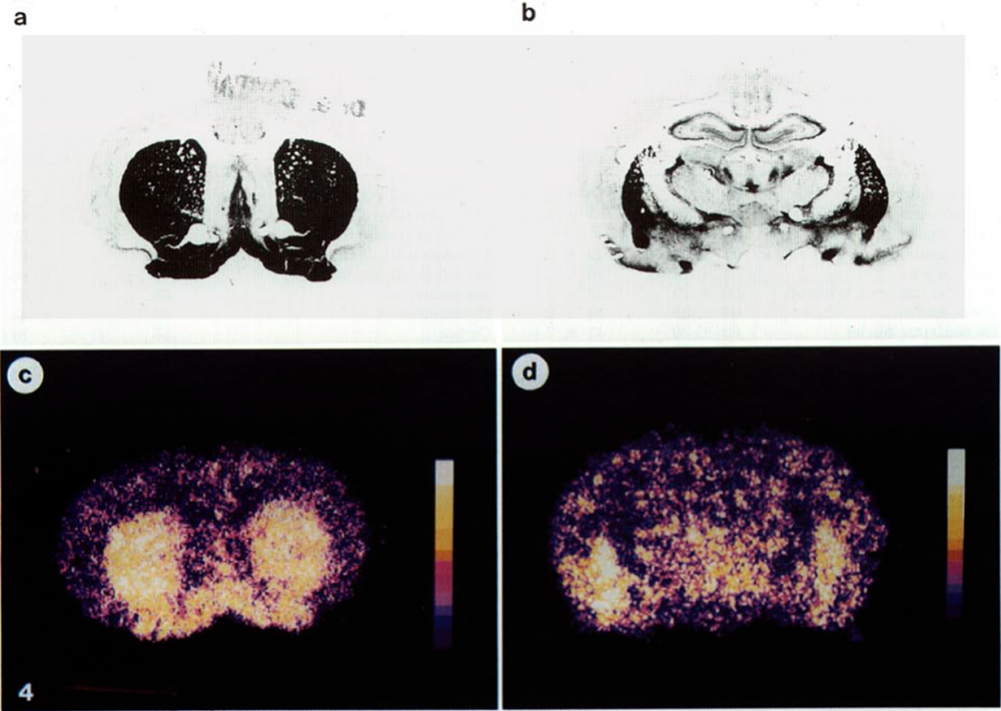
AChE stains (A,B) and corresponding autoradiograms (C,D) of two coronal slices from rat brain after [^11^C]physostigmine administration (10 min p.i.). Reproduced with permission from ref (138). Copyright 1994 Elsevier.

The cerebral distribution of [^11^C]physostigmine has been studied in rats^138^ and baboons.^139^ Male Sprague-Dawley rats were injected with [^11^C]physostigmine in the tail vein. In the rat brain, the radioactivity was significantly correlated to AChE activity, being highest in the basal ganglia, moderate in the cortex and hippocampus, and low in the cerebellum (Figure 39).^138^

**Figure 40:**
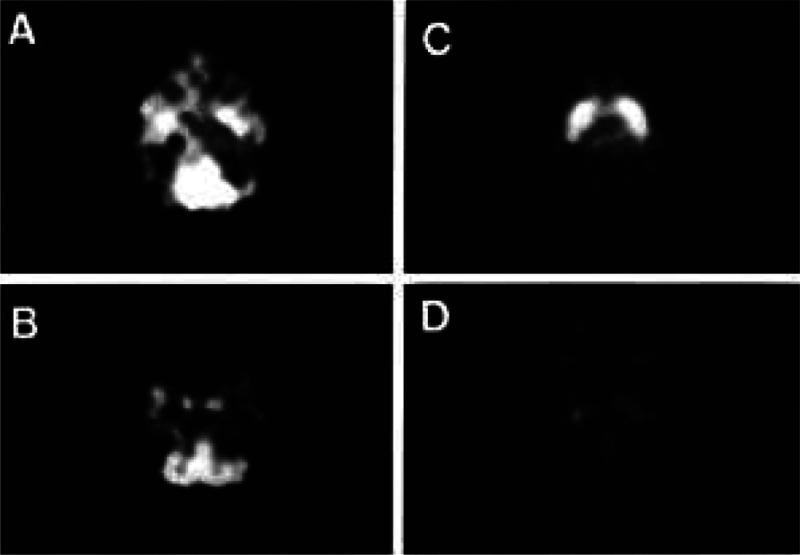
[^11^C]Physostigmine brain distribution in a baboon. (A,C) Striatal and (B,D) cerebellar imaging at early (A,B, 0-1 min p.i.) and late (C,D, 15–20 min p.i.) time points. Reproduced with permission from ref (141). Copyright 1993 Wolters Kluwer Health.

*In vivo* brain distribution and kinetics of [^11^C]physostigmine were obtained in baboons (*Papio papio*) after iv bolus injection of [^11^C]physostigmine (Figure 40).^139^ In the blood, the radioactivity peaked during the first minute and rapidly declined thereafter. An excess of unlabeled physostigmine in the brain significantly decreased the uptake of [^11^C]physostigmine in the striatum, indicating a high ratio of specific to nonspecific binding. Taking the white matter as the reference region, the ratio between the total distribution volumes in the target and reference regions showed a satisfactory correlation with the AChE concentration measured post-mortem in two baboon brains.^139^

#### 3.15.3 Clinical Studies

**Figure 41:**
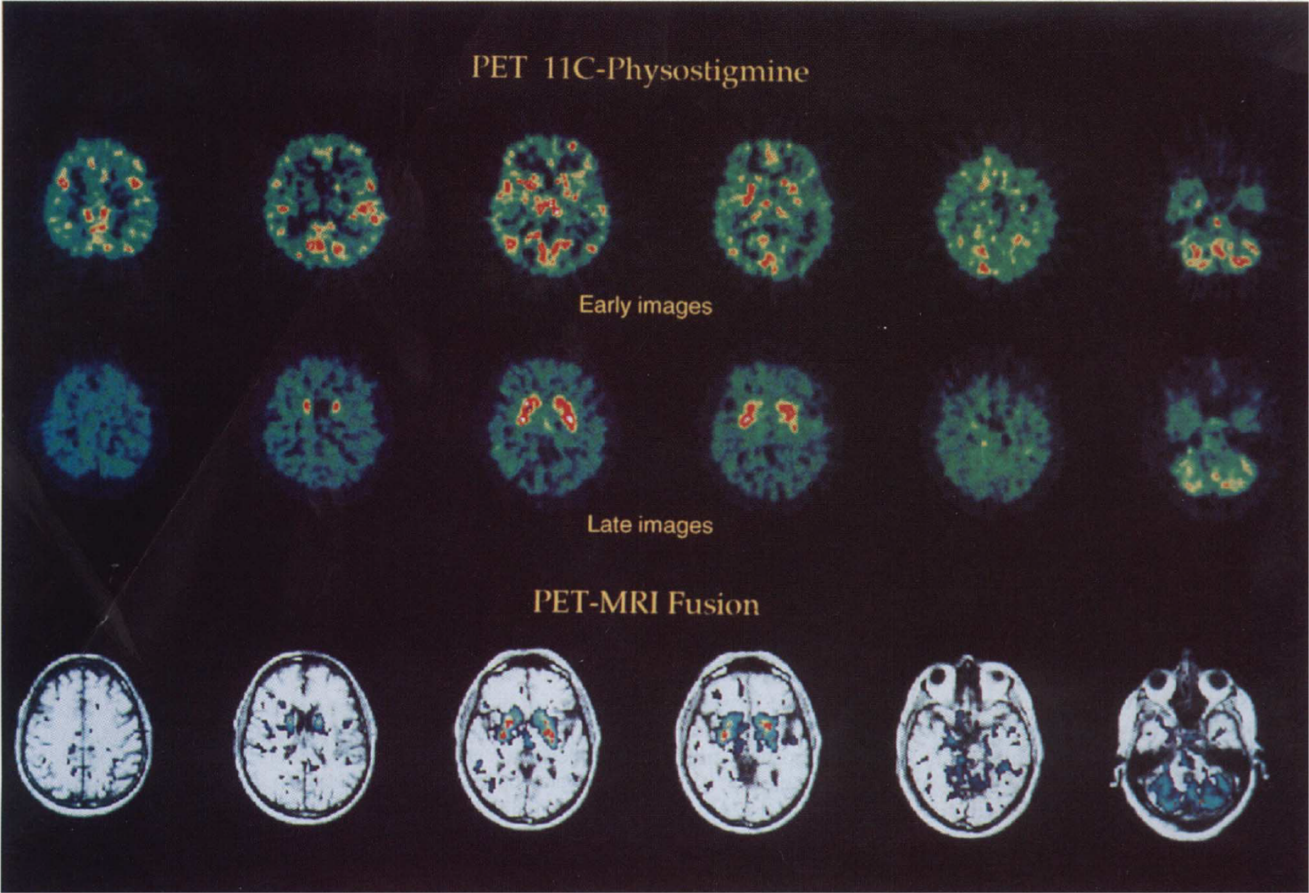
[^11^C]Physostigmine regional cerebral distribution in humans (early images = 0–4 min p.i.; late images = 25–35 min p.i.; PET-MRI fusion = 25–35 min p.i.). Reproduced with permission from ref (140). Copyright 2002 John Wiley and Sons.

The quantification of regional AChE developed using animal studies was applied to eight healthy male subjects (24–76 years old) after a bolus injection of [^11^C]physostigmine.^139^ The radioactivity rapidly crossed the BBB, reached a maximal level within a few minutes, and agreed with the known AChE concentrations measured in post-mortem studies of the human brain (Figure 41).^139^ These results suggest that PET studies with [^11^C]physostigmine can provide *in vivo* brain mapping of human AChE and are promising for studying changes in AChE levels associated with neurodegenerative diseases.^140^

### 3.16 Psilocin

#### 3.16.1 Radiosynthesis

**Figure 42:**
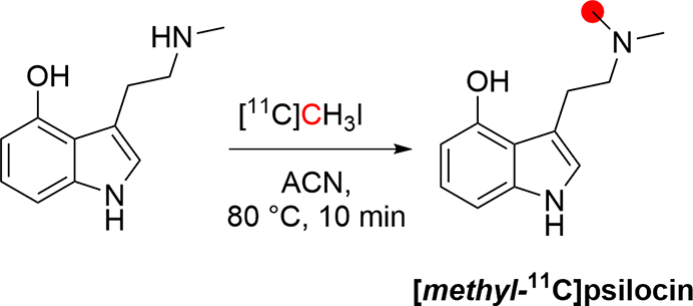
Radiosynthesis of [*N*-*methyl*-^11^C]psilocin. ^11^C radionuclide position is highlighted in red.

[*N*-*Methyl*-^11^C]psilocin was achieved by the *N*-methylation of 4-hydroxy-*N*-methyltryptamine with [^11^C]CH_3_I (Figure 42). The reaction was conducted in acetonitrile at 80 °C for 10 min, and the purification was achieved by a semipreparative reversed-phase HPLC. Radiosynthesis, purification, and formulation were accomplished in 45 min with RCY of 20 ± 5% and *A*_m_ of 33.3–85.1 GBq/μmol at EOS. RCP obtained was higher than 97%.^145^

### 3.17 Quinidine

#### 3.17.1 Radiosynthesis

**Figure 43:**
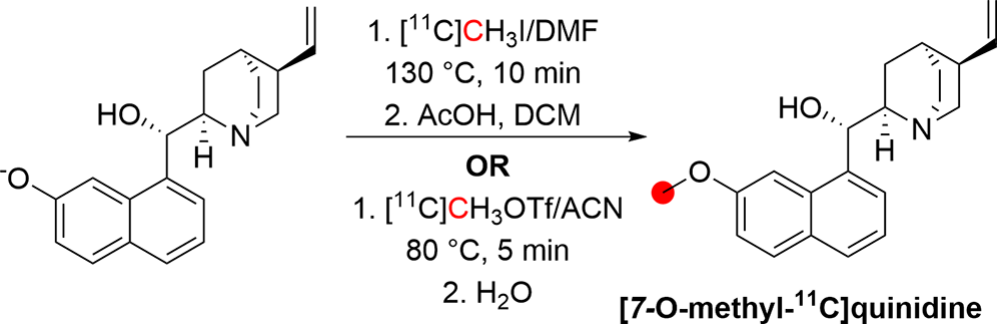
Synthesis of [*7*-*O*-*methyl*-^11^C]quinidine using [^11^C]CH_3_I or [^11^C]CH_3_OTf. ^11^C radionuclide position is highlighted in red.

The radiosynthesis of [*7*-*O*-*methyl*-^11^C]quinidine was accomplished using the potassium salt of *O*-desmethylquinidine as a precursor and by reacting it with [^11^C]CH_3_I in dry DMF at 130 °C for 10 min (Figure 43). The reaction is then quenched with acetic acid in dichloromethane and injected into the HPLC for purification. With a total processing time of 55 min from EOB, [*7*-*O*-*methyl*-^11^C]quinidine is obtained with RCY of 50–60% and *A*_m_ of 1.48–2.22 GBq/μmol calculated at EOS.^147^ A similar radiolabeling strategy was subsequently adopted by Syvänen *et al*.,^146^ with the sodium salt of *O*-desmethyl quinidine used as a precursor and [^11^C]CH_3_OTf used as a labeling agent (Figure 43). The reaction, proceeding for 5 min at 80 °C, was then quenched in water and purified *via* HPLC to return [*7*-*O*-*methyl*-^11^C]quinidine with an RCY of 55–65% and *A*_m_ of 259 ± 49 GBq/μmol calculated at EOS with an overall synthesis time of 45 min from EOB.^146^

#### 3.17.2 Preclinical Studies

Preclinical studies with [*7*-*O*-*methyl*-^11^C]quinidine, a known P-gp substrate, were performed in rodent models of epilepsy using Sprague-Dawley rats (16 male and 70 female) to assess the interaction with multidrug resistance proteins at the BBB in pathological states.^146^ Epileptic seizures were induced by implanting electrodes in the right anterior basolateral nucleus of the amygdala or by phenobarbital administration.^146^ PET studies were performed by injection of [*7*-*O*-*methyl*-]quinidine and initial assessment of the biodistribution without pharmacological alterations. Then rats were infused with Tariquidar, a P-gp inhibitor, for 10 min, and after 20 min, the second injection of [*7*-*O*-*methyl*-^11^C]quinidine and PET scanning was performed to assess any modification in the activity retained by the brain. Without pharmacological treatment, the activity in the brain was low, whereas Tariquidar infusion induced a significant increase in activity retained by the brain (+68%), confirming that [*7*-*O*-*methyl*-^11^C]quinidine is indeed a substrate for P-gp proteins at the BBB.^146^

### 3.18 Scopolamine

#### 3.18.1 Radiosynthesis

**Figure 44:**
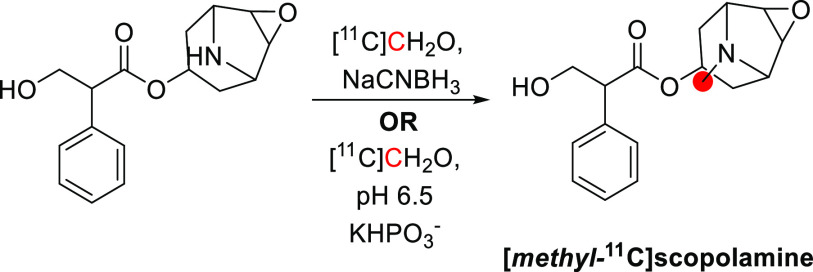
Synthesis of [*methyl*-^11^C]scopolamine from norscopolamine and [^11^C]CH_2_O and NaCNBH_3_^148^ or [^11^C]CH_2_O and KHPO_3_^–^.^152^^11^C radionuclide position is highlighted in red.

[*Methyl*-^11^C]scopolamine was synthesized within 40 min from EOB by reductive methylation of norscopolamine with [^11^C]CH_2_O and sodium cyanoborohydride and then purified using preparative HPLC (Figure 44). The RCY ranged from 30–40%, with a *A*_m_ of 0.037–0.148 GBq/μmol at the EOS.^148^

A synthesis of [*methyl*-^11^C]scopolamine in high *A*_m_ and capable of use in clinical practice was prepared by Mulholland *et al*. in 1988.^152^ [^11^C]CH_2_O, produced by catalytic oxidation of [^11^C]CH_3_OH over metallic silver, was used to *N*-^11^C-methylate norscopolamine (Figure 44). The labeling reaction was complete after 5 min at 75–80 °C, and the [*methyl*-^11^C]scopolamine was isolated by preparative HPLC with a total synthesis time of 40–45 min. The RCY ranged from 20–43% with a *A*_m_ of 0.037 GBq/μmol and RCP of 99%.^152^

#### 3.18.2 Preclinical Studies

Seven rats were given [*methyl*-^11^C]scopolamine *via* the tail vein.^148^ Approximately 0.5% reached the brain 20 min p.i. Areas of the brain with the highest concentration of muscarine receptors, such as the cortex and basal ganglia, showed the highest radioactivity uptake.^148^ Another study in rats showed the whole-body biodistribution of activity to estimate the radiation dose in humans.^149^

#### 3.18.3 Clinical Studies

The biodistribution of [*methyl*-^11^C]scopolamine in the human brain was investigated in six normal volunteers 20–60 years old.^150,151^ Radioactivity in the testes and brain likely reflects primary sites of metabolism and excretion. Scopolamine is initially delivered to the brain in a perfusion-directed pattern, where a significant brain parenchymal tracer uptake was observed. After 30–60 min, activity is lost from the cerebellum, thalamus preferentially, and other cerebral structures with low muscarinic receptor density. However, activity accumulates in receptor-rich areas, including the cerebral cortex and the basal ganglia, throughout a 2 h p.i. The total brain uptake averaged 3.2% at 70–90 min p.i., resulting in an estimated average brain muscarinic receptor concentration of 2.6 nM.^150,151^

### 3.19 Theophylline

#### 3.19.1 Radiosynthesis

**Figure 45:**
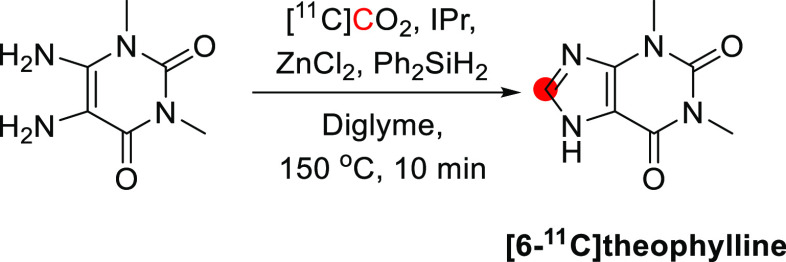
Radiosynthesis of [*6*-^11^C]theophylline using [^11^C]CO_2_. ^11^C radionuclide position is highlighted in red.

[*6*-^11^C]Theophylline was synthesized first by Liger *et al*. in 2019.^153^ This is a one-step reaction, where zinc chloride and *1*,*3*-bis(*2*,*6*-diisopropylphenyl)imidazol-*2*-ylidene (IPr) were added to the vial, followed by a solution of amine in diglyme (Figure 45). Diphenylsilane was added last, and the vial was sealed. [^11^C]CO_2_ was then released within the vial cooled at 0 °C, and the reaction vial was heated at 150 °C for 10 min. [*6*-^11^C]Theophylline was obtained with a RCY of 14–18% from the end of [^11^C]CO_2_ trapping within the vial.^153^ Unfortunately, [*6*-^11^C]theophylline has not been evaluated to the best of our knowledge.

## 4 Amino Acids

Natural l-amino acids (AAs) play a crucial role in virtually all biological processes, from protein synthesis to cell signaling. In addition, AAs can play a crucial role in metabolic cycles, and they are precursors for many other biomolecules (*e.g*., adenine, adrenaline, serotonin).^178^

The AAs incorporated biosynthetically into proteins during translation are known as proteinogenic AAs. Of the basic set of 20 amino acids, 11 can be synthesized from metabolic pathways, whereas the other nine must be obtained from dietary sources (essential amino acids).

Radiolabeled AAs have a broad scope of application as radiopharmaceutical tracers and have been used as receptor ligands, enzymes, and transporter substrates for many diseases. Additionally, PET radiolabeled AAs often results in high-contrast images that are not distorted by tissue inflammation, known for glucose-based PET tracer ^18^F-fluorodeoxyglucose ([^18^F]FDG). Also, in the CNS, [^18^F]FDG has a high glycolytic signal that often presents an advantage to using labeled amino acids as radiotracers for CNS tumor imaging.

Many proteinogenic amino acids have been radiolabeled, but only a few have been evaluated in preclinical and clinical studies. These radiolabeled amino acids differ in ease/route of synthesis, biodistribution, and formation of radiolabeled metabolites *in vivo* (Table 3). In humans, only 12 of them can be synthesized and characterized as nonessential amino acids, with nine of them to be already labeled with carbon-11:

- Alanine can be synthesized from pyruvate^179^ and has a crucial role in the glucose-alanine cycle catalyzed by alanine transaminase.^180^
- Asparagine is biosynthesized from aspartic acid and ammonia by asparagine synthetase and is required for the development and function of the brain.^181^
- Aspartic acid is biosynthesized by the aspartate transferase, which interconverts aspartate to glutamate^179^ and can be transported through the excitatory amino acid transporter 1, expressed in the plasma membrane, cardiac myocytes, astrocytes, and Bergmann glia in the cerebellum.^182^
- Glutamic acid, one of the most abundant amino acids in the body, is metabolized to a-ketoglutarate by enzymes, including glutamate dehydrogenase 1 and 2, aspartate aminotransferase 2, and alanine aminotransferase 1, in the mitochondria. Glutamate is also converted to γ-aminobutyric acid by glutamate decarboxylase 1 and 2, most abundant in the brain and pancreatic β-cells.^183^ In the brain, glutamate is converted to glutamine by glutamine synthetase.^184^ Biochemical receptors for glutamate can be categorized into three major classes: α-amino-3-hydroxy-5-methyl-4-isoxazolepropionic acid (AMPA) receptors, *N*-methyl-d-aspartate (NMDA) receptors, and metabotropic glutamate receptors. There is also a fourth class, much less abundant, known as kainate receptors, similar in many respects to AMPA receptors.^185^
- Glutamine, like glucose, has a primary role in the mammalian body and is an energy source for cellular growth and proliferation. It has a major role in transporting carbon, nitrogen, and energy between tissues. Glutamine plays an essential role in the CNS, where it is converted to the major excitatory amino acid glutamate.^186^ Moreover, cancer cells consume more than normal cells, making glutamine a perfect study target.^187^
- Glycine can be biosynthesized *via* several pathways, with the main synthetic route being by enzymatic conversion of serine to glycine from serine hydroxymethyltransferase. Glycine, and its *N*-methylated derivative, sarcosine, are part of the choline degradation pathway, in which sarcosine is metabolized to glycine by the enzyme sarcosine dehydrogenase.^188^ Glycine is a major inhibitory neurotransmitter that binds to the glycine receptor, a ligand-gated ion channel belonging to the nicotinic acetylcholine receptor family and acts as co-agonist glutamate at *N-*methyl-d-aspartic acid NMDA receptors.^189^ Glycine was first radiolabeled with carbon-11 in 1986 as a precursor to the dipeptides l*-*phenylalanylglycine and l-leueylglycine.^190^ In the following year [^11^C]glycine was explored as a tracer for imaging anaplastic astrocytoma.^191^
- [^11^C]Sarcosine has recently been investigated as a new tracer for PET imaging of the prostate,^192,193^ because highly elevated sarcosine levels have been observed in localized prostate cancer.^194^
- Proline is biosynthetically derived from the amino acid l-glutamate and has been found to act as a weak agonist of the glycine receptor and both NMDA and non-NMDA (AMPA/kainate) ionotropic glutamate receptors.^178^ Therefore, it has been proposed to be a potential endogenous excitotoxin.^189,195,196^ Proline might mainly be suited to study collagen synthesis rates. Aberrant collagen production occurs in a variety of diseased states and tumor growth.
- Serine biosynthesis starts with the oxidation of 3-phosphoglycerate to 3-phosphohydroxypyruvate and nicotinamide adenine dinucleotide (NADH) by phosphoglycerate dehydrogenase. This ketone’s reductive amination by phosphoserine transaminase yields 3-phosphoserine hydrolyzed to serine by phosphoserine phosphatase.^197,198^ Serine is the precursor to several amino acids, including glycine and cysteine, and participates in the biosynthesis of purines and pyrimidines. Serine plays an important role in the catalytic function of many enzymes, such as chymotrypsin and trypsin, occurring in their active sites. d-Serine is the second d-amino acid discovered after d-aspartate naturally existed in humans. It is present in the brain and peripheral organs, such as the kidney, as a signaling molecule.^199,200^ Endogenous d-serine is produced by the epimerization of l-serine in neurons by serine racemase. The resulting d-serine is transported into astrocytes for storage. Na^+^-independent alanine–serine–cysteine transporter-1 (ASCT1) is found exclusively in neurons. Na^+^-dependent ASCT1 and ASCT2 are present in both neurons and astrocytes. It was demonstrated that d-serine plays an important role in the formation and maturation of synaptic contacts and the earlier stages of neuronal circuit construction as a regulator of neuroblast migration in the developing brain.^201^
- Tyrosine is synthesized by hydroxylation of phenylalanine by the enzyme phenylalanine hydroxylase and is mainly incorporated into all proteins and metabolized to essential substances in the body, such as thyroxine, melanin, dopamine, and norepinephrine.^202^ It is transported across the cell membrane primarily *via*l-type amino acid transporter 1 (LAT-1), and it is absorbed from the small intestine and transported into the liver *via* the portal circulation.^202,203^

**Table 3:** Carbon-11 Labeled Amino Acids

compd	radiolabeling position	preclinical and clinical studies	synthon	*A*_M_ (GBq/μmol)	RCY	ee	total time (min)	ref
alanine	*1*-	mice,^240^ humans^240^	[^11^C]HCN	nr	20%	99%	40	(241)
	*3*-	not reported (nr)	[^11^C]CH_3_I	>0.050	20%	90%	50	(242)

asparagine	*4*-	nr	[^11^C]HCN	18.6	53%	nr	45	(243)

aspartic acid	*4*-	rats, rabbits, dogs, pigs, and monkeys,^244−247^ humans^245,248^	[^11^C]CO_2_	0.185	10%	nr	15	(244)
			[^11^C]HCN	nr	70%	97.8%	45	(249)

glutamic acid	*1*-	nr	[^11^C]CO_2_	nr	20%	nr	8	(250)
	*5*-	rats, rabbits, pigs.,^245,248,250^ humans^245,248^	[^11^C]CO_2_	nr	20%	61%	35	(250)
			[^11^C]CH_2_CHCO_2_H	nr	nr	61%	nr	(251)

glutamine	*5*-	mice,^252,253^ rats,^245^ humans^254^	[^11^C]CsCN	7.0	70%	>98%	45	(255)
glycine	*1*-	humans^191^	[^11^C]HCN	56	49%	nr	38	(192)
homocysteine	*1*-	dogs^256,257^	[^11^C]CO_2_	nr	15%	nr	45	(258)

leucine	*1*-	mice and rats,^259−261^ dogs,^262^ monkeys,^263−266^ humans^263,264,267−270^	[^11^C]HCN	nr	nr	nr	50	(271)
	*3*-	nr	[^11^C]alkyl iodide	nr	10%	nr	10	(272)
	*5*-	nr	[^11^C]CH_3_I	nr	38%	99%	38	(260)

lysine	*1*-	rats^273^	[^11^C]CO_2_	nr	15%	nr	50	(274,275)
	*6*-	nr	[^11^C]ICH_2_CH_2_CH_2_CN	nr	nr	nr	nr	(275)

methionine	*methyl*-	mice, rats, dogs, and pigs,^214,276−278^ monkeys,^279^ humans^280,281^	[^11^C]CH_3_I	nr	75	>93.7%	15	(282)

*N*-methylglycine	*N*-*methyl*-	mice, rats, and humans^193^	[^11^C]CH_3_OTf	280	14%	nr	25	(192,193)
	*1*-	mice^192^	[^11^C]NaCN	>56	4%	nr	40	(192)

*S*-methyl-l-cysteine	*methyl*-	mice,^283^ humans^284^	[^11^C]CH_3_I	nr	50%	nr	12	(283)

norleucine	*1*-	rats^214^	[^11^C]HCN	nr	35%	nr	60	(214,285)
	*3*-	nr	[*1*-^11^C]CH_3_CH_2_CH_2_CH_2_I		10%		100	(286)

norvaline	*3*-	nr	[*1*-^11^C]CH_3_CH_2_CH_2_I	nr	25%	80%	20	(272)

ornithine	*1*-	rats^273^	[^11^C]CO_2_	nr	14%	nr	50	(273)
	*5*-	nr	[^11^C]KCN	77.7%	40%	nr	50	(272)

phenylalanine	*1*-	rats^214^	nr	nr	nr	nr	nr	(212,215,287)
	*3*-	nr	[^11^C]C_5_H_5_CH_2_I	135	27%	99%	24	(217)

phenylglycine	*1*-	rats^214^	[^11^C]CO_2_	n.r	6%	nr	nr	(288)
	*2*-	nr	[^11^C]C_5_H_5_COH	0.037	6%		50	(289)

proline	*1*-	rats^273^	[^11^C]CO_2_	nr	18%	nr	45	(273)
serine	*3*-	nr	[^11^C]CH_2_O	1.85	2%	nr	50	(290)
tryptophan	*1*-	monkeys,^291^ humans^292,293^	[^11^C]CO_2_	2.5	25%	nr	55	(220)
*5*-hydroxytryptophan	*3*-	monkeys,^218,294−296^ rat,^297−300^ mice,^301^ humans^302−307^	[^11^C]CH_3_I	44	24%	nr	50	(220)
tyrosine	*1*-	humans^308,309^	[^11^C]HCN	111	15%	98%	45	(287)

valine	*1*-	rats and dogs,^310^ humans^292,311^	[^11^C]KCN	1.30	70%	racemic	45	(312)
	*3*-	nr	[*2-*^11^C](CH_3_)_2_CHI	nr	9%	80%	nr	(286)

From the nine essential amino acids, only five have been labeled with carbon-11:

- Leucine is an essential ketogenic amino acid, metabolized to acetyl-coenzyme A (acetyl-CoA) and acetoacetate.^204^ It has been radiolabeled in positions 1, 3, and 5. Leucine may be important in the study of regional cerebral protein synthesis rate and organ function.^205−208^
- Lysine is used in the biosynthesis of proteins and the crosslinking of collagen polypeptides, epigenetic regulation through histone modification, and fatty acid metabolism *via* the production of carnitine. The α-aminoadipic semialdehyde synthase protein catalyzes the primary metabolic route for lysine degradation *via* the saccharopine pathway within the mitochondria.^209^
- Methionine is involved in several biological functions transported into the cell *via* the reversible sodium-independent transport system LAT-1.^210^
- Phenylalanine has one of the highest brain uptake indexes among amino acids.^211^ Although it has good potential for investigating protein synthesis rates *in vivo* by PET, only limited *in vivo* studies have been performed using this ^11^C-labeled amino acid.^212−215^ One reason might be its peripheral metabolism to tyrosine and intense competition with tyrosine and methionine^216^ for the same transport system localized in the endothelium of the brain capillaries with consequent complications for tracer kinetic modeling. The second reason is that until recently, no automated procedure has been available for reliable production of [^11^C]phenylalanine due to complex multistep syntheses or low RCYs after isolation and purification of l-enantiomer.^217^
- Tryptophan, in addition to its role as a building block in protein synthesis, is a crucial biochemical precursor to serotonin, the hormone melatonin, and the cofactor nicotinamide adenine dinucleotide. Tryptophan is converted to serotonin in a two-step enzymatic process involving hydroxylation to 5-hydroxytryptophan (5-HTP), then decarboxylation by aromatic amino acid decarboxylase. Serotonin is catabolized by the actions of monoamine oxidase and aldehyde dehydrogenase to 5-hydroxyindoleacetic acid, which is released into the bloodstream and excreted by the kidneys. Finally, because serotonin cannot cross the BBB, it is synthesized within neurons following the transportation of tryptophan across the BBB by the large amino acid transporter. As endogenous precursors to serotonin, tryptophan and 5-HTP have been radiolabeled with carbon-11 at either the carboxyl- or β-position and investigated as PET probes to measure serotonin synthesis in health and disease. [^11^C]Tryptophan is also used for protein and kynurenine synthesis, so caution should be used as a marker of tryptophan synthesis alone. 5-HTP, on the other hand, is involved solely in serotonin synthesis through decarboxylation by aromatic d-amino acid decarboxylase (AADC) and can pass the BBB; hence [^11^C]5-HTP is advantageous as a serotonergic PET probe. The choice of labeling position is important because decarboxylation will sever the radiolabel from ^11^C-carboxyl labeled *5*-HTP, resulting in no specific signal.^218^ When labeled in the β-position, ^11^C will be retained within the resultant [^11^C]serotonin molecule. However, due to specialized enzymes for this radiochemistry, PET studies using [β-^11^C]HTP have been limited to a few research institutions.^219,220^
- Valine is used in the biosynthesis of proteins and has stimulant activity, promoting muscle growth and tissue repair.^178^ The amino acid transports across the cell plasma membrane through the monocarboxylate transporter 10. The latter is widely expressed across multiple tissues but very low in pancreatic α- and β-cell membranes. However, valine has the highest affinity for the pancreas compared to other natural AAs.^221^

Nonproteinogenic amino acids occur in nature and are also crucial as intermediates in biosynthesis or found in proteins by post-translational modification:^222^

- Homocysteine has two primary metabolic pathways: (a) through the trans-sulfuration pathway in vitamin B6-dependent reactions^223^ and (b) can cyclize to give homocysteine thiolactone catalyzed by methionyl-tRNA synthetase and vice versa.^224^ Intracellular homocysteine can be converted into *S*-adenosyl-l-homocysteine by cytosolic hydrolase, which is found in sufficient amounts in all organs, including the heart.^225^
- *S*-Methyl-l-cysteine is a natural amino acid produced by post-transcriptional methylation, mainly in plants.^226^ Several studies reported the beneficial effects of low doses of S-methyl-l-cysteine on the cardiovascular system due to hypocholesterolemic, antioxidant, antidiabetic, and hepatoprotective functions.^205−208^ The use of *S*-[^11^C]methyl-l-cysteine was proposed as a tumor imaging tool to overcome the low specificity such as [^18^F]FDG currently presents.^209^
- Norleucine, biosynthetically, arises *via* the action of 2-isopropylmalate synthase on α-ketobutyrate.^227^ It penetrates the brain cells and cell particles very slowly compared with other amino acids, and its low recovery rate indicates relatively effective excretion.^228^ Although it does not contain sulfur, it is nearly isosteric with methionine.^229^ Thus, norleucine has been used to probe the role of methionine in amyloid-β peptides in Alzheimer’s disease (AD).^230^
- Norvaline is a natural component of an antifungal peptide of *Bacillus subtilis*, a Gram-positive, catalase-positive bacterium found in the soil and the gastrointestinal tract of ruminants and humans.^231^ Norvaline promotes tissue regeneration and muscle growth and is commonly used by bodybuilders.^226,232^ Norvaline, as a non-competitive arginase inhibitor, readily crosses the BBB, and reduces arginine loss in the brain associated with the amyloid-β deposition.^233^
- Ornithine is produced in the urea cycle through the cleavage of urea from arginine and has a central role in the cycle, allowing the disposal of excess nitrogen. In addition, it is a precursor of citrulline and arginine found in mitochondria and cytoplasm.^234,235^
- Phenylglycine occurs in natural products, including almost all glycopeptide antibiotics and biologically active linear and cyclic peptides.^236,237^ Phenylglycine biosynthesis gene was not identified until 2011. It currently seems to be synthesized from phenylpyruvate, which is converted into phenylacetyl-CoA by a pyruvate dehydrogenase-like complex of protein glycosyltransferase (Pgl) B and C enzymes.^238^

In 2018 Pekosak *et al*. published a comprehensive review of developed synthesis methods for [^11^C]amino acids, including *in vivo* studies for some of them.^239^ Since then, no other synthetic approaches toward carbon-11 amino acids production have been reported. Thus, this review provides for each radiolabeled amino acid, the synthetic methods, and preclinical and clinical studies on healthy subjects illustrating the progress up to date.

### 4.1 Alanine

#### 4.1.1 Radiosynthesis

Alanine has been labeled with ^11^C in the 1- and 3- positions. A route to *l*-[*3*-^11^C]alanine was reported for the first time in 1979 when Langstrom *et al*. utilized an asymmetric synthesis procedure.^313^ Subsequently, several strategies have been developed to synthesize *l*-[*3*-^11^C]alanine.^239^ The most recent synthetic strategy was published in 2016 when Filp *et al*. utilized a phase-transfer catalysis enantioselective alkylation of a commercially available Schiff base glycine precursor with [^11^C]CH_3_I. *l*-[*3*-^11^C]Alanine was synthesized with RCY of 20% and RCP >95% within 50 min from the EOB. The *A*_m_ was >0.050 GBq/μmol at the EOS, and the highest enantiomeric excess (ee) achieved was >90%.^242^

The synthesis of *d*,*l*-[*l*-^11^C]alanine was first published by Machulla *et al.*, utilizing [^11^C]CO_2_ as a precursor of [*1*-^11^C]propanoic acid, followed by the synthesis of [*1*-^11^C]*α*-bromopropanoic acid using PBr_3_/Br_2_ and subsequent synthesis of *d*,*l*-[*l*-^11^C]alanine using NH_3_, in a RCY of 10% within 70 min.^314^ The most recent synthetic pathway for *d*,*l*-[*l*-^11^C]alanine was published by Takahashi *et al*. in 1990, where incorporation of [^11^C]HCN into [*1*-^11^C]*2*-aminopropanenitrile, followed by hydrolysis, leads to the final racemic compound with RCY of 75% and RCP >98% within 40 min from EOB. During this preparation, the final solution was sterile and pyrogen-free.^315^

**Figure 46:**
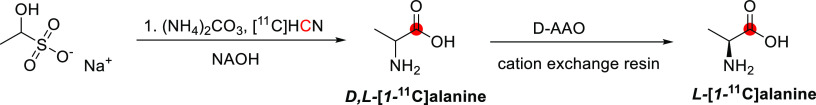
Synthesis of *d*,*l*-[*1*-^11^C]alanine using [^11^C]HCN. ^11^C radionuclide position is highlighted in red.

The preparation of enantiomerical pure *l*-[*l*-^11^C]alanine has been only reported by Ropchan *et al*. from the *d*,*l*-[*l*-^11^C]alanine by a modification of the protocol developed by Bjurling *et al*. after passing the racemate through a light-protected *d*-amino acid oxidase and *l*-alanine dehydrogenase column enzyme. The *l* enantiomer was isolated with RCY of 20% and RCP >98% within 40 min (Figure 46).^241^

#### 4.1.2 Preclinical Studies

Preclinical studies performed by Harper *et al*. in healthy mice after injecting *d*,*l*-[*l*-^11^C]alanine revealed accumulation in the pancreas and heart. However, no other details are available (results presented in the 27^th^ Annual Meeting in Detroit).^240^

#### 4.1.3 Clinical Studies

Injecting *d*,*l*-[*l*-^11^C]alanine to healthy humans showed virtually no localization in the heart or pancreas, and the radioactivity was removed as [^11^C]CO_2_ by expiration due to decarboxylation. However, this study showed that *d*,*l*-[*1*-^11^C]alanine has different distribution by comparing preclinical and clinical studies, and no other studies have been performed.^240^

### 4.2 Asparagine

#### 4.2.1 Radiosynthesis

Labeling asparagine with carbon-11 has only been performed at the urea position. The method was published as a conference abstract by Antoni *et al*. in 1995. The group developed an approach based on the enzymatic synthesis of β-cyanoalanine using cyanide as the labeled precursor. However, the report describes no purification procedure; thus, no details about RCY, RCP, or ee were mentioned. A year later, a patent from the same group was released without further follow-up (EP0733374A3·1999-06-02).

In 2001, Gillings *et al*. published the first complete synthetic labeling procedure for the preparation of *l*-[*4*-^11^C]asparagine, where a novel and rapid methodology for the synthesis of ^11^C radiolabeled AAs, known as aziridine ring-opening reactions, was described.^316^ Aziridines, as alternative electrophiles, can readily react with [^11^C]cyanide to develop different ^11^C-carbonyl AAs. *l*-[*4*-^11^C]asparagine has been reported in a RCY of 30-40% and RCP of 95% within 30 min. Stereospecific synthesis using a highly enantiomerically enriched aziridine-2-carboxylate precursor was not achieved, and only racemic amino acid products were obtained with ee 50%. The *A*_m_ of the final product was estimated to be 0.010–0.050 GBq/μmol at the EOS, based on the *A*_m_ of [^11^C]HCN.^316^

**Figure 47:**
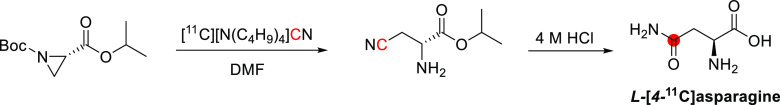
Synthesis of *l*-[*4*-^11^C]asparagine using [^11^C][N(C_4_H_9_)_4_]CN. ^11^C radionuclide position is highlighted in red.

In 2018, Xu *et al*. developed the latest synthetic pathway to prepare enantiomerically pure l-[4-^11^C]asparagine, where a chiral five-membered cyclic sulfamidate was used as the radiolabeling precursor.^243^ Utilizing a [^11^C]CN^–^ nucleophilic ring-opening reaction followed by selective acidic hydrolysis and deprotection, enantiomerically pure *l*-[*4*-^11^C]asparagine, with a total synthesis time of 45 ± 3 min, was isolated with RCY of 53 ± 2% and RCP of 96 ± 2%. The *A*_m_ of the l-[*4*-^11^C]asparagine was 18.6 ± 6.2 GBq/μmol at EOS (Figure 47). This semi-automated radiolabeling process should be adaptable to a commercially available radiosynthesizer and adjustable to a full-scale automation process, which is beneficial for synthesizing *l*-[*4*-^11^C]asparagine on a large scale. Additionally, the development of reaction conditions that retained the stereochemistry during the base-sensitive nucleophilic ^11^C-cyanation using the chiral cyclic sulfamidate precursor will benefit future design and synthesis of radioactive l-alanine moiety containing amino acids and analogues.^243^

### 4.3 Aspartic Acid

#### 4.3.1 Radiosynthesis

As with asparagine, the labeling of aspartic acid has only been performed in one position (*4*-). The first method was published by Barrio *et al*. and constituted the first example of using enzymatic synthesis for ^11^C-labeled amino acid radiotracer synthesis. Briefly, phosphoenolpyruvate carboxylase and aspartate transaminase were immobilized on sepharose activated with cyanogen bromide groups and applied to synthesize *l*-[*4*-^11^C]aspartic acid using [^11^C]CO_2_ as the ^11^C source. *l*-[*4*-^11^C]Aspartate production was completed within 15–25 min after EOB. The actual RCY was about 10%, with RCP of 99% and *A*_m_ of 0.129–0.185 GBq/μmol at EOS.^244^

**Figure 48:**
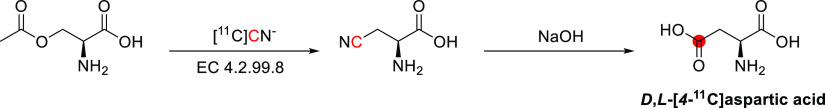
Enzymatic synthesis of *d*,*l*-[*4*-^11^C]aspartic acid using [^11^C]CN^–^. ^11^C radionuclide position is highlighted in red.

In 2001, Antoni *et al*. published the latest enzymatic procedure for the preparation of *l*-[*4*-^11^C]aspartic acid.^249^ The latter was obtained by enzymatic catalysis from *O*-acetyl-*l*-serine with carrier added [^11^C]HCN, using *O*-acetyl-*l*-serine sulfhydrylase (EC 4.2.99.8), followed by alkaline hydrolysis (Figure 48). Enantiomerically pure *l*-[*4*-^11^C]aspartic acid (ee 97.8%) was prepared with a total synthesis time of 45 min and isolated with an RCY of 60–70% and an RCP >95% from EOB.^249^ The immobilization procedures resulted in a sterile and pyrogen-free product suitable for animal and human studies.^244,248^

#### 4.3.2 Preclinical Studies

**Figure 49:**
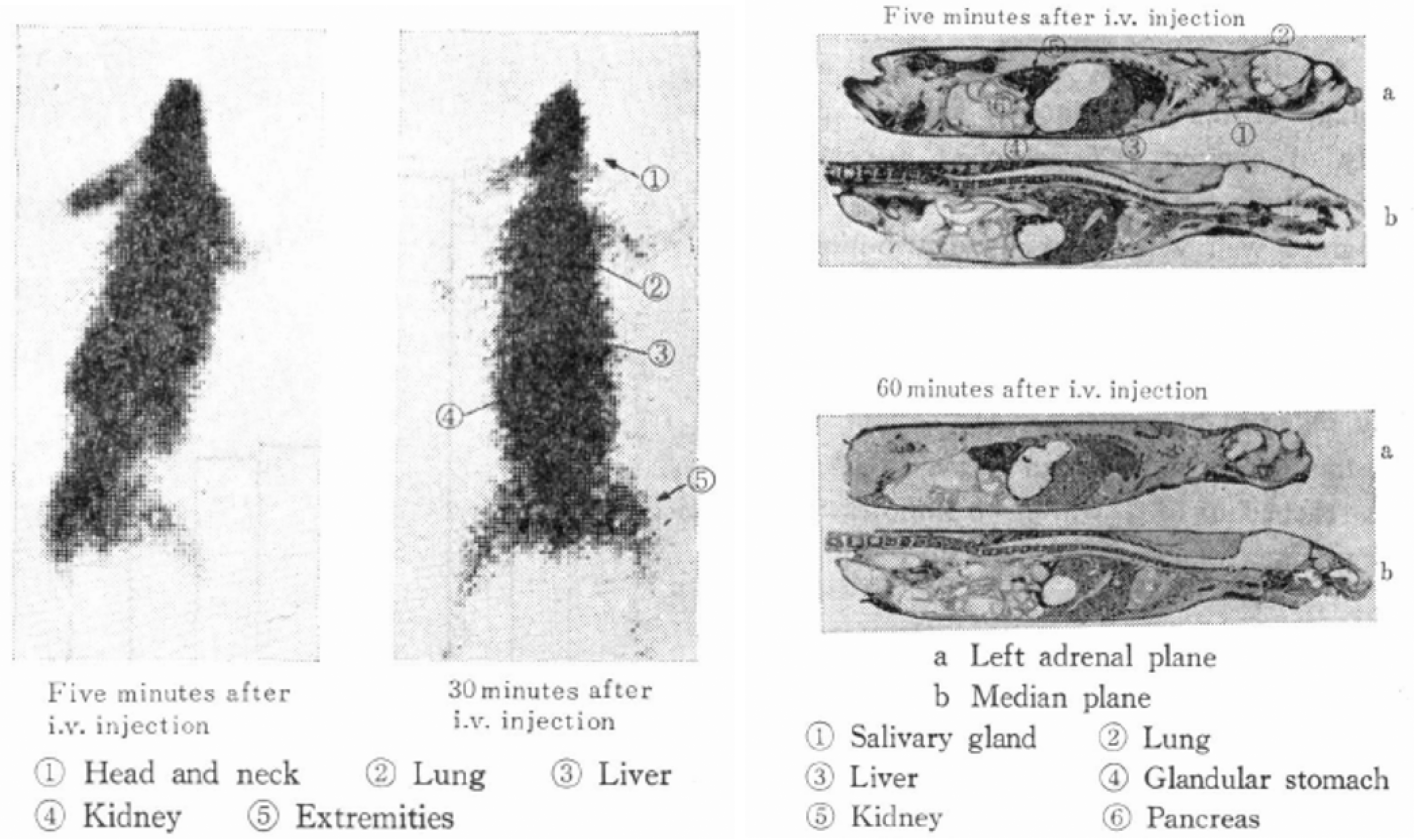
*l*-[*4*-^11^C]Aspartic acid whole-body imaging (left) and autoradiography (right) in rats. Reproduced with permission from ref (246). Copyright 1984 J-Stage. This work is licensed under a Creative Commons Attribution 4.0 International License (https://creativecommons.org/licenses/by/4.0/).

*l*-[*4*-^11^C]Aspartic acid has been evaluated in rats, rabbits, dogs, pigs, and monkeys.^245,246^ Two different studies have been performed on rats. In the first one, male Dawley rats were administered with the radiotracer via the tail vein and sacrificed at 20 min p.i. Co-injection with non-radioactive aspartate (140 mg/kg) led to a 50% increase in kidney radioactivity, while the other organs were slightly decreased (Figure 49).^245^

In a second study, Wister rats received iv *l*-[*4*-^11^C]aspartic acid. After 10 min, 90% of the radioactivity was cleared from the blood, while after 60 min, 60% had been exhaled as [^11^C]CO_2_. Whole-body distribution showed high accumulation in the salivary gland, glandular stomach, and pancreas after 30 min. In addition, high accumulation was also noticed in the head and neck, liver, lungs, and kidney at 10 min, with a tendency to gradually wash out from tissue after 60 min (Figure 49).

l-[4-^11^C]Aspartic acid has been used to study pig myocardial uptake and clearance. Preliminary results showed a low extraction of the order of 8–12%. According to the authors, the myocardium’s clearance rate was rapid, similar to [*1*-^11^C]acetate, measured in the same animal.^247^ This suggests that aspartate, once extracted, rapidly enters the tricarboxylic acid (TCA) intermediate pool.^249^

Other studies performed in dogs and monkeys also showed rapid blood clearance and high retention in the myocardium. Briefly, *l*-[*4*-^11^C]aspartic acid (0.74–1.11 MBq/0.2 mL) was injected into the left anterior descending coronary artery of open-chest instrumented healthy and aminooxyacetic acid-treated dogs. The myocardial activity was recorded for 20 min, where a rapid clearance of the activity from the vascular and extravascular space was observed, similar to the total body distribution in rats.^244^

PET imaging 5 min p.i. of 185 MBq obtained with a positron emission computed axial tomography showed that the rhesus monkey myocardium had a higher accumulation than dogs’ hearts. The PET images in dogs showed a heart/lung activity ratio of 1.2:l, while in monkeys of 32.8:l, the authors suggested that the *l*-[*4*-^11^C]aspartic acid could be essential for assessing local myocardial metabolism.^244^

#### 4.3.3 Clinical Studies

*l*-[*4*-^11^C]Aspartic acid has not been evaluated in healthy humans, but it has been used in two clinical studies with four patients with endocrine pancreatic tumors and three patients (two with carcinoid and one with the endocrine pancreatic tumor).^245,248^ In the first study, the accumulation in the pancreas was rapid and high, except in one patient with secondary pancreatic atrophy. An early peak and a rapid decrease in the kidney were seen. The tracer accumulation in muscle and bone marrow was low.^249^ In the second study, after an injection of 790–980 MBq of the tracer, a region over the liver–pancreas was examined with a dynamic imaging sequence consisting of 14 frames acquired during 45 min. The plasma radioactivity concentration was low at approximately 1.5 from 10 min p.i. The highest uptake was seen in the pancreas, followed by the spleen.^245^ The uptake was low in all the primary tumors and most hepatic metastases. These results discourage imaging neuroendocrine tumors and their metastases because they often reside in those organs.

### 4.4 Glutamic Acid

#### 4.4.1 Radiosynthesis

**Figure 50:**
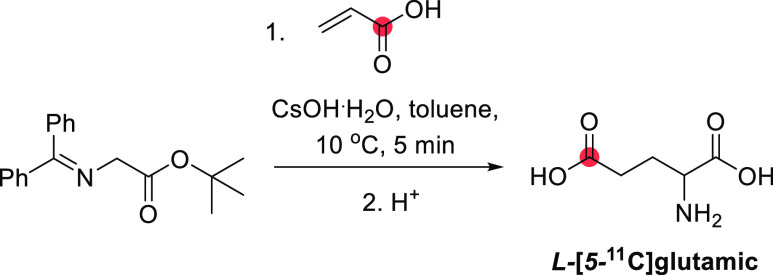
Synthesis of *l*-[*5*-^11^C]glutamic acid using [^11^C]acrylic acid. ^11^C radionuclide position is highlighted in red.

Glutamate has been labeled with ^11^C in the *1*- and *5*- positions. *l*-[*1*-^11^C]glutamic acid and *l*-[*5*-^11^C]glutamic acid were synthesized for the first time by Cohen *et al.*, in 1982, as an extension of their two previously reported biosynthetic methods for the synthesis of [^11^C]citric acid.^250^ For the synthesis of [*1*-^11^C]glutamic acid and [*5*-^11^C]glutamic acid, [^11^C]CO_2_ is first converted to [^11^C]oxaloacetate and [^11^C]acetate, respectively. From that point, the label is incorporated into a series of compounds by the same enzymes; phosphoenolpyruvate carboxylase, citrate synthase, aconitase, isocitrate dehydrogenase, and glutamate-pyruvate transaminase. According to the method, labeling in the 1- and 5-position required 16–18 and 30–35 min, respectively. Both tracers were isolated with RCY of 12–20% and high *A*_m_. According to the authors’ report, this method may be suitable for human use after modification in the final preparation. Specifically, the enzyme should be immobilized on solid- support to guarantee the absence of enzymes in the final solution.^250^ Subsequently, new synthetic methods were only published for *l*-[*5*-^11^C]glutamic acid. The latest method was developed by Filp *et al*. in 2017, utilizing [^11^C]acrylic acid in a novel Michael addition reaction, where a Schiff base glycine derivative was used as a Michael donor. *l*-[*5*-^11^C]glutamic acid was prepared in ee 61 ± 4% with a low RCP of 10–15%, but this preparation was only a proof-of-concept study (Figure 50).^251^

#### 4.4.2 Preclinical Studies

Only glutamic acid labeled in the *5*- position has been preclinically evaluated. *l*-[*5*-^11^C]glutamic acid has been studied in rats, rabbits, and pigs.^245,248,250^ Biodistribution studies in Dawley rats were performed 20 min after administering the tracer *via* the tail vein. The highest uptake was found in the pancreas, kidney, and lungs. When the organ uptake was normalized to radioactivity in blood, maximum levels were seen in the pancreas, kidney, liver, and lungs. Co-injection with non-radioactive glutamate (70 mg/kg) did not alter the uptake in the organs.^245^

Tissue distribution studies in white rabbits of different ages, weights, and species were performed after *l*-[*1*-^11^C]glutamic or *l*-[*5*-^11^C]glutamic acid injection into the ear vein. The animals were sacrificed 5 min p.i., and selected tissues were obtained. The pancreatic uptake ratio was higher in *l*-[*5*-^11^C]glutamic acid. The pancreas, heart, blood, lungs, and kidney to liver ratios for the two ^11^C compounds were significantly different. The blood radioactivity was higher for *l*-[*1*-^11^C]glutamic acid. The different distributions suggest they rapidly metabolize *via* transamination and the TCA cycle. The *l*-[*1*-^11^C]glutamic acid is rapidly decarboxylated compared to *l*-[*5*-^11^C]glutamic acid. Rabbits and humans appear to have a similar pathway for glutamic acid metabolism in the pancreas. However, the myocardial localization in rabbits cannot be reliably extrapolated to humans because of the differences in the myocardial metabolism of amino acids between species.^250^

*l*-[*5*-^11^C]Glutamic acid has been used in a pilot study of pig myocardium, measuring the myocardial uptake and clearance of the tracer. Preliminary results suggest a low extraction of about 8–12%, which was decreased after a continuous infusion of non-labeled glutamate. According to the authors, the pigs’ myocardial clearance was rapid, similar to [*1*-^11^C]acetate.^247^ Thus, they suggested that *l*-[*5*-^11^C]glutamic acid rapidly enters the TCA intermediate pool, probably as [*5*-^11^C]*2*-oxoglutaric acid.^248,317^ However, the low uptake in the heart discourages *l*-[*5*-^11^C]glutamic acid for imaging the healthy and diseased heart.

#### 4.4.3 Clinical Studies

Only the glutamic acid labeled in the 5- position has been evaluated in two clinical studies: one patient with an endocrine pancreatic tumor, two with a carcinoid tumor and liver metastases, and one with an endocrine tumor pancreatic tumor with liver metastases.^245,248^  In the first study, one patient had high radiotracer accumulation in normal pancreatic parenchyma. The healthy abdominal organs, liver and spleen, showed high uptake, while no accumulation was observed in the stomach. However, accumulation was found in segments of the intestine.^317^  In the second study, after an injection tracer, a region over the liver–pancreas was examined with a dynamic imaging sequence of 14 frames for 45 min. The highest uptake was found in the pancreas at 5–10 min p.i., which decreased at the end of the study (40 min p.i.). The kidney had a very high uptake within the first 5 min, which rapidly decreased at 15 min p.i. A study with a co-injection of non-radioactive glutamic acid showed similar results. All cases showed low uptake in the primary tumors and hepatic metastases.^245^ These results discourage its use in imaging neuroendocrine tumors and their metastases. 

### 4.5 Glutamine

#### 4.5.1 Radiosynthesis

**Figure 51:**
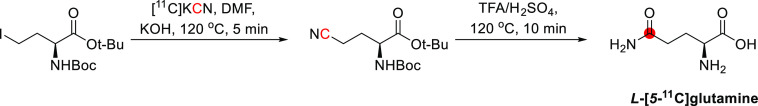
Synthesis of *l*-[*5*-^11^C]glutamine using [^11^C]KCN. ^11^C radionuclide position is highlighted in red.

The first use of *l*-[*5*-^11^C]glutamine was reported by Wu *et al.*, but there is no information about their radiosynthesis method or the purity of the compound.^245^ In 2012 Qu *et al*. published the first detailed radiosynthesis method of *l*-[*5*-^11^C]glutamine.^252^ For the synthesis of *l*-[*5*-^11^C]glutamine, [^11^C]CO_2_ is first converted to [^11^C]CH_4_ and mixed with ammonia gas to trap [^11^C]HCN in KOH solution of DMF to have [^11^C]KCN. The DMF solution of (*S*)-*tert*-butyl *2*-((*tert*-butoxycarbonyl)amino)-*4*-iodobutanoate was introduced into the mixture and heated for 5 min at 120 °C to obtain *5*-^11^C-(*S*)-*tert*-butyl *2*-(*tert*-butoxycarbonyl)amino)-*4*-cyanobutanoate. Hydrolysis was performed by an acidic workup and after purification, *l*-[*5*-^11^C]glutamine was obtained in approximately 60 min in a water solution with a *A*_m_ of 1.85 ± 0.74 GBq/μmol and RCP >94% (Figure 51).^239,318,319^ Results of this study were also published in other papers,^320,321^ and some adaptations were made.^320,321^

**Figure 52:**
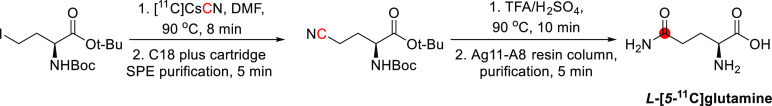
Fully automated synthesis of *l*-[*5*-^11^C]glutamine using [^11^C]CsCN. ^11^C radionuclide position is highlighted in red.

In 2015, Gleede *et al.* developed an improved radiosynthesis of *l*-[*5*-^11^C]glutamine.^255^ This new method was prepared to better understand SN_2_^11^C-cyanation reactions. In this study, different reaction conditions were reported. Using CsHCO_3_/18-C-6 as a trapping solution of [^11^C]HCN instead of KOH has significantly increased the yield of [^11^C]CN^–^ salt (Figure 52). Total synthesis time was decreased to 37–52 from 60 min with ∼80% ^11^C-cyanation yield, ∼60% overall RCY, *A*_m_ of 7.0 ± 1.5 GBq/μmol, and RCP >93%.^255^ In the same study, it was also reported that *d*-[*5*-^11^C]glutamine was observed as an optical impurity as a result of introducing the phase transfer catalyst Krypotfix 222 to the reaction.

In 2017, Filp *et al*. synthesized *l*-[*5*-^11^C]glutamine by a novel Michael addition reaction using [^11^C]methyl acrylate and a Schiff base glycine derivative.^251^ In 2019, *l*-[*5*-^11^C]glutamine was synthesized by using Synthra HCN plus synthesis module and [^11^C]HCN in 60 min (yield 43-52%, RCP >90%).^322^ Similar work was performed with *d*-[*5*-^11^C]glutamine and *l*-[*5*-^11^C]glutamine with RCY of 33.5 ± 16.0 and 34.9 ± 11.3%, respectively.^253^

Rosenberg *et al*. have recently described a reliable two-step automated radiosynthesis/production of *l*-[*5*-^11^C]glutamine under GMP for clinical use.^321^ The product was prepared in RCY of 70% (from trapped [^11^C]HCN) within 45 min from the EOB. The radiosynthesis, optimization, and automation were based upon the previous reports of *l*-[*5*-^11^C]glutamine synthesis.^252,255,323^

#### 4.5.2 Preclinical Studies

Biodistribution studies in Dawley rats were performed 20 min after administering the tracer through the tail vein. The highest uptake has been found in the pancreas, kidney, and liver. Co-injection with nonradioactive glutamine did not alter the uptake in the organs.^245^

Biodistribution studies in mice were performed at 15, 30, and 60 min after administering the tracer through the lateral tail vein.^252^ The highest uptake was found in the pancreas at 15 min. Uptake in the heart and kidneys was instant, but the heart uptake decreased rapidly after 15 min, and the tracer in the kidneys was released into the bladder. Dynamic small-animal PET studies have been performed with F344 rats and transgenic mice with mammary tumors. *l*-[*5*-^11^C]Glutamine was injected in rats bearing xenografted 9L tumors and transgenic mice bearing spontaneous mammary gland tumors.

**Figure 53:**
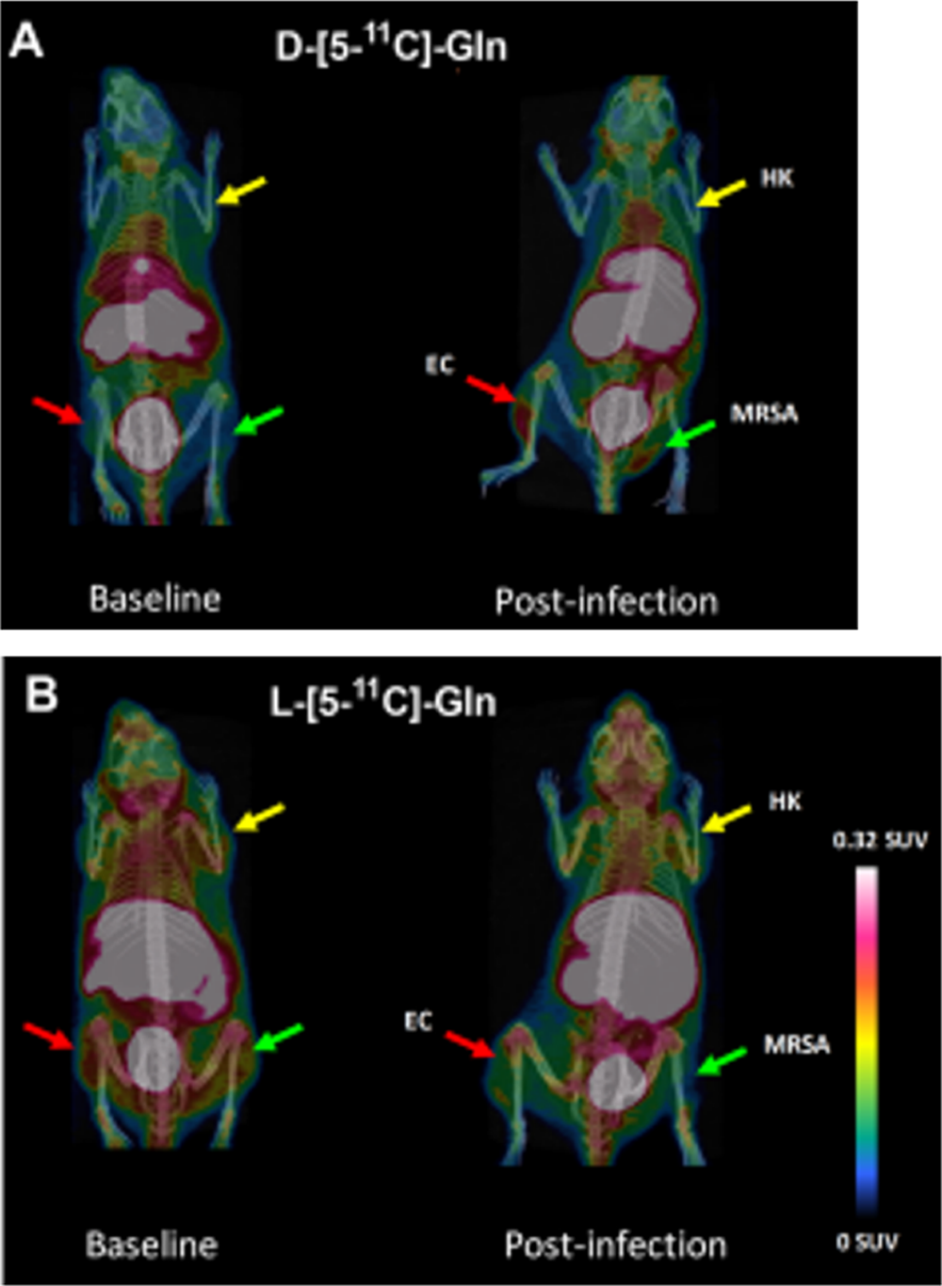
(A) *d*-[*5*-^11^C]Glutamine and (B) *l*-[*5*-^11^C]glutamine PET/CT scans of mice before and after dual myositis infection. EC, *E. coli*; HK, Heat-killed. Reproduced with permission from ref (253). Copyright 2021 American Chemical Society.

In 2021, Renick *et al*. published *in vitro*, *in vivo*, and *ex vivo* studies about *d*-[*5*-^11^C]glutamine and *l*-[*5*-^11^C]glutamine in bacterial, mammalian cell lines, and mouse models.^253^ This comparative study showed high uptakes for *l*-[*5*-^11^C]glutamine in all organs except kidneys (Figure 53).

#### 4.5.3 Clinical Studies

**Figure 54:**
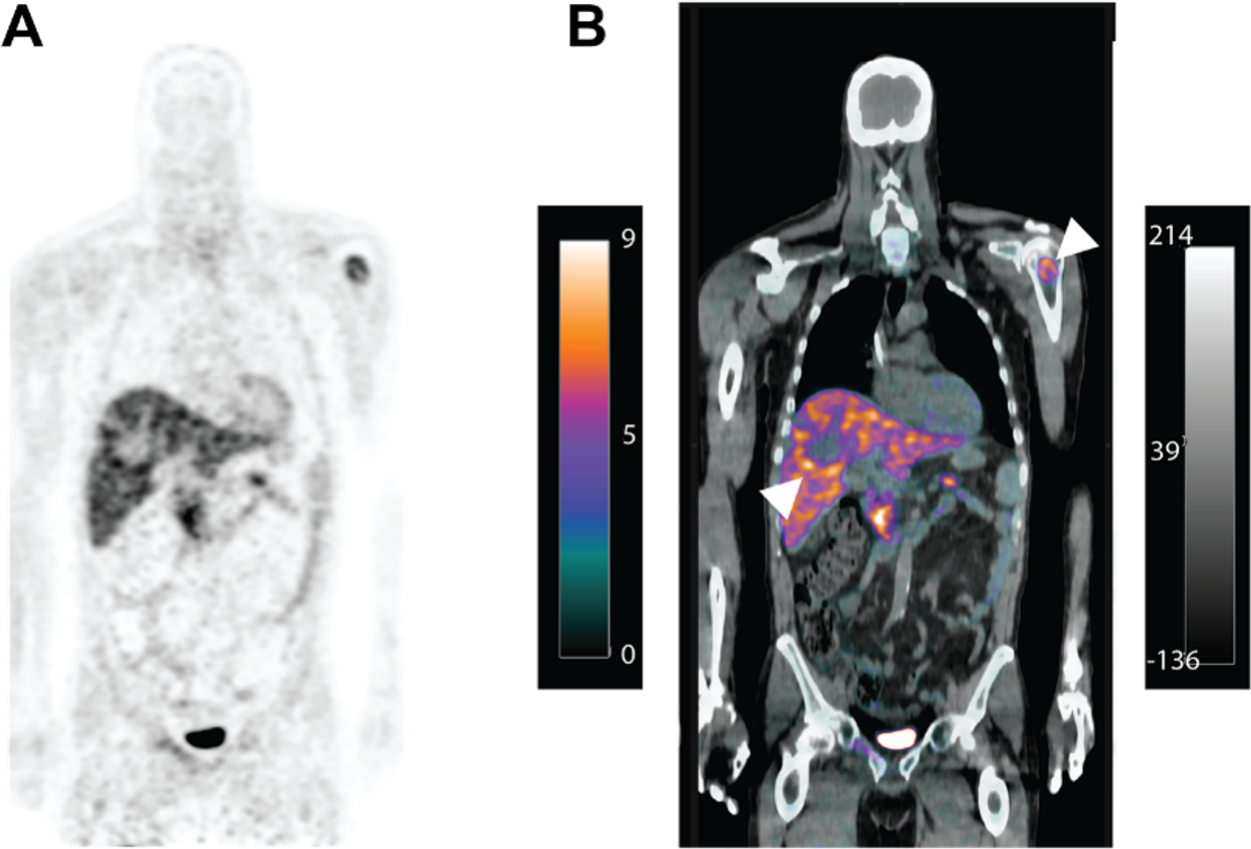
*l*-[*5*-^11^C]Glutamine whole-body PET (A) and PET/CT (B) for biodistribution and tumor imaging in a patient with metastatic colorectal cancer. Reproduced with permission from ref (254). Copyright 2022 Society of Nuclear Medicine and Molecular Imaging. This work is licensed under a Creative Commons Attribution 4.0 International License (https://creativecommons.org/licenses/by/4.0/).

*l*-[*5*-^11^C]Glutamine has been only evaluated in one clinical study with nine patients with metastatic colorectal cancer (Figure 54).^254^ With this first and only in-human study, radiologic safety and biodistribution of *l*-[*5*-^11^C]glutamine were investigated for PET imaging. *l*-[*5*-^11^C]Glutamine was given as an iv injection over 30 seconds. The total scanning time was 58 min for the tumor region and approximately 18 min for a whole-body PET scan. The highest activity was observed from the bladder, pancreas, and liver.^254^

### 4.6 Glycine and *N*-Methylglycine (Sarcosine)

#### 4.6.1 Radiosynthesis

**Figure 55:**
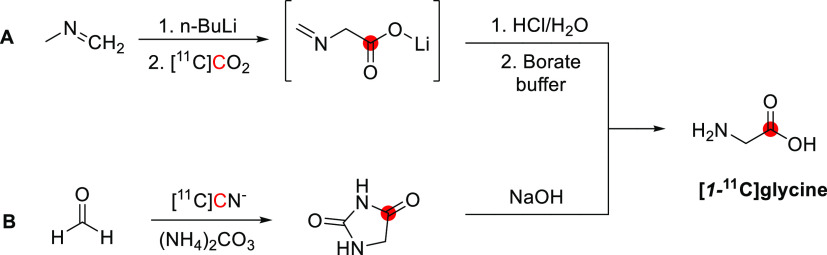
Radiosynthetic routes to [*1*-^11^C]glycine using (A) [^11^C]CO_2_ and (B) [^11^C]CN^–^. ^11^C radionuclide position is highlighted in red.

The radiosynthesis of [*1*-^11^C]glycine was first reported in 1986 by Bolster *et al*. through ^11^C-carboxylation of activated methyl isocyanide (Figure 55A).^190^ In this process, [^11^C]CO_2_ was delivered to a solution of *α*-lithiomethylisocyanide, generated in situ from methyl isocyanide and *n*-BuLi. Subsequent hydrolysis of the isocyanide group using hydrochloric acid gave [^11^C]glycine with an RCY of 10–15% and RCP of 87–94%, in a process lasting 30 min. The following year, an alternative preparation was described by Johnström *et al.*, using [^11^C]cyanide, formaldehyde, and ammonium carbonate in the Bucherer–Bergs synthesis (Figure 55B).^191^ This reaction uses carrier-added KCN and proceeds *via* the formation of the intermediate [^11^C]hydantoin, which is hydrolyzed by a strong base to produce [*1*-^11^C]glycine (synthesis time, 30–35 min; RCY, 35% based on [^11^C]cyanide, RCP > 98%). In 2017, Xing *et al*. reported the non-carrier-added synthesis under similar conditions, producing [*1*-^11^C]glycine in 49% RCY (based on [^11^C]cyanide) in a process lasting 38 min (synthesis time, 38 min; activity, 4 GBq; RCY, 49% based on [^11^C]cyanide; RCP > 95%, *A*_m_ > 56 GBq/μmol).^192^

**Figure 56:**
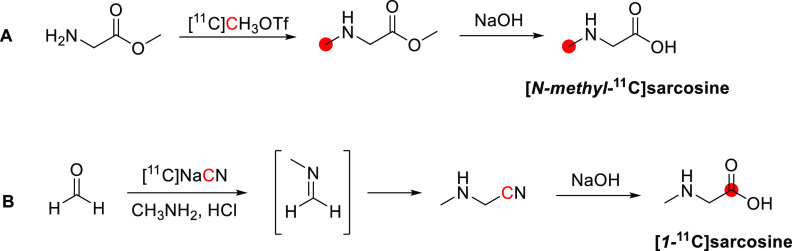
Radiosynthetic routes to [*N*-*methyl*-^11^C]sarcosine using [^11^C]CH_3_OTf (A) and [*1*-^11^C]sarcosine using [^11^C]NaCN (B). ^11^C radionuclide position is highlighted in red.

Sarcosine has been radiolabeled with carbon-11 at the *N*-methyl position,^192,193^ and carbonyl.^192^ [*N*-*methyl*-^11^C]sarcosine was prepared by methylation of methyl glycinate using [^11^C]CH_3_OTf, followed by ester hydrolysis (Figure 56A) within 25 min, with a RCY of 6–14%, RCP >95%, and *A*_m_ >280 GBq/μmol at EOS. [*1*-^11^C]Sarcosine was prepared by the Strecker synthesis using no-carrier-added [^11^C]cyanide (Figure 56B). This reaction proceeds *via* condensation of formaldehyde with methylamine and subsequent reaction with [^11^C]NaCN to generate the [^11^C]*α*-aminonitrile. The nitrile group is then hydrolyzed using a strong base to yield [*1*-^11^C]sarcosine within 40 min, with a RCY of 4% from [^11^C]CO_2_, RCP >90%, and *A*_m_ >56 GBq/μmol.

#### 4.6.2 Preclinical Studies

PET studies in mice bearing human prostate cancer xenografts showed significantly higher tumor-to-background ratios with [*N*-*methyl*-^11^C]sarcosine compared with the established prostate tracer [^11^C]choline. *In vitro* assays in prostate cancer cell lines found [*N*-*methyl*-^11^C]sarcosine uptake could be blocked with excess nonradiolabeled sarcosine, confirming a specific transport mechanism into cells. The palmitoyl acyltransferase (PAT) inhibitor, *5*-hydroxy-*l*-tryptophan competitively inhibited [*N*-*methyl*-^11^C]sarcosine uptake, confirming PAT-mediated transport.^193^

**Figure 57:**
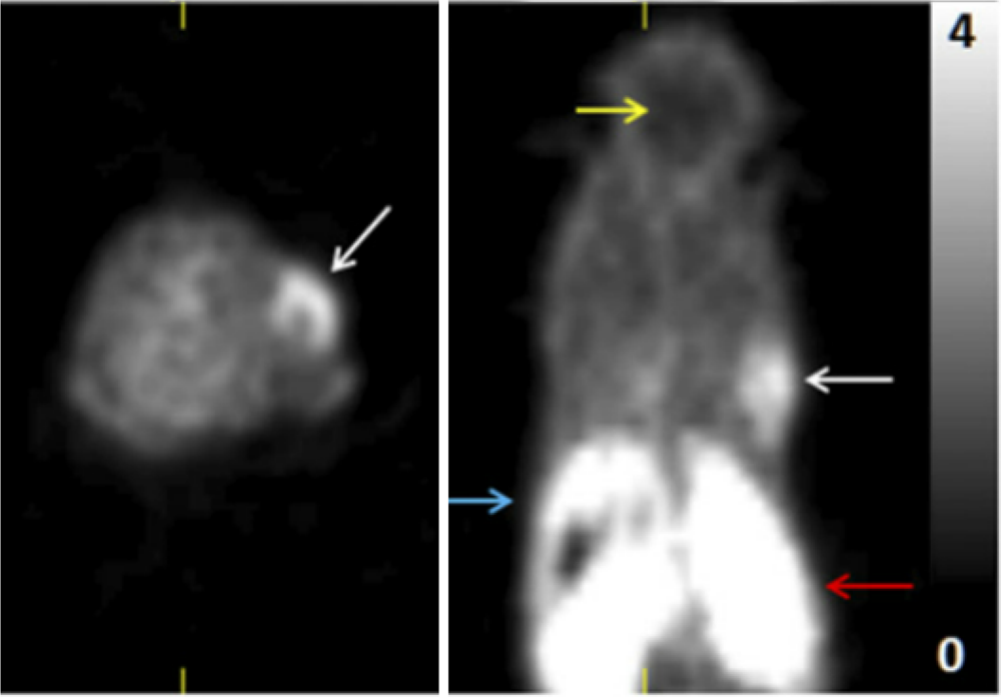
[*N*-*Methyl*-^11^C]sarcosine PET imaging of nu/nu mice highlighting tumor (white arrows) and lower hepatic (blue arrows) uptake. Reproduced with permission from ref (193). Copyright 2017 Society of Nuclear Medicine and Molecular Imaging. This work is licensed under a Creative Commons Attribution 4.0 International License (https://creativecommons.org/licenses/by/4.0/).

A biodistribution study of [*N*-*methyl*-^11^C]sarcosine in normal rats showed high activity uptake in the intestine and kidneys, elevated liver uptake compared with the mediastinum, and negligible brain uptake (Figure 57). No aqueous radio-metabolites of [*N*-*methyl*-^11^C]sarcosine were detected at any time point from blood and tissue homogenates of rat prostate and pancreas; however, radioactivity was observed in the exhaled air, suggesting that the ^11^C-methyl group of sarcosine is eliminated *via* sarcosine dehydrogenase-mediated conversion to glycine and [^11^C]formaldehyde, with the latter being ultimately degraded to [^11^C]CO_2_. Overall, a substantial majority of [*N*-*methyl*-^11^C]sarcosine *vs* [^11^C]CO_2_ was observed in the studied tissues at all time points.^193^

Nevertheless, to prevent the metabolic loss of radiolabel from sarcosine and its exhalation as volatile [^11^C]CO_2_, [*1*-^11^C]sarcosine was investigated.^192^ Preliminary PET imaging in human prostate cancer-bearing mice revealed excellent uptake, better tumor tracer retention, and a slower metabolism.^192^

#### 4.6.3 Clinical Studies

**Figure 58:**
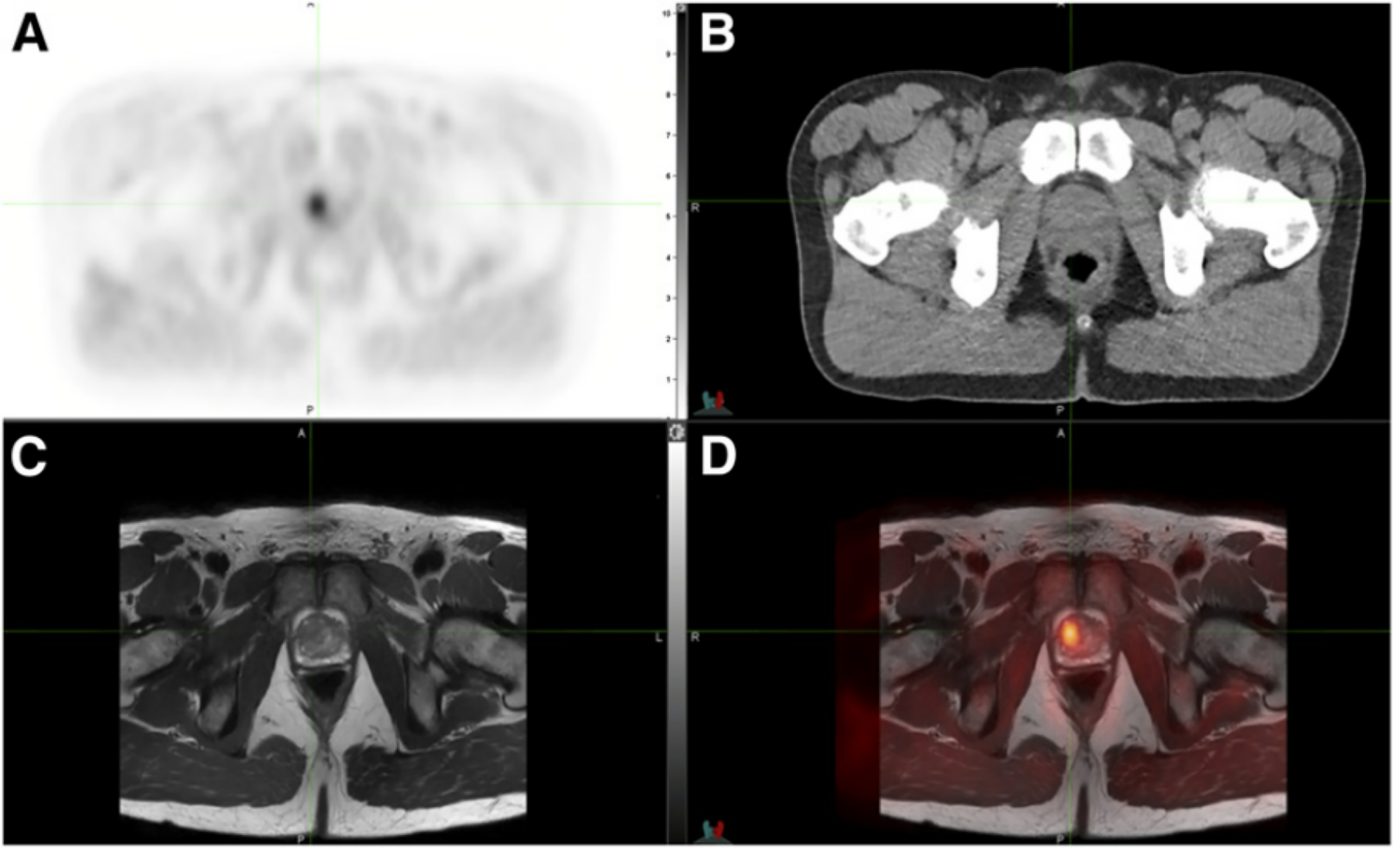
PET (A), CT (B), T2-weighted MR (C), and PET/MRI (D) registration after [*N*-*methyl*-^11^C]sarcosine administration in human. Reproduced with permission from ref (193). Copyright 2017 Society of Nuclear Medicine and Molecular Imaging. This work is licensed under a Creative Commons Attribution 4.0 International License (https://creativecommons.org/licenses/by/4.0/).

In 2017, Piert *et al*. performed the first-in-human PET imaging of sarcosine using [*N*-*methyl*-^11^C]sarcosine in a subject with localized prostate cancer (Figure 58).^193^ High-contrast images were obtained, and time–activity curves demonstrated preferential tracer uptake in the tumor compared with the total prostate and the arterial blood, with a stable lesion-to-background ratio over time. Unfortunately, no human studies with [*1*-^11^C]sarcosine have been reported, which would be interesting for comparing tracer kinetics for the two labeling positions. Nonetheless, [*N*-*methyl*-^11^C]sarcosine as a substrate for the proton-coupled amino acid transporters has emerged as a promising radiotracer in prostate cancer imaging.

**Figure 59:**
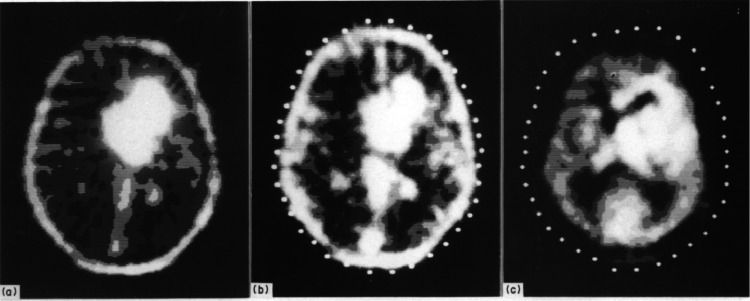
Comparison of [*1*-^11^C]glycine (A), [^68^Ga]EDTA (B), and *l*-[*S*-*methyl*-^11^C]methionine (C) PET scans in visualizing an anaplastic astrocytoma with disrupted BBB. Reproduced with permission from ref (191). Copyright 1987 Elsevier.

The only reported human PET study with [*1*-^11^C]glycine was performed by Johnström *et al*. in 1987, in which brain imaging was performed in five patients with intracerebral tumors, alongside [^68^Ga]EDTA and l-[*S*-*methyl*-^11^C]methionine (Figure 59).^191^ Because glycine lacks a specific transport system over the intact BBB, [*1*-^11^C]glycine accumulation in tumors was confined to areas with a disrupted BBB. The latter contrasts with [*S*-*methyl*-^11^C]methionine, which accumulates in tumors where disruption of the BBB breakdown is not suspected.

### 4.7 Homocysteine

#### 4.7.1 Radiosynthesis

**Figure 60:**
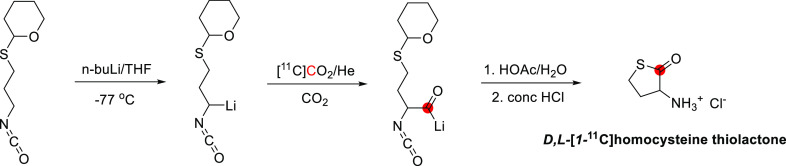
Synthesis of *d*,*l*-[*1*-^11^C]homocysteine thiolactone using [^11^C]CO_2_. ^11^C radionuclide position is highlighted in red.

Labeling of homocysteine has only been performed in the *1*-position. Hamacher *et al*.^258^ have published the only procedure based on the [^11^C]CO_2_ carboxylation method published by Vaalburg *et al*.^288^*d*,*l*-[*1*-^11^C]Homocysteine thiolactone was prepared in four steps (Figure 60). Initially, the carboxylation of α-lithiated 3-*S*-(tetrahydropyranyl)thiopropylisonitrile isocyanide using [^11^C]CO_2_ was followed by complete carboxylation of the residual lithium compound using non-radioactive CO_2_. Finally, by deprotection of the mercapto group and lactonization in an acid-catalyzed reaction, the tracer was obtained after HPLC purification with a RCY of 10–15% and RCP > 98% within 45 min at EOB. The *K* value of the equilibrium constant of *d*,*l*-[*1*-^11^C]homocysteine thiolactone to *d*,*l*-[*1*-^11^C]homocysteine was 0.72.^258^

#### 4.7.2 Preclinical Studies

**Figure 61:**
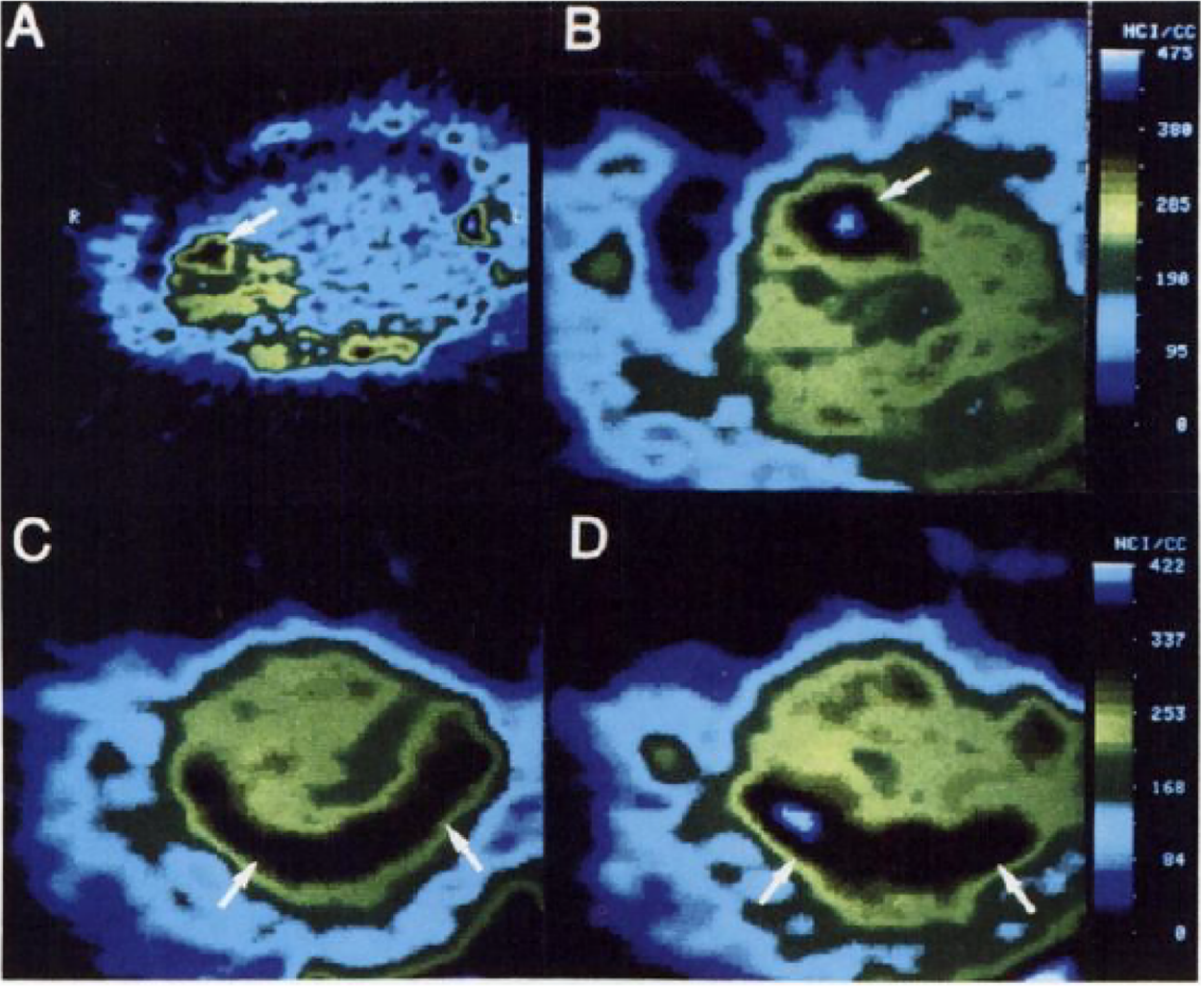
*d*,*l*-[*1*-^11^C]Homocysteine thiolactone accumulation in the thorax of infused dogs. (A) Horizontal plane at the myocardial level with elevated activity in the coronary artery (arrow). (B) Magnification of the cardiac region from (A). (C,D) left ventricular wall during perfusion. Reproduced with permission from ref (257). Copyright 1992 Society of Nuclear Medicine. This work is licensed under a Creative Commons Attribution 4.0 International License (https://creativecommons.org/licenses/by/4.0/).

Animal experiments were carried out in seven anesthetized, thoracotomized dogs after iv infusion of *d*,*l*-[*1*-^11^C]homocysteine thiolactone over 1 min. Regional radioactivity concentration was measured by PET at 60 min p.i. (Figure 61). The highest radioactivity concentration was found in the bladder and the kidney, followed by the liver, spine, heart, and skeletal muscle. The study indicated that the kidneys excreted most of the activity into the urine. The radioactivity accumulated in the spine may be due to the hemopoietic cells of the bone marrow, which may contain high levels of adenosine or SAH-hydrolase, or other metabolic routes for homocysteine conversion. PET imaging obtained 10–60 min following infusion of the tracer showed low radioactivity concentrations in the lung, mediastinum, and thoracic wall, in contrast to the high concentrations of the heart and spine. Accumulation 3 min after the end of tracer infusion is dominant in the ischemic area compared to surrounding tissues, providing a potentially sensitive method to localize regional myocardial ischemia.^256,257^

### 4.8 Leucine

#### 4.8.1 Radiosynthesis

**Figure 62:**
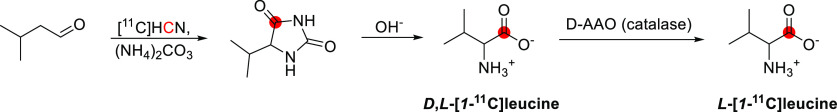
Synthesis of *l*-[*1*-^11^C]leucine using [^11^C]HCN. ^11^C radionuclide position is highlighted in red.

[*1*-^11^C]Leucine was first mentioned as a possible radiotracer in 1978 but was not pursued.^324,271^ Another method to synthesize pure *l*-[*1*-^11^C]leucine *via* a modified Bucherer–Strecker reaction was purified using a column containing *d*-amino acid oxidase.^271^ The total reaction time was 50 min with >99% RCP. In 1983, Barrio *et al*. developed a new synthetic method using isovaleraldehyde (Figure 62). The remote-control synthesis produced the amino acid in a total synthesis time of 30–40 min with >99% RCP and 1480–1850 MBq activity.^262^

Radiosynthesis of *l*-[*1*-^11^C]leucine has also been achieved from carrier added^259^ or no-carrier added [^11^C]NaCN methods.^214,285^ In the no-carrier-added method, [^11^C]HCN was produced from [^11^C]CH_4_ on Pt at 950 °C, bubbled into the reaction medium with aminosulfite, and subsequently heated to 60 °C for 10 min, followed by acid hydrolysis and purification.^214^ In 1985, Washburn and co-workers utilized a purification method using HPLC to separate *d*- and *l*- enantiomers with good resolution.^205^

**Figure 63:**
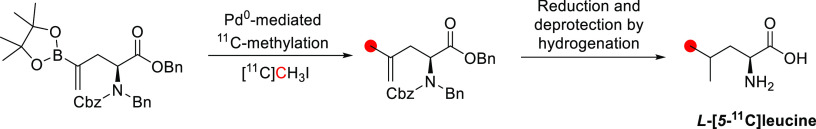
Synthesis of *l*-[*5*-^11^C]leucine using [^11^C]CH_3_I. ^11^C radionuclide position is highlighted in red.

Leucine was also radiolabeled in the *3*-position^272^ using a phase transfer alkylation reaction (in an ultrasonic bath at 45 °C, 10 min) and ^11^C-alkyl iodide (10% RCY and 97% RCP). Labeling in the *5*- position (Figure 63) has been achieved by Pd^0^-mediated [^11^C]CH_3_I methylation and microfluidic hydrogenation (total synthesis time 38 min, radioactivity 2.2–2.7 GBq; RCY 23–38%; RCP and chemical purity (CP) more than 95%, 99% ee).^260^

#### 4.8.2 Preclinical Studies

Carbon-11 labeled *l*-leucine has been used in many preclinical studies. The first preclinical study was mentioned in an annual meeting that included monkeys to measure local cerebral protein synthesis.^263^*l*-[*1*-^11^C]Leucine was introduced by iv injection and showed that it could enter the brain. It was also used on dogs to observe myocardial metabolism,^262^ on monkeys to show a shorter half-life and capability to pass BBB,^262^ and on mice as a pancreas imaging agent.^259^ In 1984, *l*-[*1*-^11^C]leucine was used in a comparative study on Donryu rats to show the distribution of amino acids throughout the body.^214^*l*-[*1*-^11^C]Leucine mainly accumulated in the pancreas at 20 min p.i. Another biodistribution study was done on pregnant Wistar rats (16–19^th^ day of pregnancy) to investigate the distribution of labeled amino acids in maternal organs and their transfer through the placenta.^261^ Radionuclide was injected through the tail vein to the pregnant rats at 30 min p.i. maternal liver showed high uptake, with the highest in fetal lungs and fetal liver. Rhesus monkeys were used to investigate cerebral protein synthesis rate (CPSR).^264−266^ The measurement method produced regional estimations of protein synthesis from an exogenous source of amino acids. In the following years, these studies continued to investigate the sensitivity and stability of the used method.^325^ In 2021, *l*-[*5*-^11^C]leucine was used in A431 tumor-bearing mice to investigate its probe functionality for tumor imaging.^260^

#### 4.8.3 Clinical Studies

The first mention of clinical *l*-[*1*-^11^C]leucine studies was in 1982.^263^*l*-[*1*-^11^C]Leucine administered to a patient with glioblastoma showed a high cerebral protein synthesis rate with the high growth rate of glioblastoma.^263,326^ In another study, nine healthy human volunteers were used to show CPSR in humans.^264^ Used model parameters for the kinetic estimations were applied to hemispheres, gray and white matter.^327^ Other research groups also studied the measurement of regional CPSR using *l*-[*1*-^11^C]leucine and PET.^267,325,328^ According to their study, *l*-[*1*-^11^C]leucine,^265,266,325^ can also be used in young, healthy men with low variance and high reproducibility.^328^*l*-[*1*-^11^C]leucine was administered to 10 healthy men volunteers (21–24 years old, right-handed) *via* intravenous lines on contralateral antecubital fossa over 1 and 2 mins for a total of 90 min dynamic PET scans and regional CPSR showed differences in most of the regions.^328^

In 2006, another study was performed on 27 healthy volunteers (11 men and 16 women between 20–50 years old, fast for 8 h before PET scanning) by introducing *l*-[*1*-^11^C]leucine *via* a venous catheter as a slow bolus injection.^268^ Blood samples were collected *via* a radial arterial catheter over 60 min PET scan at different time intervals. According to the results, the mean plasma concentration of the sum of all large neutral amino acids was 13% higher in men than in women, and the plasma leucine concentration was found to be similar in both sexes.^268^ Cerebral protein synthesis^269^ and amino acid uptake^270^ in children were also studied by the same research group showing similar results.

### 4.9 Lysine

#### 4.9.1 Radiosynthesis

**Figure 64:**
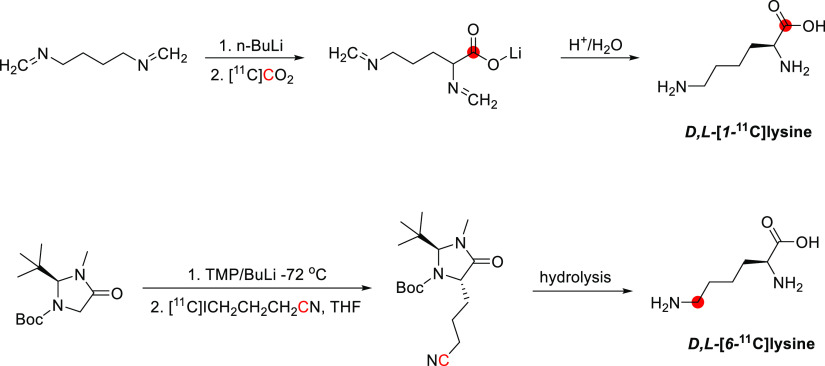
Synthesis of *d*,*l*-[*1*-^11^C]lysine using [^11^C]CO_2_ and *d*,*l*-[*6*-^11^C]lysine using [^11^C]ICH_2_CH_2_CH_2_CN. ^11^C radionuclide position is highlighted in red.

Labeling of lysine has been performed in positions *1*- or *6*- from two different groups, and no modifications or other methods have been published (Figure 64).^274,275^

*d*,*l*-[*1*-^11^C]Lysine was synthesized by carboxylation of α-lithioisocyanides, followed by hydrolysis and purification (RCY 8–14% and RCP >97%) within 50 min from EOB.^274,275^ Application of the direct carboxylation method requires the activation of the *α*-carbon, achieved by the abstraction of the proton on the *α*-carbon of *1*,*5*-pentylenediisocyanide with *n*-BuLi. The authors attempted to separate the enantiomers by HPLC using both chiral stationary phases and chiral mobile phases without success and suggested an enzymatic resolution method using an immobilized *d*-amino acid oxydase/catalase.^273^

To produce [*6*-^11^C]lysine, the anion of (*S*)-Boc-*2*-*tert*-butyl-3-methyl-*1*,*3*-imidazolidin-*4*-one was treated with lithium *2*,*2*,*6*,*6*-tetramethylpiperidide to initiate deprotonation of the *α*-carbon, followed by a ^11^C alkylation with *4*-[^11^C]iodobutyronitrile, and subsequent reduction and hydrolysis to furnish the *d*,*l*-[*6*-^11^C]lysine. However, these were only preliminary findings, and no details about the RCY, RCP, or ee were provided.^275^

#### 4.9.2 Preclinical Studies

**Figure 65:**
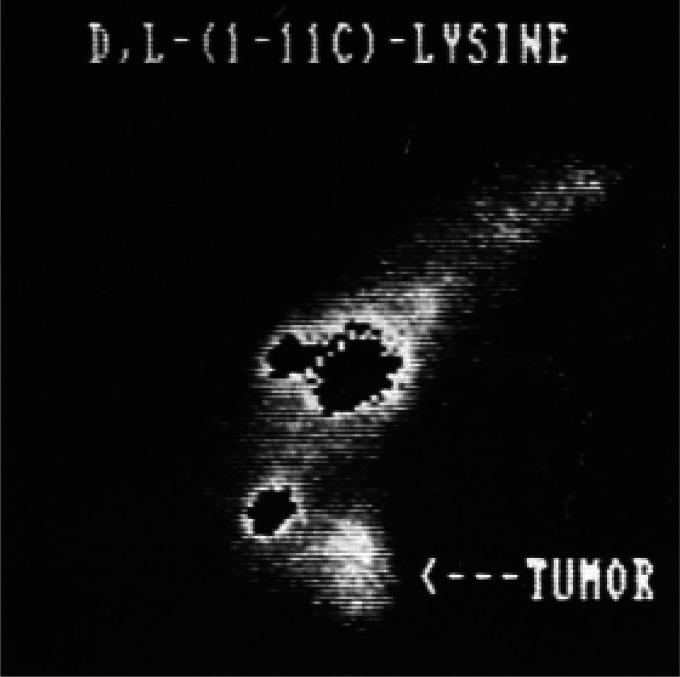
Distribution of *d*,*l*-[*1*-^11^C]lysine 5 min after injection in rats with a Walker 256 carcinosarcoma transplanted in the hind leg. Reproduced with permission from ref (273). Copyright 1985 Elsevier.

*d*,*l*-[*1*-^11^C]Lysine has been evaluated for tumor imaging in Wistar rats (Figure 65).^273^ Bolster *et al*. injected 1.85 MBq of *d*,*l*-[*1*-^11^C]lysine in the tail vein and examined radioactivity distribution at 5 min p.i. Unfortunately, the authors reported only the tumor/muscle ratio (4.5), and no other results were discussed. Therefore, a precise interpretation of the whole-body distribution cannot be given through imaging analysis.

### 4.10 Methionine

#### 4.10.1 Radiosynthesis

**Figure 66:**
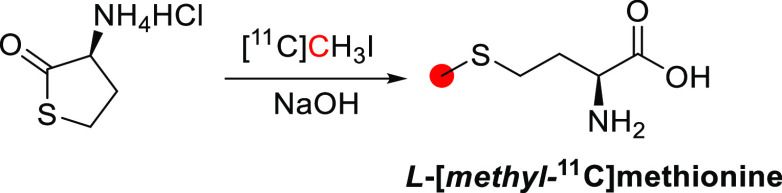
Synthesis of *l*-[*methyl*-^11^C]methionine using the precursor l-homocysteine thiolactone hydrochloride and [^11^C]CH_3_I. ^11^C radionuclide position is highlighted in red.

Methionine is a sulfur-containing amino acid that can be rapidly synthesized without complicated purification steps.^239^ Numerous synthetic pathways can be used based on the alkylation of the corresponding sulfide anion of l-homocysteine with either [^11^C]CH_3_I (Figure 66) or [^11^C]CH_3_OTf.^329^ Moreover, *l*-[*methyl*-^11^C]methionine can be efficiently produced by ^11^C-methylation of l-cysteine thiolactone hydrochloride or l-homocysteine in solution using the “bubbling” method,^330^ on a solid-phase extraction cartridge^282^ or by using the captive solvent method (“in the loop”) with or without semipreparative HPLC purification.

Langstrom *et al*. produced *l*-[*methyl*-^11^C]methionine *viaS-*^11^C-methylation of *l*-homocysteine in liquid NH_3_ using [^11^C]CH_3_I, obtaining a RCY of 65–90% within 30–40 min after EOB with a *A*_m_ of 0.4 GBq/μmol and RCP of 99.5%. This method is time-consuming and challenging to automate.^331^

Radiosynthesis and purification on solid supports (C18 Sep Pak cartridge) have gained more attention due to simplicity (room temperature, immobilized precursor, easier to automate) and shorter reaction time due to the elimination of semipreparative HPLC purification. Up to 60% ΜRCYs (at the EOB) have been reported, which provided enough activity for multiple patient administration from a single run in a short total synthesis time (<15 min).^332^ More recently, Gomzina *et al*. produced *l*-[*methyl*-^11^C]methionine *via*^11^C-methylation of *l*-homocysteine thiolactone hydrochloride on C18 solid-phase cartridges, resulting in the produced activity of 4–5.7 GBq, RCY of 75 ± 3% based on [^11^C]CH_3_I activity, RCP of 99.7 ± 0.2%, and enantiomeric purity of 93.7 ± 0.5%.^282^

Generally, a conventional method, using LiAlH_4_/HI, is used to produce [^11^C]CH_3_I, which gave higher overall yields but lower *A*_m_ than the “gas phase” method. However, free methionine concentration in blood plasma is around 5 mg/L. Therefore, the European Pharmacopeia does not regulate the *A*_m_ of *l*-[*methyl*-^11^C]methionine. Also, the maximum quantity of other byproducts (homocysteine precursor, homocysteine), which might be co-injected with methionine, would be negligible compared to the quantity ordinarily present in the blood plasma ruling out any metabolic interference with the radiotracer itself and byproducts.^332^

#### 4.10.2 Preclinical Studies

**Figure 67:**
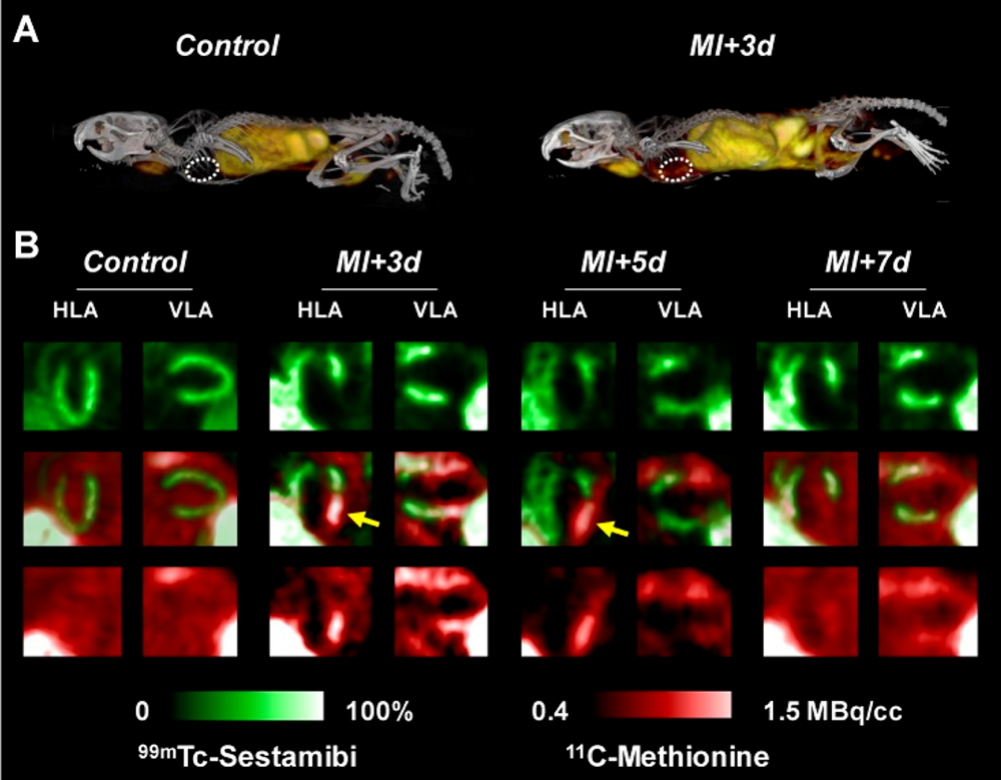
Maximum intensity projection (MIP) PET/CT images in a healthy mouse (red squared images) show no *l*-[*methyl*-^11^C]methionine uptake in the myocardium. Reproduced with permission from ref (277). Copyright 2016 Ivyspring. This work is licensed under a Creative Commons Attribution 4.0 International License (https://creativecommons.org/licenses/by/4.0/).

This radiopharmaceutical was evaluated *in vivo* in mice, rats,^214,276^ dogs, pigs, and monkeys.^279^ Thackeray *et al*. evaluated the whole-body distribution of the radiotracer in healthy mice. It accumulated in the kidneys and leukocyte-rich regions such as the spleen, thymus, bone marrow, liver, and metabolic organ. Lower uptake was also observed in blood, quadriceps, myocardium, and brain (Figure 67).^277^

**Figure 68:**
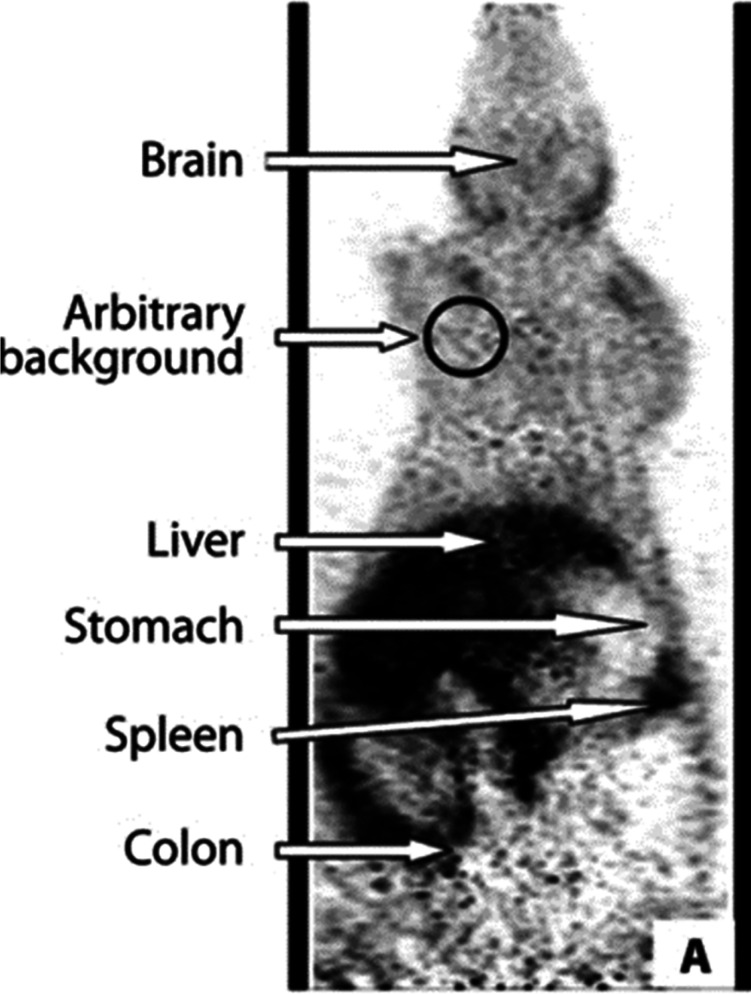
*l*-[*Methyl*-^11^C]methionine normal distribution in a rat liver, spleen, and distal part of the gastrointestinal tract. Reproduced with permission from ref (276). Copyright 2011 Slovak Toxicology Society SETOX. This work is licensed under a Creative Commons Attribution 4.0 International License (https://creativecommons.org/licenses/by/4.0/).

In 2011, Štolc *et al*. published a study on the physiological biodistribution of *l*-[*methyl*-^11^C]methionine in Wistar rats. Images were acquired 15 min after tracer administration revealing high uptake in the liver, followed by the spleen and colon. Low uptake in the brain and heart (Figure 68) was observed.^276^

**Figure 69:**
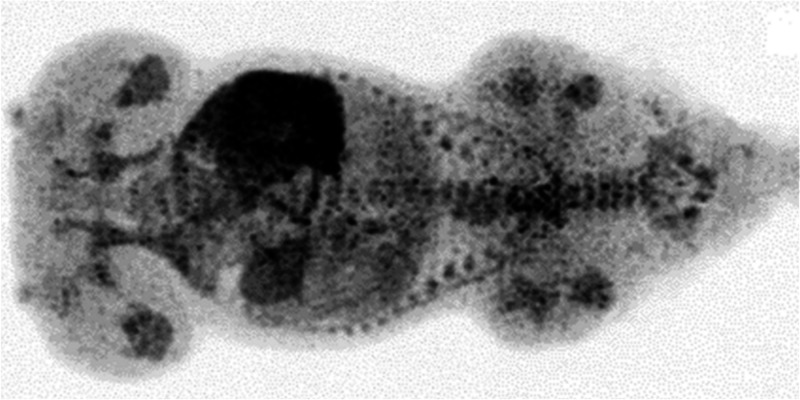
*l*-[*Methyl*-^11^C]methionine PET scan showing intense uptake in the liver and small intestines. Reproduced with permission from ref (333). Copyright 2016 AJNMMI. This work is licensed under a Creative Commons Attribution 4.0 International License (https://creativecommons.org/licenses/by/4.0/).

The accumulation of *l*-[*methyl-*^11^C]methionine in domestic, juvenile female pigs was also studied (Figure 69). The highest uptake was found in the small intestines, liver, kidney, thymus, pyloric antrum/duodenum, and bones. Reduced radiotracer uptake was also observed in the colon, heart, brain, and bladder.^333^

#### 4.10.3 Clinical Studies

**Figure 70:**
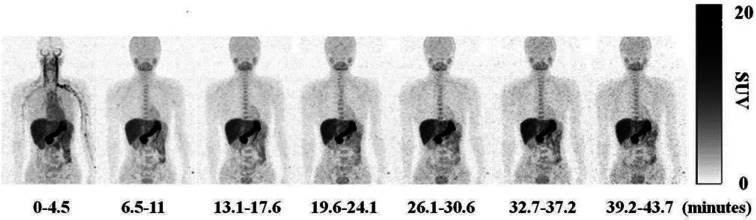
*l*-[*Methyl*-^11^C]methionine MIP PET images of a 24-year-old-healthy male showed increased liver and pancreas uptake for the whole scan duration. Reproduced with permission from ref (280). Copyright 2013 Isohashi *et al*., licensee Springer. This work is licensed under a Creative Commons Attribution 4.0 International License (https://creativecommons.org/licenses/by/2.0/).

A study by Isohashi *et al*. aimed to standardize the *l*-[*methyl*-^11^C]methionine protocol by performing dynamic images of the radiotracer for 43.7 min after administration in 11 healthy volunteers (Figure 70)^280^ Rapid blood clearance and high accumulation in the pancreas and liver were observed. High uptake was also observed in the stomach, kidney, and spleen. *l*-[*Methyl*-^11^C]methionine accumulation in the parotid glands and myocardium was moderate, and low uptake in the brain and lung was observed throughout the imaging period.^280^ These biodistribution observations agree with the above findings in healthy mice and rats^276,277^ but disagree with pigs.^333^

*l*-[*Methyl*-^11^C]methionine is one of the most commonly used radiotracers in PET imaging of brain tumors. Because of its relatively low uptake in brain tissue, *l*-[*methyl*-^11^C]methionine incorporates into most brain tumors, including low-grade gliomas, and its clinical use applies to a wide range of applications.^334,335^*l*-[*Methyl*-^11^C]methionine can also be used to study non-Hodgkin lymphoma, breast cancer, lung cancer, and melanoma.^336^*l*-[*Nethyl*-^11^C]methionine has also been used with high sensitivity in the preoperative localization of primary hyperparathyroidism to localize the abnormal parathyroid glands.^337^

Metabolite studies have been carried out in the plasma of eight patients with cancer. It was found that the total radioactivity remained constant after a rapid clearance with a rapid increase in protein-bound radioactivity. Plasma metabolite analysis revealed that the most abundant metabolites are *4*-methylthio-*2*-oxobutyrate and serine.^281,338^

### 4.11 *S*-*Methyl*-*l*-cysteine

#### 4.11.1 Radiosynthesis

**Figure 71:**
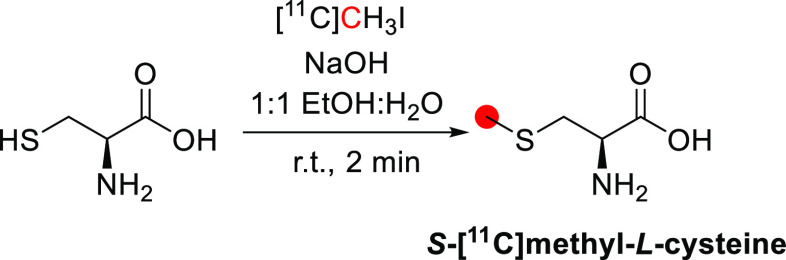
Synthesis of *S*-[^11^C]*methyl*-*l*-cysteine using [^11^C]CH_3_I. ^11^C radionuclide position is highlighted in red.

The radiosynthesis of *S*-[^11^C]*methyl*-*l*-cysteine was accomplished with a fully automated system by delivering [^11^C]CH_3_I into a cartridge loaded with a solution of *l*-cysteine and NaOH in ethanol:water (Figure 71).^283^ The product is then eluted from the cartridge using phosphate buffer, returning the desired *S*-[^11^C]*methyl*-*l*-cysteine within 12 min from EOB with an activity yield of >50% (based on [^11^C]CH_3_I activity).^283^ Quality control was also performed to verify the final product’s radiochemical and chemical purity, pH, and sterility for human use.^283^ The same radiochemical methodology was also employed in the synthesis of the d isomer (*S*-[^11^C]*methyl*-*d*-cysteine), which was obtained with the same RCY and purity.^339^

#### 4.11.2 Preclinical Studies

C57BL/6J mice (9–10 weeks old, female and male) were prepared by either inducing inflammation or inoculating tumor while a group was unmodified and used as control.^283^ The mice were injected with the radiotracer and the uptake was initially assessed, revealing a high uptake in the liver, stomach, and heart. A quick washout and poor *S*-[^11^C]*methyl*-*l*-cysteine protein binding were also identified.^283^ Whole-body PET studies were executed to compare the efficacy of *S*-[^11^C]*methyl*-l-cysteine against the clinical standards *S*-[^11^C]*methyl*-*l*-cysteine and [^18^F]FDG, highlighting a much higher tumor-to-muscle ratio and low uptake in inflammation models for *l*-[*S*-*methyl*-^11^C]cysteine compared to[^18^F]FDG, suggesting that *S*-[^11^C]*methyl*-*l*-cysteine may be a much more specific radiopharmaceutical for cancer staging.^283^ Subsequent studies by Huang *et al*. explored the efficacy of the *d* isomer, *S*-[^11^C]*methyl*-*d*-cysteine, in tumor-bearing (S180 fibrosarcoma) and inflammation model (turpentine-induced) mice. The results suggest that *S*-[^11^C]*methyl*-*d*-cysteine has a higher specificity towards tumors than the l isomer.^339^ The specificity of *S*-[^11^C]*methyl*-*l*-cysteine for brain tumor imaging was then investigated by administering the radiopharmaceutical to glioma-implanted Male Wistar rats.^340^

#### 4.11.3 Clinical Studies

**Figure 72:**
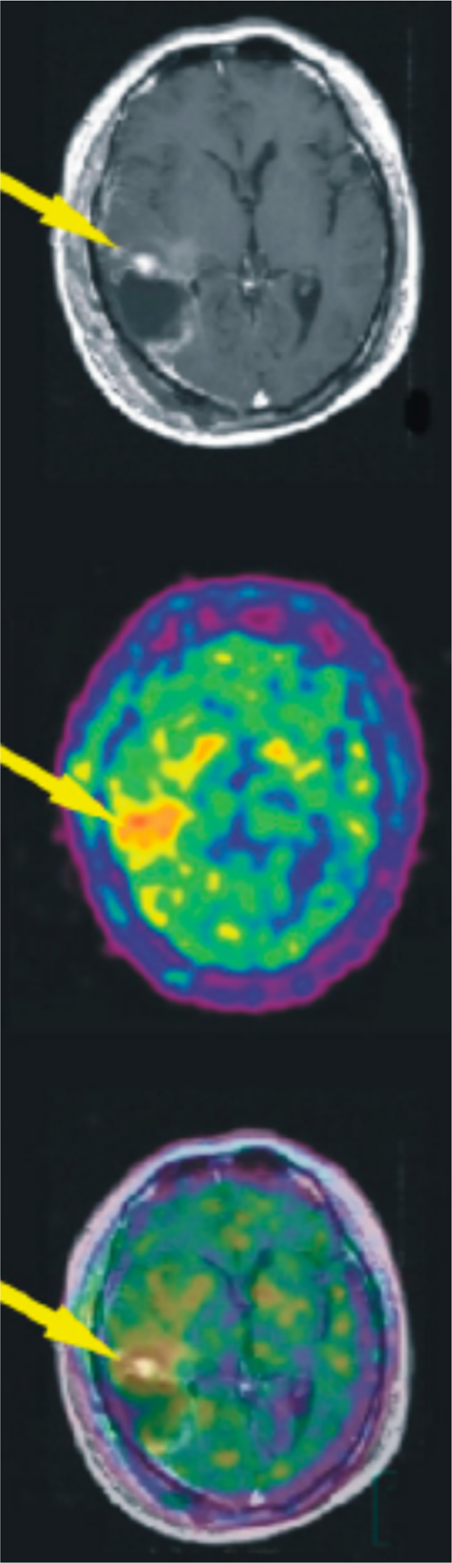
*S*-[^11^C]*Methyl*-*l*-cysteine MRI, PET, and coregistered PET/MRI images (from top to bottom) show a hypermetabolic lesion (arrow) in the same area where the tumor was located *via* MRI. Reproduced with permission from ref (283). Copyright 2011 Society of Nuclear Medicine. This work is licensed under a Creative Commons Attribution 4.0 International License (https://creativecommons.org/licenses/by/4.0/).

A 45-year-old patient with grade iv glioma was scanned with *S*-[^11^C]*methyl*-*l*-cysteine, showing a specific signal (Figure 72). Further studies focused on depicting the human biodistribution and radiation dosimetry following *S*-[^11^C]*methyl*-*l*-cysteine administration.^284^ For this purpose, six healthy volunteers (six men and six women aged 41–56 years) were injected and scanned for 70–85 min. All volunteers showed high retention of activity in the liver, spleen, pancreas, heart, kidneys, and uterus (only in women), which quickly washed out. The total body radiation dose showed the most affected organ to be the liver.^284^ Given the reported data, the radiation-absorbed dose is considered within accepted limits and *S*-[^11^C]*methyl*-*l*-cysteine to be a safe radiopharmaceutical for cancer staging.^284^

### 4.12 Norleucine

#### 4.12.1 Radiosynthesis

Labeling of norleucine has been performed in positions 1 or 3 from two different groups. *d*,*l*-[*1*-^11^C]Norleucine was synthesized by a no carrier added synthesis method. This method was described in detail by Iwata *et al*. in 1987,^285^ where [^11^C]HCN was produced by the catalytic reaction of [^11^C]CH_4_ on Pt at 950 °C and directly bubbled into a reaction solution that contained sodium *1*-aminopentane-*1*-sulfonate. The mixture was heated for 10 min, and the aminonitrile was then extracted with ether. After acid hydrolysis, *d*,*l*-[*1*-^l1^C]norleucine was purified. The preparation was carried out with RCY of 35% within 60 min after EOB.^214,285^

**Figure 73:**
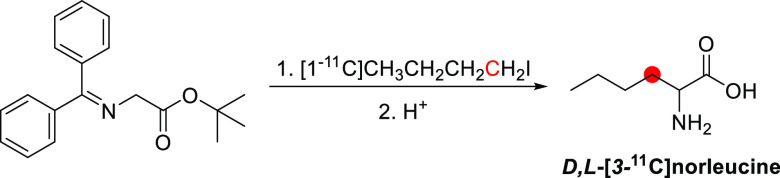
Synthesis of *d*,*l*-[*3*-^11^C]norleucine using [*1*-^11^C]CH_3_CH_2_CH_2_CH_2_I.^272^^11^C radionuclide position is highlighted in red.

For the labeling in the *3*-position, Antoni *et al.*, in 1987, performed a phase-transfer alkylation reaction on *N*-(diphenylmethylene)-glycine *tert*-butyl ester with [*1*-^11^C]CH_3_CH_2_CH_2_CH_2_I followed by acidic hydrolysis (Figure 73). The labeled norleucine was obtained in RCY of 10% and RCP of 97% after a total synthesis time of about 100 min.^286^

#### 4.12.2 Preclinical Studies

*d*,*l*-[*1*-^11^C]Norleucine, identical to *d*,*l*-[*1*-^11^C]leucine^214^ and *d*,*l*-[*1*-^11^C]methionine,^214^ has been evaluated *in vivo* in young male Donryu rats inoculated with transplantable ascitic hepatoma (AH109A) cells. In the study, five rats were used for each data point. The animals fasted for 24 h, and *d*,*l*-[*1*-^11^C]norleucine was injected intravenously. The authors reported a low tumor/blood, tumor/muscle, and tumor/liver ratio at 20 min p.i. Also, a low pancreas uptake was reported; however, no other outcomes were mentioned.^214^

### 4.13 Norvaline

#### 4.13.1 Radiosynthesis

**Figure 74:**
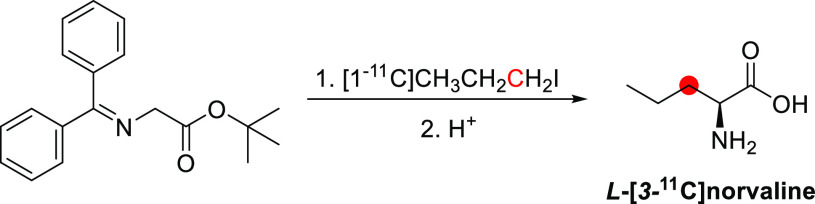
Synthesis of *d*,*l*-[*3*-^11^C]norvaline using [*1*-^11^C]CH_3_CH_2_CH_2_CH_2_I.^272^^11^C radionuclide position is highlighted in red.

*l*-[*3*-^11^C]Norvaline was synthesized by asymmetric alkylation of *N*-(diphenylmethylene)-glycine *tert*-butyl ester using [*1*-^11^C]CH_3_CH_2_CH_2_I and, as a final step, the hydrolysis of the protecting groups (Figure 74). The final product was isolated with RCY of 25%, RCP of 99%, and ee 80%. After HPLC purification, *l*-[*3*-^11^C]norvaline was isolated within 20 min.^272^

### 4.14 Ornithine

#### 4.14.1 Radiosynthesis

**Figure 75:**
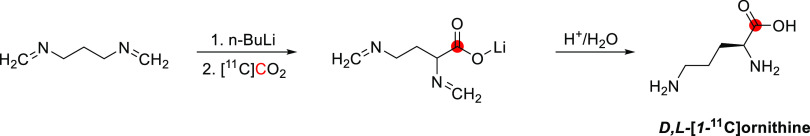
Synthesis of *d*,*l*-[*1*-^11^C]ornithine using [^11^C]CO_2_.^273^^11^C radionuclide position is highlighted in red.

Labeling of ornithine has been performed in positions *1*- or *5*- from two different groups, and no modifications or other method has been published.^272,273^ In position *1*-, *d*,*l*-[*1*-^11^C]ornithine was synthesized with the same procedure as described for *d*,*l*-[*1*-^11^C]lysine by carboxylation of *α*-lithioisocyanides, followed by hydrolysis and isolation, with RCY of 8–14% and RCP >97% within 50 min after EOB (Figure 75).^273^ For the labeling in the *5*- position, a displacement reaction of potassium [^11^C]cyanide with the functionally protected *y*-bromohomoserine followed by selective reduction of the [^11^C]nitrile with cobalt chloride-sodium borohydride complex, deprotection with 6 M HCl, and purification by HPLC gave the *l*-[*5*-^11^C]ornithine with RCY of 25–40% and *A*_m_ > 77.7 GBq/μmol within 50 min after EOB.^272^

#### 4.14.2 Preclinical Studies

*d*,*l*-[*1*-^11^C]Ornithine, similar to *d*,*l*-[*1*-^11^C]lysine, has been evaluated *in vivo* in both male and female Wistar rats to detect tumors.^273^ The authors reported a good tumor/muscle ratio without mentioning other outcomes. A high accumulation in the liver, kidney, and bladder can be observed through imaging analysis. However, the image lacks a precise interpretation of the whole-body distribution.

### 4.15 Phenylalanine

#### 4.15.1 Radiosynthesis

**Figure 76:**
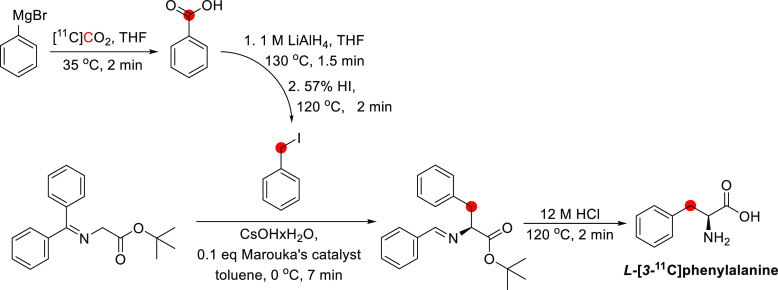
Synthesis of *l*-[*3*-^11^C]phenylalanine using [^11^C]CO_2_ by applying a phase-transfer reaction/catalysis.^217^^11^C radionuclide position is highlighted in red.

[^11^C]Phenylalanine has been labeled at the *1*- (the *carboxyl*) and *3*-carbon positions.^211,341^ Racemic carboxyl-labeled *d*,*l*-[*1*-^11^C]phenylalanine was prepared by a modified Bücherer–Strecker synthesis using [^11^C]cyanide^212,215,287^ as a radiolabeling agent and by ^11^C-carboxylation of the activated α-lithioisonitrile precursor with [^11^C]CO_2_ followed by acid hydrolysis of the intermediate.^213,288^ On the other hand, [*3*-^11^C]phenylalanine was prepared by ^11^C-alkylation either *via* condensation reaction of [^11^C]benzaldehyde with *2*-aryl-*5*-oxazolone^342,343^ and by ^11^C-benzylation of commercially available *N*-(*diphenylmethylene*)glycine ester by applying a phase-transfer reaction/catalysis.^217,344−346^ Also, the biosynthetic/enzyme approach was used either for the resolution of racemic *d*,*l*-[*1*-^11^C]phenylalanine^215,272,345^ (which was later replaced by chiral HPLC) or for the preparation of pure *l*-[*1*-^11^C]phenylalanine^343,347^ starting from the corresponding ^11^C-labeled precursor. Considerable progress has been made in the enantioselective preparation of l-[*3-*^11^C]phenylalanine,^346,347^ and ^11^C-radiolabeled amino acids at the *3*-carbon during the last three decades.^239,348−350^ In this regard, Pekošak *et al*. have recently reported rapid and efficient five-step radiosynthesis to obtain selectively *l*-[*3*-^11^C]phenylalanine with ee >90% by utilizing enantioselective ^11^C-alkylation of *tert*-butyl ester protected Schiff base glycine precursor in the presence of selected chiral quaternary ammonium salt phase-transfer catalyst without chiral separation as shown in Figure 76.^217^ The first three steps comprised of a one-pot preparation of [^11^C]benzyl iodide as the ^11^C-labeled synthon within 11 min starting from ^11^C-carboxylation of phenylmagnesium bromide (Grignard reagent) and subsequent reduction, iodination, and K_2_CO_3_/Mg_s_SO_4_ column purification.^351^ Afterwards, [^11^C]benzyl iodide dissolved in toluene was slowly transferred in the second reaction vessel to allow asymmetric [^11^C]benzylation (radiochemical conversion (RCC) >90%) at the *α*-carbon of the commercially available *N*-(diphenylmethylene)glycine *tert*-butyl ester in the presence of l-selective Maruoka chiral PTC and the excess of CsOH×H_2_O at 0 °C. In the last step, the quantitative deprotection under acidic conditions yielded *l*-[*3*-^11^C]phenylalanine as the desired product. The radiosynthesis of enantiomerically pure product was also scaled up and fully automated on the in-house built platform under optimized conditions with a total synthesis time of 24 min, and RCY of 27 ± 7%, calculated from EOB. The *A*_m_ of the product was 85–135 GBq/μmol at EOS. This general radiosynthesis methodology has enabled the preparation of enantiopure *d*-[*3*-^11^C]phenylalanine^217^ using *d*-selective Maruoka chiral PTC and *l*-[^11^C]alanine,^242^ which the same group previously reported. This strategy would allow the preparation of carbon-11 radiolabeled peptides as well.

#### 4.15.3 Preclinical Studies

*d*,*l*-[*1*-^11^C]Phenylalanine has been evaluated in young male Donryu rats inoculated with transplantable ascitic hepatoma (AH109A) cells. The animals fasted for 24 h, and *d*,*l*-[*1*-^11^C]phenylalanine was injected through the vein. The authors reported a low tumor/blood, tumor/muscle, and tumor/liver ratio at 20 min p.i. Also, a low pancreas uptake was reported; however, no other outcomes were mentioned.^214^

### 4.16 Phenylglycine

#### 4.16.1 Radiosynthesis

**Figure 77:**
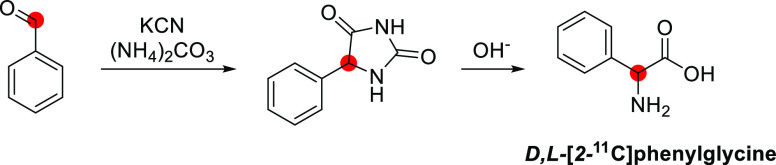
Synthesis of *d*,*l*-[*2*-^11^C]phenylglycine using [^11^C]benzaldehyde. ^11^C radionuclide position is highlighted in red.

*d*,*l*-[*1*-^11^C]Phenylglycine was prepared for the first time by a method developed by Vaalburg *et al*. in 1976.^288^ The method is based on the carboxylation of *α*-lithiobenzylisocyanide with [^11^C]CO_2_, followed by acid hydrolysis. *d*,*l*-[*1*-^11^C]Phenylglycine was obtained with a RCY of 6% and specific activity (*A*_s_) of 0.037 GBq/mg (Figure 77).^288^ A new method for preparing the *d*,*l*-[*2-*^11^C]phenylglycine was published almost 10 years later. Specifically, they first prepare the [^11^C]benzaldehyde in a two-step reaction from the corresponding [^11^C]benzoic acid salt *via* the [^11^C]benzyl alcohol, starting with a reaction of [^11^C]CO_2_ with phenylmagnesium bromide. Then through a modified Bücherer–Strecker reaction with ammonium carbonate and potassium cyanide, followed by hydrolysis, *d*,*l*-[*2-*^11^C]phenylglycine was isolated with a RCY of 2–5% and RCP of 99% within 50 min.^289^

#### 4.16.2 Preclinical Studies

*d*,*l*-[*1*-^11^C]Phenylglycine has been evaluated *in vivo* in young male Donya rats inoculated with transplantable ascitic hepatoma (AH109A) cells. In the study, five rats were used for each data point. The animals fasted for 24 h, and *d*,*l*-[*1*-^11^C]phenylglycine was administered. The authors reported a low tumor/blood, tumor/muscle, and tumor/liver ratio at 20 min p.i. Also, a low pancreas uptake was reported; however, no other outcomes were mentioned.^214^

### 4.17 Proline

#### 4.17.1 Radiosynthesis

**Figure 78:**
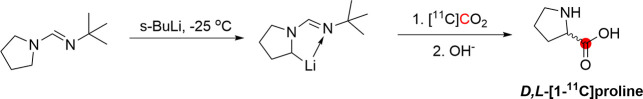
Synthesis of *d*,*l*-[*1*-^11^C]proline using [^11^C]CO_2_. ^11^C radionuclide position is highlighted in red.

*d*,*l*-[*1*-^11^C]Proline has been synthesized only with one method described by Bolster *et al*. in 1985 (Figure 78). The preparation was achieved by carboxylation of *α*-lithiopyrrolidyl-*N*-*tert*-butyl-formamidine followed by regeneration of the amine functionality with a RCY of 12-18% and RCP >95% within 45 min. However, no attempts have been made to resolve the *d*,*l*-[*1*-^11^Clproline in its enantiomers.^273^

#### 4.17.2 Preclinical Studies

**Figure 79:**
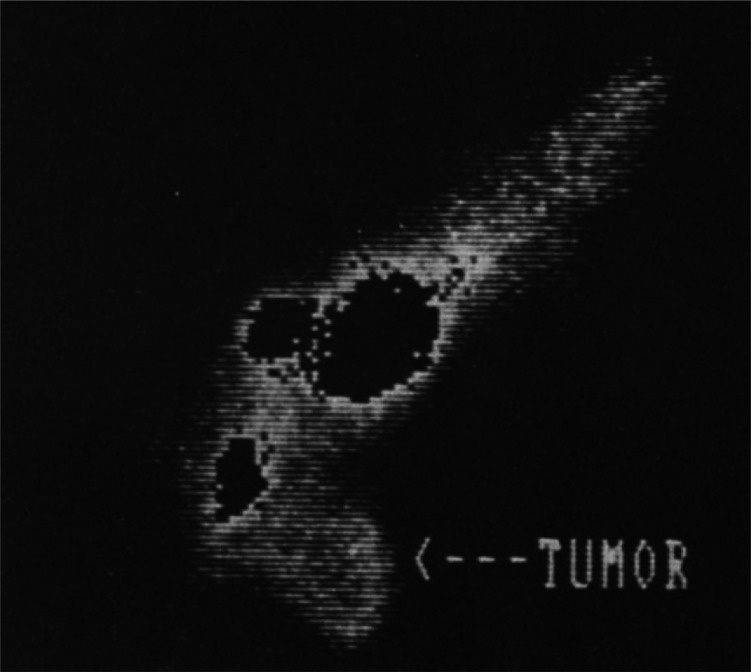
*d*,*l*-[*1*-^11^C]Proline distribution in rats with Walker 256 carcinosarcoma transplanted in the hind leg (5 min p.i.). Reproduced with permission from ref (273). Copyright 1985 Elsevier.

*d*,*l*-[*1*-^11^C]Proline has been evaluated *in vivo* in Wistar rats to detect Walker carcinosarcoma tumors.^273^ The authors reported a good tumor/non-tumor ratio at 45 min p.i. without mentioning any other results. However, image analysis shows a high accumulation in the liver, kidney, and bladder (Figure 79).

### 4.18 Serine

#### 4.18.1 Radiosynthesis

**Figure 80:**
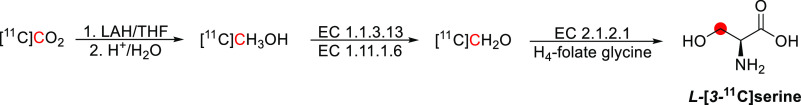
Enzymatic synthesis of *l*-[*3*-^11^C]serine using [^11^C]CO_2_ in four steps.^290^^11^C radionuclide position is highlighted in red.

A multi-enzymatic synthesis of carbon-11 labeled *l*-serine was published in 1990. The synthesis of *l*-[*3*-^11^C]serine from [^11^C]CO_2_ was performed in four steps (Figure 80). The first step was the reduction of [^11^C]CO_2_ to [^11^C]CH_3_OH, followed by selective oxidation by alcoholoxidase to [^11^C]formaldehyde, which was then condensed nonenzymatically with H_4_-folate. Finally, the *N*,^5^*N*^10^-[^11^C]methylenetetrahydrofolate was transferred to glycine and catalyzed by serine hydroxymethyltransferase and pyridoxalphosphate. *l*-[*3*-^11^C]Serine (*A*_m_ 0.30–1.85 GBq/μmol) was obtained in a RCY l–2% within 45–50 min from the EOB.^290^

For the preparation of *d*-[^11^C]serine, a Ni^II^-complex of the Schiff base of (*S*)-*N*-(2-benzoylphenyl)-*1*-benzylpyrrolidine-*2*-carboxamide with glycine was added to [^11^C]CH_2_O prepared by the oxidation of [^11^C]CH_3_I. This glycine synthon enables the creation of desired stereochemistry of the chiral center of *d*-serine with a RCY >50% based on [^11^C]CH_3_I and a high diastereomeric excess (80%) in a 1 min reaction.^201^

### 4.19 Tryptophan/*5*-Hydroxytryptophan

#### 4.19.1 Radiosynthesis

**Figure 81:**
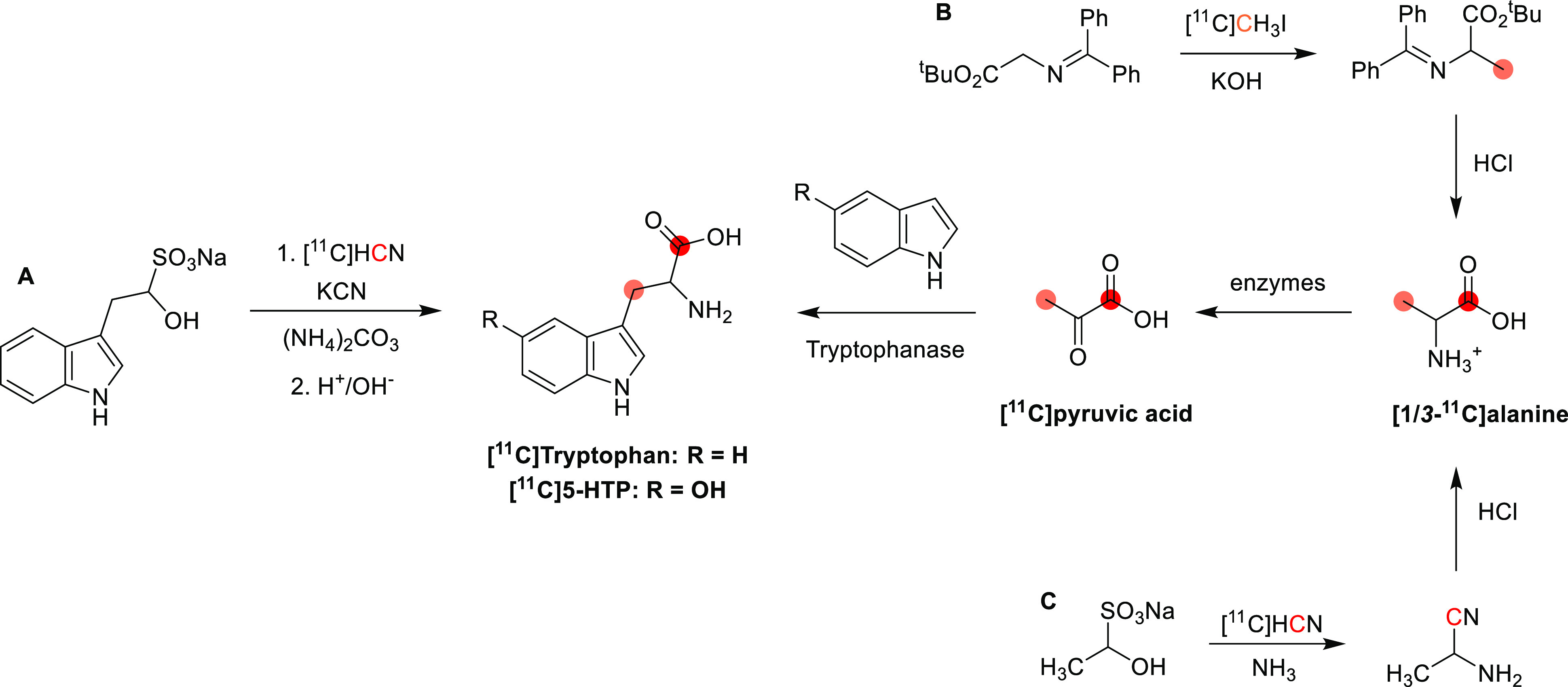
Radiosynthetic routes to [*carboxyl*-^11^C]tryptophan/*5*-HTP and [*β*-^11^C]tryptophan/*5*-HTP using [^11^C]CH_3_I or [^11^C]HCN. ^11^C radionuclide position is highlighted in red and orange.

Initial efforts focused on synthesizing racemic [*carboxyl*-^11^C]tryptophan. This was first reported in 1978 by Hayes *et al.*,^324^ utilizing the Bucherer–Bergs reaction (Figure 81A). In this process, the bisulfite adduct derived from 3-indoleacetaldehyde reacts with carrier-added [^11^C]cyanide and ammonium carbonate at high temperatures to generate the corresponding hydanton intermediate. Subsequent hydrolysis using NaOH produces *d*.*l*-[*carboxyl*-^11^C]tryptophan in 30-40% RCY (based on CN^–^) following SPE purification, lasting 40 min.^291,324^ This process was modified for routine clinical use by Zalutsky *et al*. in 1981, including HPLC purification, achieving a reduced synthesis time of 28 min, and RCYs of ∼50% (based on CN^–^).^352^

In 1989, researchers at Uppsala University reported the synthesis of enantiomerically pure *l*-tryptophan and *l*-*5*-HTP radiolabeled in the metabolically stable *β*-position *via* a multienzymatic reaction using racemic [*3*-^11^C]alanine, prepared by ^11^C-methylation of a glycine derivative (Figure 81B).^353^ Enzymatic syntheses were carried out in a one-pot reaction using d-amino acid oxidase/catalase and glutamic-pyruvic transaminase to produce [^11^C]pyruvic acid, which was converted to *l*-[*β*-^11^C]tryptophan or *l*-[*β*-^11^C]5-HTP by the action of the tryptophanase enzyme in the presence of indole or 5-hydroxyindole, respectively (synthesis time, 50–55 min; RCY,: 25% (from [^11^C]CO_2_), RCP > 98%; *A*_m_, 2.5 GBq/μmol). This procedure has been used for numerous preclinical and clinical PET investigations with [*β*-^11^C]*5*-HTP and, in 2006, was implemented at the University of Groningen (synthesis time: 50 min, RCY: 24% (from [^11^C]CH_3_I), RCP > 99%; *A*_m_, 44 GBq/μmol).^220^

Tryptophan and *5*-HTP labeled in the carboxyl position can also be produced by the multienzymatic reaction utilizing [*1*-^11^C]alanine as the feedstock (Figure 81C).^354^ [*1*-^11^C]Alanine is produced by Strecker reaction of the bisulfite adduct of acetaldehyde with [^11^C]cyanide and ammonia, followed by hydrolysis of the resulting ^11^C-aminonitrile (synthesis time, 50 min; RCY, 45-60% (based on [^11^C]CN^–^), RCP > 98%; *A*_m_,, 0.4–2.0 GBq/μmol).

#### 4.19.2 Preclinical Studies

In rhesus monkeys, [*β*-^11^C]tryptophan produced a homogenous distribution of radioactivity in the brain, with a near-zero rate constant for striatal uptake, suggesting low conversion to serotonin.^218^ Rat biodistribution experiments using [*carboxyl*-^11^C]tryptophan showed the highest tracer uptake in the muscle, lung, spleen, and pancreas at 30 min.^291^

Preclinical experiments with [^11^C]*5*-HTP have been performed to study AADC activity/serotonin synthesis in the rhesus monkey,^218,294−296^ rat,^297−300^ and mice.^301^ A study of [*β*-^11^C]HTP metabolism in monkeys found that the parent fraction in plasma gradually decreased to ∼50% at 60 min, with [^11^C]hydroxyindoleacetic acid ([^11^C]HIAA) being the major metabolite (39%).^296^ Lundquist *et al*. found that [*β*-^11^C]HTP PET was suitable for probing the decarboxylase step in serotonin synthesis in the monkey brain,^295^ although Visser *et al*. concluded that this was not the case in rodent brain.^299^ Biodistribution studies of [*β*-^11^C]HTP in rat^298,299^ found the highest radioactivity uptake in the kidneys and pancreas, with lesser uptake in the duodenum, spleen, stomach, and liver at 60 min.^298^ Pancreas uptake may be relevant because serotonin regulates insulin secretion by *β*-cells in the islets of Langerhans.

#### 4.19.3 Clinical Studies

AADC, which converts *5*-HTP to serotonin, is widely distributed in the human body, with the highest peripheral expression in the GI tract, kidney, and liver^355^ while in the brain, it is widely expressed in monoaminergic neurons.^295^ Accordingly, [*β*-^11^C]*5*-HTP PET has been used to probe serotonin biosynthesis in various states of health and disease. [*β*-^11^C]5-HTP brain PET has been used to measure serotonin synthesis rates in healthy subjects^302−307^ and psychiatric disorders such as depression,^302^ social anxiety disorder,^305,306,356^ and premenstrual dysphoria.^307,357^ Outside of the brain, [*β*-^11^C]*5*-HTP has been used to detect endocrine tumors,^358−370^ based on the amine precursor uptake and decarboxylation concept in which endocrine tumor cells are known to take up *5*-HTP for conversion to serotonin.^371^ [*β*-^11^C]*5*-HTP has also been used as an endocrine marker to study diabetes.^300,362,372,373^

[*β*-^11^C]*5*-HTP is rapidly cleared from the blood (<10% in blood at 20 min)^374^ but is metabolically stable (80% unchanged tracer present in plasma at 60 min, alongside ≤16% [^11^C]HIAA and ≤4% [^11^C]serotonin).^304^ Radioactivity uptake is observed in the kidneys, renal system, and the bladder, with minimal activity in all soft tissue structures.^374^ In the brain, radioactivity concentrations in the basal ganglia are about two times higher than those in cortical areas, reflecting the higher expression of serotonergic neurons. The highest accumulation rate constants were observed in the putamen and the caudate.^304^

The use of [^11^C]tryptophan as a PET probe in humans is limited to early studies by Hubner *et al*. using ^11^C-carboxyl-labeled tryptophan for pancreatic imaging,^292^ and to study human brain tumors.^293^

### 4.20 Tyrosine

#### 4.20.1 Radiosynthesis

**Figure 82:**
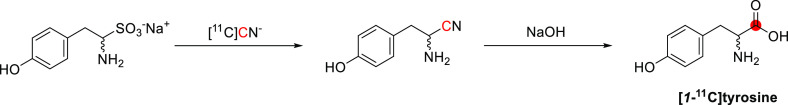
Synthesis of [*1*-^11^C]tyrosine using [^11^C]CN^–^.^11^C radionuclide position is highlighted in red.

The primary reaction path of *l*-tyrosine is *via* incorporation into proteins. However, significant decarboxylation can occur; thus, the labeling of the carboxylic group is essential.^375^ Studies reported in this section labeled tyrosine with ^11^C at the carbon of carboxylic acid. Production of [*1*-^11^C]tyrosine based on isocyanide route with [^11^C]CO_2_ was for the first time reported for both preclinical and clinical studies in 1986 by Bolster *et al*. [*1*-^11^C]tyrosine was prepared semiremotely *via* carboxylation of the appropriate α-lithioisolyanide, followed by hydrolysis of the isocyanide function and removal of the protecting methoxy group, with the second butyllithium as the base to lithiated *p*-methoxyphenylethylisocyanide and tetrahydrofuran. This reaction resulted in low RCY, 2–4%.^375^ The microwave-induced Bucherer–Strecker synthesis method is the most common [*1*-^11^C]tyrosine synthesis method. Groot *et al*. successfully synthesized [*1*-^11^C]tyrosine *via* this approach, affording a RCP >95%, an average activity yield of 800 MBq, and a *A*_m_ >29.20 GBq/μmol.^203^ An improvement in RCY was possible using the Studenov *et al*. method (Figure 82): RCY of 15.0 ± 4.0%, *A*_m_ of 74–111 GBq/μmol, and a synthesis time of 40–45 min starting from [^11^C]cyanide.^287^ More recently, Hienzsch *et al*. reported the radiolabeling of [*1*-^11^C]tyrosine using a microfluidic platform and an asymmetric version of the Strecker synthesis, resulting in a RCY of 39.0 ± 6%.^239^

#### 4.20.2 Preclinical Studies

In the late 1980s, the application of tyrosine was characterized in preclinical studies, and the first published *in vivo* was also reported as a first-in-human study.^239^ [*1*-^11^C]tyrosine has been studied in mice and rats, but no biodistribution studies in healthy animals have been performed.

#### 4.20.3 Clinical Studies

To our knowledge, no studies have assessed the whole-body biodistribution of [*1*-^11^C]tyrosine in healthy humans. However, Halldin *et al*. studied the regional uptake of radioactivity in the human brain of a 27-year-old healthy male volunteer. After crossing the BBB, the radiotracer accumulated in cortical and subcortical structures, especially in the thalamus, occipital, and limbic cortex.^308^

**Figure 83:**
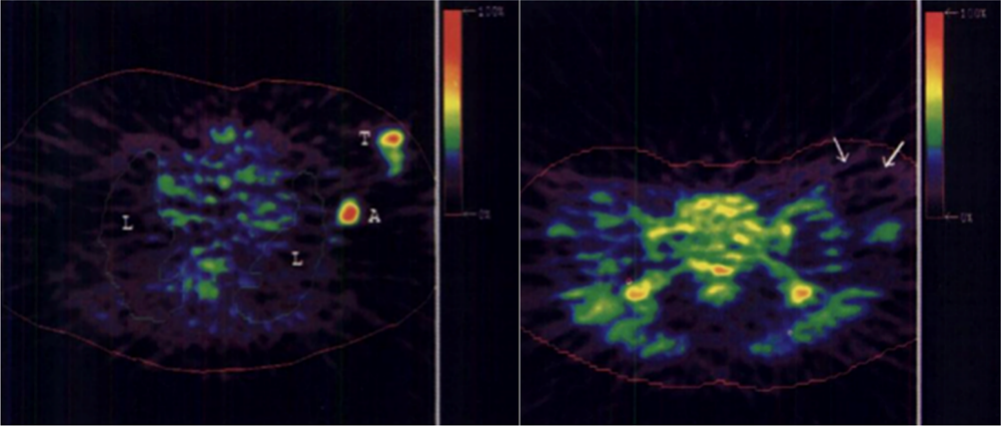
[*1*-^11^C]Tyrosine uptake in a patient with ductal carcinoma and metastatic lesion in the axilla (left image) and right inguinal region (right image, indicated by arrows). Reproduced with permission from ref (309). Copyright 1997 Society of Nuclear Medicine. This work is licensed under a Creative Commons Attribution 4.0 International License (https://creativecommons.org/licenses/by/4.0/).

In the last decade, [*1*-^11^C]tyrosine has been subjected to exciting applications. Kole *et al*. evaluated [*1*-^11^C]tyrosine to measure protein synthesis rate and tumor visualization.^309^ Twenty-two patients were injected with the radiotracer, followed by a 50 min of dynamic study performed immediately after administration (Figure 83). [*1*-^11^C]Tyrosine accumulation in various types of malignancy was high, whereas in benign lesions, the uptake was low, and this uptake was moderately correlated with the protein synthesis rate quantification.^309^

Because tyrosine is an essential AA in patients with phenylketonuria, a metabolic disorder caused by elevated blood levels of phenylalanine, Hoeksma *et al*. used [^11^C]tyrosine to study the relationship between the *in vivo* plasma phenylalanine concentrations and protein synthesis rate in 16 patients presented with this disease. Results revealed a significant association between the patient’s age and increased plasma phenylalanine with decreased protein synthesis rate in the brain.^376^

Although [^11^C]tyrosine was first used to measure protein synthesis *in vivo*, nowadays, the AA transport for imaging tumor metabolism has also been evaluated. Studies performed in patients with prolactinomas, a benign pituitary gland tumor that produces an excessive amount of the hormone prolactin, assessed the effect of bromocriptine treatment, showing the potential use of this radiotracer for investigating the salivary glands. The incorporation of tyrosine explains this into secretory proteins synthesized in salivary glands.^239,377,378^ However, a later study to detect cervical metastases revealed the unsuitability of [^11^C]tyrosine due to the increased bilateral uptake in the salivary glands, which impaired the visualization of metastases located within this area.^378^ Therefore, [^11^C]tyrosine is probably unsuitable for imaging the liver, pancreas, or salivary glands due to high accumulation in these organs.

In humans, the plasma metabolism of [^11^C]tyrosine has been studied in patients with primary or recurrent brain tumors. It was found that at 40 min p.i., more than 50% of total plasma radioactivity was from the plasma metabolites [^11^C]CO_2_, ^11^C-proteins, and l-[^11^C]dihydroxyphenylalanine (DOPA). Moreover, the [^11^C]CO_2_ level became significant within 5 min p.i. and reached a plateau of 25% of total plasma radioactivity at 20 min p.i. ^11^C-proteins were negligible for the first 20 min, and l-[^11^C]DOPA was the only acid-soluble radioactive metabolite detected with levels ≤8% at 40 min p.i.^379^

### 4.21 Valine

#### 4.21.1 Radiosynthesis

**Figure 84:**
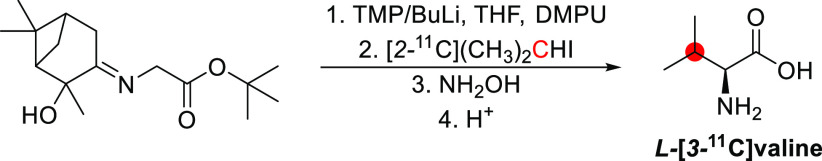
Synthesis of *l*-[*3*-^11^C]valine using [*2*-^11^C](CH_3_)_2_CHI.^286^^11^C radionuclide position is highlighted in red.

Valine has been labeled with carbon-11 in *1*- or *3*- position. A method for labeling *d*,*l*-[*1*-^11^C]valine was published in 1978.^311^ It was synthesized and purified by procedures analogous to those used for the production of *1*-[^11^C]aminocyclopentanecarboxylic acid ([^11^C]ACPC),^312^ except that isobutyraldehyde replaces the cyclopentanone used in the [^11^C]ACPC synthesis. Briefly, the aldehyde is labeled with [^11^C]KCN under high temperature and pressure to give the hydantoin, which is then hydrolyzed with a base at a high temperature. The RCY for the two-step synthesis of the racemic *d*,*l*-[*1*-^11^C]valine was approx 70% with an RCP >95%. The *A*_m_ was 0.56–1.30 GBq/mg at the EOS, which required approximately 45 min. The most recent method for labeling valine to position *3*- and high enantiomeric excess was published almost 10 years later.^286^*l*-[*3*-^11^C]valine was synthesized by asymmetric alkylation of [(+)-*2*-hydroxypinanyl-*3*-idene]glycine *tert*-butyl ester using [*2*-^11^C](CH_3_)_2_CHI and as a final step, the hydrolysis of the protecting groups (Figure 84). The final product was isolated with RCY 9%, high RCP 99%, and ee 80%.^286^

#### 4.21.2 Preclinical Studies

*d*,*l*-[*1*-^11^C]Valine was evaluated *in vivo* in 1978. Washburn *et al*. performed the biodistribution studies of *d*,*l*-[*1*-^11^C]valine in healthy male Fischer 344 rats and sex mongrel dogs. At that time, it was assumed that d-AAs were not metabolized as the l-AAs and remained in the blood flow. Thus, injecting a racemic PET tracer has not been considered trouble. The tracer administration through the tail-vein in rats and cephalic vein in dogs was followed by a tissue distribution of 30 min p.i. and a whole-body retention and imaging studies 40 min p.i. The initial results in rats showed a rapid total metabolic loss of 41.3% in 60 min p.i. due to decarboxylative loss of [^11^C]CO_2_ through the lungs and urinary excretion. The uptake in rat pancreas was 50 times lower than in dogs, while the ratios of pancreas-to-tissue for almost all tissues doubled in rats compared to dogs. These significant differences may be observed due to a lack of attention to the feeding protocol, which was later shown to be very significant. The imaging studies in dogs indicated no sex difference in tissue distribution, and no evidence of kidney activity was seen in the dog scans. The most important observation was the effect of various feeding protocols on the quality of pancreas scans. The typical arch-shaped dog’s pancreas was easily visible after fasting, followed by protein ingestion before administering the tracer. The *d*,*l*-[*1*-^11^C]valine was described as a potential pancreas imaging agent and highly promoted in clinical studies.^310^

#### 4.21.3 Clinical Studies

**Figure 85:**
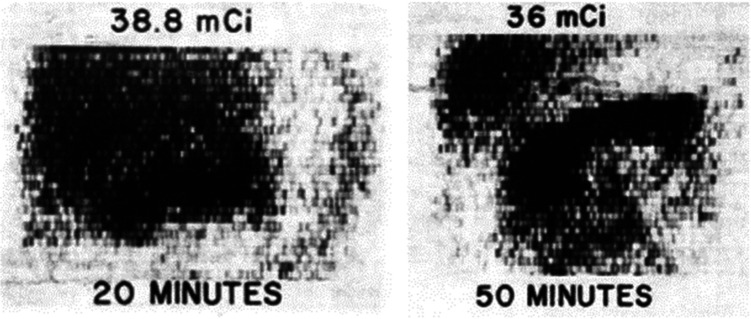
*d*,*l*-[*1*-^11^C]Valine PET scans of a patient recovering from acute pancreatitis (left image) and a patient with mesenteric lymphoma using (right). Reproduced with permission from ref (311). Copyright 1979 Society of Nuclear Medicine\. This work is licensed under a Creative Commons Attribution 4.0 International License (https://creativecommons.org/licenses/by/4.0/).

Two clinical trials in patients have been published since 1978. In the first study, the tracer was administrated in nine patients by iv injections of *d*,*l*-[*1*-^11^C]valine, followed by a highly shielded conventional rectilinear scanning within 5 min p.i. (Figure 85).^311^ The second study was performed on 12 patients with pancreatic disease proven or clinically suspected. The sum of the whole-body retention and urinary excretion indicated a slight loss of activity through decarboxylation, contrasting with results obtained in the preclinical studies. No changes were observed in any hematological parameters or urinalyses, and no toxic or other side effects were observed. Blood clearance and whole-body retention illustrated the rapid plasma clearance of this tracer, where 15 min p.i., the vascular content of the agent had dropped to less than 20% of the administered dose. Also, 14% was present in urine 40 to 60 min p.i. Rectilinear scans in three patients with healthy pancreas showed good pancreatic uptake (Figure 85), high kidney concentration, and a high distinction between pancreas and liver. Therefore, reliable conclusions could not be made because no sufficient model for quantitative analysis existed.^292^

## 5 Enzyme Cofactors and Vitamins

Vitamins and enzyme cofactors are central in many biological processes such as body energy production, metabolism, and intracellular signaling pathways for the normal functioning of the body, and their use has been claimed to prevent neurodegeneration, cancer, and cardiovascular disease. By incorporating carbon-11 in their structure, radiolabeled vitamins could elucidate their absorption, distribution, metabolism, and excretion in a living organism and provide a qualitative and quantitative method through PET imaging.

Enzyme cofactors and vitamins have a unique role *in vivo*, which could potentially be explored using PET imaging. Below, we describe *in vivo* characteristics of compounds that have been labeled with carbon-11 (Table 4).

- *5*,*10*-Methylenetetrahydrofolate (*5*,*10*-Me-THF) is biologically produced *via* methylation of tetrahydrofolate during serine and glycine metabolism.^380^*5*,*10*-Me-THF takes part in the folate cycle, acting as a cofactor of the enzyme thymidylate synthase (TS) that converts deoxyuridine monophosphate to deoxythymidine monophosphate, serving as both methylene and hydride donor.^381^ Due to the overexpression of TS in some tumors, [^11^C]*5*,*10*-Me-THF was considered a tool for cancer diagnosis.^382,383^ Moreover, 5,10-Me-THF interacts with folate receptors, especially folate receptor-α, and might be used to trace the expression of these targets.^383^
- Coenzyme Q_10_ (CoQ_10_) is a co-factor in the respiratory chain of the mitochondrial electron-transfer system and represents an essential endogenous compound in mammals. Furthermore, it plays a significant role in cellular antioxidant defense, switching between the reduced (ubiquinol-10) and oxidized form (ubiquinone-10).^384^
- Acetyl-coenzyme A (acetyl-CoA) is a fundamental endogenous metabolic intermediate that plays a wide variety of biological roles: energy storage thanks to its thioester bond, acetyl carrier for macromolecule biosynthesis, and substrate/product of different catabolic/anabolic pathways. Carnitine has an essential role in the uptake of long-chain FAs into mitochondria and controls the level between free CoA and bound CoA, converting it into acyl-carnitine when it becomes excessive.^385^ Thus, it could be a powerful radiotracer for detecting metabolic disorders.
- *S*-Adenosylmethionine (SAM) is a metabolic product of methionine that is transported into the liver and converted to SAM by methionine-adenosyltransferase (MAT).^386,387^ Therefore, SAM plays a crucial role in diverse cellular processes such as nucleic acid and protein synthesis,^388^ and it serves as the primary donor of methyl groups required in the synthesis of neuronal messengers and membranes.^389^ Found throughout the human body, SAM is highly concentrated in the liver, adrenal glands, pineal gland, and brain.^389^
- *1α*,*25*-Dihydroxyvitamin D_3_ is the physiologically active form of vitamin D. It is obtained in the body after two hydroxylation reactions. The first occurs in the liver and converts vitamin D_3_ to *25*-hydroxyvitamin D_3_, while the second occurs in the kidneys by the action of *25*-hydroxyvitamin D_3_ 1-α-hydroxylase and forms *1α*,*25*-dihydroxyvitamin D_3_. *1α*,*25*-Dihydroxyvitamin D_3_ binds to a specific intracellular receptor^390,391^ and maintains calcium homeostasis, inhibits proliferation, stimulates the differentiation, and induces apoptosis in a wide range of normal and malignant cells.^392,393^
- Biotin is essential for cellular growth, development, and well-being.^394^ It is involved in fatty acid biosynthesis, gluconeogenesis, and catabolism of amino and fatty acids. Biotin is taken up by the cells *via* a sodium-dependent vitamin transporter (SMVT), expressed in the cytoplasm and the mitochondrial membranes of the gastrointestinal tract, liver, kidneys, retina, heart, brain, and skin.^395^
- Alitretinoin is an active metabolite of Vitamin A produced in the pancreas,^396^ liver, kidneys, small intestine, and other tissues,^397^ with a high affinity to retinoid X and retinoic acid receptors.^398^ So far, very little is known about the *in vivo* synthesis pathway.^399^
- Vitamin B_3_ is a family of vitamins that includes niacin, nicotinamide, and nicotinamide riboside found in food and used as a dietary supplement and medication. Niacin is absorbed in the intestine *via* proton- and sodium-coupled monocarboxylate transporters (MCT1 and SMCT, respectively)^400,401^ and is required for the biosynthesis of nicotinamide adenine dinucleotide (NAD) and nicotinamide adenine dinucleotide phosphate (NADP), two coenzymes involved in a variety of redox reactions crucial for cell survival, apoptosis, differentiation, and metabolism of carbohydrates and fats.^402^ It also acts as a lipid-lowering agent interacting with a G protein-coupled receptor (GPR109A), found primarily in the adipose tissue; therefore, it is used to treat hypertriglyceridemia to reduce the progression of atherosclerosis and the risk for cardiovascular. Nicotinamide occurrence in the systemic bloodstream, based primarily on urinary excretion, is higher and more significant than niacin.^403^
- Thiamine is absorbed in the upper small intestine and converted into phosphorylated active forms, which are involved in many cellular processes, such as the metabolism of glucose and amino acids. The uptake of thiamine and its derivatives by cells of the blood and other tissues is mediated by two transporters, hTHTR1 and hTHTR2.^404^
- Ascorbic acid is absorbed in the intestine *via* the sodium-dependent active transporters (SVCT1-2) and in the presence of reactive oxygen species (ROS), it is converted to its oxidative state as dehydroascorbic acid, which is a substrate for glucose transport (GLUT 1, 3, 4). Dysregulation of ROS in several disease states, including cancer, neurodegeneration, chronic inflammation, and diabetes, provides a powerful motivation to develop [*1*-^11^C]ascorbic and [*1*-^11^C]dehydroascorbic acid as noninvasive biomarkers of oxidative stress; indeed, Carrol *et al*. prepared the two tracers and performed PET which has the potential, as a highly sensitive and nontoxic technique, to detect ROS in a preclinical setting.^405^

**Table 4:** Carbon-11 Labeled Enzyme Cofactors and Vitamins

comp		radiolabeling position	preclinical and clinical studies	synthon	*A*_M_ (GBq/μmol)	RCY	total time (min)	ref
*1α,25*-dihydroxyvitamin D_3_		*26*,*27*-	nr^a^	[^11^C]CH_3_I	3	nr	48	(406)
*5*,*10*-methylenetetrahydrofolate		*5*,*10*-*methyl*-	nr	[^11^C]CH_2_O	1.11	95%	2^b^	(407)

acetyl-coenzyme A		*1*-	monkeys^408^	[^11^C]CO_2_	nr	nr	nr	(409)
		*2*-	monkeys^408^	[^11^C]CH_3_I	nr	70%	45	(408)

carnitine		*O*-*1*-*acetyl*- and *O*-*2*-*acetyl*-	monkeys,^408^ humans^410^	[^11^C]CO_2_	nr	70%	45	(408)
		*N*-*methyl*-	nr	[^11^C]CH_3_I	nr	80%	40	(408)

acetyl-*l*-carnitine		*N*-*methyl*-	monkeys^408^	[^11^C]CH_3_I	nr	60%	30	(408)
biotin		*2*′-	mice^411^	[^11^C]CO_2_	7	19%	32	(411)
coenzyme Q_10_	ubiquinone-10	*5*-*methoxy*-	mongolian gerbils,^412,413^ rats,^261,414^ dogs^415^	[^11^C]CH_3_I	5.39	nr	33	(416)
	ubiquinol	*39*-*methyl-*	rats^417^	[^11^C]CH_3_I	76	39%	38	(418)
vitamin A		*5*-*methyl*-	nr	[^11^C]CH_3_I	nr	25%	32	(419)

vitamin B_3_	niacin	*carbonyl*-	mice,^420^ humans^421^	[^11^C]CO	750	65%	27	(422)
				[^11^C]CO_2_	7	17%	25	(420)
	nicotinamide	*carbonyl*-	monkeys^423^	[^11^C]CO	1600	54%	b	(422,424)
				[^11^C]HCN	74	45%	30	(423)

*S*-adenosylmethionine		*S*-*methyl*-	mice,^425,426^ rats,^426,427^ rabbits^428^	[^11^C]CH_3_I	7.326	80%	45	(428)
				[^11^C]CH_3_OTf	1363	17%	28	(387,425)

thiamine		*4*-*methyl*-	rats^429^	[^11^C]CH_3_I	nr	nr	60	(430)

vitamin C		*carbonyl*-	mice,^431^ rats^405^	[^11^C]HCN	15.27	18.1%	35	(431)

a nr: Not reported.

b Time of synthesis.

### 5.1 *1α*,*25*-Dihydroxyvitamin D_3_

#### 5.1.1 Radiosynthesis

**Figure 86:**
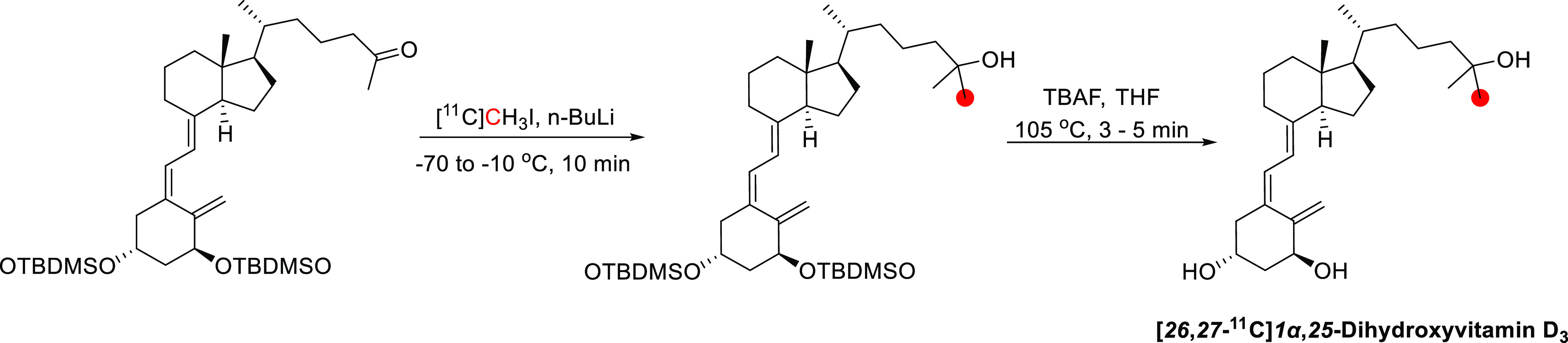
Synthesis of [*26*,*27*-^11^C]*1α*,*25*-dihydroxyvitamin D_3_ using [^11^C]CH_3_I. ^11^C radionuclide position is highlighted in red.

[*26*,*27*-^11^C]*1α*,*25*-Dihydroxyvitamin D_3_ was synthesized *via* a two-step [^11^C]methylation reaction. In the first step, [^11^C]CH_3_I (obtained from [^11^C]CO_2_, lithium aluminum hydride, and HI) was trapped at −70 °C in a reaction vial pre-charged with a methyl ketone precursor (*1*(*S*),*3*(*R*)-*bis*[(*tert*-butyldimethylsilyl)oxy]-*25*-*keto*-*9*,*10*-*seco*-*27*-norcholesta-*5*(*Z*),*7*(*E*),*10*(*19*)-triene) dissolved in THF; after the addition of butyllithium, the vial was kept at −10 °C for 10 min, providing the ^11^C intermediate with the positron-emitting radionuclide incorporated at the *26*,*27* carbons in about 30–50% RCY. In the second step, the reaction was quenched with tetrabutylammonium fluoride, and the vial was heated at 105 °C for 5 min to allow the cleavage of the two *tert*-butyldimethylsilyl (TBDMS) protecting groups of the ^11^C intermediate (Figure 86). At the end of synthesis and semipreparative HPLC purification, [*26*,*27*-^11^C]*1α*,*25*-dihydroxyvitamin D_3_ produced with an *A*_m_ of 2.5–3 GBq/μmol. Based on the HPLC analysis of the final eluent fraction, the RCP was >99%, and the chemical purity was about 79%. The entire process required 48 min from the end of the bombardment, providing the desired compound in a time frame compatible with the short half-life of the carbon-11 radionuclide and suitable for developing PET studies in animals and humans.^406^ However, the compound has not been evaluated to the best of our knowledge.

### 5.2 *5*,*10*-Methylenetetrahydrofolate

#### 5.2.1 Radiosynthesis

**Figure 87:**
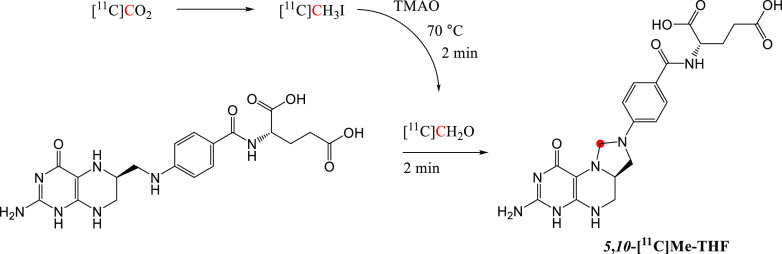
Synthesis of *5*,*10*-[^11^C]Me-THF. ^11^C radionuclide position is highlighted in red.

Radiolabeling of *5*,*10*-Me-THF with ^11^C on the methylene position was accomplished using [^11^C]CH_2_O as a radioactive synthon (Figure 87).^407^ [^11^C]CH_3_I was initially treated with trimethylamine oxide for 2 min, yielding [^11^C]CH_2_O (RCY of 80%), which then reacted with tetrahydrofolate yielding the desired *5*,*10*-[^11^C]Me-THF with a nonisolated RCY of 95% (calculated from the radio HPLC chromatogram) within 2 min from [^11^C]CH_2_O delivery and *A*_m_ of 0.37-1.11 GBq/μmol.^407^ Despite the good RCY, the compound has not been evaluated to the best of our knowledge.

### 5.3 Acetyl-coenzyme A, Carnitine, and Acetyl-*l*-carnitine

#### 5.3.1 Radiosynthesis

**Figure 88:**
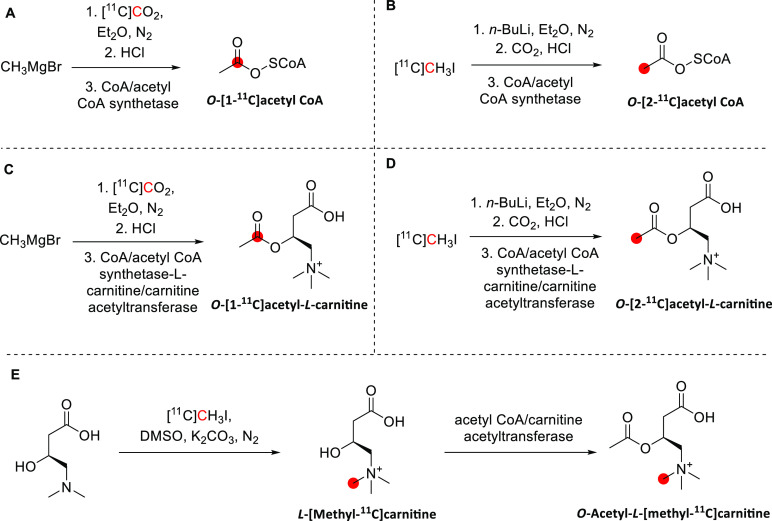
Radiosynthetic schemes of [^11^C]acetyl CoA, [^11^C]acetyl-*l*-carnitine, and *l*-[^11^C]carnitine. ^11^C radionuclide position is highlighted in red.

*O*-[*1*-^11^C]Acetyl CoA (Figure 5) was prepared from incubation of [*1*-^11^C]acetic acid, obtained according to a previously reported procedure,^409^ with a solution of coenzyme A, enzyme acetyl CoA synthetase, adenosine triphosphate (ATP), and magnesium chloride at pH 8. After 5 min at 37 °C, proteins were denatured with HCl and purified with preparative HPLC.^408^*O*-[*2*-^11^C]Acetyl CoA was prepared from incubation of [*1*-^11^C]acetic acid, obtained according to a previously reported procedure,^409^ with a solution of coenzyme A, enzyme acetyl CoA synthetase, ATP, and magnesium chloride at pH 8. After 5 min at 37 °C, proteins were denatured with HCl and purified with preparative HPLC.^408^*O*-[*2*-^11^C]Acetyl CoA (Figure 88) was prepared following the same procedure of *O*-[*1*-^11^C]acetyl CoA starting from [*2*-^11^C]acetic acid, obtained according to a previously reported procedure.^432^ Both acetyl CoA radiotracers were obtained with 60–70% RCY with respect to [^11^C]acetate within 45 min from EOB (in a typical run starting with 4.1 GBq of [^11^C]acetate, 1.6 GBq of products were obtained)^408^ was prepared following the same procedure of *O*-[*1*-^11^C]acetyl CoA starting from [*2*-^11^C]acetic acid, obtained according to a previously reported procedure.^432^ Both acetyl CoA radiotracers were obtained with 60–70% RCY with respect to [^11^C]acetate within 45 min from EOB (in a typical run starting with 4.1 GBq of [^11^C]acetate, and 1.6 GBq of products were obtained.^408^

*O*-[*1*-^11^C]Acetyl-l-carnitine and *O*-[*2*-^11^C]acetyl-l-carnitine (Figure 88) were obtained following the same procedure above reported for acetyl CoA radiotracers with the addition of a solution of *l*-carnitine and enzyme carnitine acyltransferasein 5 min incubation. Both acetylcarnitine radiotracers were obtained with 70–80% RCY with respect to [^11^C]acetate within 45 min from EOB.^408,408^

*l*-[*Methyl*-^11^C]carnitine (Figure 88) was prepared from the reaction between [^11^C]CH_3_I and *N*-desmethyl-l-carnitine with potassium carbonate as the base in dimethyl sulfoxide. After 5 min at 90 °C, the reaction was quenched with water, and the product was purified through cation exchange resin, obtaining *l*-[*methyl*-^11^C]carnitine with 60% RCY concerning [^11^C]CH_3_I within 30 min from EOB. *O*-Acetyl-*l*-[*methyl*-^11^C]carnitine (Figure 88) was obtained by reacting *l*-[*methyl*-^11^C]carnitine with acetyl CoA and carnitine acyltransferase in Tris buffer, obtaining *O*-acetyl-*l*-[*methyl*-^11^C]carnitine with 70-80% RCY based on *l*-[*methyl*-^11^C]carnitine within 40 min from EOB.^408,408^

#### 5.3.2 Preclinical Studies

Preliminary PET studies in monkeys were conducted to evaluate the *in vivo* behavior of *O*-[^11^C]acetyl CoA, *O*-[^11^C]acetyl-l-carnitine, and *l*-[^11^C]carnitine.^408^ Plasma clearance in all cases was fast, decreasing immediately after iv injection. Only ^11^C-labeled acetylcarnitine seemed to have slightly lower clearance rates. *O*-[*1*-^11^C]acetyl CoA had high initial uptake in the myocardium (myocardium-plasma ratio around 4–10 min after injection), followed by a fast washout. ^11^C-Radiolabeled carnitines showed increased uptake in myocardium over 60 min. Renal uptake was the highest, with renal/plasma ratio of around 20. Renal excretion of *1*- and *2*-[^11^C]acetyl CoA radiotracers were faster than carnitine-labeled radiotracers because they probably undergo different metabolic pathways. In the liver, higher uptake was registered for ^11^C carnitine-bearing derivatives, despite being lower than in the kidneys.^408^ In the brain, an immediate uptake was registered for all tracers with a fast washout. The uptakes of carnitine-labeled tracers were different, depending on the position of the ^11^C-label, with *O*-[*1*-^11^C]acetyl-*l*-carnitine showing the highest uptake after 60 min in the cerebral cortex.^433^ The significant differences between acetylcarnitine radiotracers (*O*-[*1*-^11^C]acetyl-*l*-carnitine, *O*-[*2*-^11^C]acetyl-*l*-carnitine, and *O*-acetyl-*l*-[*methyl*-^11^C]carnitine) brain uptake suggests that they are rapidly metabolized into different radiolabeled species, reflecting the different labeling positions.^433,434^

#### 5.3.3 Clinical Studies

**Figure 89:**
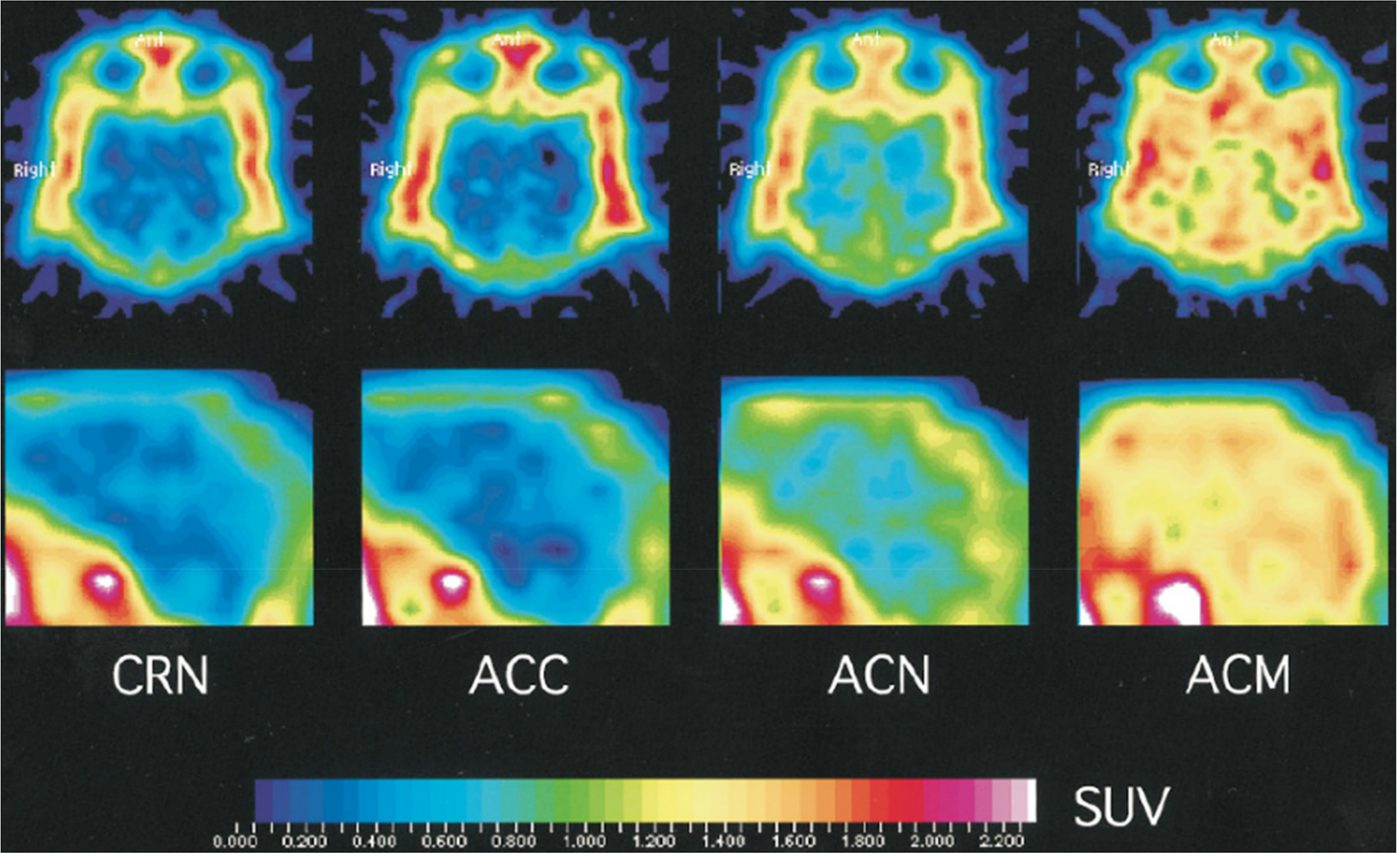
[*2*-^11^C]Acetyl-*l*-carnitine (ACM), [*1*-^11^C]acetyl-*l*-carnitine (ACN), acetyl-*l*-[*methyl*-^11^C]carnitine (ACC), and *l*-[*methyl*-^11^C]carnitine (CRN) distribution in a horizontal section (top) and sagittal section (bottom) of rhesus monkeys brain (30–45 min p.i.). Reproduced with permission from ref (433). Copyright 1997 Elsevier.

After iv injection, *O*-[*1*-^11^C]acetyl-l-carnitine registered high brain uptake in healthy volunteers.^410^*O*-[*2*-^11^C]Acetyl-l-carnitine was used to detect chronic fatigue syndrome (CFS), a pathological condition where the value of free carnitine remains normal, while an apparent decrease of acyl-carnitine combined with fatty acid was displayed.^410^ Patients suffering from CFS registered a lower uptake in the brain, particularly in the visual cortex, and a lower erythrocyte-to-blood plasma ratio than healthy volunteers (Figure 89).^410^

### 5.4 Biotin

#### 5.4.1 Radiosynthesis

**Figure 90:**
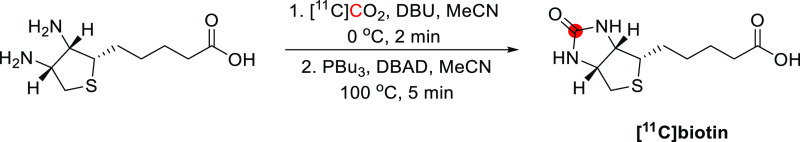
Synthesis of [^11^C]biotin using [^11^C]CO_2_. ^11^C radionuclide position is highlighted in red.

[^11^C]Biotin was synthesized by applying a rapid and efficient ^11^C-urea labeling method *via* a simple one-pot reaction in a fully automated system.^435^ In the first step, cyclotron-produced [^11^C]CO_2_ was bubbled into a reaction vial containing diamino biotin as a precursor and *1*,*8*-diazabicyclo[*5*.*4*.*0*]undec-*7*-ene (DBU) dissolved in acetonitrile (MeCN) at 0 °C.^435^ At the end of the [^11^C]CO_2_ delivery, a solution of Mitsunobu reagents [di-*tert*-butyl azodicarboxylate (DBAD) and tributylphosphine (PBu_3_)] was added to the reaction vial, and the reaction mixture was heated at 100 °C for 5 min. (Figure 90) The reaction was subsequently cooled to room temperature and quenched with a phosphate-buffered saline (PBS) solution. [^11^C]Biotin was purified by semipreparative HPLC, and the formulated solution was used for preclinical studies. [^11^C]Biotin was obtained after 32 min (total synthesis time from the end of delivery (EOD) including HPLC purification) with an isolated RCY of 19 ± 2%, a RCP > 99%, and *A*_m_ of 7 ± 1 GBq/μmol. The final amount of [^11^C]biotin was 352 ± 38 MBq in 4–5 mL PBS with 2.5% ethanol.^411^

#### 5.4.2 Preclinical Studies

**Figure 91:**
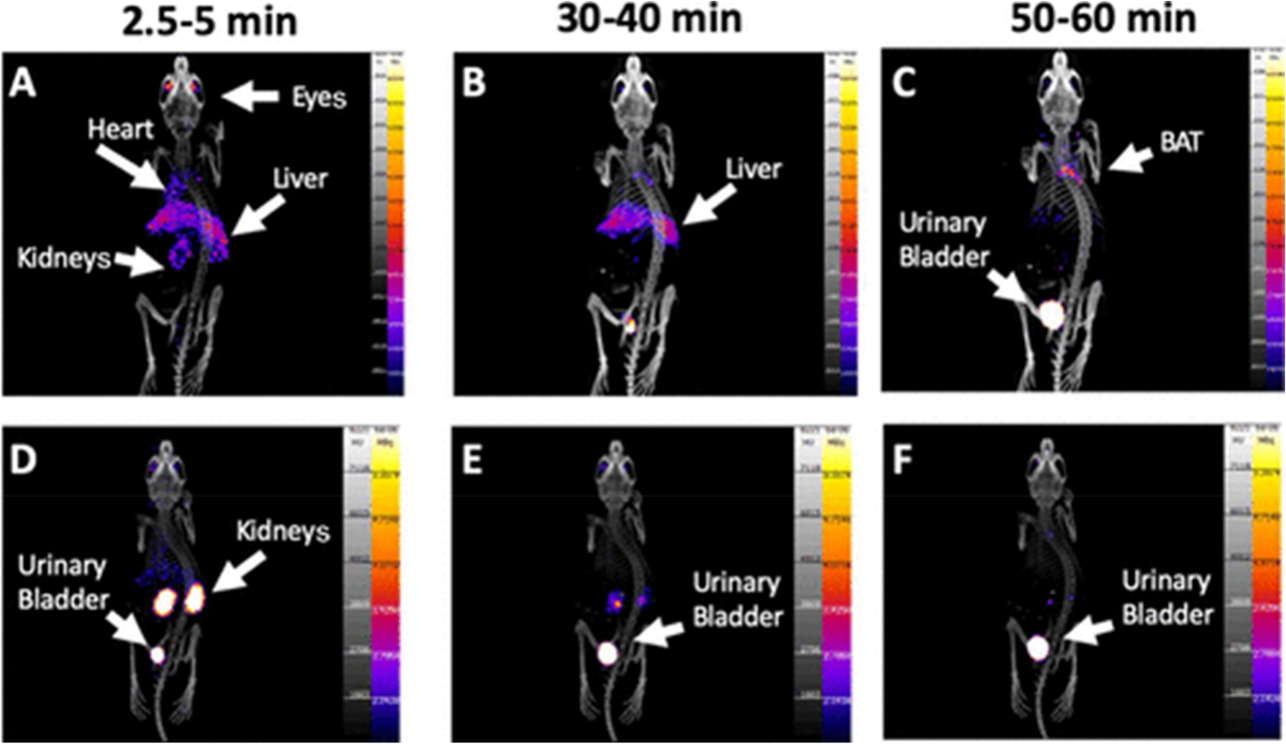
[^11^C]Biotin PET scans of non-biotin added (A–C) and biotin-challenged (D–F) mice show displacement of the tracer. Reproduced with permission from ref (411). Copyright 2020 American Chemical Society.

**Figure 92:**
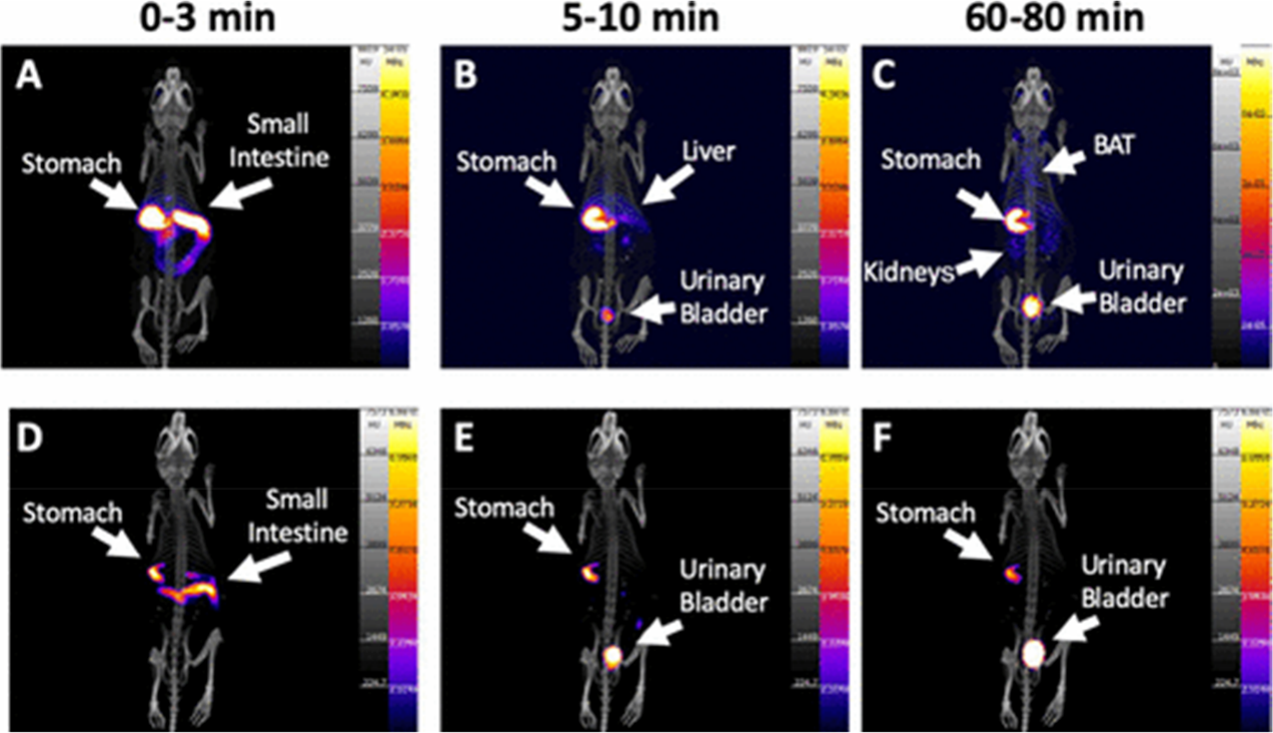
[^11^C]Biotin PET scans after oral administration in non-biotin added (A–C) and biotin-challenged (D–F) mice. Reproduced with permission from ref (411). Copyright 2020 American Chemical Society.

To examine the [^11^C]biotin trafficking *in vivo*, [^11^C]biotin was administered iv in healthy anesthetized mice placed on a high-resolution micro-PET scanner, and the dynamic PET image data were acquired for 60 min.^411^ PET imaging demonstrated the [^11^C]biotin distribution in the liver, heart, brain, eyes, and kidneys, consistent with the known expression of the biotin transporter SMVT in these organs. Surprisingly, accumulation in the interscapular brown adipose tissue (BAT) was also detected (Figures 91 and 92). Furthermore, to investigate the gastrointestinal uptake of biotin and its body circulation *in vivo*, [^11^C]biotin was orally administered in isoflurane-anesthetized mice, and PET imaging studies were performed for 120 min. Once delivered into the intestine, [^11^C]biotin was rapidly absorbed in the duodenum and, entering the systemic circulation was distributed throughout the body in the liver, heart, eyes, brain, and interscapular BAT. The organ distribution of [^11^C]biotin administered orally is similar to that observed after iv administration. Preadministration of nonradioactive biotin decreased [^11^C]biotin uptake in all SMVT-expressing organs and increased its elimination through the kidneys to the urinary bladder, suggesting SMVT saturation by the administered biotin.^411^

### 5.5 Coenzyme Q_10_ (Ubiquinone-10)

#### 5.5.1 Radiosynthesis

**Figure 93:**
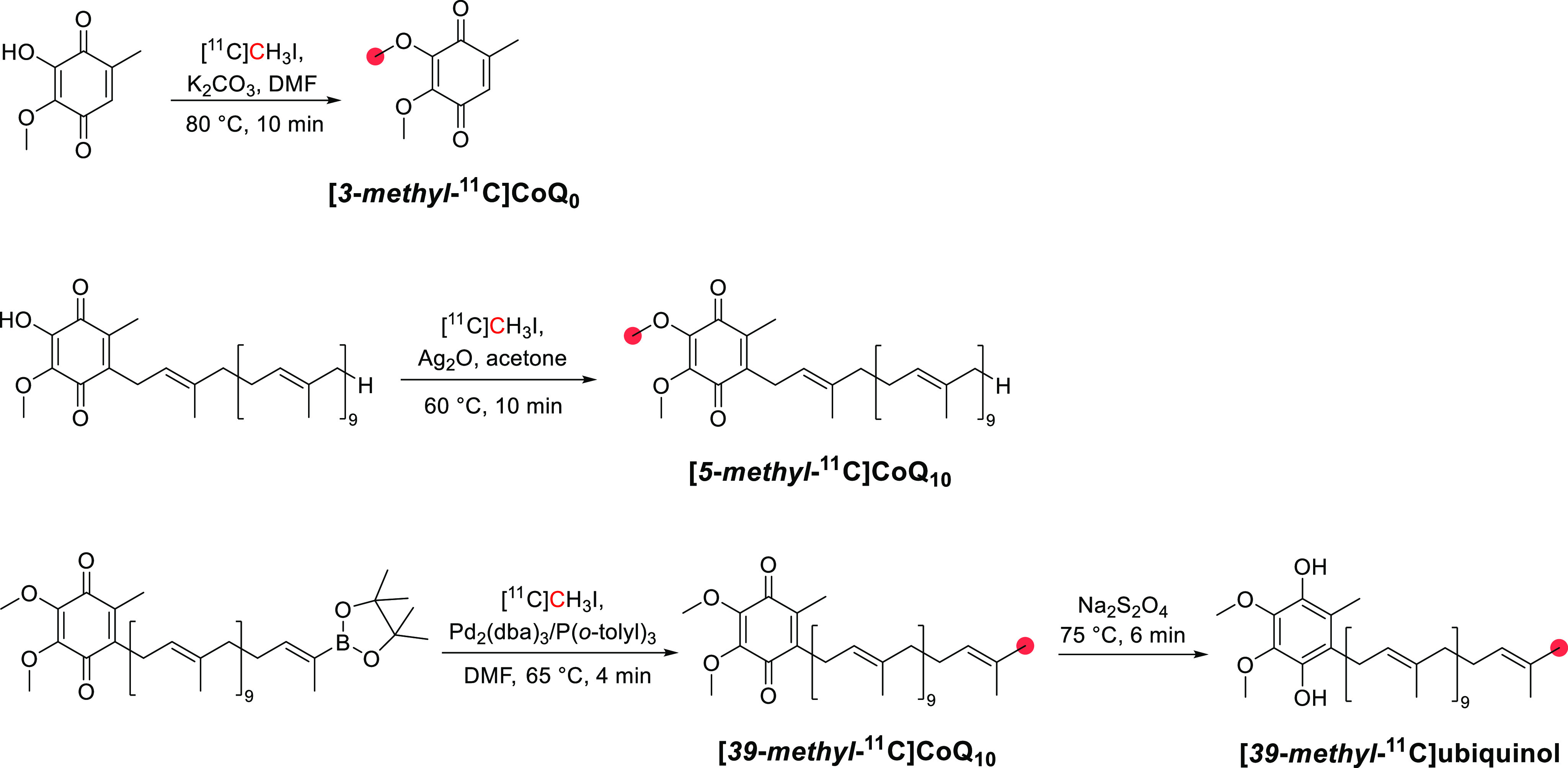
Radiosynthetic schemes of [*3*-*methyl*-^11^C]CoQ_0_, [*5*-*methyl*-^11^C]CoQ_10_, [*39*-*methyl*-^11^C]CoQ_10_, and [*39*-*methyl*-^11^C]ubiquinol. ^11^C radionuclide position is highlighted in red.

The first preparation of [^11^C]CoQ_10_ was reported in 1982 by Takahashi *et al*. [*3*-Methyl-^11^C]CoQ_10_ was obtained from [^11^C]methylation of *3*-demethylCoQ_10_ in acetone at 50–60 °C for 10 min, purification through chromatographic separation on the silica-gel column, and final reformulation (Figure 93). It was prepared in 40–50 min from bubbling of [^11^C]CH_3_I with RCY of 15.7% (achieving 880.6 MBq from 5.56 GBq of [^11^C]CH_3_I), RCP >99%, and *A*_m_ of 0.148–0.185 GBq/μmol.^412,413^ Final reformulations of [*5*-*methyl*-^11^C]CoQ_10_ have been reported in two different ways, depending on the emulsifying agent exploited: in saline with polyoxyethylene hydrogenated castor oil ([^11^C]CoQ_10_-HCO-60) or ethanol within liposomes ([^11^C]CoQ_10_-liposomes).^413,414^ Similarly, a CoQ_10_ derivative without a side chain, [^11^C]CoQ_10_, has been labeled with [^11^C]CH_3_I, desmethyl precursor, and potassium carbonate in DMF for 10 min at 80 °C. It was obtained within 33 min in 38.9 ± 10% RCY with 5.39 ± 1.73 GBq/μmol as *A*_m_.^416^ More recently, [^11^C]CoQ_10_ has been labeled at the terminal position of the polyprenylated side chain of ubiquinone. [e9-*Methyl*-^11^C]CoQ_10_ has obtained from Pd-mediated [^11^C]methylation of 39-demethyl-39-(pinacolboryl)ubiquinone in DMF at 65 °C for 4 min, purified in reverse-phase semipreparative HPLC and final reformulation in saline, glycol, and Tween80. It was prepared in 36 min with 53% RCY, achieving 0.4–3.5 GBq with *A*_m_ of 21–78 GBq/μmol. [39-*Methyl*-^11^C]CoQ_10_ prepared as above was then used to achieve [^11^C]ubiquinol through reduction with sodium dithionite at 75 °C for 6 min. Particularly, [^11^C]ubiquinol was obtained in 38 min of radiosynthesis with 39% RCY and 95% RCP but 77% chemical purity, achieving 0.16–1.4 GBq of product with *A*_m_ of 48–76 GBq/μmol. Noteworthy, in this case, the final reformulation included ascorbic acid to partially avoid [^11^C]ubiquinol degradation.^418^

#### 5.5.2 Preclinical Studies

**Figure 94:**
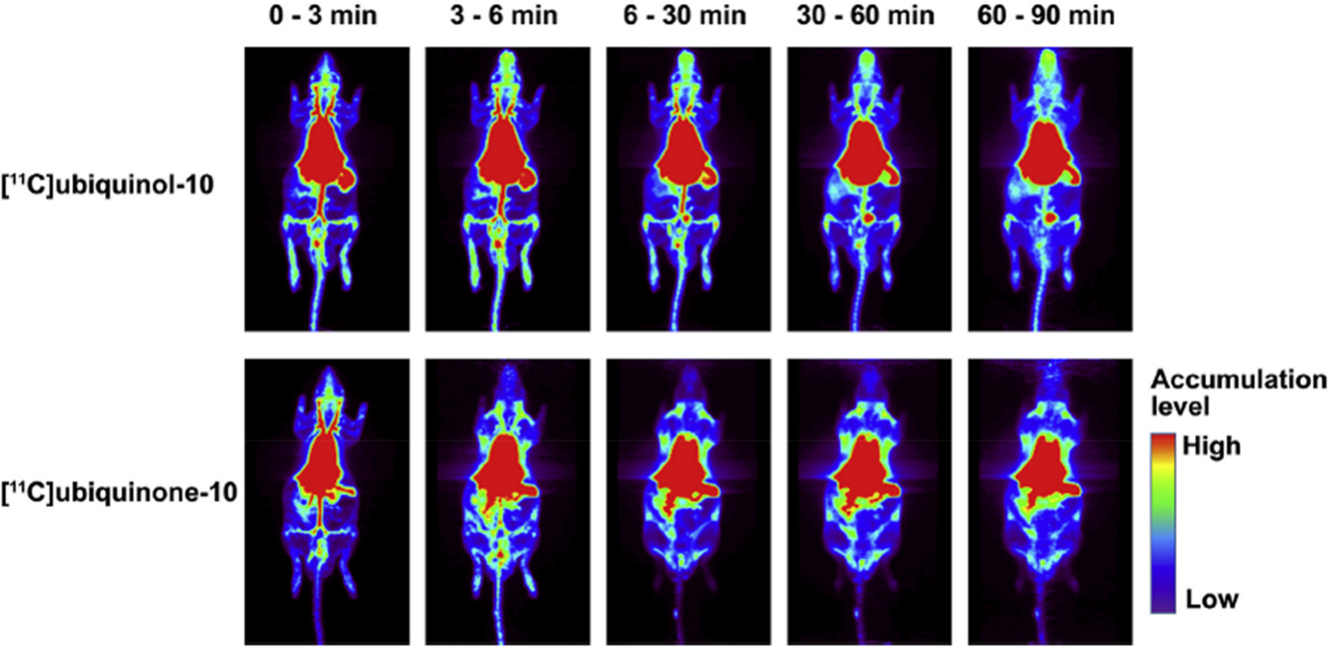
[*39*-*Methyl*-^11^C]CoQ_10_ and [*39*-*methyl*-^11^C]ubiquinol pharmacokinetics in ddY mice. Reproduced with permission from ref (417). Copyright 2019 Elsevier.

[^11^C]CoQ_10_-HCO-60 injected in Mongolian gerbils through a lateral tail vein revealed high and prolonged retention of activity in the blood, followed by lung, spleen, gall bladder, and kidney, accounting for its excretion through urine and feces. The uptake in the brain was low but increased with time.^412,413^ In pregnant Wistar rats (16–19^th^ day of gestation) [^11^C]CoQ_10_-HCO-60 accumulated mainly in maternal than fetal organs, albeit a fetus-to-placenta ratio increasing with time, with relatively high uptake in fetal brain than the maternal brain (fetal/maternal brain uptake ratio at 30 min around 153).^261^ Subsequently, [^11^C]CoQ_10_-HCO-60 and [^11^C]CoQ_10_-liposomes biodistributions were compared in adult and newborn Wistar rats (injected in dorsal veins and intraperitoneally, respectively).^414^ In this case, [^11^C]CoQ_10_-liposomes were rapidly cleared from the blood and quickly incorporated into the spleen and liver. The heart-to-blood ratio significantly increased in liposome formulation in adult and newborn rats. Both [^11^C]CoQ_10_-HCO-60 and [^11^C]CoQ_10_-liposomes demonstrated very low uptake in the brain.^414^ Based on these premises, [^11^C]CoQ_10_-liposomes were further evaluated as a myocardial imaging tracer in dogs injected through the femoral vein.^415^ In contrast to rats, the heart-to-blood ratio was only 0.5 after 30 min (*vs* 10 in rats), preventing acceptable myocardial imaging. Furthermore, increased heart uptake of [^11^C]CoQ_10_-liposomes, corrected for blood spillover of radioactivity, was registered, demonstrating incorporation of exogenous [^11^C]CoQ_10_ in normal myocardium over a short period (45 min).^415^ More recently, biodistributions of the oxidized and reduced form of [^11^C]CoQ_10_ (ubiquinone-10 and ubiquinol-10 labeled at 39 positions) were evaluated in Sprague-Dawley rats.^417^ As shown in Figure 94, radiotracers mainly accumulated in the liver, lung, and spleen combined with an enhanced and persistent uptake in the heart, aortas, and head region for [^11^C]ubiquinol. Similar liver uptakes were registered, while cerebellum, cerebrum, and adipose tissue provided a significant major accumulation of activity for [^11^C]ubiquinol at 90 min p.i. On the contrary, [^11^C]CoQ_10_ showed a more remarkable and significant uptake in the spleen.^417^ In male ddY mice, [^11^C]CoQ_10_ confirmed similar high abdomen accumulation in the kidney, lung, liver, and heart. [^11^C]CoQ_0_ (congener substrate of complex I without isoprenoid side chain) revealed an increasing uptake in the brain until 5 min p.i., which slowly decreased over time even if background bloodborne activity remains too high for brain imaging.^416^

### 5.6 Vitamin A

#### 5.6.1 Radiosynthesis

**Figure 95:**
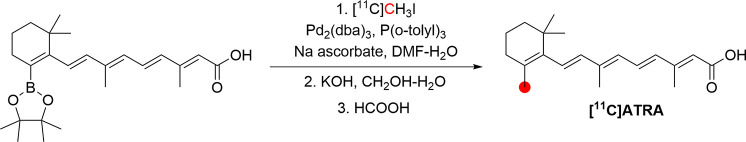
Radiosynthesis of [^11^C]ATRA using [^11^C]CH_3_I. ^11^C radionuclide position is highlighted in red.

[^11^C]All-*trans*-retinoic acid (ATRA) was reported for the first time in 2014, using a combination of rapid Pd(0)-mediated C-[^11^C]methylation of an alkenyl boron precursor, which was produced *via* an eight-step method. The Pd(0)-mediated [^11^C]methylation was performed using [^11^C]CH_3_I in the presence of Pd_2_(dba)_3_, P(*o*-tolyl)_3_, K_2_CO_3_ (1:4:9) in DMF at 65 °C for 4 min, followed by basic hydrolysis of the ethyl ester at 100 °C for 2 min to synthesize [^11^C]ATRA in 14% yield (HPLC analytical yield). Sodium ascorbate was added to prevent radiolysis, and rapid [^11^C]methylation in the presence of sodium ascorbate led to [^11^C]ATRA formation. The RCY was 25% based on [^11^C]CH_3_I, and RCP >99% with a total synthesis time, including HPLC purification and formulation, of 32 min. *9*-*cis*-[^11^C]Retinoic acid was also one of the products of this reaction (Figure 95).^419^

### 5.7 Vitamin B_3_

#### 5.7.1 Niacin

##### 5.7.1.1 Radiosynthesis

[*Carboxyl*-^11^C]niacin has been synthesized by four different methods. Machulla *et al*. in 1979 prepared [*carboxyl*-^11^C]niacin by reaction of [^11^C]CO_2_ with *3*-pyridyl-lithium in ether, followed by hydrolysis and addition of HCl. However, the authors did not mention any details about the RCY, RCP, or *A*_m_.^436,436^

Karimi *et al*. introduced another method using [^11^C]CO.^422^ Briefly, in a micro-autoclave at high pressure, pre-charged with [^11^C]CO, a mixture of tetrakis(triphenyl-phosphine)palladium(0), *3*-iodopyridine, and tetra-methylammonium hydroxide in dry THF was added. The micro-autoclave was heated at 180 °C for 5 min and purified by semipreparative liquid chromatography. [*Carboxyl*-^11^C]niacin was isolated within 27 min, with a RCY of 65 ± 3% and *A*_m_ of 750 ± 30 GBq/μmol after EOB.^422^

**Figure 96:**
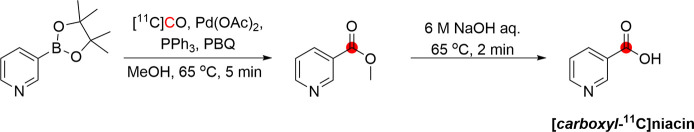
Synthesis of [*carboxyl*-^11^C]niacin using [^11^C]CO. ^11^C radionuclide position is highlighted in red.

Ishii *et al.*,^424^ in 2015, developed a new method for the Pd(0)-mediated [^11^C]carbonylation of 3-pyridine boronic acid pinacol ester with [^11^C]CO in the presence of *p*-benzoquinone and triphenylphosphine in a mixture of DMF and MeOH under ambient pressure at 65 °C. Ishii *et al.*,^424^ in 2015, developed a new method for the Pd(0)-mediated [^11^C]carbonylation of *3*-pyridine boronic acid pinacol ester with [^11^C]CO in the presence of *p*-benzoquinone and triphenylphosphine in a mixture of DMF and MeOH under ambient pressure at 65 °C (Figure 96). This method converted the boronate to the corresponding methyl ester of [*carboxyl*-^11^C]niacin. The addition of NaOH led to the formation of [*carboxyl*-^11^C]niacin with a RCY of 76 ± 14%.^424^ This method converted the boronate to the corresponding methyl ester of [*carboxyl*-^11^C]niacin. The addition of NaOH led to the formation of [*carboxyl*-^11^C]niacin with a RCY of 76 ± 14%.^424^

**Figure 97:**
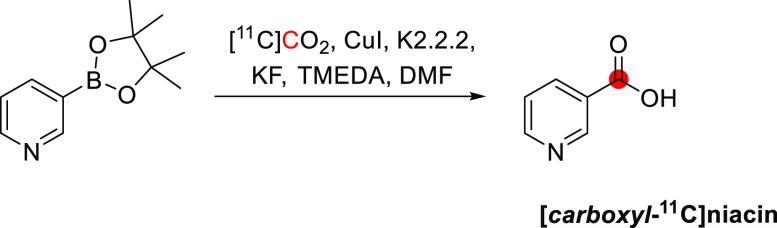
Synthesis of [*carboxyl*-^11^C]niacin using [^11^C]CO_2_. ^11^C radionuclide position is highlighted in red.

In 2020, Bongarzone *et al.* synthesized [*carboxyl*-^11^C]niacin *via* a simple, rapid, one-step Cu-mediated ^11^C-carboxylation reaction in a fully automated system using [^11^C]CO_2_, *3*-pyridine boronic acid pinacol ester precursor (Figure 97).^420^ The 10 min reaction at 110 °C used TMEDA as a base/ligand, CuI as a catalyst, and KF/K2.2.2 as a fluoride ion source in DMF. The product was obtained with a RCY of 17 ± 2%, RCP >99%, and *A*_m_ of 7 ± 1 GBq/μmol at EOD. Total synthesis time, including HPLC purification, was 25 ± 1 min.

##### 5.7.1.2 Preclinical Studies

**Figure 98:**
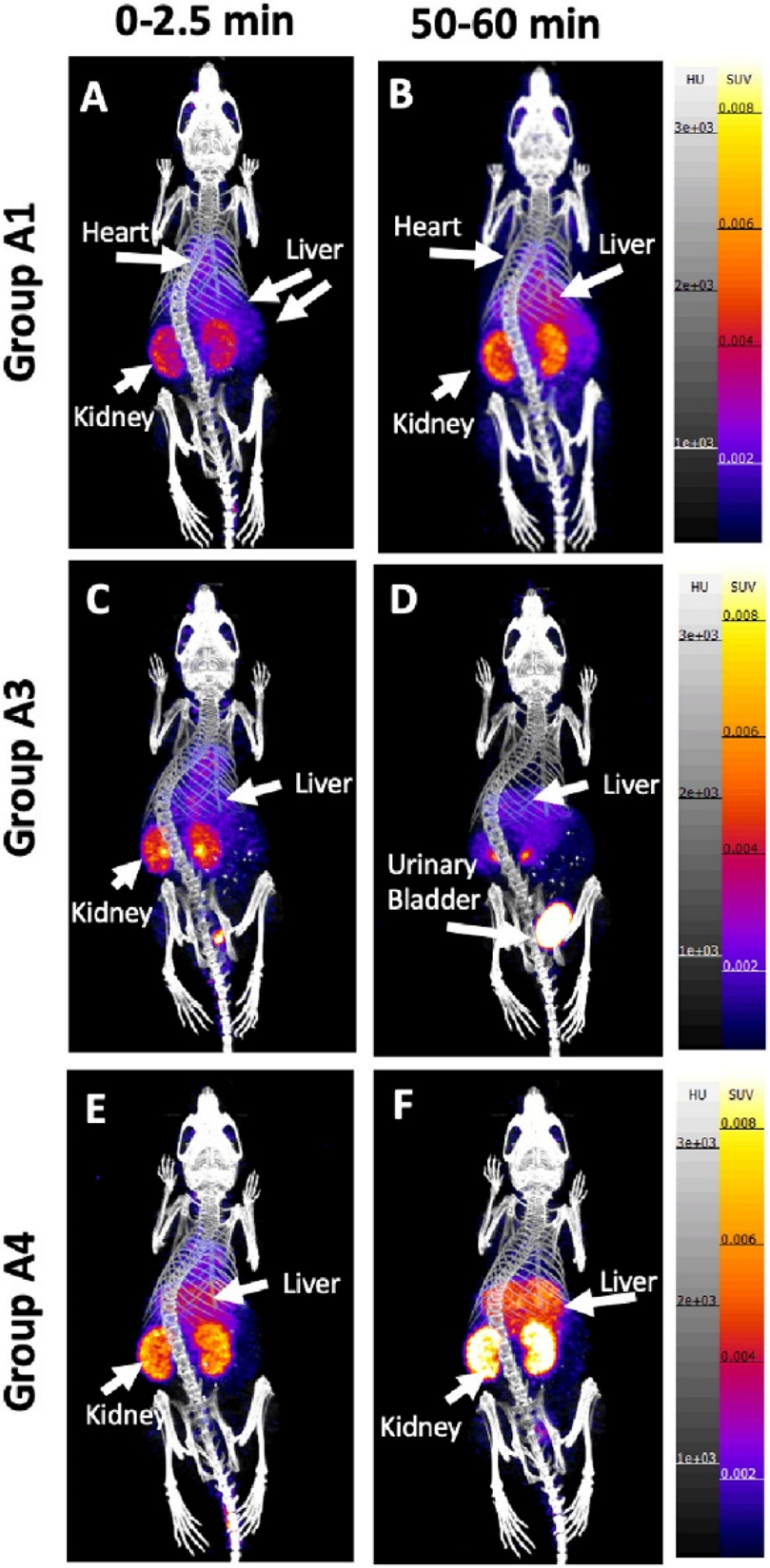
[*Carboxyl*-^11^C]niacin PET images of no-niacin added (A,B), niacin-challenged mice (C,D) AZD3965-challenged (E,F) mice. Reproduced with permission from ref (420). Copyright 2020 Elsevier. This work is licensed under a Creative Commons Attribution 4.0 International License (https://creativecommons.org/licenses/by/4.0/).

**Figure 99:**
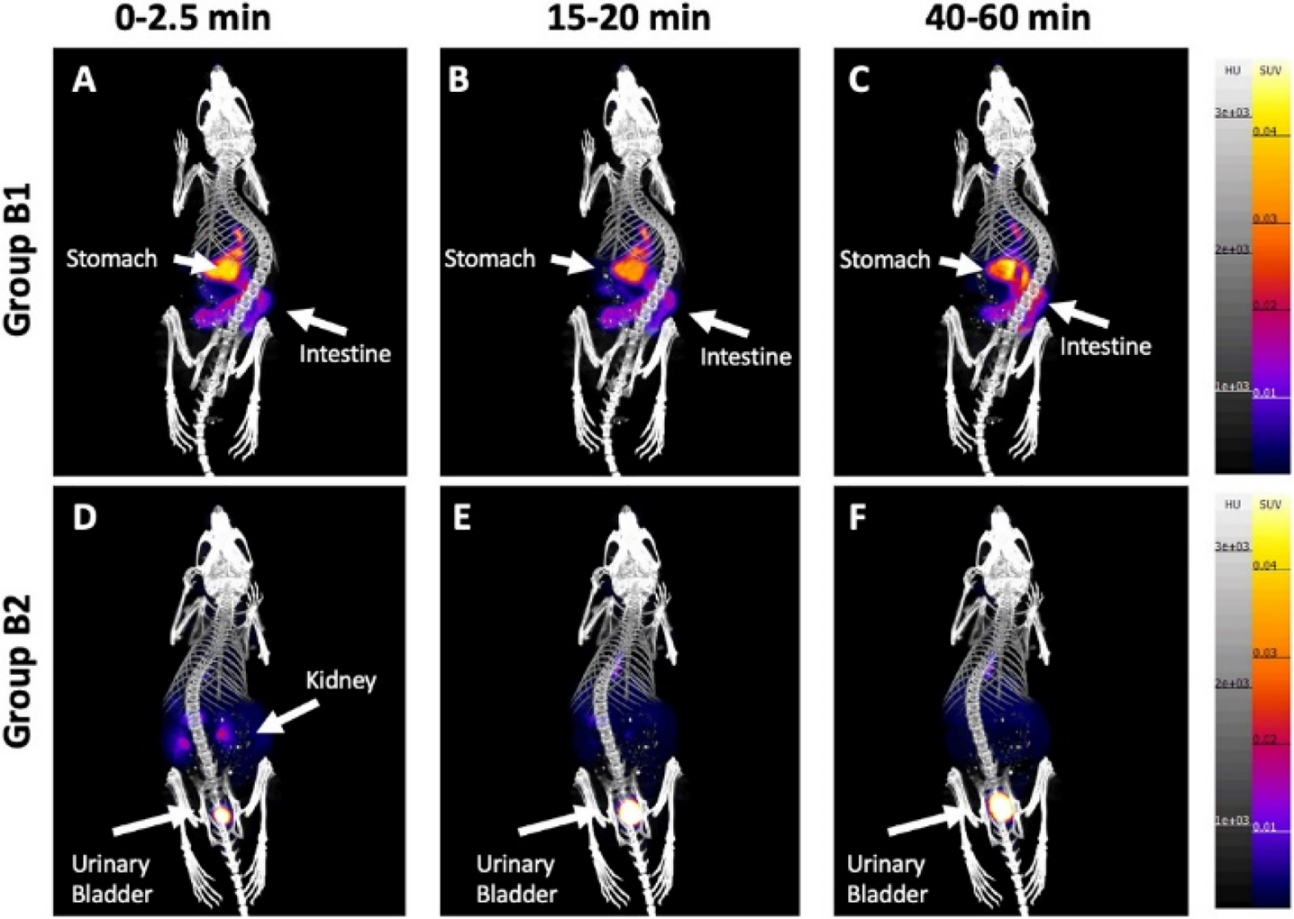
[*Carboxyl*-^11^C]niacin PET images after oral administration in no-niacin added (A–C) and niacin-challenged (D–F) mice. Reproduced with permission from Bongarzone ref (420). Copyright 2020 Elsevier. This work is licensed under a Creative Commons Attribution 4.0 International License (https://creativecommons.org/licenses/by/4.0/).

[*Carboxyl*-^11^C]niacin was administered intravenously in healthy mice, and dynamic PET data were acquired for 60 min (Figures 98 and 99).^420^ [*Carboxyl*-^11^C]niacin accumulated in the kidney, liver, retina, and heart, where SMCTs and MCT1 transporters are primarily expressed. Pre-administration of nonradioactive niacin or a potent MCT1 inhibitor (AZD3965) increased urinary excretion and decreased the uptake in MCT1-expressing organs of [*Carboxyl*-^11^C]niacin was administered orally in mice, and emission data were acquired for 120 min (Figures 98 and 99). No-carrier added [*carboxyl*-^11^C]niacin accumulated in the intestine. In addition, the carrier added [*carboxyl*-^11^C]niacin resulted in a preferential distribution to the excretory organs and other tissues expressing niacin transporters.

##### 5.7.1.3 Clinical Studies

[*Carboxyl*-^11^C]niacin has been evaluated in one case study of a 67-year-old-male who had no known neurological disorders to study the permeability of the BBB.^421^ Data of the activity concentration in the blood and tissues were collected using PET immediately after the iv injection (*A*_m_ of 0.066 GBq/μmol). PET images showed that the brain signal was low compared to the signal from CNS blood vessels, leading to the conclusion that the tracer did not pass the BBB.^421^

#### 5.7.2 Nicotinamide

##### 5.7.2.1 Radiosynthesis

**Figure 100:**
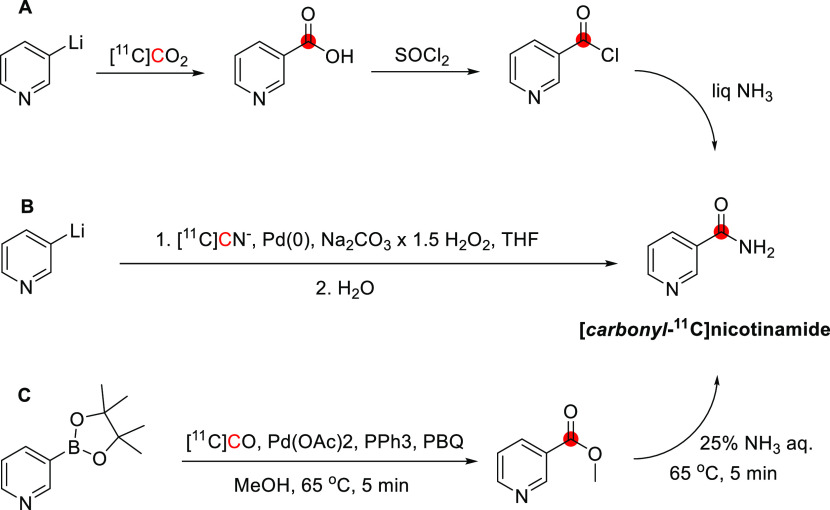
Synthesis of [*carbonyl*-^11^C]nicotinamide using (A) [^11^C]CO_2_, (B) [^11^C]CO, and (C) [^11^C]CN^–^. ^11^C radionuclide position is highlighted in red.

[*Carbonyl*-^11^C]nicotinamide has been synthesized by four different methods. First, Machulla *et al*. prepared [*carbonyl*-^11^C]nicotinamide starting with a reaction of *3*-pyridyl-lithium and [^11^C]CO_2_ in ether, followed by the addition of SOCl_2_ in DMF and last by the addition of liquid NH_3_ (Figure 100A). The [*carbonyl*-^11^C]nicotinamide was prepared within 60 min with a RCY of 20–45% and RCP >99.9% after chromatographic separation.^436,436^

In 1994, Andersson *et al*. prepared [*carbonyl*-^11^C]nicotinamide from the *3*-bromopyridine and [^11^C]HCN utilizing a Pd(0)-assisted coupling reaction and subsequent hydrogen peroxide conversion of the cyano group to the amide with sodium percarbonate. A one-pot procedure for synthesizing the [*carbonyl*-^11^C]nicotinamide was developed (Figure 100B). The synthesis time for the carbon–carbon bond-forming reaction was 5 min. [*Carbonyl*-^11^C]nicotinamide was prepared with a total 30–35 min synthesis time, RCY of 45%, and RCP >99%. The *A*_m_ was in the order of 74 GBq/μmol.^423^

In 2002, Karimi *et al*. added NH_3_ in anhydrous dioxane to a reaction mixture of tetrakis(triphenylphosphine)palladium and *3*-bromopyridine or *3*-iodopyridine, and the vial was shaken just before injection into the micro-autoclave precharged with [^11^C]CO.^422^ The mixture was heated at 180 °C for 5 min, and [*carbonyl*-^11^C]nicotinamide was isolated with the use of a semipreparative LC with RCY of 11 ± 1% (using *3*-bromopyridine) and 54 ± 4% (using 3-iodopyridine) and *A*_m_ of 1600 GBq/μmol.^422^

Another method, developed by Ishii *et al*. in 2015, used [^11^C]CO to prepare the methyl ester of [*carboxyl*-^11^C]niacin (described above), which was treated with aqueous ammonium to give the corresponding [*carbonyl*-^11^C]nicotinamide with RCY of 35 ± 2 5% (Figure 100C).^424^

##### 5.7.2.2 Preclinical Studies

**Figure 101:**
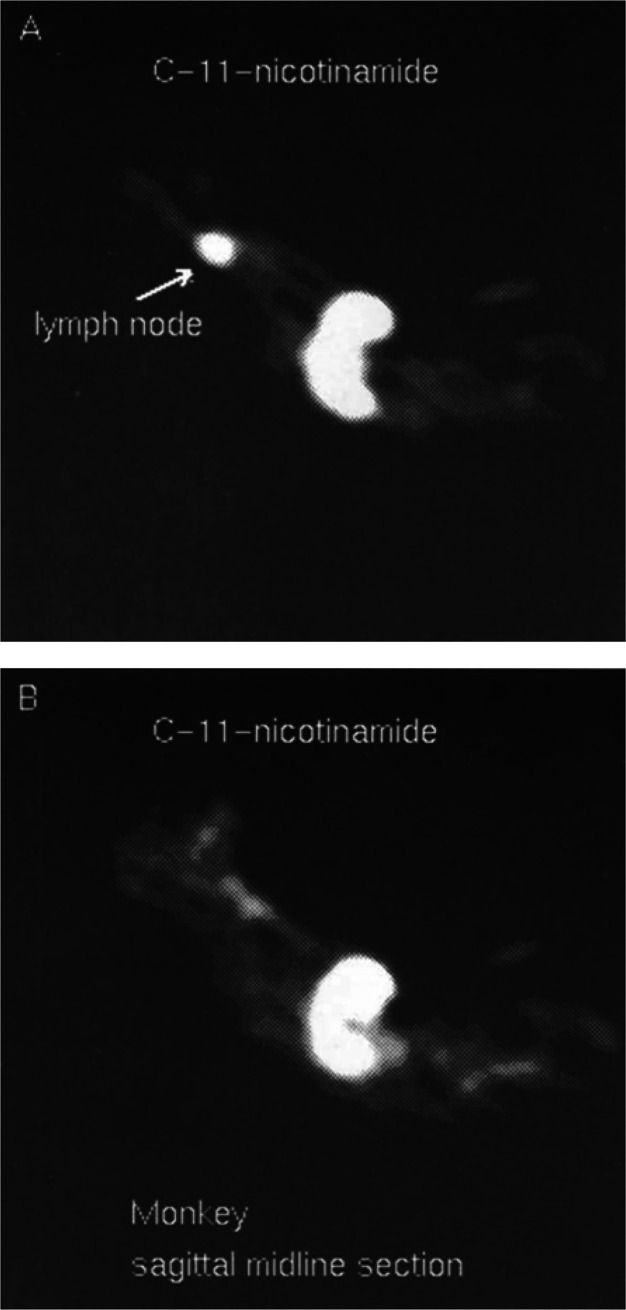
[*Carbonyl*-^11^C]nicotinamide PET scans in a rhesus monkey showing accumulation in lymph nodes (A) and a background section (B). Reproduced with permission from ref (423). Copyright 1994 Elsevier.

Initial distribution and kinetic studies were performed on one rhesus monkey using a dose of 200 MBq as a rapid bolus injection. Immediately p.i., a dynamic imaging sequence was started, including I5 scans for 40 min in a whole-body PET camera (GE 4096). These studies demonstrated that the blood clearance was fast for [*carbonyl*-^11^C]nicotinamide. The brain uptake was low but rapidly accumulated in the liver, kidney, and lymph nodes without significant washout (Figure 101). The blood radioactivity of [*carbonyl-*^11^C]nicotinamide decreased quickly.^423^

### 5.8 *S*-Adenosylmethionine

#### 5.8.1 Radiosynthesis

**Figure 102:**
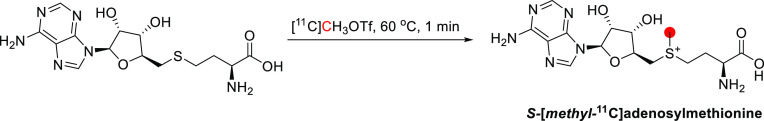
Synthesis of *S*-[*methyl*-^11^C]adenosylmethionine from *S*-adenosyl homocysteine and [^11^C]CH_3_OTf.^387^^11^C radionuclide position is highlighted in red.

The radiosynthesis of *S*-[*methyl*-^11^C]adenosylmethionine was reported in 1981 by Gueguen *et al*. through enzymatic synthesis, prepared by condensation of *l*-[*methyl*-^11^C]methionine with ATP. This is a two-step process: firstly, *l*-[*methyl*-^11^C]methionine is synthesized using [^11^C]CH_3_I and *l*-homocysteine thiolactone as the precursor. Secondly, the enzymatic step involves the incubation of *l*-[*methyl*-^11^C]methionine with ATP, followed by purification. This method yielded 80% (synthesis time 20 min) and a *A*_m_ of 7.33 GBq/μmol 45 min after EOB.^428^ A few years later, Ishiwata *et al.* reported a very similar method but using rat-liver extract as an enzyme source in addition to *l*-[*methyl*-^11^C]methionine and ATP (total synthesis time of 45–50 min, RCY of 29–68%, RCP of 94.2–99.4%, and *A*_m_ of 0.13–0.83 GBq/μmol).^427^ Several limitations are associated with the enzymatic synthesis, such as the preparation of the enzyme source from the rat liver, the two-step synthesis involving the preparation of *l*-[*methyl*-^11^C]methionine before the enzymatic process, and several techniques involved in the product isolation. Therefore, in 2017, Zopollo *et al*. reported the one-pot and automated radiosynthesis of *S*-[*methyl*-^11^C]adenosylmethionine based on the direct *S*-[^11^C]methylation of *S*-adenosyl homocysteine, producing *S*-[*methyl*-^11^C]adenosylmethionine with a RCY of 17 ± 4% (based on [^11^C]CH_3_OTf) within 28 ± 1 min from delivery of the [^11^C]CO_2_ to the formulated final product, and RCP of 97.7 ± 0.3% (Figure 102).^387^*S*-[*Methyl*-^11^C]adenosylmethionine was obtained under GMP conditions using an automated platform *via* the *S*-methylation reaction of SAH with the ^11^C-methylating agent [^11^C]CH_3_OTf. The RCP >90% and *A*_m_ of 207–1363 GBq/μmol taking into account only the (*S*, *S*) isomer.^387,425^

#### 5.8.2 Preclinical Studies

*S*-[*Methyl*-^11^C]adenosylmethionine has been studied preclinically *in vivo* in mice,^426^ rabbits,^428^ and rats^426,427,428^ in healthy or disease-model animals. Biodistribution studies in normal mice, rats, and rabbits have been performed. A biodistribution study of *S*-[*methyl*-^11^C]adenosylmethionine in healthy mice showed a rapid blood clearance with the highest uptake of activity in the bladder and urine, followed by kidneys which might reflect not only the excretion of *S*-[*methyl*-^11^C]adenosylmethionine due to its high hydrophilicity but also the transmethylation into macromolecules. Low uptake was observed in the other organs: heart, liver, spleen, gut, and lungs. In the same study, mice bearing prostate cancer xenografts showed significantly higher tumor uptake with *S*-[*methyl*-^11^C]adenosylmethionine compared with the established prostate radiotracer [^11^C]choline.^425^

Similarly, Ishiwata *et al*. showed that the highest uptake of *S*-[*methyl*-^11^C]adenosylmethionine was found in the kidneys. Accumulation was also observed in the small intestine, pancreas, adrenal gland, liver, and spleen.^427^ In 1985, Ishiwata *et al*. performed biodistribution studies of *S*-[l-^11^C]adenosylmethionine in pregnant rats (16th–19th day of gestation), showing high activity uptake in the blood, placenta, and lung at 5 min p.i. In addition, a relatively higher uptake in the fetal brain was observed compared to the maternal brain.^261^ Finally, a biodistribution study employing this radiotracer in rabbits demonstrated high kidney uptake.^428^

### 5.9 Thiamine

#### 5.9.1 Radiosynthesis

**Figure 103:**
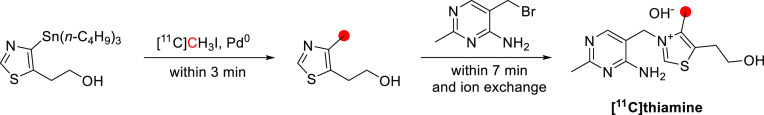
Synthesis of [^11^C]thiamine using [^11^C]CH_3_I. ^11^C radionuclide position is highlighted in red.

The rapid and multistep synthesis involved the (1) incorporation of a [^11^C]methyl group into a heteroaromatic thiazole ring *via* rapid Pd^0^-mediated [^11^C]methylation in the presence of CuBr and CsF at 100 °C for 5 min, and (2) rapid benzylation using 4-amino-5-(bromomethyl)-2-methylpyrimidine hydrobromide in DMF at 150 °C for 7 min (Figure 103). The total synthesis was accomplished within 60 min. The radioactivity of the formulated injectable solution was 400–700 MBq. The RCP was 99%. To obtain higher-quality PET tracers and meet the criteria intended for clinical studies, the synthesis of [^11^C]thiamine was then improved by the adoption of dual-port irradiation in the cyclotron system and further optimization of the reaction conditions and the purification procedures, as shown in Figure 103.^430^

#### 5.9.2 Preclinical Studies

**Figure 104:**
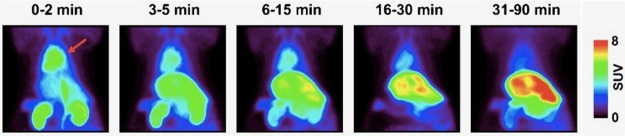
[^11^C]Thiamine PET scans in mice show a rapid washout from the heart. Reproduced with permission from ref (429). Copyright 2018 Springer Nature.

[^11^C]Thiamine was administered in the tail veins of anesthetized rats and placed in the microPET scanner. Emission PET data were acquired for 90 min. The organs were dissected, weighed, and their radioactivity determined. No [^11^C]thiamine accumulation was found in the heart (Figure 104).^429^

### 5.10 Vitamin C

#### 5.10.1 Radiosynthesis

**Figure 105:**
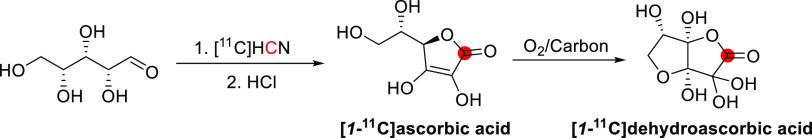
Synthesis of [*1*-^11^C]ascorbic and [*1*-^11^C]dehydroascorbic acid using [^11^C]HCN. ^11^C radionuclide position is highlighted in red.

Cyclotron-produced [^11^C]HCN was eluted with water or aqueous KCN (carrier added) into a vial containing *l*-xylosone (Figure 105). The resulting imine intermediate was hydrolyzed with the addition of HCl and heated for 10 min at 150 °C to yield the desired product. [^11^C]Ascorbic acid was isolated with RCP >99%, with different RCY and *A*_m_ based on the carrier added. [^11^C]Ascorbic acid was then oxidized with rapid bubbling of O_2_ for 10 min, and [^11^C]dehydroascorbic acid was obtained (Figure 105).^405^ [*1*-^11^C]Ascorbic acid has also been synthesized, fully automated, in 35 min from EOB with a RCY of 18.1 ± 2.6%, and *A*_m_ of 15.27 ± 3.76 GBq/μmol.^431^

#### 5.10.2 Preclinical Studies

[*1*-^11^C]Ascorbic and [*1*-^11^C]dehydroascorbic acid have been evaluated in mice^431^ and rats.^405^ Studies of [*1*-^11^C]ascorbic acid in arthritic mice using PET/CT showed significant uptake in the liver and kidney due to high toxin/ROS expression and renal excretion, respectively. The tracer evaluated the effects of indomethacin treatment, resulting in reduced inflammation based on a decrease in tracer uptake.^431^

**Figure 106:**
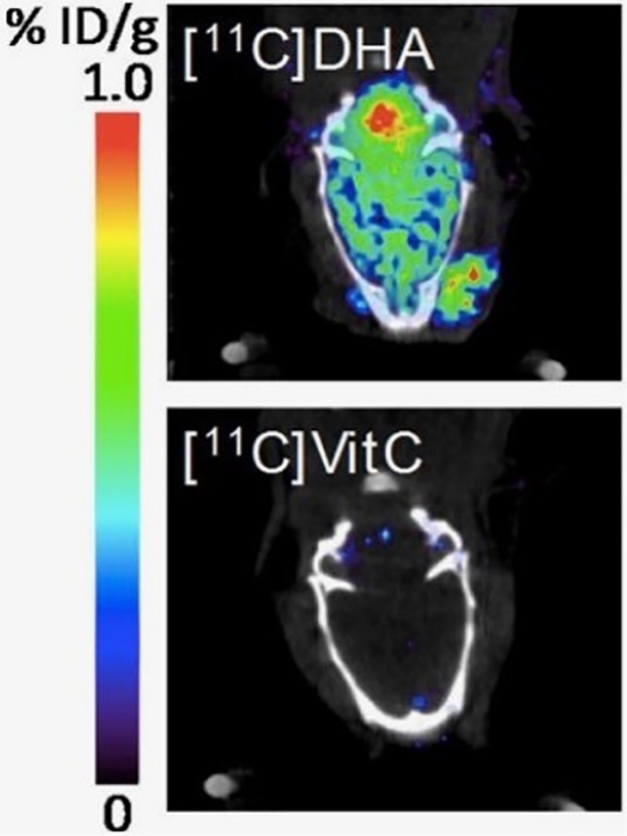
[*1*-^11^C]Ascorbic ([^11^C]VitC) and [^11^C]dehydroascorbic acid ([^11^C]DHA) microPET images in a rat brain (0–30 min p.i.). Reproduced with permission from ref (405). Copyright 2016 Royal Society of Chemistry.

[*1*-^11^C]Ascorbic and [*1*-^11^C]dehydroascorbic acid ([^11^C]DHA) were administered to normal rats *via* tail vein injection, and a 40 min dynamic scan was obtained using a micro/PET-CT scan in the brain (Figure 106). Accumulation of [^11^C]DHA was remarkably higher than that of [*1*-^11^C]ascorbic acid, confirming that only dehydroascorbic acid crosses the BBB transported by GLUT1.^405^ The results also confirm the authors’ hypothesis regarding the detection of changes in uptake based on oxidized *vs* reduced forms of ascorbic acid using PET. In contrast, the transport of vitamin C into the brain *via* SVCT2 is a slower process. [*1*-^11^C]Ascorbic showed lower accumulation than [^11^C]DHA in the brain, but higher retention in the lung and liver 1 h after administration, possibly due to high expression of SVCT in these tissues. [^11^C]DHA reduction to [*1*-^11^C]ascorbic acid represents a potential trapping mechanism, with unreduced [^11^C]DHA likely washed out of the cell. However, there was no difference in the ^11^C radiopharmaceutical retention rate in major organs between the normal and diethyl maleate (DEM) treated groups.^437^

## 6 Endogenous Gases

Biologically active gases naturally occurring in the body include CO_2_, CO, and CH_4_. In the human body, CO_2_ is formed as a waste product of cellular respiration from the metabolism of carbohydrates, fats, and AAs. The body removes the excess CO_2_ by exhalation.^438,439^ The central role of CO_2_ in the body is to regulate the blood’s pH and maintain a physiological acid–base balance. The latter is processed by the bicarbonate buffering system in which carbonic anhydrase catalyzes the reaction that converts CO_2_ and water into H_2_CO_3_. In turn, it rapidly dissociates into H^+^ and HCO_3_^–^ as per the following equation: CO_2_ + H_2_O ↔ H_2_CO_3_ ↔ H^+^ + HCO_3_^–^.^438,440^ CO has shown a broad and remarkable spectrum of biological activity in several tissues, including: antiproliferative, anti-inflammatory, antiatherogenic, and antiapoptotic, and vasodilating. The endogenous CO is produced from the oxidative degradation of heme proteins (heme catabolism) *via* the enzyme heme oxygenase, which degrades heme to produce CO and biliverdin.^441^ The CO produced is removed from the cell *via* diffusion to the blood, where it avidly binds to hemoglobin (Hb), and then it is transported as carboxyhemoglobin until it is solely excreted *via* the lungs.^442^ Methane is an alkane involved in critical biological properties such as anti-inflammatory, antioxidant, and antiapoptotic. It is a constituent of human breath derived from bacterial fermentation in the intestinal lumen, in which anaerobic flora converts undigested carbohydrates into CH_4_.^443^ These have all been radiolabeled with carbon-11, mainly for brain imaging, to quantify blood volume and perfusion in several organs, in the 1980s. However, [^11^C]CO_2,_ [^11^C]CO and [^11^C]CH_4_ are also important labeling precursors for a variety of functionalized molecules (Table 5).

**Table 5:** Carbon-11 Labeled Endogenous Gases

compd	preclinical and clinical studies	target gas	synthon	ref
carbon dioxide	rats,^444^ dogs,^445^ monkeys,^445^ humans^446−450^	N_2_/O_2_ 99.5/0.5	NA^a^	(451)
carbon monoxide	rats,^452^ rabbits,^452−454^ monkeys,^452−454^ humans^455−461^	N_2_/O_2_ 99.5/0.5	[^11^C]CO_2_	(462)

methane	dogs,^463^ humans^463,464^	N_2_/O_2_ 99.5/0.5	[^11^C]CO_2_	(458,464,465)
		N_2_+10%H_2_	NA	(466)

a NA: not applicable.

### 6.1 Carbon Dioxide

#### 6.1.1 Radiosynthesis

^11^C is produced mainly as [^11^C]CO_2_, one of the most common and versatile primary labeling precursors used as a starting point for synthesizing many ^11^C-labeled compounds.^467^ [^11^C]CO_2_ is produced using a cyclotron in high *A*_m_ (111–222 GBq/μmol) by the bombardment of nitrogen gas with high-energy protons.^451^ The preparation of [^11^C]CO_2_ in most of the preclinical and clinical studies has been described by Welch *et al*.^468^

Johnson *et al*. absorbed [^11^C]CO_2_ as [^11^C]HCO^_3_–^ in a NaOH solution, then titrated it with HCl to the desired pH. In this case, at a pH below 5, over 95% of the ^11^C was in the form of CO_2_.^449^ Shields *et al*. trapped [^11^C]CO_2_ using liquid argon and then bubbled through whole blood with nitrogen gas for less than 5 min before injecting, resulting in a mixture of labeled CO_2_ and HCO_3_^–^. Physiologic saline was added, and the solution was injected into dogs. The RCP was over >99% using gas chromatography,^445^ obtaining *A*_m_ of 14.8 GBq/μmol.^444^ Regarding the gas preparation for inhalation studies, no reagents were required in the system, and the gas was passed through a copper measuring spiral in a high-pressure ionization chamber. Then, the gas could be dispensed either continuously or batch-wise.^469^

#### 6.1.2 Preclinical Studies

[^11^C]CO_2_ was studied in rats, dogs, and monkeys, who received the administration *via* inhalation or iv injection. However, to our knowledge, no studies have yet assessed the whole-body biodistribution in healthy animals.

*In vivo* evaluation of brain acid–base measurements by male Wistar rats were achieved using a single-breath inhalation of [^11^C]CO_2_. Lockwood *et al*. found that 30% of the label was metabolically trapped in the brain within 30 min p.i.al.^444^

Shields *et al*. demonstrated that the injection of [^11^C]CO_2_ in dogs generated 33% of its excretion by exhalation in the first 20 min and 56% over the first hour.^445^ Moreover, approximately 10% of the blood activity was converted into a nonvolatile form over 60 min p.i. of [^11^C]CO_2_.^445^

#### 6.1.3 Clinical Studies

Only a few studies have reported using [^11^C]CO_2_ in healthy humans. Normal biodistribution of [^11^C]CO_2_ in the clinical setting has been determined by Brooks *et al*.^446^ Four normal subjects were enrolled in the study, and serial images of the brain were taken during continuous inhalation of [^11^C]CO_2_. Normal biodistribution is seen especially in the peripheral cortical grey matter, followed by whole brain and white matter uptake.^446^

The first study in humans with [^11^C]CO_2_ dates back to 1962, and investigations were conducted until the late 1980s with the administration of the radiotracer *via* gas inhalation either by continuous inhalation or rebreathing of [^11^C]CO_2_ from a rubber bag diluted with air.^447^

Fowler *et al*. studied the distribution of [^11^C]CO_2_ in relation to the immediate CO_2_ storage capacity. It was found that the excretion of [^11^C]CO_2_*via* inhalation in two patients was 49% and 69% of the injected activity over 45 min.^448^ Interestingly, these results were consistent with those of dogs studied by Shields *et al.*, which also found a similar excretion rate of 56% over the first hour.^445^

[^11^C]CO_2_ readily diffuses across the intact BBB,^449^ so inhalation of the gas has also been used to measure the brain pH, providing information on the regional brain tissue acid–base. Brooks *et al*. also investigated the brain pH in patients with brain tumors as well as the effect of BBB disruption after continuous inhalation of [^11^C]CO_2_.^450^ Tumors with a disrupted BBB had a similar regional brain pH (mean pH 6.98) to that of equivalent regions of contralateral tissue (mean pH 6.99). Furthermore, tumors with an intact BBB were found to be more alkaline (mean pH 7.09) and less aggressive than tumors with a disrupted BBB.^446^

### 6.2 Carbon Monoxide

#### 6.2.1 Radiosynthesis

The most common methods for [^11^C]CO production include the reduction of [^11^C]CO_2_ through a heated column pre-charged with a reducing agent, which could be metallic zinc at 400 °C or molybdenum at 850 °C.^470^ However, catalysts and specialist equipment are also required besides the high temperatures. Zeisler *et al*. produced [^11^C]CO from [^11^C]CO_2_ after reaction with molybdenum, in which the unconverted [^11^C]CO_2_ was removed by passing a gas stream through soda-lime, and [^11^C]CO was collected in a silica trap cooled with liquid argon, obtaining a RCY of 54% 15 min at the EOB and *A*_m_ of 555 GBq/μmol.^462^ A new method developed by Dahl *et al*.^471^ used zinc supported on fused silica particles at 485°C, which is above the melting point of zinc (420°C), producing [^11^C]CO with a RCY >96%.^471^

Several other methods have also been investigated, from chemical conversion of [^11^C]CO_2_ by fixation with silyllithium^472^ or disilane^473^ to the decomposition of [^11^C]formate^470^ and [^11^C]formyl chloride^470^ to [^11^C]CO. All of these methods have been recently reviewed by Eriksson *et al.*.^474^

#### 6.2.2 Preclinical Studies

**Figure 107:**
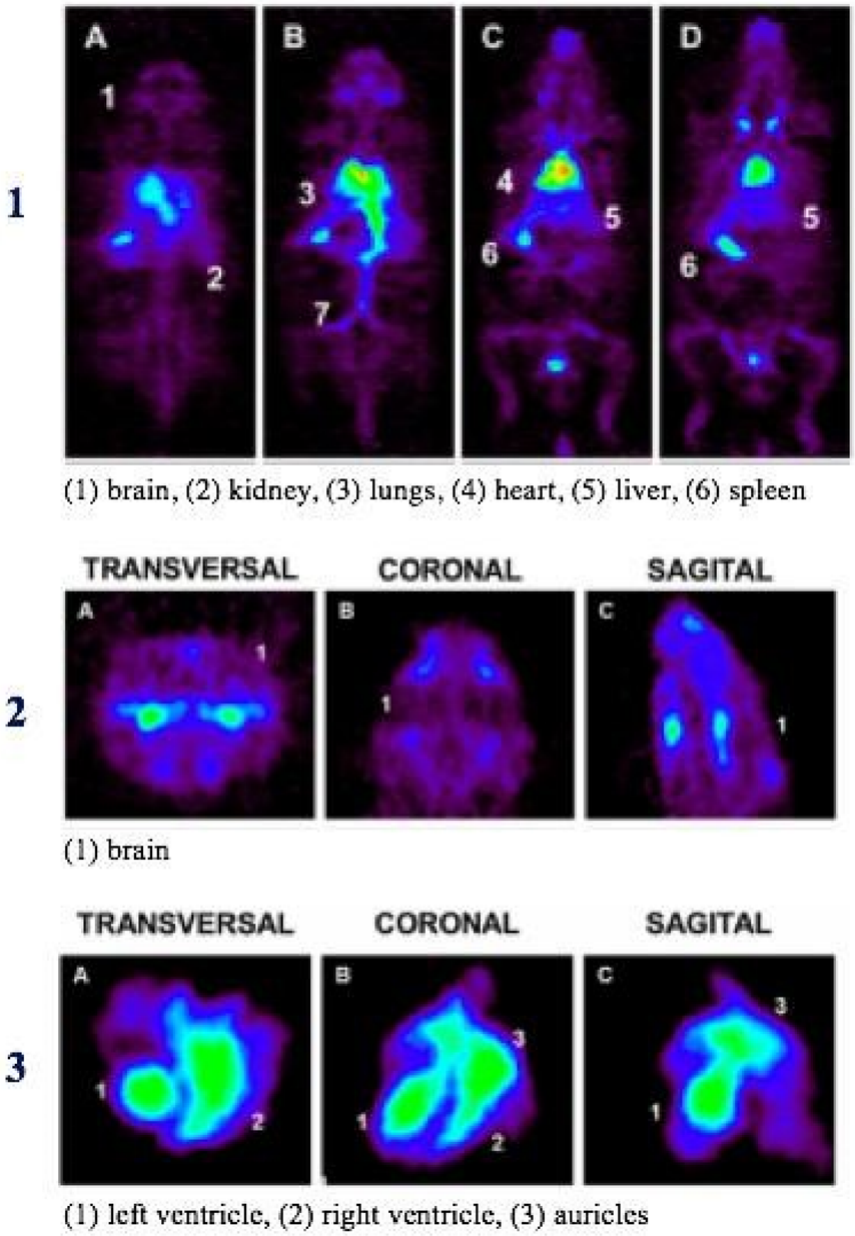
[^11^C]CO biodistribution studies in adult male Sprague-Dawley rats showing (**1**) whole-body, (**2**) brain, and (**3**) heart distribution. Reproduced with permission from ref (452). Copyright 2013 John Wiley and Sons.

*In vivo* studies have been conducted in rats, rabbits, and rhesus monkeys to quantify blood volume in several organs and study the effect of thermal injury on blood volume.^452−454^ A biodistribution study of [^11^C]CO on four adult male Sprague-Dawley rats was performed by [^11^C]CO inhalation. Blood volume quantification was investigated, and whole-body dynamic images were acquired for 30 min. *In vivo* biodistribution showed favorable results, with the major vessels and cardiac cavities having the highest radiotracer accumulation, followed by the lungs. Radioactivity in the liver, kidneys, and brain was low (Figure 107).^452^

#### 6.2.3 Clinical Studies

Shishido *et al*. published the biodistribution of [^11^C]CO in healthy individuals after a single-breath inhalation of the radiotracer. Increased uptake has been noted in areas of high blood pooling, such as venous sinuses, the great vein of Galen, and an insular portion.^455^ Also, the uptake in the grey matter concerning white matter is higher due to the greater blood volume in this structure.

**Figure 108:**
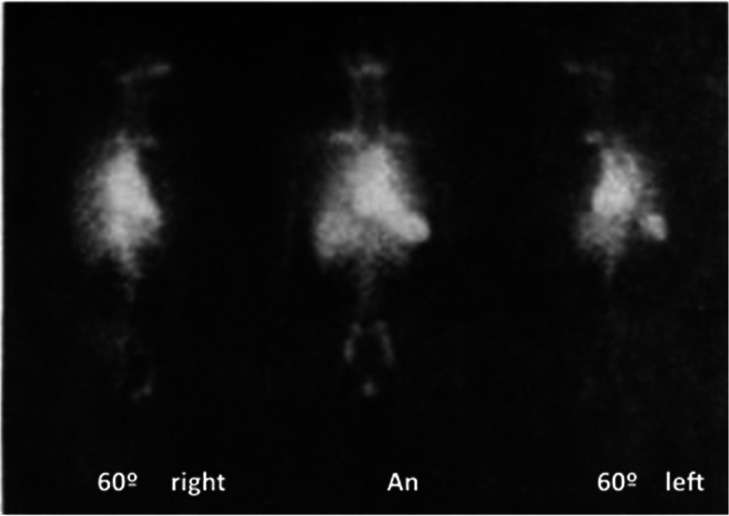
[^11^C]CO inhalation whole-body PET scans in humans. Reproduced with permission from ref (457). Copyright 1979 Society of Nuclear Medicine. This work is licensed under a Creative Commons Attribution 4.0 International License (https://creativecommons.org/licenses/by/4.0/).

Weinreich *et al*. also observed that after a single-breath inhalation, [^11^C]CO was longest retained in the blood, head, and liver.^456^ Despite the blurred and poor image quality, Figure 108 represents the whole-body and upper torso biodistribution of [^11^C]CO, where increased uptake is seen in the liver, spleen, lungs, and heart.^457^

No metabolism of [^11^C]CO into [^11^C]CO_2_ or other compounds has been observed. Therefore, part of the radiotracer migrates from the blood into the intercellular space while the rest is exhaled intact.^456^

[^11^C]CO was one of the first ^11^C-radiopharmaceutical for imaging the blood volume measurements in humans,^458^ one of its main clinical applications, studied under several conditions, especially in cerebrovascular diseases and cancer (breast, brain).^459^

Shishido *et al*. evaluated the blood volume in cerebrovascular diseases. It was found that the quantitative accuracy of PET in measuring brain blood volume and perfusion with [^11^C]CO might allow the location and assessment of cerebrovascular diseases.^455^

**Figure 109:**
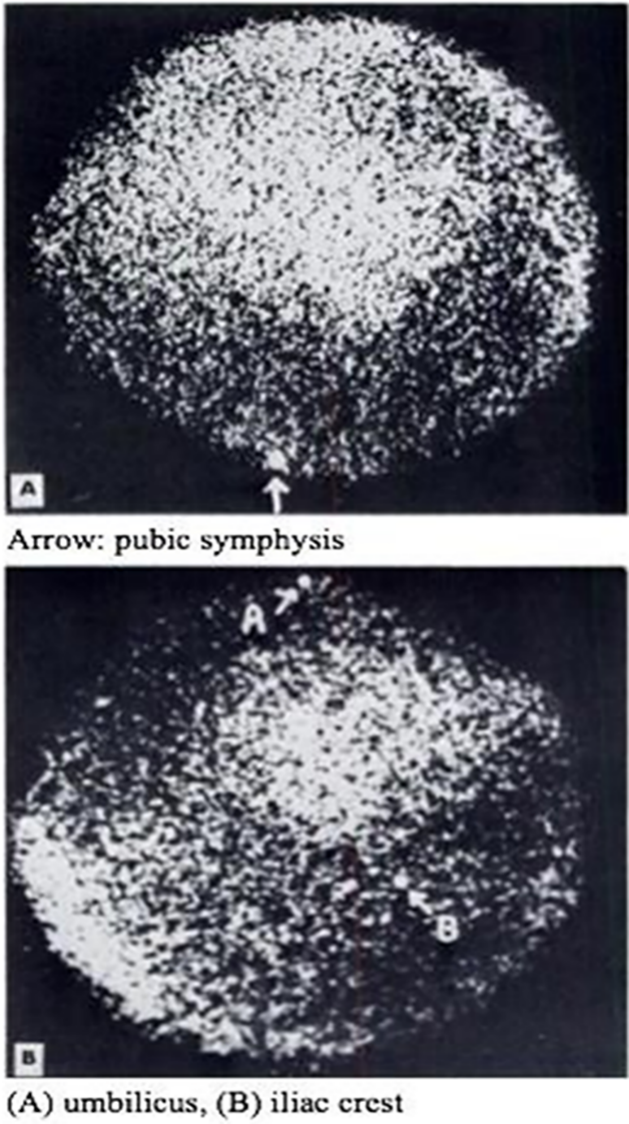
[^11^C]CO gamma camera scans of the abdominal cavity for placenta localization. (A) Anterior projection and (B) right lateral projection. Reproduced with permission from ref (461). Copyright 1968 Society of Nuclear Medicine. This work is licensed under a Creative Commons Attribution 4.0 International License (https://creativecommons.org/licenses/by/4.0/).

[^11^C]CO has also measured tumor blood volume in prostate and breast cancer.^460^ Interestingly, some other applications of [^11^C]CO are placental localization, which can manage antepartum hemorrhage. The same research group investigated the use of [^11^C]CO in two studies involving 135 patients. Overall, it was found that it is possible to localize the placenta in any of its positions accurately (anterior, posterior, lateral), as represented in Figure 109.^461^

### 6.3 Methane

#### 6.3.1 Radiosynthesis

[^11^C]CH_4_ is produced in the gas target during proton bombardment of nitrogen-14 in the presence of 5%^475^ or 10% H_2_^466^ as well as by reduction of purified [^11^C]CO_2_ with over hot nickel (375–450 °C).^458,464,465^

#### 6.3.2 Preclinical Studies

**Figure 110:**
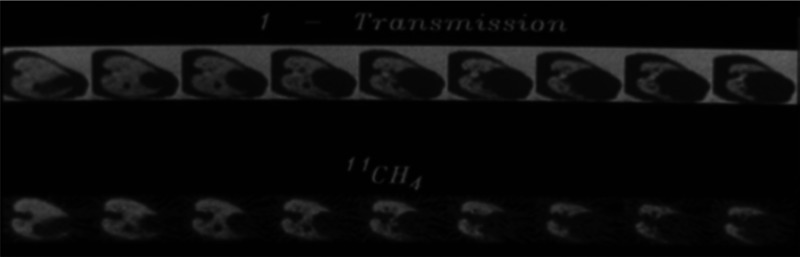
Distribution of pulmonary gas volume during transmission (upper image) and emission images in dogs with [^11^C]CH_4_ (lower image). Reproduced with permission from ref (463). Copyright 1996 Wolters Kluwer Health.

The regional cerebral blood flow has been investigated *via* inhalation of [^11^C]CH_4_ in the myocardium of dogs (Figure 110).^463^

#### 6.3.3 Clinical Studies

The regional cerebral blood flow has been investigated *via* inhalation of [^11^C]CH_4_ in two healthy volunteers.^464^ [^11^C]CH_4_ has also been used to assess pulmonary gas distribution, which has proven effective in human studies and eliminates the need for an additional lung emission scan.^463^

## 7 Fatty Acids

Fatty acids (FAs) are ubiquitous molecules shared among the three domains of life, which represent the building blocks of the lipids’ family. They constitute the major components of triglycerides, phospholipids, and other complex lipids.^476^ FAs act as energy sources, thus representing the main contributor to human dietary fats and essential membrane constituents. Furthermore, they modulate cell metabolism, function, and signaling by regulating gene expression, transcription factor activity, and membrane structure.^477^ In humans, only two FAs are recognized as essential, those necessary for the biological function to be introduced from dietary sources (*e.g*., linoleic and α-linolenic acid), while the others are physiologically produced or commonly assumed with the diet.^478^

In addition to their fundamental impact on human health, FAs, and their metabolic dysregulation can also influence several diseases, including cardiovascular and inflammatory disorders.^477^ Taken these premises, combined with their distinctive carbon backbone, which allows several radiolabeling sites, ^11^C-labeled FAs represent essential family radiotracers, allowing us to deepen the role of FAs at the physiological level (Table 6):

- Acetic acid, obtained from dietary intake and the catabolism of endogenous compounds, has a pivotal role in cellular function as an essential building block for lipid biosynthesis and cell metabolism.^479−483^ Due to its peculiar cellular involvement, a promising role in PET investigations has been ascribed, especially for imaging tissues correlated to high metabolic activity.^484^
- Acetoacetic acid and β-hydroxybutyrate are produced mainly in the liver from the β-oxidation of FAs. During low carbohydrate intake or fasting, it replaces glucose as fuel for high-metabolic rate organs and acts as a starting point for lipid biosynthesis. Renewed interest in ketone bodies and their promiscuous metabolism depending on different patho/physiological conditions make labeled acetoacetic acid an interesting PET probe.^485^ [^11^C]Acetone is widely used as the building block for the radiosynthesis of labeled drugs.^486,487^
- Arachidonic acid is a crucial second messenger and regulates inflammatory processes, ion channel activity, membrane fluidity, and synaptic plasticity.^488,489^
- Suberic, azelaic, and sebacic acids are endogenous alkyl dicarboxylic acids produced during the β-oxidation of FAs, found in trace amounts in human urine,^490,491^ although their concentration significantly increases during metabolic disorders.^491,492^
- Butyric acid is produced by several types of bacteria that can be found naturally in the human colon. Its isobutyric isomer can also be found in smaller amounts. Butyric acid dissociated to its anionic form, butyrate, absorbed and metabolized rapidly in the mitochondria to produce energy.^493,494^
- Decanoic acid is involved in the biosynthesis of long-chain fatty acids and has been shown to exert potent antimicrobial and anti-inflammatory effects.^495^
- Docosohexaenoic acid is one of the more prevalent fats in the brain, with up to 40% of FAs in some gray matter regions and a fundamental role in membrane and synaptic plasticity or neuroreceptor signaling. It is essential in fetal brain and retina development.^496−499^
- Dodecanoic acid belongs to the class of medium-chain fatty acids recognized as a marker of several metabolic disorders.^495,500,501^
- Formic acid is an essential building block in purine synthesis, thymidylate synthesis, and the provision of methyl groups for synthetic, regulatory, and epigenetic methylation reactions.^502^ Although no direct PET investigations have been performed with [^11^C]formate, it has been observed as a radiometabolite following *in vivo* tracer degradation *via* demethylation of ^11^C-methylated tracers.^38,503−511^
- Hexanoic acid is mainly present in humans as a metabolite of FA catabolism.^512^ In PET imaging, it has been evaluated to assess FA metabolism in myocardial and brain tissue.^513,514^
- Linoleic acid is an essential FA crucial for the biosynthesis of arachidonic acid. Due to its biological role, it could be used for visualizing FAs metabolism in physio-/pathological conditions.^515^
- Octanoic acid is a metabolite of FA catabolism in humans and is used to visualize FA metabolism in CNS as a glutamate/glutamine metabolism marker.
- Stearic acid is one of the most common saturated FAs in nature and can be biosynthesized by elongating palmitic acid by FA elongases.^516^ Stearate may also be desaturated by the enzyme stearoyl-CoA desaturase to form oleic acid. Oleic acid is the most common fatty acid in nature and is present in fats, phospholipid membranes, cholesterol esters, and wax esters.^517^^11^C-labeled stearic and oleic acids have been used to evaluate myocardial metabolism in preclinical studies.^518,519^ A series of long-chain saturated FAs, including stearic acid, have been radiolabeled as potential cardiac imaging agents; however, no human studies have been reported with [^11^C]stearic acid or [^11^C]oleic acid.
- Palmitic acid is the most abundant saturated FA in the human body, accounting for 20–30% of total FAs in membrane phospholipids. PET studies with radiolabeled palmitic acid can help to deepen our knowledge of its metabolism in physio-/pathological conditions.^520^
- Pentanoic acid, produced by the gut microbiota, is mainly present in the intestine. Recently, it has been correlated to promising gut protective effects and histone deacetylase (HDAC) inhibitory potencies, other than being a potential marker of metabolic disorders.^521−523^
- Propanoic acid is obtained from dietary sources but mainly from fermenting undigested food, such as carbohydrates, peptides, and FAs, by the anaerobic colonic microbiota. Furthermore, propanoic acid is considered a major energy source in ruminants. Thus, it is a major mediator in the link between nutrition, microbiota, and physiology in the human body, mainly in inflammatory conditions, by lowering plasma FA levels.
- Tetradecanoic acid is involved in endogenous FA biosynthesis. It mainly accumulates fat in the body and acts as a cardioprotective agent and metabolic process regulator.^524^

**Table 6:** Carbon-11 Labeled Enzyme Fatty Acids

compd	radiolabeling position	preclinical and clinical studies	synthon	*A*_M_ (GBq/μmol)	RCY	total time (min)	ref
acetic acid	*1*-	mice,^525−527^ rats,^525,528^ pigs,^432,529,530^ dogs,^531^ monkeys,^432,530^ humans^532^	[^11^C]CO_2_	200	43%	30	(533)
	*2*-	pigs^432^	[^11^C]CH_3_I	>0.5	43%	25	(432)

acetoacetic acid	*1*-	rats,^534^ cats,^534^ monkeys,^535^ humans^536−542^	[^11^C]CO_2_	66.6	35%	16	(535)
acetone	*2*-	baboons^543^	[^11^C]CO_2_	100	54%	48	(486)

arachidonic acid	*1*-	monkeys,^544^ humans^545−550^	[^11^C]CO_2_	3.7	23%	35	(551,545)
	*19*-	nr^a^	[^11^C]CH_3_CH_2_I	1.6	23%	52	(552)

azelaic, sebacic, and suberic acids	*1*-	nr	[^11^C]NH_4_CN	15^b^	40%	60	(553)

butyric acid	*1*-	rats,^554^ dogs,^531^ baboons^c^,^555^ humans^c^^556^	[^11^C]CO_2_	37	50%	40	(555)
	*4*-		[^11^C]CH_3_I	nr	64%	45	(557)
isobutyric acid	*1*-	dogs^531^	[^11^C]CO_2_	nr	96%	20	(531)
decanoic acid	*10*-	nr	[^11^C]CH_3_I	nr	35%	44	(558)
docosahexaenoic acid	*1*-	monkeys,^590^ humans^559,560^	[^11^C]CO_2_	>18.5	18.3%	<43	(551,559)
dodecanoic acid	*12*-	pigs^247,561^	[^11^C]CH_3_I	nr	28%	45	(558)
formic acid	*1*-	nr	[^11^C]CO_2_	9	>98%	nr	(507,508)

hexanoic acid	*1*-	mice,^513,514^ cats,^562^ dogs^531^	[^11^C]CO_2_	nr	71%	15	(563)
	*6*-	nr	[^11^C]CH_3_I	nr	36%	47	(558)

linoleic acid	*18*-	nr	[^11^C]CH_3_I	20	48%	45	(564)

octanoic acid	*1*-	mice,^565^ rats,^566,567^ cats,^566^ dogs,^531^ pigs,^561^ humans^565,568−570^	[^11^C]CO_2_	nr	64%	35	(558)
	*7*-	nr	[^11^C]CH_3_CH_2_I	nr	23%	52	(558)
	*8*-	nr	[^11^C]CH_3_I	nr	32%	44	(558)

oleic and stearic acid	*1*-	dogs^518,519^	[^11^C]CO_2_	nr	nr	nr	(519)
			[^11^C]NaCN	nr	83%	50	(571)

palmitic acid	*1*-	rats,^572^ dogs,^572^ pigs,^561,573,574^ monkeys,^575^ humans^574^	[^11^C]CO_2_	12.95	38%	8	(576−578)
			[^11^C]HCN	nr	78%	58	(315)
	*8*- and *14*-	nr	[^11^C]CH_3_(CH_2_)_7_CH_2_I	nr	22%	65	(558)
	*16*-	rats,^572^ dogs^572^	[^11^C]CH_3_I	nr	73%	46	(557)

pentanoic acid	*1*-	dogs^531^	[^11^C]CO_2_	nr	59%	47	(531)
	*5*-	nr	[^11^C]CH_3_I	nr	27%	47	(558)

propanoic acid	*1*-	dogs^531^	[^11^C]CO_2_	nr	98%	15	(531)
			[^11^C]CO	nr	nr	3	(579)

tetradecanoic acid	*14*-	pigs^561^	[^11^C]CH_3_I	nr	23%	45	(558)

*β*-hydroxybutyrate	*1*-	humans^580^	[^11^C]CO_2_	nr	10%	36	(581)
			^11^C]NH_4_CN	nr	30%	50	(582,583)

a nr: not reported.

b Azelaic acid.

c Radiolabeling position is not clarified.

### 7.1 Acetic Acid

#### 7.1.1 Radiosynthesis

[*1*-^11^C]Acetic acid radiosynthesis was firstly reported in 1943 by Buchanan and coworkers, exploiting the same procedure reported in detail by Pike *et al*. in 1981.^554,584^ Methyl magnesium bromide in diethyl ether after [^11^C]carbonation under inert atmosphere and a final acidification step with HCl 6 M with further ether extraction, allowed obtaining of [*1*-^11^C]acetic acid in 20 min with 73% RCY and *A*_m_, at time of preparation, exceeding 18.5 GBq/μmol.^409,584^ The same procedure optimized with a remotely-controlled system reduced the preparation time to 11.5 min.^585^ The final solvent extraction represented a limitation for this automated radiosynthesis. Therefore, further simplified purification methods, *i.e.*, solid-phase adsorbent, distillation, or ion-exchange cartridge, were developed, maintaining roughly the same preparation time and RCY.^432,563,586−589^ In 1995, Kruijer *et al*. selectively separated [*1*-^11^C]acetic acid from byproducts through an anion-exchange column in 15 min radiosynthesis and a RCY of 60–65%.^590^ Davenport *et al*. overcame the purification problem using methyl magnesium bromide adsorbed in a narrow tube, efficiently trapping and releasing radioactivity (95% and 92%, respectively) during [^11^C]carboxylation, obtaining [*1*-^11^C]acetic acid in 16 min from radionuclide production with a RCY of 72%.^591^ An automated combination of these two techniques, *i.e.*, loop method for Grignard reagent carboxylation and SPE purification, was set up to obtain [*1*-^11^C]acetic acid with a RCY of 60–70% in 12 min from EOB.^592^ Some of these procedures involved THF as the solvent instead of ether, as reported by Berridge *et al.*, where solvent and impurities were removed by evaporation with ethanol obtaining [*1*-^11^C]acetic acid within 15 min from EOB in RCY of 60%.^593^ More recent automated or fully robotic-controlled procedures generally maintain RCY ∼70% and preparation time 15 min.^594,595^ An alternative radiosynthetic approach passed through hydrolysis of radiolabeled acetyl chloride. The dry salt formed after [^11^C]carboxylation of methyllithium or methyl magnesium bromide reacted with phthaloyl dichloride and was finally trapped as sodium [^11^C]acetate by hydrolysis with isotonic saline, affording [*1*-^11^C]acetic acid within 25–30 min from EOB, RCY of 43% and *A*_m_ 90–200 GBq/μmol at EOS.^533^

**Figure 111:**
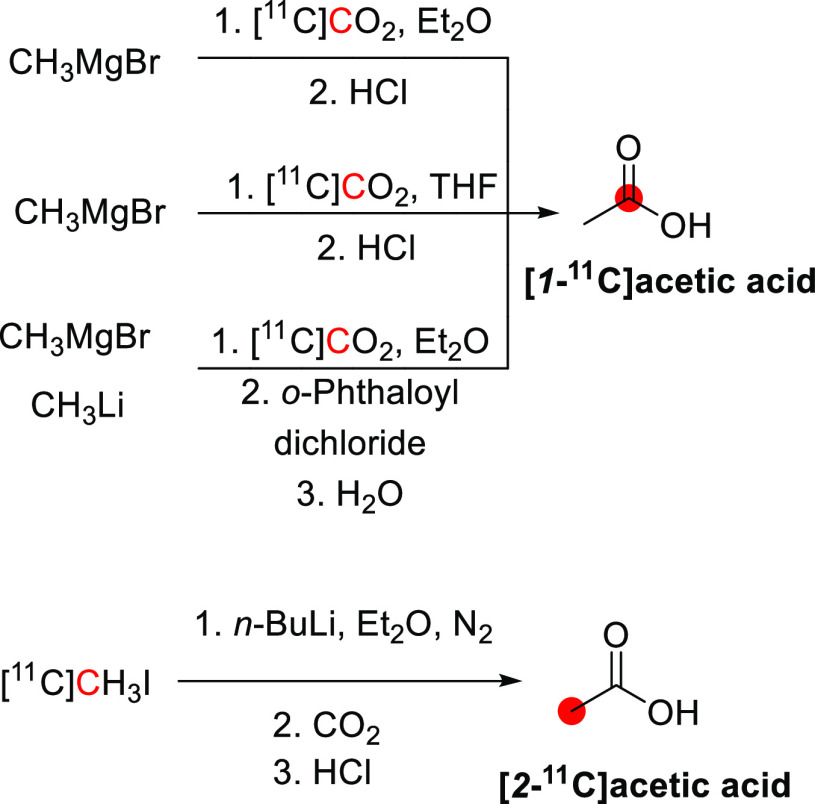
Radiosynthetic schemes of [^11^C]acetic acid. ^11^C Radionuclide position is highlighted in red.

The only reported procedure for [*2*-^11^C]acetic acid (Figure 111) was published in 1994 by Kihlberg *et al.*.^432^ [^11^C]CH_3_I in diethyl ether was firstly converted to [^11^C]CH_3_Li using *n*-butyl lithium, carboxylated at 60 °C with CO_2_ and finally acidified with HCl/NaCl_(aq)_, obtaining [*2*-^11^C]acetic acid within 25 min, RCY of 43%, and *A*_m_ > 0.5 GBq/μmol.^432^ was published in 1994 by Kihlberg *et al*. [^11^C]CH_3_I in diethyl ether was firstly converted to [^11^C]CH_3_Li using *n*-butyl lithium, carboxylated at 60 °C with CO_2_, and finally acidified with HCl/NaCl_(aq)_, obtaining [*2*-^11^C]acetic acid within 25 min, RCY of 43%, and a *A*_m_ > 0.5 GBq/μmol.^432^

[^11^C]Acetate can be routinely produced on a large scale (for several consecutive PET imaging protocols) on various commercially available (reconfigured) or in-house built automated modules *via*^11^C-carboxylation of methyl magnesium chloride/bromide (CH_3_MgCl, CH_3_MgBr) in anhydrous ether solvents (Et_2_O/THF) employing reactor-based (bubbling)^589,595−597^ or captive-solvent methodology.^598−600^ Overall synthesis time is 10–20 min with 30–80% RCY based on [^11^C]CO_2_. However, despite various improvements and flexibility of synthesis modules throughout these years, reliable production of [^11^C]acetate and other ^11^C-labeled fatty acids is still challenging due to the difficulties of using Grignard reagents.

#### 7.1.2 Preclinical Studies

Around 60% of iv injected [*1*-^11^C]acetic acid in rats expired as [^11^C]CO_2_ in the first 15 min.^525^ This was further confirmed by Ng *et al*. performing the [^11^C]CO_2_ metabolite analysis on simultaneously collected arterial and venous blood samples of pigs at different time points, following iv administration of [*1*-^11^C]acetic acid. It was found that a significant fraction of the total radioactivity was represented by the [^11^C]CO_2_ metabolite over 30 min p.i., where [^11^C]CO_2_ rose from 4% to 64%.^529^ A study conducted in prostate tumor-bearing mice evaluated that uptake in the tumor region in the first minute is due to reversibly labeled tumor pool (tricarboxylic acid cycle metabolites and bicarbonate), while irreversibly labeled pool (tumor lipid) is dominant at a second stage. If the latter is confirmed, a higher tumor-to-noise ratio can be achieved later, suggesting a worthwhile effort for longer acquisition PET protocols.^525^ In pigs, after iv injection, *1*- and [*2*-^11^C]acetic acid was rapidly cleared from the blood, where ^11^C arterial radioactivity remained only 10% after 60 s.^432^ [*1*-^11^C]acetic acid concentration in venous blood rapidly decreased after 5 min in monkeys and pigs.^432^ In the monkeys, [*1*-^11^C]acetic acid was mainly uptake in the liver, salivary glands, pancreas, small bowel, and spleen. At the same time, in pigs, its biodistribution was like in monkeys, except for pronounced retention in the renal cortex, gall bladder wall, and bone and less uptake in the salivary gland. Very low excretion of [*1*-^11^C]acetic acid’s metabolites was found in bile or urine in both species.^530^ Biodistribution data in Sprague-Dawley male rats confirmed fast clearance (30–60%) from all organs at 1 h, apart from the pancreas.^528^ In dogs, [*1*-^11^C]acetate showed a very fast progression to diffuse whole-body biodistribution with time with a marked accumulation in the liver and abdomen 2 min p.i.^531^ [*1*-^11^C]Acetic acid was also evaluated in mouse models of multiple myeloma and multidrug resistance gene-2 deficient hepatocellular carcinoma, confirming its ability to detect the presence of cancer cells and response to therapy *in vivo*.^526,527^

#### 7.1.3 Clinical Studies

**Figure 112:**
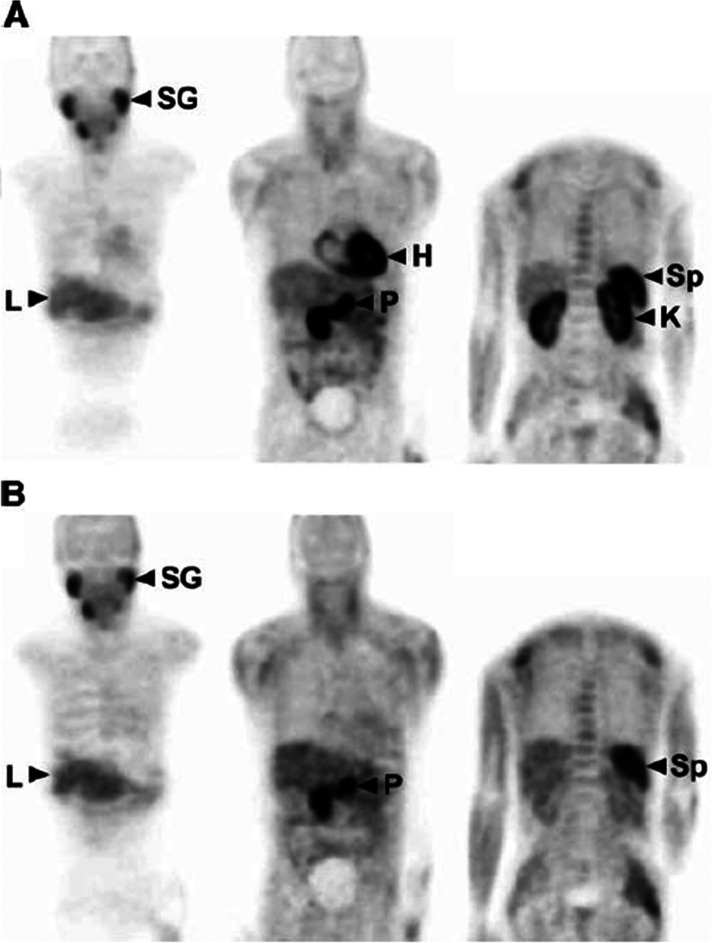
[^11^C]Acetate whole-body PET images at 2 min (A) and 28 min (B) p.i. L = liver, H = heart, K = kidney, P = pancreas, SG = salivary glands, Sp = spleen. Reproduced with permission from ref (532). Copyright 2004 Society of Nuclear Medicine. This work is licensed under a Creative Commons Attribution 4.0 International License (https://creativecommons.org/licenses/by/4.0/).

A preliminary dynamic whole-body PET on healthy volunteers was performed by Seltzer *et al*. to evaluate [*1*-^11^C]acetic acid’s biodistribution (Figure 112).^532^ At the early point (2 min p.i.), intense radiotracer uptake was seen in the salivary glands, heart, pancreas, kidneys, spleen, and bowel. At the late time point (28 min p.i.), activity has almost completely cleared from the heart and kidneys, reflecting the rapid oxidative metabolism of these organs. At the same time, radioactivity is significantly retained in the salivary glands, pancreas, liver, spleen, and bowel. The high tracer concentration within the pancreas is due to a high rate of lipid synthesis within pancreatic acinar cells. No urinary excretion of [*1*-^11^C]acetic acid was detected, supporting the potential advantage of acetate PET in evaluating pelvic tumors.^532^

Further clinical PET studies in abdominal parenchymal organs confirmed the pancreas as the organ with high uptake, with no statistically significant differences found between the head and body of the organ.^601,602^ Other studies highlighted a prolonged clearance of [*1*-^11^C]acetic acid from the pancreas (roughly 64% of activity retained 30 min post-injection) with a pancreas-to-liver ratio 10–20 min post-injection ranging from 2.1 to 4.5, among the normal subjects studied, also suggesting its potential to evaluate exocrine pancreas function and disease-related.^603,604^

[*1*-^11^C]Acetic acid has been exploited to assess oxidative and lipid metabolism in various tissues, including the heart, brown adipose tissue, and pancreas, under physio-/pathological conditions.^603,605−608^ Furthermore, it was evaluated for the detection/evaluation of peripheral tumors (prostate cancer or hepatocellular carcinoma).^609,610^ More recently, it has been evaluated for PET imaging of neurological disorders such as cerebral glioma and multiple sclerosis.^611,612^

### 7.2 Acetoacetic Acid

#### 7.2.1 Radiosynthesis

**Figure 113:**
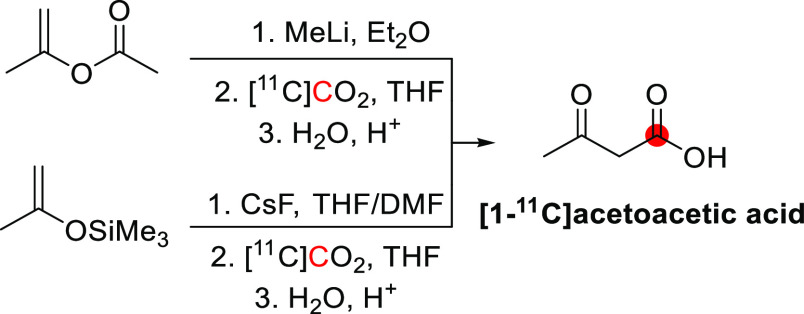
Radiosynthetic schemes of [^11^C]acetoacetic acid. ^11^C radionuclide position is highlighted in red.

Radiosynthesis of [*1*-^11^C]acetoacetic acid (Figure 113) was firstly reported in 1974 from [^11^C]carboxylation in THF of enolate anion of acetone, previously achieved from the reaction between methyl lithium and isoprenyl acetate in ether, and the final acidification which allows reaching [*1*-^11^C]acetoacetic acid in 55% RCY within 40 min from EOB.^613^ Further optimization using an HPLC purification method furnished [1-^11^C]acetoacetic acid with 24–58% RCY and *A*_m_ 0.0222 GBq/μmol 30 min after EOB. In contrast, an automated one-pot synthesis system with an ion-exchange column purification step provided the final compound within 18 min with 34% RCY.^534,614^ The same automated procedure with another synthetic module achieved [*1*-^11^C]acetoacetic acid with higher *A*_m_ (66.6 GBq/μmol) with approximately the same time and RCY (16 min and 35%).^535^ Recently, following a fluorine-mediated desilylation ^11^C-labeling approach, (isopropenyloxy)trimethylsilane was first converted to a cesium enolate intermediate using cesium fluoride in THF/DMF solution, and then [^11^C]carboxylated with the final acidification step, which obtained [*1*-^11^C]acetoacetic acid in 57% RCY within 29 min from [^11^C]CO_2_ collection.^615^

#### 7.2.2 Preclinical Studies

In a female vervet monkey, 30 min post iv injection, organ biodistribution of [*1*-^11^C]acetoacetic acid demonstrated high renal clearance with lower uptake in the heart and liver. In the brain, there was rapid uptake followed by a fast washout.^535^ In rats, after 4 min post iv injection, radioactivity in the heart, liver, kidney, and brain remain constant.^534^

In the cat, the same pattern, with generally higher uptakes, was observed, except for the heart, where after the initial peak, a rapid decrease in the first min until a steady state was reached. Only a very low amount of [*1*-^11^C]acetoacetic acid was excreted through blood in cats.^534^

#### 7.2.3 Clinical Studies

[*1*-^11^C]Acetoacetic acid revealed a promising radiotracer for detecting heart failure and assessing cardiomyopathy in a rat model.^536^ Furthermore, it could be helpful in imaging tissue with a high metabolic rate (tumors or brain), where ketone bodies were used instead of glucose and following how ketone metabolism changed in health and disease.^537−542^

### 7.3 Acetone

#### 7.3.1 Radiosynthesis

**Figure 114:**
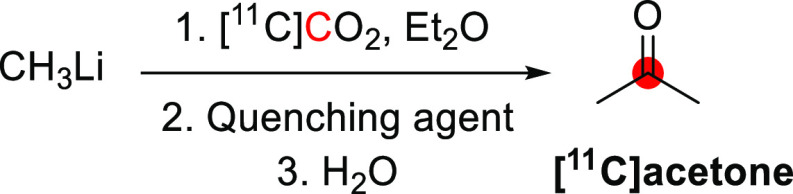
Radiosynthetic scheme of [^11^C]acetone. ^11^C radionuclide position is highlighted in red.

[^11^C]Acetone was obtained through the reaction of [^11^C]CO_2_ with methyl lithium in ether (Figure 114). The excess methyl lithium was further quenched to avoid excess formation of byproduct [^11^C]*tert*-butanol. In this way, [^11^C]acetone was obtained in 30 min with *A*_m_ of 37 GBq/μmol.^616^ Further optimization, by adding diphenylamine as a quenching reagent, increased the yield to 100% of [^11^C]acetone, making it suitable for iv administration.^617^ In the end, there are two quenching steps: the first with diphenylamine to neutralize methyl lithium in excess, while the second with acid water to quench lithium diphenylamide ^11^C-labeled lithium olate complex. [^11^C]Acetone obtained in this way was further achieved with 54% RCY.^486^

#### 7.3.2 Preclinical Studies

[^11^C]Acetone iv injected in baboons revealed a fast uptake in the brain, followed by a slow clearance after 60 min in the cerebellum and white matter.^543^

### 7.4 Arachidonic Acid

#### 7.4.1 Radiosynthesis

**Figure 115:**
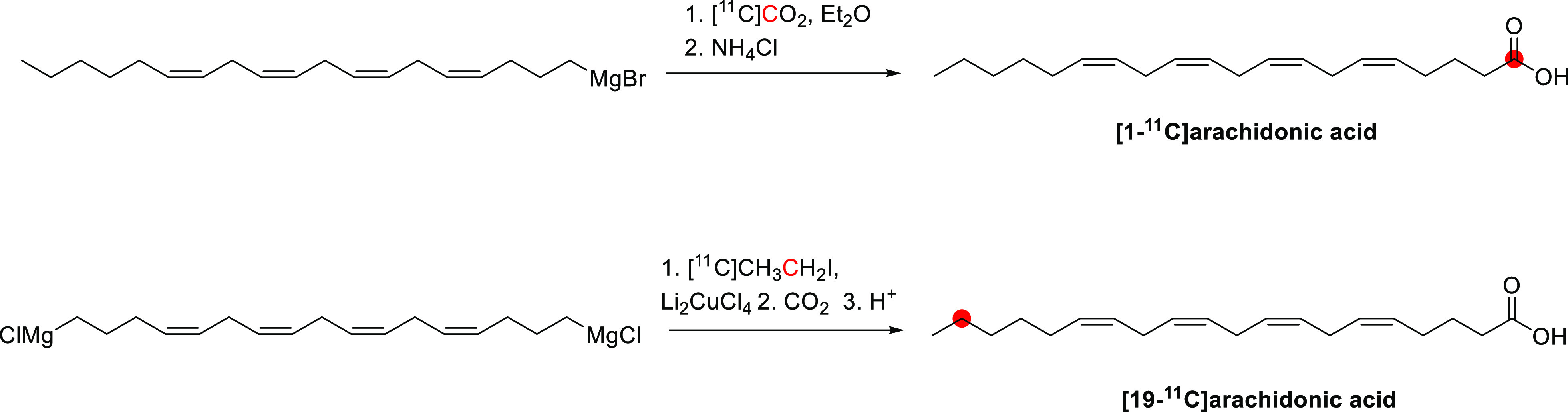
Radiosynthetic schemes of [^11^C]arachidonic acid. ^11^C radionuclide position is highlighted in red.

[*1*-^11^C]Arachidonic acid was synthesized starting from a *1*-bromo precursor, activated by reacting the corresponding Grignard reagent with magnesium in ether (Figure 115). [^11^C]CO_2_ in helium was delivered *in situ*, and the reaction was quenched with NH_4_Cl. Magnesium salts were precipitated, and the crude was purified, obtaining [*1*-^11^C]arachidonic acid with 23% RCY at EOS within 35 min from the EOB.^551^ Further re-radiosynthesis of [*1*-^11^C]arachidonic acid reached *A*_m_, exceeding 3.70 GBq/μmol.^545^

[*19*-^11^C]Arachidonic acid was synthesized starting from bisGrignard reagent through a Cu-mediated reaction with [^11^C]ethyl iodide and final carbonation with 23% RCY and 1.6 GBq/μmol *A*_m_ within 52 min (Figure 115).^552^

#### 7.4.2 Preclinical Studies

[*1*-^11^C]Arachidonic acid administered iv to normocapnic and hypercapnic monkeys were used to evaluate fatty acids incorporation in the brain, examining brain PL metabolism.^544^ Brain uptake reached a maximum of 10 min p.i. with around 0.51% of the injected dose and remained constant in this steady-state for 45 min. [*1*-^11^C]Arachidonic acid showed a plasmatic half-life of only 1.1 min associated with plasma radioactivity falling at 5% by 10 min after infusion. The latter is due to its rapid incorporation in brain lipids with fast peripheric wash-out for oxidative lipid metabolism. [*1*-^11^C]Arachidonic acid brain incorporation rates are 20–40% higher in the whole brain and cortex than in white matter. [*1*-^11^C]Arachidonic acid brain incorporation rates (k*) are not dependent on cerebral blood flow, so much so that incorporation rate (k*) is unaffected by 2.6 increment of CBF due to inhaling 5% CO_2_ in the air because of the high amount of fatty acids bound to plasma protein.^544^

#### 7.4.3 Clinical Studies

Several methods and strategies were attempted to evaluate radiolabeled arachidonic acid uptake and metabolism in the brain. Based on the irreversible uptake model derived from rat studies, the k* was evaluated in healthy adults at rest, equaling 5.6 and 2.6 μL/min·mL in grey and white matter, respectively, plateauing at 15 min, remaining constant from 20 to 60 min.^546^ Further PET analysis also revealed that [*1*-^11^C]arachidonic acid’s brain incorporation rate is not affected by age.^547^ A PET study on [*1*-^11^C]arachidonic acid revealed how proper visual stimulation increases its k* around 2–8% in visual and related areas of the human brain, confirming arachidonic acid’s pivotal role in cellular signaling processes.^548^ [*1*-^11^C]Arachidonic acid’s half-life in plasma was evaluated around 20 min. More patient-friendly and non-invasive methods, *i.e*., population-based metabolite correction and image-derived input function, than common arterial blood-based ones to evaluate [*1*-^11^C]arachidonic acid’s k* were developed, retaining comparable repeatability and validity.^549^

[*1*-^11^C]Arachidonic acid uptake was evaluated in eight Alzheimer’s disease (AD) patients, showing higher brain uptake compared to healthy control, particularly in areas with a high density of neuritic plaques and activated microglia.^545^ [*1*-^11^C]Arachidonic acid was also used to visualize perturbation in dopamine neurotransmission, opening opportunities for further related disease PET studies.^550^

### 7.5 Azelaic, Sebacic, and Suberic Acids

#### 7.5.1 Radiosynthesis

**Figure 116:**
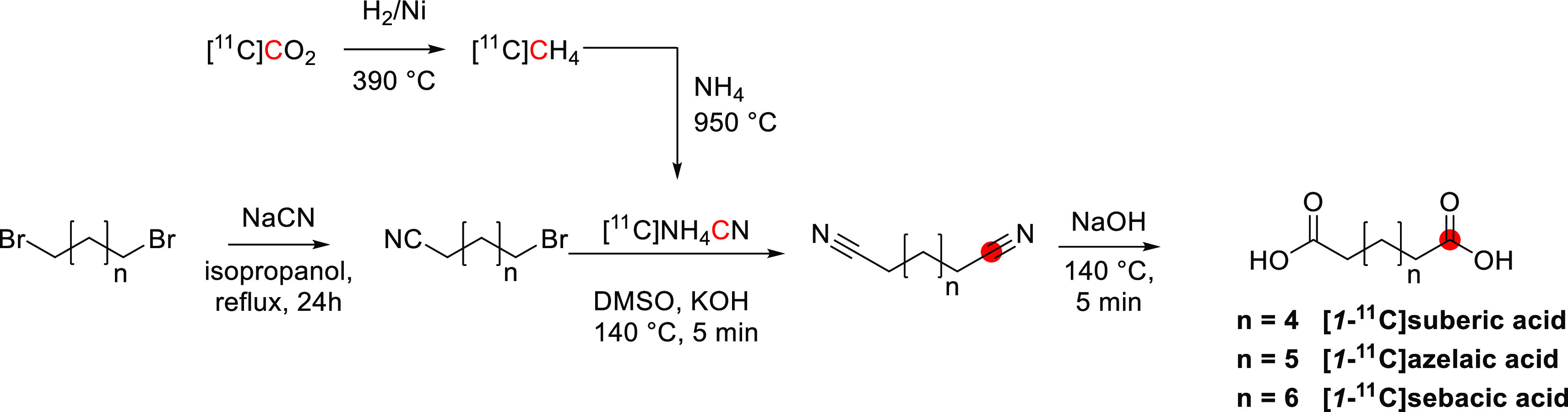
Synthesis of [*1*-^11^C]suberic acid (*n* = 4), [*1*-^11^C]azelaic acid (*n* = 5), and [*1*-^11^C]sebacic acid (*n* = 6) using [^11^C]NH_4_CN. ^11^C radionuclide position is highlighted in red.

The ^11^C-labeling of suberic, azelaic, and sebacic acids was developed using [^11^C]NH_4_CN as a radioactive synthon. A dibromoalkane derivative (Figure 116) was initially converted into the corresponding bromonitrile derivative by reaction with NaCN in refluxing 2-propanol for 24 h (33–35%). [^11^C]NH_4_CN was prepared by reduction of [^11^C]CO_2_ to [^11^C]CH_4_ over nickel catalysis and subsequent reaction with ammonia gas in a palladium furnace (Figure 116). [^11^C]NH_4_CN was then added to the bromonitrile analogue and reacted in DMSO and in the presence of potassium hydroxide for 5 min at 140 °C to produce the corresponding ^11^C-labeled dinitrile species (Figure 116) with non-isolated RCYs of 83–90% (calculated by radioTLC). The ^11^C-dinitrile intermediate was then hydrolyzed to the desired ^11^C-carboxylic acid by aqueous NaOH at 140 °C for 5 min. The total synthesis time was 60 min from EOB, and all three products were obtained with isolated RCYs ranging between 30 and 40% (relative to trapped [^11^C]NH_4_CN) and *A*_m_ of 15 GBq/μmol (calculated only for [*1*-^11^C]azelaic acid).^553^

### 7.6 Butyric and Isobutyric Acids

#### 7.6.1 Radiosynthesis

**Figure 117:**
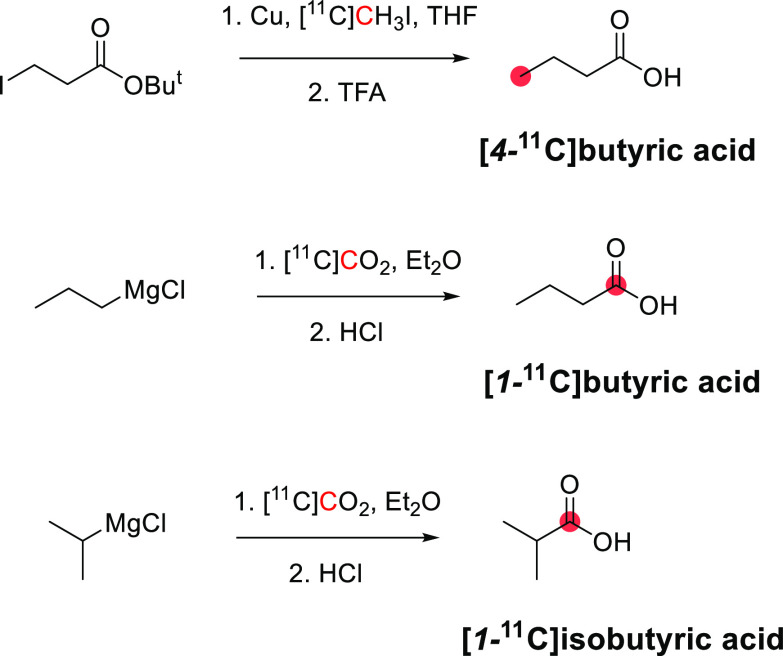
Synthesis of [^11^C]butyric and [^11^C]isobutyric acid. ^11^C radionuclide position is highlighted in red.

There are two strategies to radiolabel butyric acid with carbon-11. The first one was proposed in 1997 to radiolabel butyric acid in position 4 (Figure 117). [^11^C]CH_3_I reacted with *3*-iodopropionic acid *tert*-butyl ester and copper complex, then heated for 1 min at 70 °C, cooled rapidly at 0 °C, and deprotection with trifluoroacetic acid occurred. The mixture was heated for 5 min at 70 °C and purified through solid-phase extraction and semipreparative HPLC. In this way, [4-^11^C]butyric acid was obtained within 45 min from the end of radionuclide production with 64 ± 7% isolated RCY.^557^ [*1*-^11^C]Butyric acid and [*1*-^11^C]isobutyric acid was obtained using the corresponding Grignard reagent and [^11^C]CO_2_ in anhydrous ether (Figure 117). After carbonation, Grignard reagents were hydrolyzed under acidic conditions, and the [^11^C]carboxylic acids obtained were ultimately treated with a 6% aqueous solution of sodium bicarbonate to achieve the corresponding [^11^C]carboxylates. Total synthesis time from adding HCl to Grignard to the reformulation was 15–20 min, achieving 98% and 96% RCY for [*1*-^11^C]butyrate and [*1*-^11^C]isobutyrate, respectively, with an average yield of 2812 MBq.^531^ Actually, [*1*-^11^C]butyric acid radiolabeling was first reported in 1943 by Buchanan *et al*. through [^11^C]CO_2_ fixation with Grignard reagent but without further experimental details.^554^ Similarly, [^11^C]CO_2_ was passed through a helium stream into a solution of propyl magnesium chloride in THF at 25°C, then quenched with water, hydrochloric acid, and NaOH solution subsequentially to obtain [*1*-^11^C]butyric acid in 40 min from the EOB to the final formulation. The isolated RCY ranged 31–50%, and the *A*_m_ was 7.4–37 GBq/μmol at EOB.^555^

#### 7.6.2 Preclinical Studies

**Figure 118:**
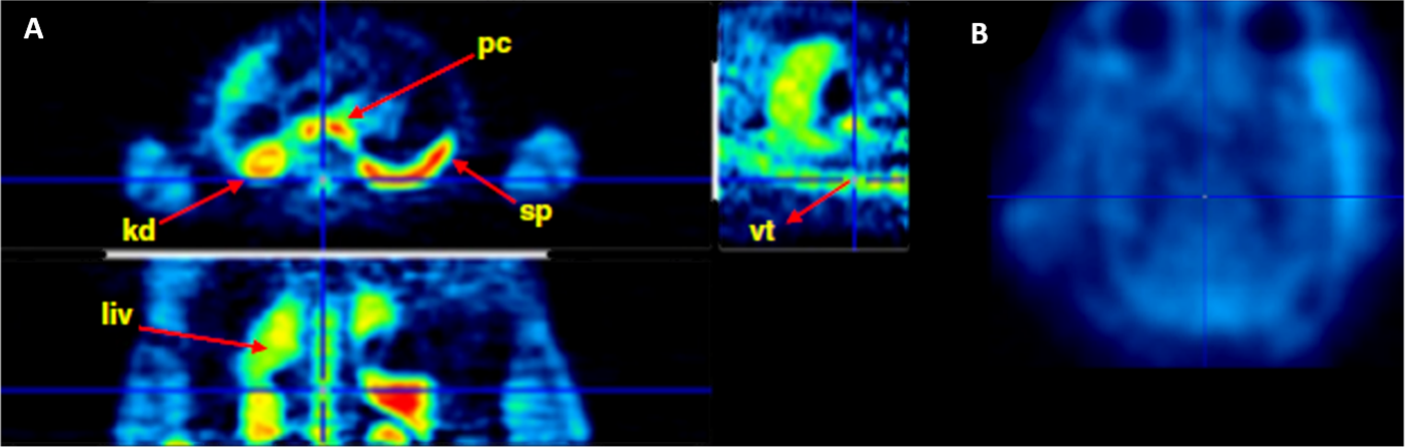
Thorax and abdomen (A) and brain (B) PET images of [^11^C]butyric acid (30–90 min p.i.). pc = pancreas, k = kidneys, sp = spleen, liv = liver, vt = vertebrae, and hrt = heart. Reproduced with permission from ref (555). Copyright 2013 Elsevier.

In dog iv, injection of [^11^C]butyrate and [^11^C]isobutyrate showed high uptake in the liver and abdomen within 2–4 min p.i., mimicking physiologic lipid storage site distribution. [^11^C]Isobutyrate activity in the heart gradually increased over time, reaching a homogenous whole-body distribution at 30 min.^531^ In white rats, [^11^C]butyric acid was administered along with glucose to evaluate liver glycogen formation. Radioactivity of expired [^11^C]CO_2_ demonstrated that it is metabolized within 30 min. Furthermore, 55% of the total radioactivity of liver glycogen was accounted for [^11^C]CO_2_ incorporation, indicating that [^11^C]butyrate should also be metabolized *via* the carbohydrate pathway.^554^ More recently, [^11^C]butyric acid was injected into six female baboons by radial vein, then scanned over 90 min using PET imaging. The plasma analysis showed rapid metabolism and fast clearance as the percentage of the unchanged [^11^C]butyric acid in plasma dropped to less than 20% within 5 min p.i. Biodistribution studies showed high [^11^C]butyric acid uptake in the pancreas, kidney, spleen, liver, and vertebrae and very low uptake in the heart (Figure 118). Very low activity was registered in the brain, which peaked at 10 min.^555^

#### 7.6.3 Clinical Studies

[^11^C]Butyrate was evaluated in 32 male patients (mean age 45 ± 8 yrs) with coronary artery disease and regional left ventricular dysfunction to assess the affected tissues’ oxidative metabolism. [^11^C]Butyrate was iv injected in fasted patients demonstrating fast elimination in myocardial segments with normal oxidative metabolism, whereas, in the ischemic region, an increased uptake was registered after 23–30 min. Furthermore, it allowed visualizing myocardial viability in several dysfunctional segments in the presence of oxidative events.^556^ Furthermore, [^11^C]butyrate iv injected in 24 postoperative patients with brain tumors (15 male and 9 female, mean age 41 ± 12 years) has allowed detection and differential diagnosis of benign and malignant brain tumors and monitoring efficacy of therapy.^556^ Lastly, a study in 27 patients (18 male and 9 female, mean age 41 ± 14 years) has resulted in a significant uptake in adenocarcinomas while benign lesions and chronic pancreatitis demonstrated significantly decreased uptake.^556^

### 7.7 Decanoic Acid

#### 7.7.1 Radiosynthesis

**Figure 119:**
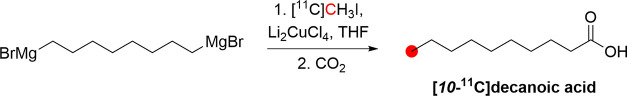
Radiosynthetic schemes of [*10*-^11^C]decanoic acid. ^11^C radionuclide position is highlighted in red.

A solution of *1*,*8*-bis-(bromomagnesium)octane in THF was added to [^11^C]CH_3_I trapped in a reaction vessel and Li_2_CuCl_4_ in THF. A stream of CO_2_ was further introduced, obtaining pure [*10*-^11^C]decanoic acid (Figure 119) after chromatography purification in 44 min with a RCY of 35%.^558^

### 7.8 Docosahexaenoic Acid

#### 7.8.1 Radiosynthesis

**Figure 120:**
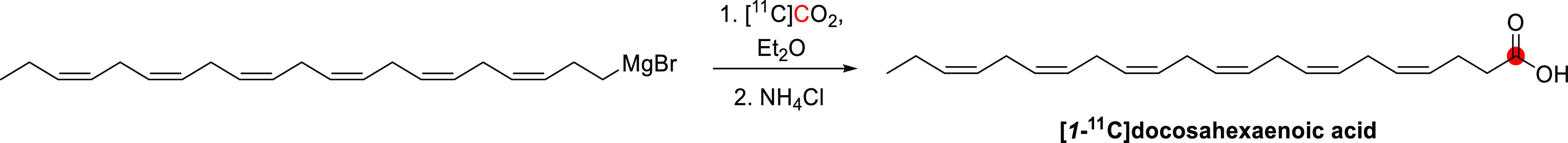
Radiosynthetic schemes of [1-^11^C]docosahexaenoic acid. ^11^C radionuclide position is highlighted in red.

To a solution of *1*-bromomagnesium heneicosa-*3*,*6*,*9*,*12*,*15*,*18*-hexaene in diethyl ether was delivered [^11^C]CO_2_ in helium. Unreacted gases were trapped on molecular sieves, and a solution of ammonium chloride was added to quench the reaction. After purification through the extraction column, pure [*1*-^11^C]docosahexaenoic acid (Figure 120) in less than 43 min with a RCY of 15.9–18.3% from EOS.^551^ It was further synthesized as described with a *A*_m_ > 18.5 GBq/μmol.^559^

#### 7.8.2 Preclinical Studies

**Figure 121:**
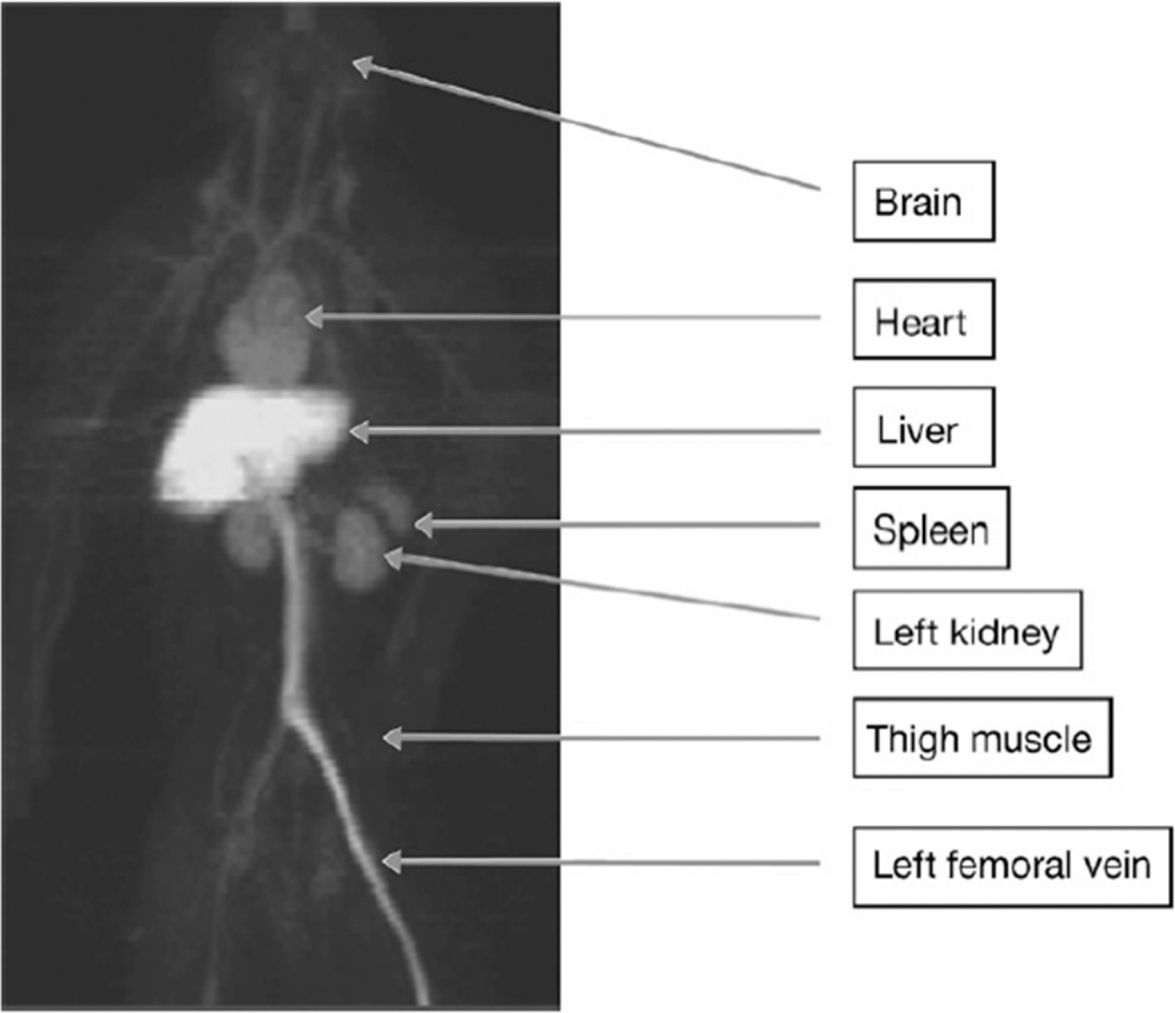
[*1*-^11^C]Docosahexaenoic acid whole-body summed retention in monkeys (0-6 min p.i.). Reproduced with permission from ref (559). Copyright 2008 American Society for Biochemistry and Molecular Biology. This work is licensed under a Creative Commons Attribution 4.0 International License (https://creativecommons.org/licenses/by/4.0/).

In monkeys [*1*-^11^C]docosahexaenoic acid was injected iv, and rapid uptake in the liver without apparent washout over 2 h was observed. No hepatobiliary excretion was registered, while elimination *via* urine was moderate. The highest uptake was observed in the liver, heart, kidney, gall bladder, spleen, and lungs. A small amount of activity was seen in the brain, accounting only for 0.5% of [*1*-^11^C]docosahexaenoic acid injected (Figure 121).^559^

#### 7.8.3 Clinical Studies

In healthy humans, [*1*-^11^C]docosahexaenoic acid demonstrated slow plasma metabolism: from 87% unchanged radiotracer 5 min p.i. to 35% after 60 min. The net incorporation rate of docosahexaenoic acid into the whole brain was equivalent to a consumption rate of 3.8 mg/day.^559,560^

### 7.9 Dodecanoic Acid

#### 7.9.1 Radiosynthesis

**Figure 122:**
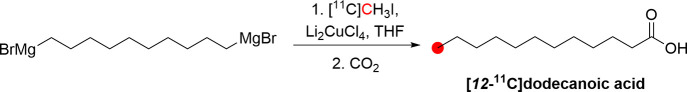
Synthesis of [*12*-^11^C]dodecanoic acid using [^11^C]CH_3_I. ^11^C radionuclide position is highlighted in red.

Dodecanoic acid has been labeled with ^11^C in the *1*- and *12*- positions. However, the chemistry for the labeling in position *1*- is not described. Regarding position *12*-, a solution of *1*,*10*-bis-(bromomagnesium)decane in THF was added to [^11^C]CH_3_I trapped in a reaction vessel and Li_2_CuCl_4_ in THF. A stream of CO_2_ was further introduced, obtaining pure [*12*-^11^C]dodecanoic acid (Figure 122) after chromatography purification in 45 min with a RCY of 28%.^558^

#### 7.9.2 Preclinical Studies

In pigs, [*12*-^11^C]dodecanoic acid was used to evaluate myocardial energy metabolism due to its involvement in the TCA cycle and *β*-oxidation processes. It was rapidly cleared from the blood: at 1 min after iv injection, only 10% of radioactivity was found in arterial blood. [*1*-^11^C]Dodecanoic acid shows slower wash-out kinetics compared to [*12*-^11^C]dodecanoic acid due to the different metabolic pathways. The myocardial concentration was around 10 times the amount of tracer administered per gram of body weight, irrespective of labeling position. These tracers’ kinetics are directly related to cardiac workload and indistinguishable from that of [^11^C]acetate.^247^ Due to the rapid clearance of the tracer, 90% of the uptake occurs in the first 2 min p.i.^561^

### 7.10 Formic Acid

#### 7.10.1 Radiosynthesis

**Figure 123:**
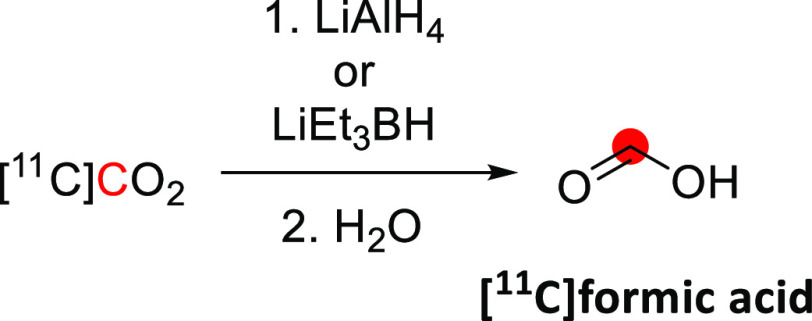
Radiosynthesis of [^11^C]formic acid. ^11^C radionuclide position is highlighted in red.

[^11^C]Formic acid was first synthesized in 1942 as an intermediate in [^11^C]CH_3_I synthesis by enzymatic reduction of [^11^C]CO_2_ using formate dehydrogenase present in a suspension of *Escherichia coli* bacteria.^618^ [^11^C]Formic acid synthesis did not receive further attention until 2001 when Roeda *et al*.^507^ investigated the product distribution obtained from the reaction of [^11^C]CO_2_ with lithium aluminum hydride at varying temperatures (Figure 123).^507^ This reaction, commonly used to make [^11^C]CH_3_OH as an intermediate in “wet method” [^11^C]CH_3_I synthesis,^619^ was found to produce significant amounts of [^11^C]formic acid, especially at low temperatures. Between −50°C and −20°C, [^11^C]formic acid was formed immediately in a RCY of 80–90%, with the remainder of the activity present as [^11^C]formaldehyde (<20%) and [^11^C]CH_3_OH (<5%).^39,507^ At −10 °C and above, the [^11^C]CH_3_OH yields increase with increasing temperature at the expense of [^11^C]formic acid, while [^11^C]CH_2_O levels increase to ∼20% and remain almost constant. Longer reaction times did not have a significant effect on the product distribution.

To further improve the yield of [^11^C]formic acid, Roeda *et al*. found that using the weaker reducing agent LiEt_3_BH succeeded in converting [^11^C]CO_2_ to [^11^C]formic acid virtually quantitatively at −10 °C.^507,508^ This reaction proceeded instantaneously to produce [^11^C]formate in a RCY >98% and *A*_m_ of 9 GBq/μmol (corrected to EOB). This reagent has been utilized as a ^11^C-formulating agent,^509−511^ and as a precursor to radiolabeling reagents [^11^C]CO and [^11^C]CH_3_OH.^38^

### 7.11 Hexanoic Acid

#### 7.11.1 Radiosynthesis

**Figure 124:**
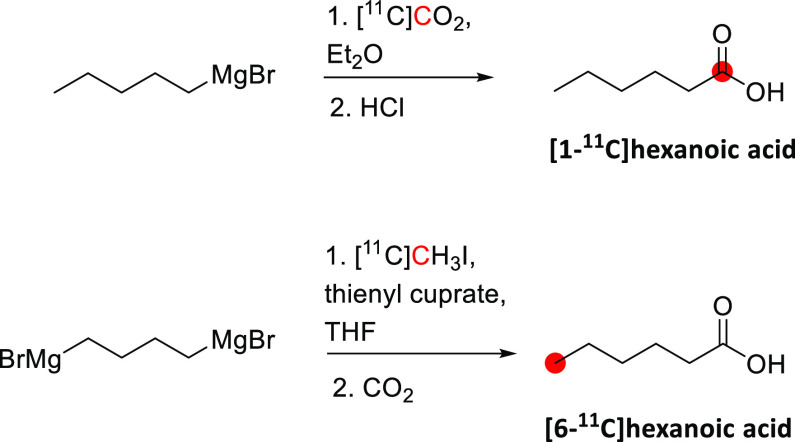
Synthesis of [^11^C]hexanoic acid using [^11^C]CO_2_ or [^11^C]CH_3_I. ^11^C radionuclide position is highlighted in red.

[*1*-^11^C]Hexanoic acid was prepared from [^11^C]carboxylation of pentylmagnesium bromide 2M in diethyl ether at −40 °C, following later acidification with HCl and a final liquid extraction with diethyl ether. Subsequent evaporation and dissolution into a saline solution made [*1*-^11^C]hexanoic acid ready for a biological test. In this way, it was obtained with 28% RCY as a sodium salt.^514^ An automated procedure exploiting final cartridge purification instead of liquid extraction and using 1M solution of pentylmagnesium bromide in THF at 5–12 °C achieved a RCY of 71% within 15 min from the end of irradiation.^563^ [*6*-^11^C]Hexanoic acid (Figure 124) was obtained from the bis-Grignard reagent in a two-step procedure: [^11^C]methylation in THF using thienyl cuprate at −72 °C and the following carbonation with CO_2_. After evaporation and column purification, [*6*-^11^C]hexanoic acid was obtained with a RCY of 36% in 47 min from EOB.^558^

#### 7.11.2 Preclinical Studies

**Figure 125:**
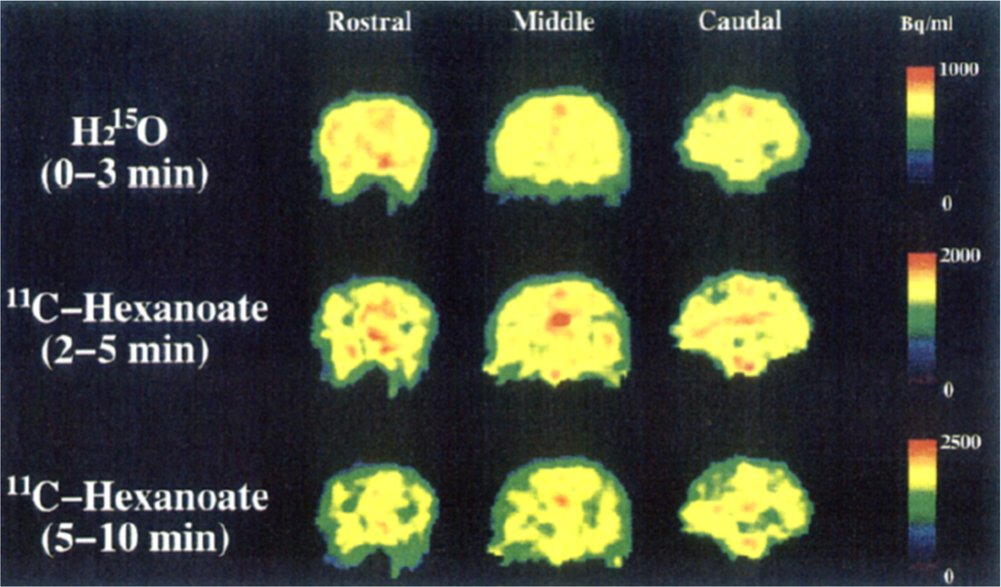
PET images of cat brain acquired with [^15^O]labeled water, [*1*-^11^C]hexanoate (within 2 and 5 min), and [*1*-^11^C]hexanoate (within 5 and 10 min). Reproduced with permission from ref (562). Copyright 1996 Springer Nature.

In mice after iv injection, [*1*-^11^C]hexanoic acid was mainly uptake in kidneys (6.14%ID/g at 5 min), followed by spleen, pancreas, lungs, liver, brain, and heart. It had a fast washout in all organs except the pancreas (3.51%ID/g at 90 min). A high percentage of tracer was excreted into urine (4.0%ID/g at 90 min). In the heart and plasma, there was a peak immediately after injection and a fast decrease in radioactivity. [*1*-^11^C]Hexanoic acid was more retained in the brain with a brain-to-plasma ratio increasing for the first 5 min, constant until 30 min, and then decreased.^514^ At 3 min post iv injection in the brain, there was almost no traces of unmetabolized [*1*-^11^C]hexanoic acid, with 9% of [^11^C]CO_2_/HCO_3_^–^ and a high percentage of glutamate/glutamine metabolites.^513^ In starvation/fasting conditions, a higher uptake in both brain and heart was registered.^513,514^ In cat brain, an increasing uptake in the brain of [*1*-^11^C]hexanoic acid was registered in the first 2 min, followed by a fast decrease and a second peak at 7–10 min after iv injection with a final gradual washout (Figure 125). At the same time, in blood at 5 min, there was almost no parent radiotracer present, but only [^11^C]CO_2_/HCO_3_^–^. These findings suggest that the second phase of brain activity uptake could be due to the [^11^C]CO_2_/HCO_3_^–^ from the periphery.^562^ In dogs, [*1*-^11^C]hexanoate showed significant uptake in the liver, kidney, and abdomen within 3 min p.i., followed by a homogenous whole-body distribution after 1 h.^531^

### 7.12 Linoleic Acid

#### 7.12.1 Radiosynthesis

**Figure 126:**
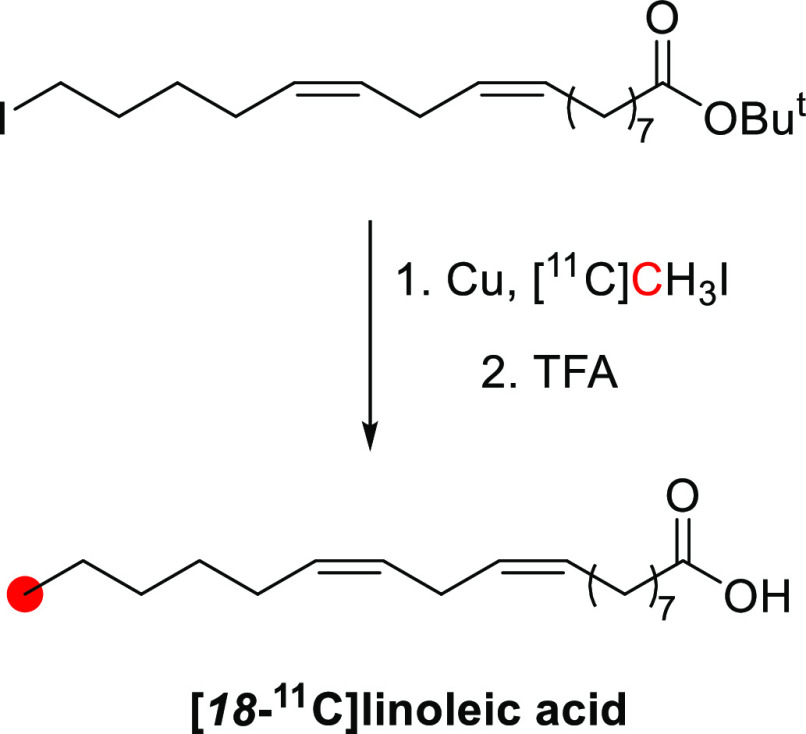
Radiosynthetic scheme of [*18*-^11^C]linoleic acid. ^11^C radionuclide position is highlighted in red.

Radiosynthesis of [*18*-^11^C]linoleic acid was performed *via* a coupling reaction between unsaturated *17*-iodo heptadecanoic acid with carboxylic function protected as a *tert*-butyl ester (Figure 126). It was treated with copper complex as the coupling agent and [^11^C]CH_3_I, with final cleavage of acid function using trifluoroacetate. [*18*-^11^C]Linoleic acid was obtained with 36–48% isolated RCY, *A*_m_ ∼20 GBq/μmol within 45 min from EOB.^564^

### 7.13 Octanoic Acid

#### 7.13.1 Radiosynthesis

**Figure 127:**
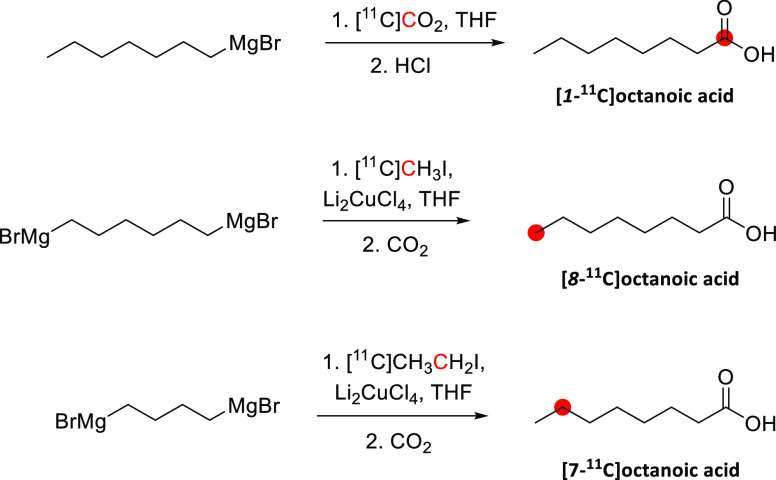
Radiosynthetic schemes of [^11^C]octanoic acid using [^11^C]CO_2_, [^11^C]CH_3_I, or [*1*-^11^C]CH_3_CH_2_I. ^11^C radionuclide position is highlighted in red.

[*1*-^11^C]Octanoic acid (Figure 127) was obtained from carboxylation of a Grignard reagent (heptyl magnesium bromide) in THF and the following acidification with HCl. Final purification through liquid extraction with ether and reformulation with saline made it feasible for further biological evaluation.^620^ Automated procedures with an HPLC purification instead of liquid extraction were developed, achieving pure [*1*-^11^C]octanoic acid within 40 min from EOB.^621,622^ Purification with cation-exchange resin allows achieving a RCY of 68% within 15 min.^563^ Modifications, such as using ether as solvent at 35°C or exploiting a tube-based method produced [*1*-^11^C]octanoic acid with a RCY of 64% within 35 min and 37% in 30 min, respectively.^558^

[*8*-^11^C]Octanoic acid and [*7*-^11^C]octanoic acid were achieved after [^11^C]alkylation of the appropriate bis-Grignard reagent (Figure 127) in THF using dilithium tetrachlorocuprate as the catalyst and final carbonation using CO_2_. [*8*-^11^C]Octanoic acid and [*7*-^11^C]octanoic acid were obtained within 44 and 52 min from EOB with 32% and 23% RCY, respectively.^558^

#### 7.13.2 Preclinical Studies

**Figure 128:**
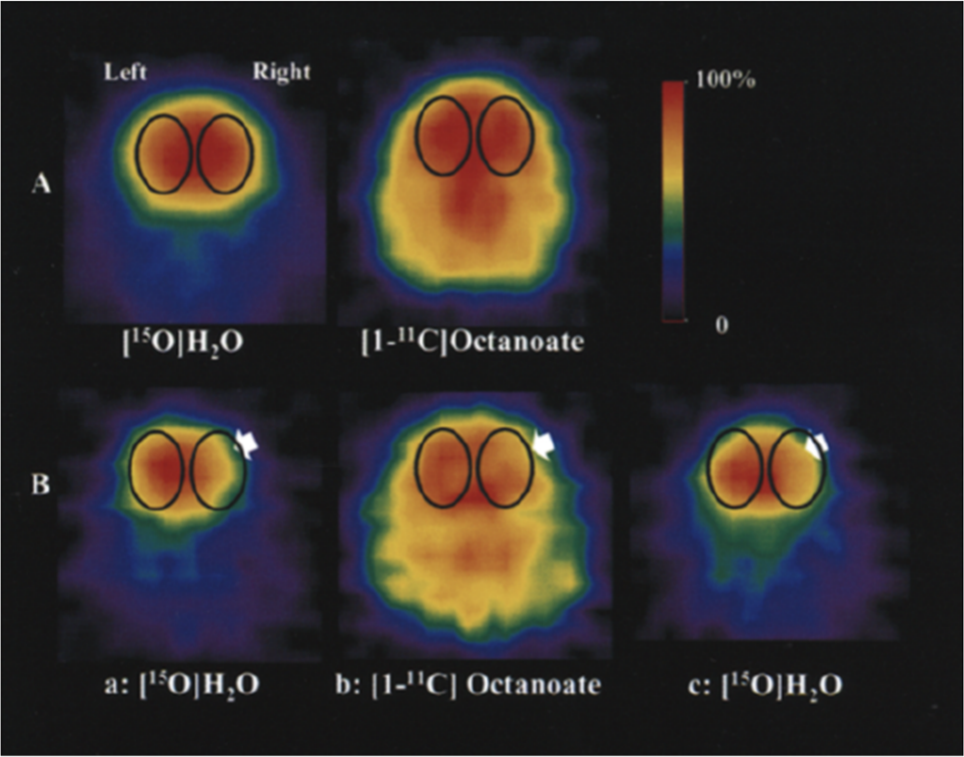
Radioactivity retention and reactive oxygen intermediates of [^15^O]H_2_O (0–2 min) and [*1*-^11^C]octanoate (5–15 min) in rats brain with (B) or without (A) focal cerebral ischemia recorded 1–2 h (a), 3–4 h (b), and 5–6 h (c) after middle cerebral artery occlusion. Arrows show reduced radioactivity in the lesioned (right) hemisphere. Reproduced with permission from ref (570). Copyright 2000 Springer Nature.

In rats, 60 min after [*1*-^11^C]octanoic acid iv injection, high radioactivity was detected in the liver, kidneys, harderian glands, and submaxillary glands. Lower activity was detected in the brain.^566^ In cats, activity was rapidly cleared from the blood, while in the brain, there was a transient peak immediately after injection and a second peak 5–10 min later with a gradual decline. In cats, the highest radioactivity was registered in submaxillary glands.^566^ In healthy mice, [*1*-^11^C]octanoic acid was highly retained in kidneys with a fast washout, followed by heart, lung, blood, and liver. In the liver, it was mainly taken up in parenchymal cells (98% of total liver activity 5 min p.i.), and most of the radioactivity was extracted in an aqueous layer, suggesting metabolization through β-oxidation instead of more lipophilic esterification.^565^ In rats, [*1*-^11^C]octanoic acid rapidly entered the brain, followed by a slow washout, with a brain-to-blood ratio increasing over time. In the brain, it is metabolically trapped, possibly as glutamine or glutamate. At 0.5 min p.i., only 8% parent compound remained in the blood (Figure 128).^567^ In pigs, [*1*-^11^C]octanoic acid was used to evaluate its fractional oxidation and metabolic rate compared to longer fatty acids, showing a comparatively low concentration in the blood.^561^ In dogs, [*1*-^11^C]octanoate showed marked activity in the liver and abdomen within 4 min, with initial and prolonged retention in the lungs, probably due to trapping in lipoprotein membranes of the alveolar capillary.^531^

#### 7.13.3 Clinical Studies

Only one preliminary PET study in a healthy volunteer was performed, revealing a clear image of the liver with a rapid hepatic clearance in two phases.^565^ [*1*-^11^C]Octanoic acid was a potential imaging agent for cerebral ischemia in rat and canine models and to evaluate the pathophysiology of thromboembolic stroke in a canine model. At the same time, it was determined not to be useful for imaging myocardial ischemia in dogs.^568−570^

### 7.14 Oleic and Stearic Acid

#### 7.14.1 Radiosynthesis

**Figure 129:**
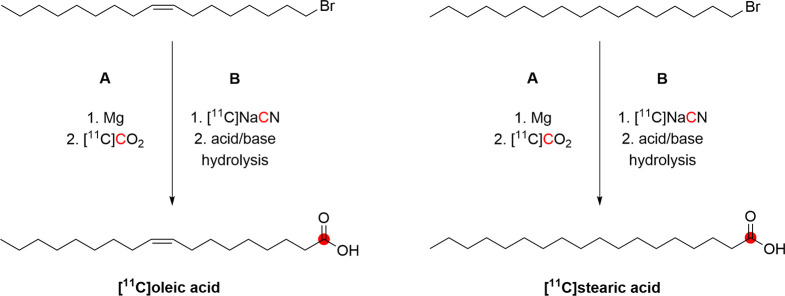
Synthesis of [^11^C]oleic acid and [^11^C]stearic acid using [^11^C]CO_2_ or [^11^C]NaCN. ^11^C radionuclide position is highlighted in red.

In 1975, Poe *et al*. radiolabeled stearic acid and oleic acid at the ^11^C-carboxyl position by reacting the appropriate long-chain Grignard reagent with [^11^C]CO_2_ (Figure 129A).^519^ In this process, [^11^C]CO_2_ was bubbled through the Grignard reagent for 20 min until radioactive equilibrium was reached, producing between 70–260 MBq of the ^11^C-fatty acid. Purification techniques and product purities were not discussed.

An alternative radiosynthetic route to ^11^C-fatty acids that does not require Grignard reagents was described by Takahashi *et al*. in 1990.^571^ Utilizing [^11^C]NaCN as the radiolabeling reagent, alkyl bromides were converted to the corresponding ^11^C-alkyl nitrile, which was then hydrolyzed using acid or base to produce the ^11^C-fatty acid (Figure 129B). Purification was performed using a C18 Sep-Pak cartridge. In a process lasting about 50 min, [^11^C]oleic acid and [^11^C]stearic acid were obtained with a RCY of 70–83% (based on [^11^C]HCN) and RCP of 93–98%.

#### 7.14.2 Preclinical Studies

To evaluate the potential of radiolabeled fatty acids to measure myocardial blood flow *in vivo*, Poe *et al*. performed studies in anesthetized dogs comparing the behavior of ^11^C-labelled stearic acid and oleic acid with their ^131^I-iodinated analogues.^518,519^

Following direct tracer injection into the coronary artery, the radioactivity of the heart was measured using a scintillation detector, enabling the myocardial retention of each fatty acid to be estimated. Similar myocardial retentions were observed for [^11^C]stearic acid, [^11^C]oleic acid, and the terminally iodinated hexadecenoic acid. The radiotracers were found to clear quickly from the blood, with <20% remaining at 10 min p.i.^518^

### 7.15 Palmitic Acid

#### 7.15.1 Radiosynthesis

[*1*-^11^C]Palmitic acid was produced from *n*-pentadecyl magnesium bromide and [^11^C]CO_2_ in diethyl ether. Adding acid to the solution, the Grignard reagent in excess was quenched, and [*1*-^11^C]palmitic acid obtained was solubilized with serum albumin (solution 4% in saline), providing [*1*-^11^C]palmitic acid in injectable form for the biological test. The entire procedure took 30 min from the carbonation and produced 400–700 MBq of [*1*-^11^C]palmitic acid starting from 7.4 GBq of [^11^C]CO_2_.^584,623^ Further optimizations of this radiosynthetic procedure allowed increased isolated RCY, Am, and reaction time.^576,577,624−626^ A remote semiautomated chemical process system developed by Padgett *et al*. with a more accessible product purification, through column chromatography on acetic acid-treated neutral alumina oxide, in 30 min synthesis time obtained [*1*-^11^C]palmitic acid with *A*_m_ higher than 0.259 GBq/μmol.^625^ A captive solvent method has been exploited using microporous propylene powder or alumina cartridges to obtain [*1*-^11^C]palmitic acid until 74% isolated RCY.^624^ Furthermore, an automated SPE-based radiosynthesis achieved [*1*-^11^C]palmitic acid in 33% isolated RCY within 10 min from [^11^C]CO_2_ trapping, which increased in a solid-based method obtaining [*1*-^11^C]palmitic acid in 8 min from EOB, 38% isolated RCY, and *A*_m_ of 9.25–12.95 GBq/μmol and 50% isolated RCY in 15 min using column extraction method.^576−578^ Recently, Amor-Coarasa *et al*. developed a 3D-printed automated synthesis unit able to produce [*1*-^11^C]palmitic acid with 57% isolated RCY within 6.5 min.^626^

[*1*-^11^C]Palmitic acid was also synthesized through [^11^C]HCN addition to precursor *n*-pentadecyl magnesium bromide with potassium hydroxide in DMSO and final acidic hydrolysis, achieving [*1*-^11^C]palmitic acid in 78% isolated RCY within 58 min from EOB.^315^

**Figure 130:**
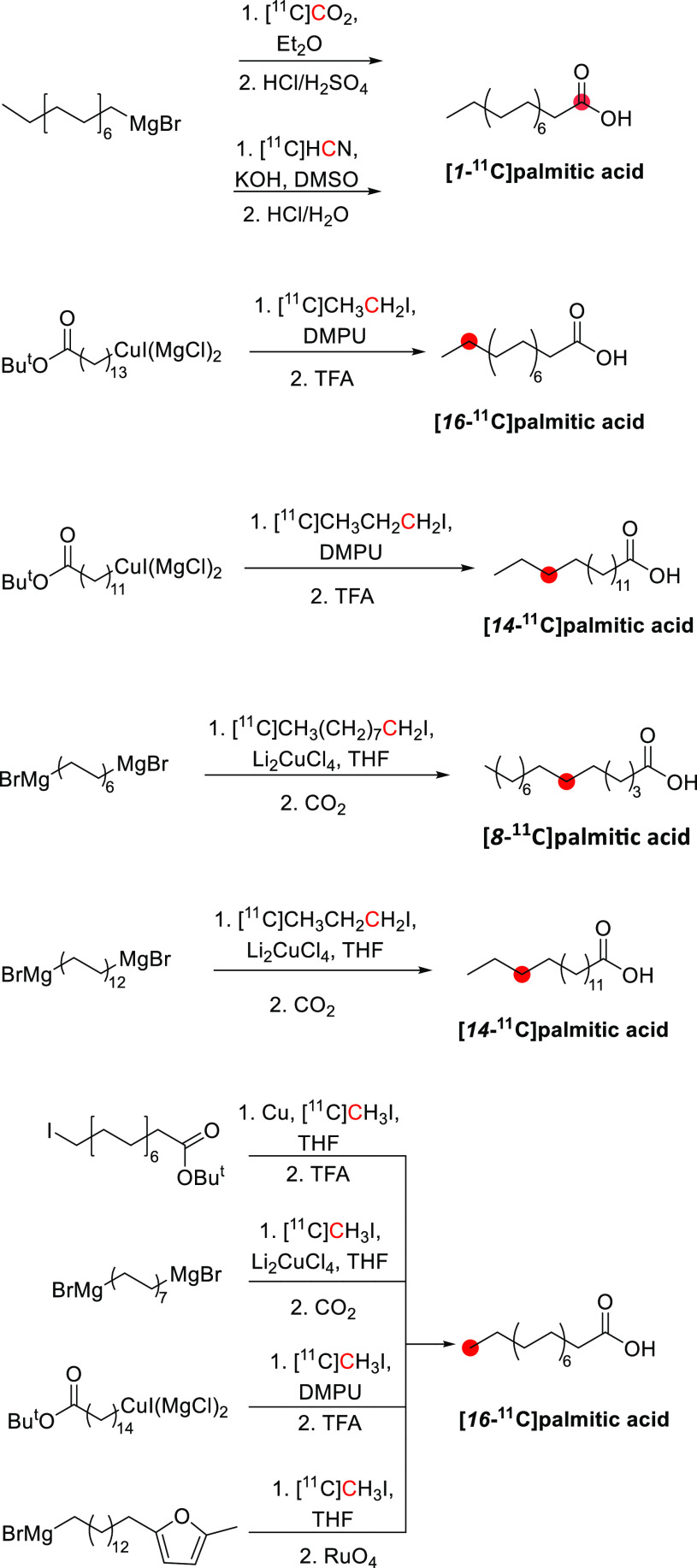
Radiosynthetic schemes of [^11^C]palmitic acid. ^11^C radionuclide position is highlighted in red.

[*1*-^11^C]Palmitic acid, [*14*-^11^C]palmitic acid, and [*16*-^11^C]palmitic acid were synthesized through a cross-coupling reaction between Bu^t^-protected ω-iodo fatty acid precursors, properly converted in a copper–zinc reagent as labeling precursor (Figure 130). The final deprotection with TFA gives radiolabeled derivatives with free carboxylic functions. RCY of 6% within 30 min from EOB and RCP 88% were achieved.^627^

[*16*-^11^C]Palmitic acid was also radiosynthesized starting from Bu^t^-protected ω-iodopentanoic acid with copper complex and [^11^C]CH_3_I in THF with 73% isolated RCY within 46 min from the end of radionuclide production.^557^ Similar strategy but with starting alkylfuran Grignard precursor and final oxidative cleavage with ruthenium tetraoxide lead to 5 in 75 min of total synthesis time, purified through a resin-packed column, and incubated with human serum albumin to obtain an injectable solution.^572^

[*8*-^11^C]Palmitic acid, [*14*-^11^C]palmitic acid, and [16-^11^C]palmitic acid were also synthesized from the proper bis-Grignard precursors reacting with [^11^C]alkyl iodide with copper catalyst and final-stage carboxylation in THF with RCY of 12–22% in 45–65 min.^558^

Because the [^11^C]palmitic acid has very poor water solubility, the preparation for injection requires the radiopharmaceutical to be water-solubilized by binding to human serum albumin. Consequently, the purification and formulation of [*1*-^11^C]palmitate compared to [^11^C]acetate is more time-consuming and challenging, producing lower yields. Despite technological advances and optimizations, developing a reliable, fully automated [*1*-^11^C]palmitic acid procedure is challenging. Alejandro Amor-Corasa *et al*. recently developed and reported the lightweight (4 kg) and fully automated 3D-printed cassette-based synthesis unit for ^11^C-labeled fatty acid production,^626^ giving [^11^C]palmitic acid with a RCYs 57.2 ± 12.4% to the EOS within 10 min.^576,626^

#### 7.15.2 Preclinical Studies

**Figure 131:**
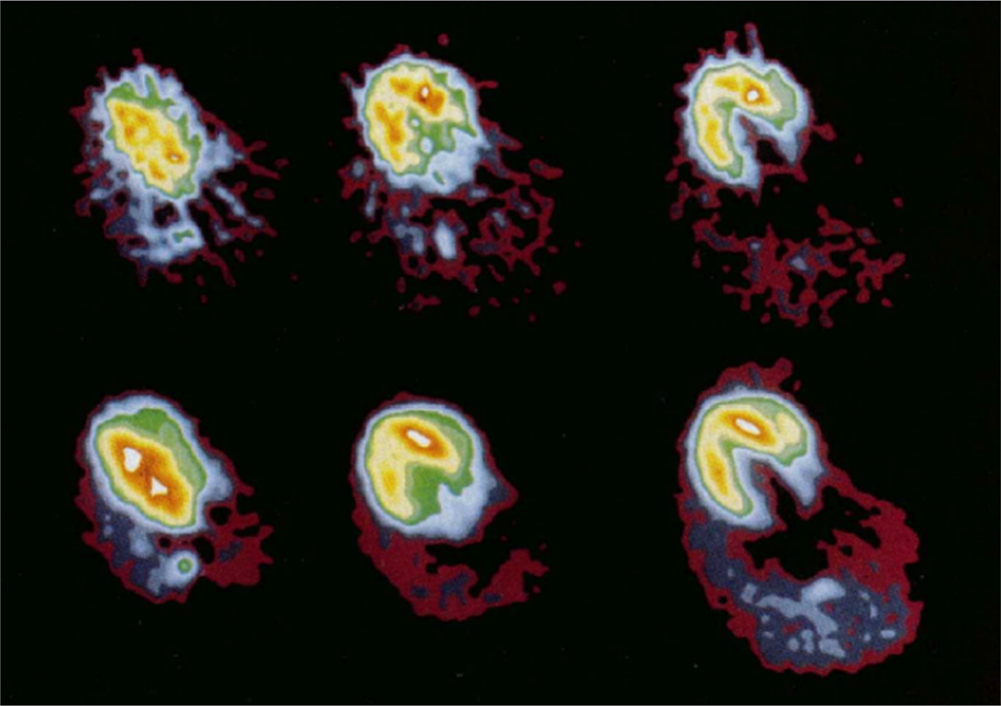
[*16*-^11^C]Palmitic acid (top) and [*1*-^11^C]palmitic acid (bottom) midventricular PET images of a dog heart. Reproduced with permission from ref (572). Copyright 1994 American Chemical Society.

[*1*-^11^C]Palmitic acid and [*16*-^11^C]palmitic acid showed different biodistribution in rats iv administered: [*16*-^11^C]Palmitic acid exerted around 50% higher uptake in the heart, probably due to the egress of [^11^C]CO_2_ from the heart after [*1*-^11^C]palmitic acid metabolic oxidation. They exerted high uptake in the liver and low absorption in blood, lung, kidney, and muscle. At the same time, they showed similar biodistribution and uptake in dogs’ hearts.^572^ [*1*-^11^C]Palmitic acid was further evaluated as a PET radiotracer for myocardial fatty acid metabolism in normal and increased metabolic demand and acute myocardial ischemia in dogs (Figure 131),^573^ in liver fatty oxidation in pigs.^573^ In monkeys, its incorporation rate was almost double in the temporal and parietal cortex compared to white matter.^575^ The primary metabolite of [*1*-^11^C]palmitic acid was considered [^11^C]CO_2_ due to peripheral oxidation of fatty acids, as confirmed by the integrated plasma [^11^C]CO_2_ increasing after [*1*-^11^C]palmitic acid administration. The latter was confirmed in a methyl palmoxirate-treated group (mitochondrial fatty acid β-oxidation blocker), where integrated plasma [^11^C]CO_2_ was around 50% less than control.^575^ In pigs, [*1*-^11^C]palmitic acid caused increased radioactivity in the heart, liver, spleen, and kidneys, with almost no urine excretion.^574^ Furthermore, in pigs, [*1*-^11^C]palmitic acid was used to assess oxidative fatty acids consumption. It showed a low clearance rate from blood paired with a low transfer rate constant, with its oxidative utilization strongly decreased by inhibiting fatty acid oxidation.^561^

#### 7.15.3 Clinical Studies

In healthy patients, [*1*-^11^C]palmitic acid showed a biological half-life of around 20 min and mainly accumulated in the liver and heart, as seen in preclinical studies. No radiotracer was detected in urine, suggesting that all [*1*-^11^C]palmitic acid bound to albumin and was metabolized as esterified fatty acid (*e.g*., triglycerides) or oxidized: this was confirmed by the increased radioactivity registered in CO_2_ and triglycerides fraction with a reduction of ^11^C radioactivity in free fatty acid fraction after bolus injection, confirming that [*1*-^11^C]palmitic acid also underwent to a liver re-esterification and not only metabolic oxidation.^574^ [*1*-^11^C]Palmitic acid uptake and biodistribution were evaluated in patients with heart-related diseases due to impairment of fatty acid oxidation and the resulting ROS overexpression in these pathologies. Particularly, PET studies with [*1*-^11^C]palmitic acid in cardiomyopathy, ischemic heart disease, and myocardial infarction patients could help to assess the pathological mechanisms involved and evaluate the efficacy of proper therapeutic interventions.^574^

### 7.16 Pentanoic Acid

#### 7.16.1 Radiosynthesis

**Figure 132:**
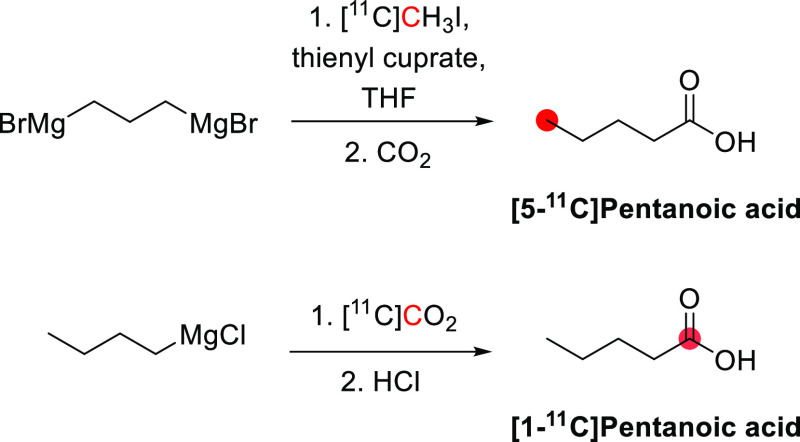
Radiosynthetic schemes of [^11^C]pentanoic acids. ^11^C radionuclide position is highlighted in red.

A solution of thienyl cuprate and *1*,*3*-bis(bromomagnesium)propane in THF was added to [^11^C]CH_3_I trapped in a reaction vessel. A stream of CO_2_ was further introduced, obtaining pure [*5*-^11^C]pentanoic acid (Figure 132) after chromatography purification in 47 min with a RCY of 27%.^558^ [*1*-^11^C]Pentanoic acid was obtained from [^11^C]CO_2_ fixation to the respective Grignard reagent with the following acidic workup in 15–20 min overall preparation, albeit it was further tested as [*1*-^11^C]pentanoate sodium salt. It was achieved with a RCY of 59%, with an average of 2.812 GBq.^531^

#### 7.16.2 Preclinical Studies

In dogs, [*1*-^11^C] pentanoate activity was concentrated in the kidney and liver, mainly excreted in bile and concentrated in the gallbladder.^531^

### 7.17 Propanoic Acid

#### 7.17.1 Radiosynthesis

**Figure 133:**
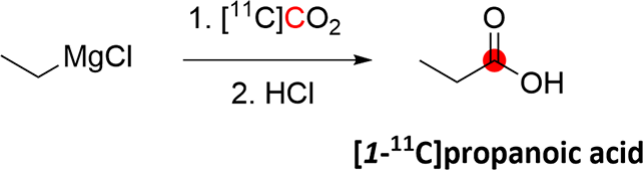
Radiosynthesis of [*1*-^11^C]propanoic acid from CH_3_CH_2_MgBr and [^11^C]CO_2_. ^11^C radionuclide position is highlighted in red.

[*1*-^11^C]Propanoic acid was firstly synthesized by Fang *et al*. with a RCY of 99% within 30 min. However, [*1*-^11^C]propanoic acid was obtained through reaction with the proper Grignard reagent (*i.e*., ethyl magnesium chloride) and [^11^C]CO_2_ followed by an acidic workup to achieve it in neutral form (Figure 133). The total preparation time was 15–20 min with a RCY of 98%.^531^ [*1*-^11^C]Propanoic acid has also been synthesized during the development of a method for the preparation of [*1*-^11^C]propyl iodide from [^11^C]CO and its use in alkylation reactions. In this case, [*1*-^11^C]propanoic acid was prepared by a palladium-mediated formylation of ethene with [^11^C]CO within 3 min.^579^ The total preparation time was 15–20 min with a RCY of 98% and an average of 2812 MBq obtained.^531^

#### 7.17.2 Preclinical Studies

In dogs, [*1*-^11^C]propanoate revealed high uptake in the cardiovascular system and abdomen within 3--6 min, followed by a homogenous whole-body biodistribution after 30 min. Particularly, the accumulation in the liver and diffusely in the abdomen suggested that it equilibrates with lipid storage sites with high perfusion rates.^531^

### 7.18 Tetradecanoic Acid

#### 7.18.1 Radiosynthesis

**Figure 134:**
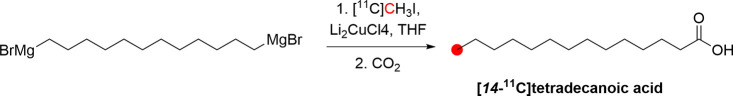
Radiosynthetic schemes of [*14*-^11^C]tetradecanoic acid. ^11^C radionuclide position is highlighted in red.

A solution of *1*,*12*-bis-(bromomagnesium)dodecane in THF was added to [^11^C]CH_3_I trapped in a reaction vessel and Li_2_CuCl_4_ in THF. A CO_2_ stream was introduced, obtaining pure [*14*-^11^C]tetradecanoic acid (Figure 134) after chromatography purification in 45 min with RCY of 23%.^558^

#### 7.18.2 Preclinical Studies

In pigs, [*14*-^11^C]tetradecanoic acid has been used to investigate the oxidative utilization of fatty acids. The study showed a relatively fast blood clearance, rational oxidative utilization of around 82%, and a distribution volume of 25%. Furthermore, the transfer rate constants of tracer from blood to myocardium and the myocardial uptake were reduced by oxfenicine, an inhibitor of long-chain fatty acid oxidation.^561^

### 7.19 *β*-Hydroxybutyrate

#### 7.19.1 Radiosynthesis

**Figure 135:**
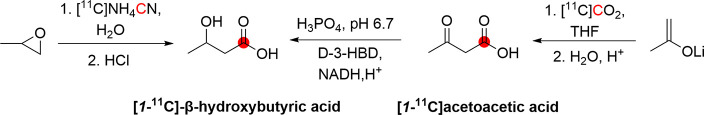
Synthesis of [*1*-^11^C]-*β*-hydroxybutyric acid using [^11^C]NH_4_CN or [^11^C]CO_2_. ^11^C radionuclide position is highlighted in red.

Propylene oxide was reacted with [^11^C]ammonium cyanide for 10 min at 40 °C, followed by acid hydrolysis for 5 min at room temperature (Figure 135). After HPLC purification, [*1*-^11^C]-*β*-hydroxybutyric acid was obtained in 45–50 min from the end of trapping with a RCY of 20–30%.^582,583^ A stereospecific synthesis was also developed, obtaining the final product with >87% enantiomeric excess, using only HCl in the hydrolysis step.^583^

Another reported synthesis procedure started from radiolabeled acetoacetate, obtained with the same procedure previously reported, and final enzymatic conversion to [*1*-^11^C]-*β*-hydroxybutyric acid using (*d*)-*β*-hydroxybutyrate dehydrogenase (*d*-*3*-HBD). The optimized procedure was performed at pH 6.7 with phosphoric acid in the presence of nicotinamide adenine dinucleotide with purification by ion exchange column produced [*1*-^11^C]-*β*-hydroxybutyric acid in 36 min with 10% RCY.^581^

#### 7.19.2 Clinical Studies

[*1*-^11^C]-*β*-Hydroxybutyric acid was assessed in healthy volunteers to determine the regional cerebral uptake of ketone bodies.^580^ The concentration of unmetabolized tracer in the brain was very low, indicating that the BBB passage was the rate-limiting step in ketone body utilization. The utilization rate increased linearly with plasma concentration, and it was higher in grey than white matter.^580^

## 8 Hormones and Neurotransmitters

Hormones and neurotransmitters are fundamental chemical messengers in maintaining homeostasis in the human body. Their function enables the communication between cells, which selectively respond to a signal by interaction with surface receptors. Whilst neurotransmitters act between neighboring neurons, hormones are released in the bloodstream and interact with cells distant from the hormone release site. The radiolabeling of hormones and neurotransmitters with carbon-11 allows for studying these signaling molecules’ physiological and pathological functions and sometimes monitoring the activity and expression of the dedicated receptors (Table 7).

**Table 7:** Carbon-11 Labeled hormones and neurotransmitters

compd		radiolabeling position	preclinical and clinical studies	synthon	*A*_M_ (GBq/μmol)	RCY	total time (min)	ref
dopamine		*1*-	dogs,^663^ monkeys^664^	[^11^C]HCN	nr	30%	65	(663)
				[^11^C]CH_3_NO_2_	37	20%	45	(664)

epinephrine		*N*-*methyl*-	rabbit,^665^*pigs,^666^* humans^637^	[^11^C]CH_3_I	nr^a^	10%	35	(667)
				[^11^C]CH_3_OTf	nr	25%	35	(667)
				*l*-[*methyl*-^11^C]methionine	7.4	20%	30	(665)

GABA		*4*-	nr	[^11^C]HCN	4	65%	40	(638)

l-DOPA		*3*-	rats,^668,669^ monkeys,^635,670−672^ humans^673−677^	[*3*-^11^C]alanine	0.4	30%	50	(678)
		*carbonyl*-	humans^679^	[1-^11^C]alanine	2.5	60%	50	(678)

melatonin		*1*-	humans^679^	[^11^C]CH_3_COCl	3.49	13%	35	(650)
				[^11^C]CH_3_COOH	155	12%	45	(680)
				[^11^C]CO_2_	100	36%	2^**^	(681)

norepinephrine		1-	dogs,^682^ monkeys^642,683^	[^11^C]NaCN	nr	25%	40	(684)
				[^11^C]CH_3_NO_2_	56	25%	70	(685)

octopamine	*m*-	*1*-	nr	[^11^C]HCN	nr	2.3%	60	(686,687)
	*p*-	*1*-	nr	[^11^C]HCN	nr	2.3%	60	(686,687)
				[^11^C]CH_3_NO_2_	56	25%	60	(685)

progesterone		*21*-	nr	[^11^C]CH_3_I	0.74	13%	60	(688)
				[^11^C]CH_3_(*2*-thienyl)Cu	14	35%	nr	(689)

serotonin		*1*-	rabbits,^690^ dogs,^690^ humans^691^	[^11^C]KCN	5.1	13%	78	(692)
tyramine		*1*-	nr	[^11^C]CH_3_NO_2_	37	20%	45	(664)

a nr: not reported

Dopamine is a fundamental neurotransmitter for the healthy functioning of the human brain. It involves several key functions, such as reward mechanisms, motor control, learning, and emotions.^628^ Dopamine also fulfills other vital functions outside of the brain.^629,630^*l*-*3*,*4*-Dihydroxyphenylalanine (l-DOPA) is the precursor of the neurotransmitter dopamine *via* interaction with the enzyme l-amino acid decarboxylase.^631^ Growing evidence suggests that the role of *l*-DOPA is not only as a metabolic precursor but also as a neurotransmitter and neuromodulator.^631,632^*l*-[^11^C]DOPA has also been used to investigate dopaminergic metabolism^632,633^ and study the behavior of *6*-[^18^F]fluoro-*l*-DOPA, which proved to be quite different in crucial aspects.^634,635^

The use of carbon-11 labeled epinephrine was suggested as a tool to evaluate the functional integrity of the cardiac sympathetic nervous system and detect cardiac diseases. Epinephrine is the main signaling agent of the sympathetic nervous system alongside its demethylated analogue norepinephrine. The biosynthesis of epinephrine mainly occurs in the adrenal medulla and the medulla oblongata from norepinephrine by the enzyme phenylethanolamine *N*-methyltransferase.^630^ Its action mainly activates the fight-or-flight response by binding to the α and β adrenergic receptors.^630,636,637^

It was suggested that PET studies using ^11^C-labeled *γ*-aminobutyric acid (GABA) would give a deeper insight into the pathophysiology of associated diseases and clarify the CNS trafficking of GABA.^638^ The neurotransmitter GABA plays a pivotal role in the CNS in the early stages of embryonic life,^639^ while in the mature CNS, it acts as the primary inhibitory signaling agent by binding to ligand-gated ion channels (GABA_A_ and GABA_B_ receptors)^640^ that favor the movement of chloride anions through the neuronal membrane.^639^ GABA activity is further regulated by transmembrane sodium symporters known as GABA transporters.^638,641^

Norepinephrine is the predominant transmitter of sympathetic innervation and regulates the body’s fight-or-flight response by binding to the α and β adrenergic receptors, and its uptake is regulated by norepinephrine transporters.^642^ The use of a carbon-11 variant of norepinephrine allowed sympathetic innervation imaging in the myocardium and diagnosis of cardiac diseases.^643^

The neurotransmitter serotonin is most active in the brain, regulating many behavioral and psychological aspects such as anxiety, aggression, learning, and depression.^644^ Serotonin can also be found outside the CNS, particularly in the lungs, with regulatory functions on respiration and vasoconstriction.^645,646^ A viable way to assess serotonin concentration in the lungs would be to determine the amount of neurotransmitters released by the lung endothelium, a physiological phenomenon that occurs while breathing.^647^ Serotonin release can be easily assessed by administration of [^11^C]serotonin and subsequent quantification of its release by serotonin lung extraction.

Even though melatonin, dihydroxyphenylalanine, progesterone, and tyramine have been labeled with carbon-11, they have not been evaluated. Melatonin its physiological function is to adapt body activity in relation to circadian rhythms.^648^ Within its functions, the most renowned is the sleep-inducing action after interaction with the suprachiasmatic nucleus in the hypothalamus.^649,650^ Octopamine is an endogenous agonist of human trace amine-associated receptor 1^651−653^ and initiates cell signal transduction pathways through binding to G-protein coupled receptors.^654^ It is related structurally and functionally to noradrenaline and acts as a “stress hormone” in fight-or-flight responses.^655^ Progesterone is a steroid hormone that regulates physiological female reproductive functions.^656^ However, it also plays a vital role in the mammary gland, cardiovascular, CNS, and bones.^656−658^ Tyramine is a decarboxylation product of tyrosine and a dehydroxylated analogue of dopamine.^659^ The presence of tyramine was initially confirmed in the human brain by post-mortem analysis^660^ and subsequently confirmed by discovering the G-coupled receptor, trace amine-associated receptor 1.^661,662^

### 8.1 Dopamine

#### 8.1.1 Radiosynthesis

**Figure 136:**
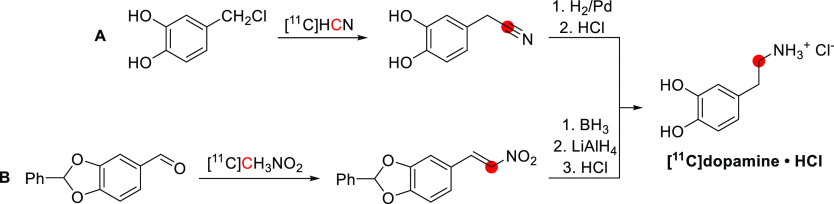
Synthesis of [^11^C]dopamine·HCl using [^11^C]HCN or [^11^C]CH_3_NO_2_. ^11^C radionuclide position is highlighted in red.

The synthesis of the radiopharmaceutical was achieved in its hydrochloride form ([^11^C]dopamine·HCl, Figure 136) using either the synthon [^11^C]HCN or [^11^C]CH_3_NO_2_. In the former case, [^11^C]HCN was reacted with *3*,*4*-hydroxybenzyl chloride to yield a [^11^C]*3*,*4*-hydroxybenzylcyanide, which was subsequently reduced with hydrogen over palladium (Figure 136). The hydrochloride form was obtained by adding HCl. The entire procedure requires 65 min, and the radiotracer was obtained with an isolated RCY of 25–30% based on the activity of [^11^C]HCN.^663^ In the latter strategy, instead, [^11^C]CH_3_NO_2_ was reacted with 3,4-benzylidenedioxybenzaldehyde to form a [^11^C]nitrovinylbenzene that was reduced to [^11^C]dopamine by the use of BH_3_ and LiAlH_4_ and deprotected by the addition of HCl with a total processing time of 45–50 min, isolated RCY of 8–20% to the initial production of [^11^C]CO_2_ and *A*_m_ of 15–37 GBq/μmol at time of injection. (Figure 136).^664^

#### 8.1.2 Preclinical Studies

A biodistribution study on five Mongrel dogs highlighted a high uptake of [^11^C]dopamine in the adrenal medulla compared to other analyzed organs (kidney, liver, heart, adrenal cortex, blood). Injecting the radiotracer in the presence of nonradioactive dopamine (0.01 mg/kg) also showed binding displacement of [^11^C]dopamine·HCl and a four-fold lower signal in the adrenal medulla.^663^

**Figure 137:**
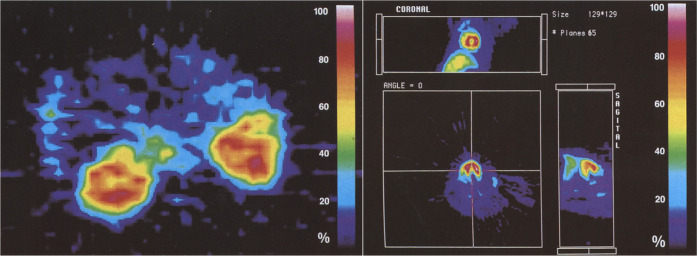
[^11^C]Dopamine PET biodistribution in cynomolgus monkeys at renal (left) and cardiac (right) levels (21 min p.i.). Reproduced with permission from ref (664). Copyright 1993 Elsevier.

These results were further explored by testing the distribution of [^11^C]dopamine in cynomolgus monkeys. This experiment used three animals, a different target organ was examined in each monkey. The first animal was scanned for brain distribution 21 min post [^11^C]dopamine injection, showing low brain activity compared to other organs (*e.g.*, heart, kidney, adrenal medulla). These findings suggest that dopamine does not cross the BBB and that the recorded low activity was related to [^11^C]dopamine in the cerebral blood volume.^664^ On the other hand, high amounts of activity were found in the pituitary gland, suggesting the potential use of [^11^C]dopamine for clinical evaluation of the pituitary. The second monkey was tested for heart and liver retention, with the heart having higher activity retention than the liver. The activity decreased in both organs to ^1^/_3_ peak concentrations within 63 min of scanning.^664^ The third animal, scanned for 38 min, showed that [^11^C]dopamine was quickly washed out from the kidneys with partial retention in the adrenal cortex (Figure 137).^664^

### 8.2 Epinephrine

#### 8.2.1 Radiosynthesis

**Figure 138:**
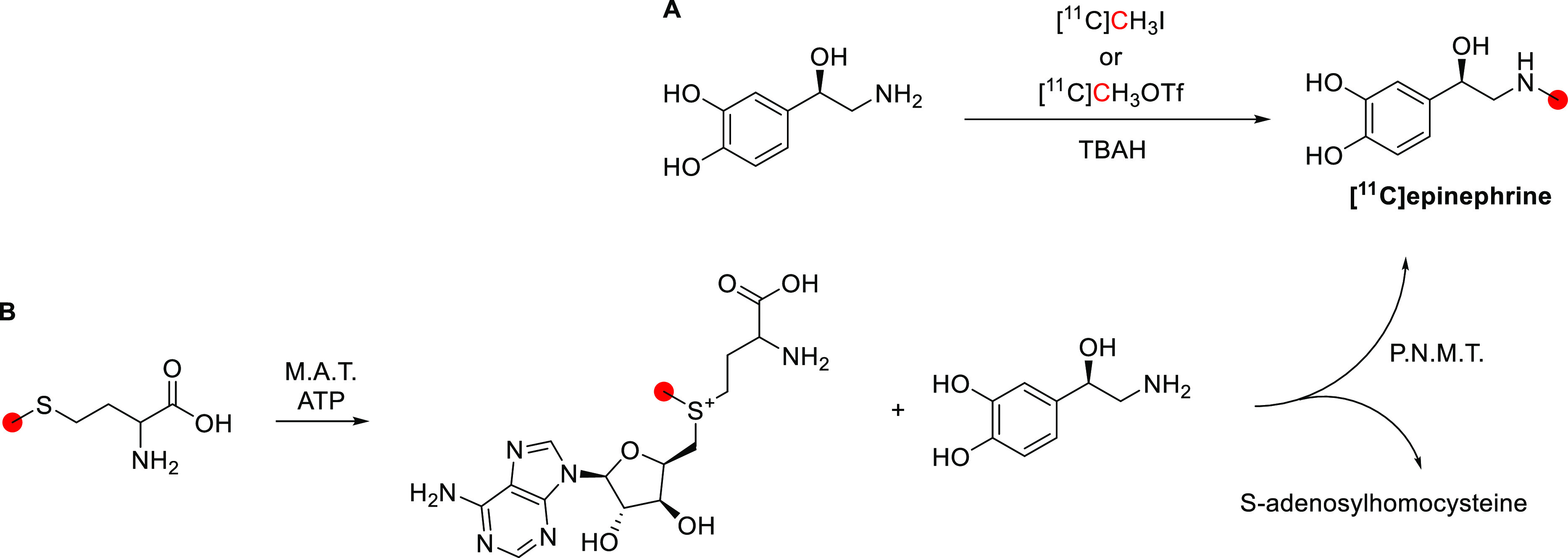
Synthesis of [^11^C]epinephrine. ^11^C radionuclide position is highlighted in red.

[^11^C]Epinephrine can be synthesized by either direct methylation of *R*-(−)-norepinephrine or by enzymatic transmethylation of *l*-[*methyl*-^11^C]methionine (Figure 138). The former synthetic route proceeds with the aid of tetrabutylammonium hydroxide (TBAH) as a base and [^11^C]CH_3_I or [^11^C]CH_3_OTf as methylating agents (Figure 138A). The isolated RCY resulted slightly higher when the more reactive [^11^C]CH_3_OTf was employed as a synthon (15–25% with [^11^C]CH_3_OTf *vs* 5–10% with [^11^C]CH_3_I). The total processing time was 35–40 min for both methods.^667^ The *A*_m_ with [^11^C]CH_3_I and [^11^C]CH_3_OTf were comparable and ranged between 33 and 81 GBq/μmol at EOS. The second method requires the initial synthesis of *l*-[*methyl*-^11^C]methionine *via*^11^C-methylation, which was then used as a substrate for the enzymatic reaction with l-methionine-*S*-adenosine transferase in the presence of ATP to yield *S*-adenosyl-*l*-[*methyl*-^11^C]methionine. Then, this intermediate undergoes another enzymatic transformation carried by phenylethanolamine *N*-methyltransferase (PNMT) that moves the [^11^C]methyl group onto *R*(−)-norepinephrine (Figure 138B). [^11^C]Epinephrine was obtained in 30–35 min with an isolated RCY of 20% from *l*-[*methyl*-^11^C]methionine and *A*_m_ of 7.4 GBq/μmol at 35 min from *l*-[*methyl*-^11^C]methionine synthesis.^665^

#### 8.2.2 Preclinical Studies

[^11^C]Epinephrine was evaluated *in vivo* by injection in one rabbit (28 min scanning). The radiopharmaceutical was mainly biodistributed in the heart, liver-pancreas, kidneys, and adrenal glands. The activity in the heart, liver, and pancreas was constant during the scan. Surprisingly, the expected signal in the lungs was neglectable despite the high expression of adrenergic receptors in this tissue. An increase in activity in the kidneys (∼40%) and bladder (3.5-fold higher) was also observed during the time of the scan, indicating a fast excretion of [^11^C]epinephrine. Given this data, the biological half-life of [^11^C]epinephrine was estimated to be 3 min.^665^*In vivo* studies also aimed to understand the effects of myocardial infarction on sympathetic innervation in the heart.^666^ [^11^C]Epinephrine (dynamic image acquisition for 40 min) was administered to 11 pigs with induced myocardial infarction, revealing a decrease of 44% in radioactivity retention compared with healthy animal control.^666^

#### 8.2.3 Clinical Studies

**Figure 139:**
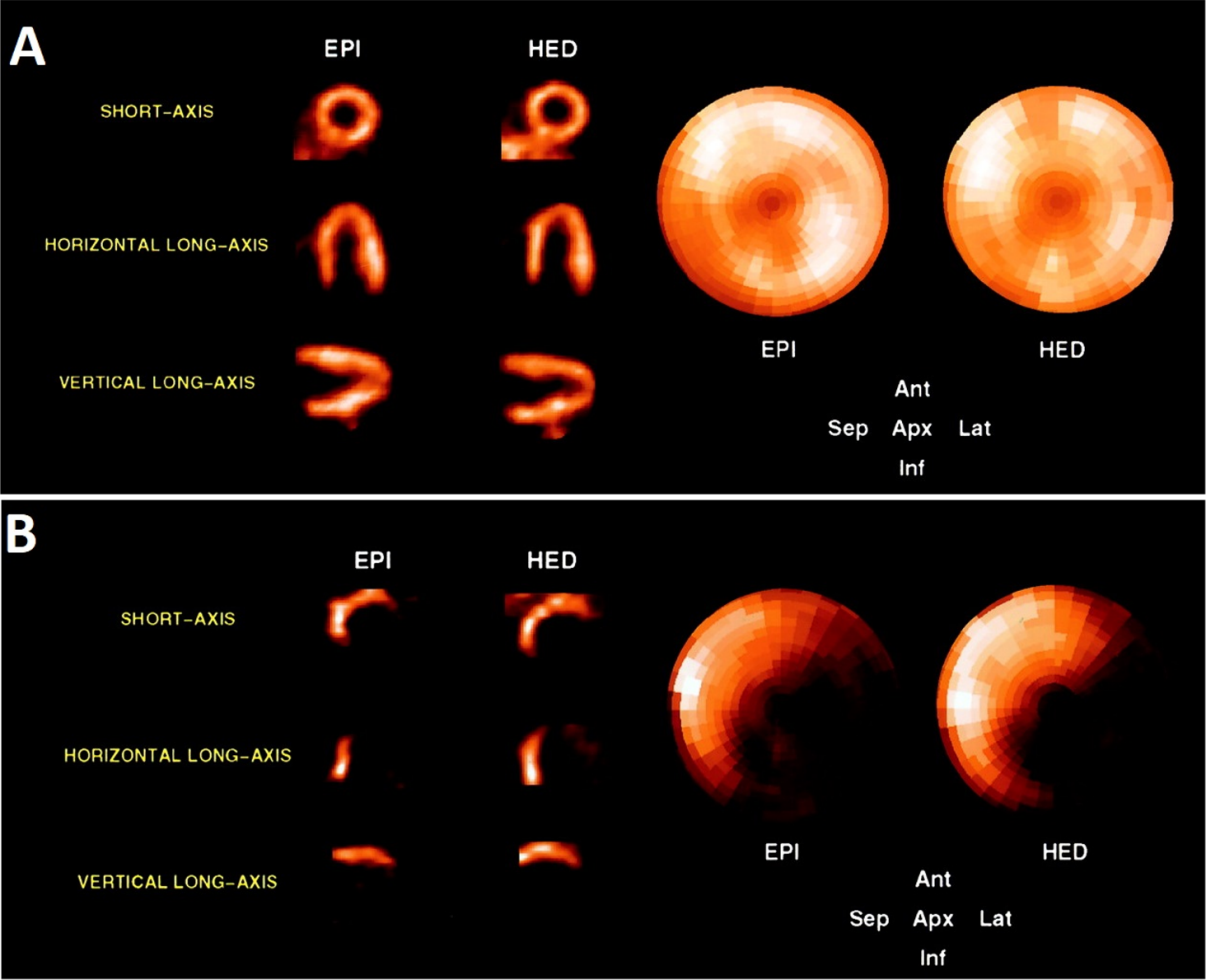
[^11^C]Epinephrine (EPI) and [^11^C]hydroxyephedrine (HED) PET images and corresponding polar maps in (A) a healthy volunteer and (B) a patient who underwent heart transplantation 37 months before PET studies. Reproduced with permission from ref (637). Copyright 2000 Wolters Kluwer Health.

Modifications on the heart sympathetic innervation with [^11^C]epinephrine were also studied in the case of heart transplantation. Ten patients who underwent heart transplantation 3.5–48.0 months before the study were injected with [^11^C]epinephrine and scanned for 60 min. The results highlighted a considerable decrease in activity in the myocardium compared to seven healthy volunteers (−75% of activity detected, Figure 139). Metabolite analysis was also carried out by collecting blood samples at different time points after the injection. As early as 5 min after tracer injection, the amount of unmetabolized [^11^C]epinephrine was only 65% of total blood radioactivity, which decreased to 14% after 60 min, thus highlighting a quick metabolism and washout of the tracer. The metabolite identification was not performed. No significant differences were observed between the healthy volunteers and the transplant recipients.^637^

### 8.3 Gamma-Aminobutyric Acid (GABA)

#### 8.3.1 Radiosynthesis

**Figure 140:**
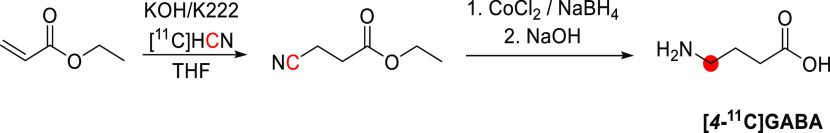
Synthesis of [*4*-^11^C]GABA using [^11^C]HCN. ^11^C radionuclide position is highlighted in red.

The synthesis of [^11^C]GABA was accomplished *via* Michael’s addition of [^11^C]KCN (previously obtained by dissolving [^11^C]HCN in a KOH/K222 solution) on an ethyl acrylate precursor followed by a selective reduction and hydrolysis of the resulting amino ester (Figure 140). With this methodology, the radiolabeled [*4*-^11^C]GABA was obtained with a RCY of 60–65% based on the activity of [^11^C]HCN within 40 min from [^11^C]HCN addition.^638^ The final product had a *A*_m_ of 4 GBq/μmol.^638^

### 8.4 *l*-DOPA

#### 8.4.1 Radiosynthesis

**Figure 141:**
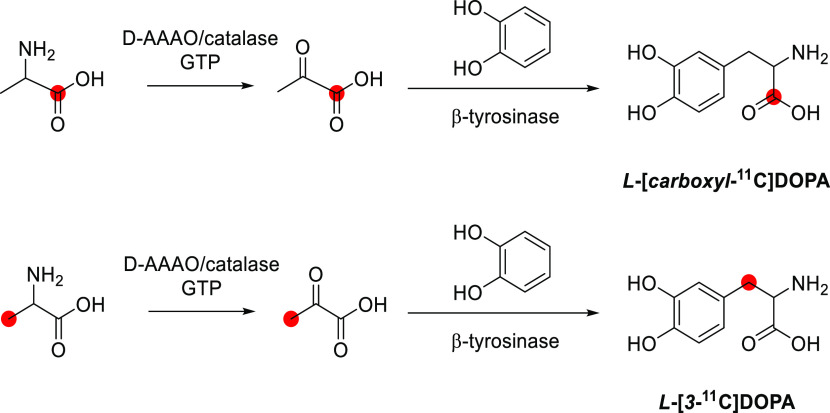
Synthesis of *l*-[*β*-^11^C]DOPA and *l*-[*carboxyl*-^11^C]DOPA *via* a multienzymatic procedure. ^11^C radionuclide position is highlighted in red.

The synthesis of the radiopharmaceutical was achieved in both *β* (*l*-[*β*-^11^C]DOPA) and terminal carboxylic position (*l*-[*carboxyl*-^11^C]DOPA) by a combination of organic synthesis methods and a multienzymatic procedure.^693,694^ A ^11^C-labeled alanine was used as a substrate for the enzymes d-amino acid oxydase, catalase, glutamic-pyruvic transaminase, and *β*-tyrosinase, which in turn oxidizes the [^11^C]alanine and then attach it to a catechol precursor (Figure 141). Both *l*-[*β*-^11^C]DOPA and *l*-[*carboxyl*-^11^C]DOPA were obtained within 50 min and with an isolated RCY of 25–30% and 45–60%, respectively, and *A*_m_ ranging between 0.4 and 2.5 GBq/μmol.^678^ The *l*-[*β*-^11^C]DOPA synthesis was also fully automated using an immobilized enzyme column.^694^

#### 8.4.2 Preclinical Studies

Both variants of *l*-[*β*-^11^C]DOPA were tested *in vivo* to disentangle l-DOPA’s metabolic pathways and assess its distribution in the brain, the rate of decarboxylation to dopamine and the effect of pharmacological treatments. Studies in rhesus monkeys highlighted a prominent accumulation of radioactivity in the striatal region.^635,670^ The maximum accumulation of activity in the striatum was reached after 10 min,^671^ and the binding specificity was confirmed *via* levodopa blocking studies.^672^ The use of pharmacological treatments such as *6R*-BH_4_ (a cofactor of AADC, catalyzing dopamine synthesis) significantly increased the influx of *l*-[^11^C]DOPA in the striatum. In contrast, the administration of tolcapone or catechol-*O*-methyltransferase inhibitors did not give significant variations.^635,670,672^ High doses of 6R-BH4 also increased the rate of decarboxylation of *l*-[^11^C]DOPA to [^11^C]dopamine. This effect was further amplified by the simultaneous administration of *6R*-BH_4_ and *l*-tyrosine.^635,670−672,695^

The infusion of scopolamine, a muscarinic cholinergic antagonist, or nicotine also resulted in a dose-dependent increase in uptake and decarboxylation rate of *l*-[^11^C]DOPA in the striatum (0.017 MBq/mL with scopolamine *versus* 0.012 MBq/mL without scopolamine; 33% increase in *l*-[^11^C]DOPA decarboxylation rate when nicotine was administered), confirming the presence of a connection between cholinergic and dopaminergic systems in the striatal region.^671,696^ AADC inhibition resulted in increased retention of activity in the brain of 43–50%, whereas infusion of reserpine or *l*-deprenyl (to reduce the accumulation in presynaptic vesicles) did not significantly alter l-[^11^C]DOPA uptake.^672^

**Figure 142:**
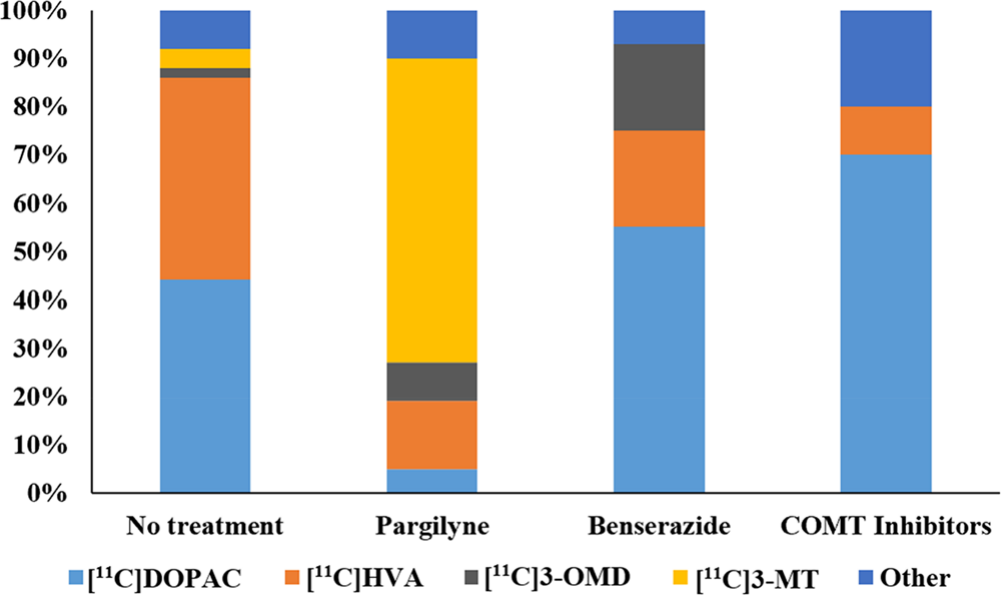
*l*-[^11^C]DOPA metabolism analyzed by microdialysis studies on male Sprague-Dawley rats (“Other” indicates an unidentified ^11^C-labeled metabolite). Graph was prepared with data from Okada et al.^668^

Microdialysis studies on male Sprague-Dawley rats made a deeper understanding of dopaminergic metabolism. l-[^11^C]DOPA was metabolized in [^11^C]*3*,*4*-dihydroxyphenylacetic acid ([^11^C]DOPAC) for 44% and [^11^C]homovanillic acid ([^11^C]HVA) for 42% within 10 min. Pharmacological treatments, however, can modify the metabolic pathway of *l*-[^11^C]DOPA. The use of the monoamine oxidase inhibitor pargyline 30 min before *l*-[^11^C]DOPA administration lowered the production of the two species mentioned above in favor of [^11^C]3-methoxytyramine ([^11^C]*3*-MT). In contrast, the infusion of COMT inhibitors or benserazide significantly favored the formation of [^11^C]DOPAC (Figure 142).^668^ Furthermore, the administration of 6*R*-l-erythro-5,6,7,8-tetrahydrobiopterin (*6R*-BH_4_, 50 mg/kg 40 min after l-[^11^C]DOPA injection) showed to enhance the uptake and turnover of l-[^11^C]DOPA in the striatum. The simultaneous infusion of l-tyrosine further enhanced the effect of 6*R*-BH_4,_ whereas only *l*-tyrosine did not have any effect.^669^

Alterations in the dopaminergic synthesis were subsequently studied by concomitant administration of *d*-amphetamine and *l*-[*β*-^11^C]DOPA to eight anesthetized rhesus monkeys to study the variation in the decarboxylation rate of *l*-[*β*-^11^C]DOPA.^697^d-Amphetamine was administered as a bolus dose of 0.1–0.4 mg/kg 30 min prior to *l*-[*β*-^11^C]DOPA, and the scan proceeded for 60 min. Results showed an increased decarboxylation rate of *l*-[*β*-^11^C]DOPA of 5% when 0.1 mg/kg of d-amphetamine was administered and 12% with a bolus dose of 0.4 mg/kg, confirming the positive effect of amphetamines on dopamine production. The constant intravenous infusion showed no prominent increase in *l*-[*β*-^11^C]DOPA decarboxylation as bolus administration.^697^

#### 8.4.3 Clinical Studies

Clinical studies in humans confirmed what preclinical experiments suggested. The scanning of eight healthy volunteers showed high retention of activity in the striatal region and mesial frontal cortex (in line with dopaminergic neuron presence) and a steep washout from the cerebellum 15 min p.i. of *l*-[^11^C]DOPA.^673,674^ In contrast to preclinical studies,^672^ pretreatments with unlabeled *l*-DOPA increased brain activity.^673^ Due to the rapid decarboxylation in the body, *l*-[*carbonyl*-^11^C]DOPA was quickly metabolized into [^11^C]carboxylates which nonspecifically bind in all brain regions, lowering the specificity of the carboxy-labeled analogue. *l*-[*β*-^11^C]DOPA, instead, does not generate any nonspecific metabolites.^673^

Another clinical study on 10 healthy male volunteers focused on the relationship between the concentration of neutral amino acids in the blood and *l*-[*β*-^11^C]DOPA influx in the brain by injection of *l*-[*β*-^11^C]DOPA.^674^ A strong negative correlation was found between the amount of *N*-acetylaspartate in the blood pool and the influx rate constant, with a decrease of 25% in the influx rate constant in the putamen region when the NAA concentration increases by 40%. Thus, this data suggests that *l*-[^11^C]DOPA is transported inside the CNS by the *N*-acetylaspartate transporter system at the BBB and competes with *N*-acetylaspartates for the transporter.^674^

Alterations in the dopaminergic system are frequently seen in brain disorders, particularly Parkinson’s disease and schizophrenia.^675−677^ The use of *l*-[^11^C]DOPA would help to depict these modifications. A study on eight idiopathic Parkinson’s disease patients highlighted a 35% decrease in *l*-[^11^C]DOPA accumulation in the brain compared to healthy references.^673,676^ On the other hand, a comparative study between 113 patients with schizophrenia and 131 healthy controls showed a 14% increase in dopamine synthetic capacity in schizophrenic subjects.^677^

### 8.5 Melatonin

#### 8.5.1 Radiosynthesis

**Figure 143:**
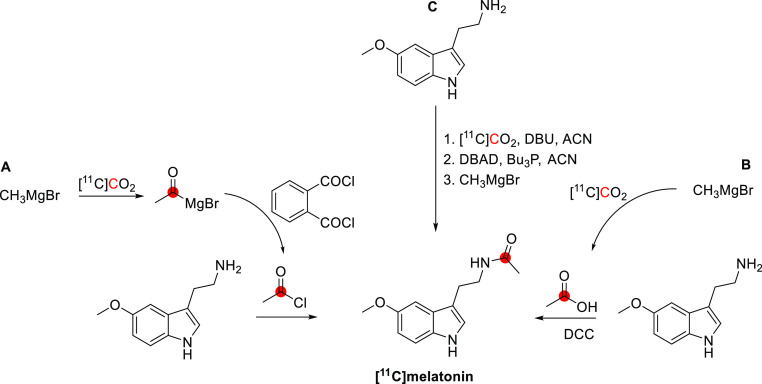
Synthesis of [^11^C]melatonin using [^11^C]CH_3_COCl, [^11^C]CO_2_ and [^11^C]CH_3_CO_2_H. ^11^C radionuclide position is highlighted in red.

Three strategies for the synthesis of a ^11^C-labeled analogue of melatonin were proposed in the past years to better understand its activity in the brain.^650,680,681^ All these methodologies proceed with radiolabeling the terminal acetyl group of melatonin (Figure 143). The first requires the production of the synthon [^11^C]CH_3_COCl by reacting CH_3_MgBr with the cyclotron-produced [^11^C]CO_2_, obtaining [^11^C]acetate, which was subsequently functionalized by reaction with phthaloyl chloride.^650^ The synthon [^11^C]CH_3_COCl then reacts with the precursor 5-methoxytryptamine producing [^11^C]melatonin with a RCY of 13% and *A*_m_ of 3.49 GBq/μmol within 35 min from the EOB (Figure 143A).^650^

The second, instead, exploits [^11^C]acetate as the radiolabeling agent, obtained by the reaction of CH_3_MgBr with [^11^C]CO_2_.^680^ The newly synthesized labeling agent was then reacted with the precursor *5*-methoxytryptamine with the aid of *N*,*N*′-dicyclohexylcarbodiimide (DCC) (Figure 143B).^680^ [^11^C]Melatonin was obtained with RCY of 12% and *A*_m_ of 155 GBq/μmol in 45 min from EOB.^680^

Improvements in [^11^C]CO_2_ chemistry allowed the development of the third method for radiolabeling [^11^C]melatonin.^681^ Using a CO_2_-fixating agent (*1*,*8*-diazabicyclo[*5*.*4*.*0*]undec-*7*-ene (DBU)), Mitsunobu reagents (tri-*n*-butyl phosphine (Bu_3_P) and di-*tert*-butyl azodicarboxylate (DBAD)), and CH_3_MgBr, the radiopharmaceutical was obtained with RCY of 36% (non-isolated, calculated from the radio HPLC chromatogram) and *A*_m_ of 70–100 GBq/μmol in only 2 min from EOB.^681^ Neither preclinical nor clinical studies have been reported (Figure 143C).

#### 8.5.2 Clinical Studies

Biodistribution studies with [^11^C]melatonin were performed on a 38-year-old volunteer. After injection of the tracer, PET images and 23 blood samples were regularly taken over a 75 min period to monitor the accumulation of radioactivity in the body and the formation of radiometabolites. [^11^C]Melatonin quickly crossed the BBB, and activity in the brain peaked within 8.5 min, whereas plasma concentration apexed in 3.5 min. The main metabolite was 6-sulfatoxymelatonin, and its concentration plateaued after 20 min. However, it was impossible to locate the specific binding sites of [^11^C]melatonin in the human brain, potentially due to the low affinity or low molar activity of the produced tracer.^679^

### 8.6 Norepinephrine

#### 8.6.1 Radiosynthesis

**Figure 144:**
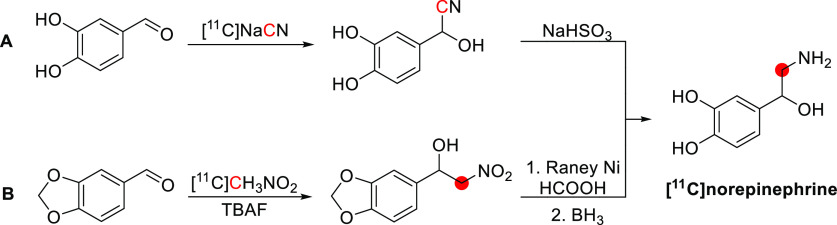
Synthesis of [^11^C]norepinephrine. ^11^C radionuclide position is highlighted in red.

The synthesis of [^11^C]norepinephrine was achieved with two strategies involving the synthons [^11^C]HCN or [^11^C]CH_3_NO_2_. The radiolabeling with [^11^C]HCN proceeds with the initial trapping of the synthon as [^11^C]NaCN and the subsequent reaction with 3,4-hydroxybenzaldehyde in the presence of NaHSO_3_ as a reducing agent (Figure 144A). The use of HCl then allows the purification *via* ion-exchange chromatography and gives [^11^C]norepinephrine·HCl with an isolated RCY of 20–25% (at the end of [^11^C]HCN synthesis) within 40 min.^684^ The second strategy involving [^11^C]CH_3_NO_2_, instead, requires the two hydroxyl groups to be protected as dioxole. [^11^C]CH_3_NO_2_ reacts with a *3*,*4*-methylenedioxybenzaldehyde in the presence of TBAF, forming a [*β*-^11^C]*3*,*4*-(methylenedioxy)-*β*-nitrophenetyl alcohol, which was subsequently reduced using Raney nickel and formic acid and deprotected with BH_3_ (Figure 144B).^685^ [^11^C]Norepinephrine was synthesized with an isolated RCY of 20–25% at the end of [^11^C]CH_3_NO_2_ synthesis and *A*_m_ of 26–56 GBq/μmol with a total processing time of 65–70 min.^685^

#### 8.6.2 Preclinical Studies

The biodistribution of [^11^C]norepinephrine was tested with *in vivo* studies on four dogs. The radiopharmaceutical showed a quick blood clearance with an estimated biological half-life of 2 min.^682^ The highest retention was detected in the adrenal medulla, heart, and kidneys, whereas very little activity was detected in the lungs. The activity in each organ was stable for the whole scanning time (90 min), thus showing a slow washout from the target organs.^682^

Other *in vivo* studies focused on how pharmacological agents affect the pharmacokinetics of [^11^C]norepinephrine. In particular, the effect of desipramine (inhibitor of the norepinephrine reuptake transporter) and cocaine on [^11^C]norepinephrine biodistribution was investigated in cynomolgus monkeys.^642,683^ Both desipramine and cocaine had a negative effect on [^11^C]norepinephrine retention in the heart (−80% with desipramine and −33% with cocaine).^642,683^

Metabolite studies were also performed in cynomolgus monkeys. [^11^C]Norepinephrine showed a high metabolic resistance in the plasma, with 90% of the parent molecule remaining intact at 30 min after the tracer injection.^642,643^

### 8.7 Octopamine

#### 8.7.1 Radiosynthesis

**Figure 145:**
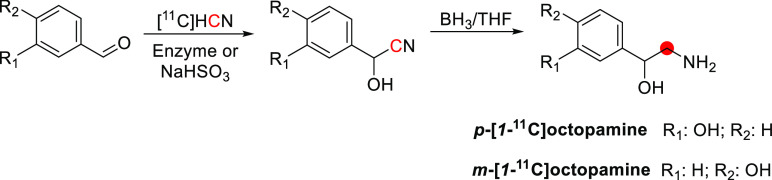
Synthesis of *p*- and *m*-[*1*-^11^C]octopamine from [^11^C]HCN in a two-step sequence.^686,687^^11^C radionuclide position is highlighted in red.

*p*- and *m*-[^11^C]Octopamine have been first synthesized from [^11^C]HCN in a two-step sequence by Maeda *et al*. (Figure 145).^686,687^ Chemical and enzymatic approaches have produced [^11^C]cyanohydrin intermediates as the critical step. [^11^C]octopamine was prepared within 40–60 min, with a RCY of 0.7–2.3% at EOS, and ee of 92% in the (*S*)-enantiomer for *p*- and 42% in the (*R*)-enantiomer for *m*-[*1*-^11^C]octopamine, through the enzymatic process, as determined by HPLC without any derivatization.^686,687^

**Figure 146:**
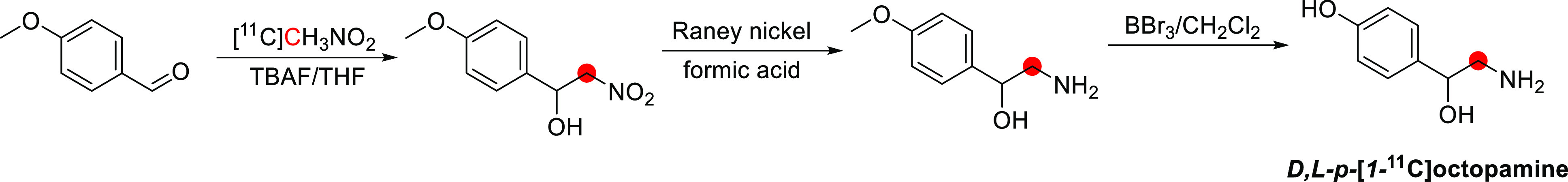
Synthesis of *d*,*l*-*p*-[*1*-^11^C]octopamine from [^11^C]CH_3_NO_2_.^685^^11^C radionuclide position is highlighted in red.

*d*,*l*-*p*-[*1*-^11^C]Octopamine has also been prepared by reaction of 4-methoxy-benzaldehyde and the formed [*2-*^11^C]*4*-methoxy-nitrophenethyl alcohol, followed by Raney nickel reduction and boron tribromide deprotection as described in Figure 146. *d*,*l*-*p*-[1-^11^C]Octopamine was prepared within 60–65 min, with a RCY of 20–25% (from [^11^C]CH_3_NO_2_), RCP of >98%, and *A*_m_ of 26–56 GBq/μmol.^685^

### 8.8 Progesterone

#### 8.8.1 Radiosynthesis

**Figure 147:**
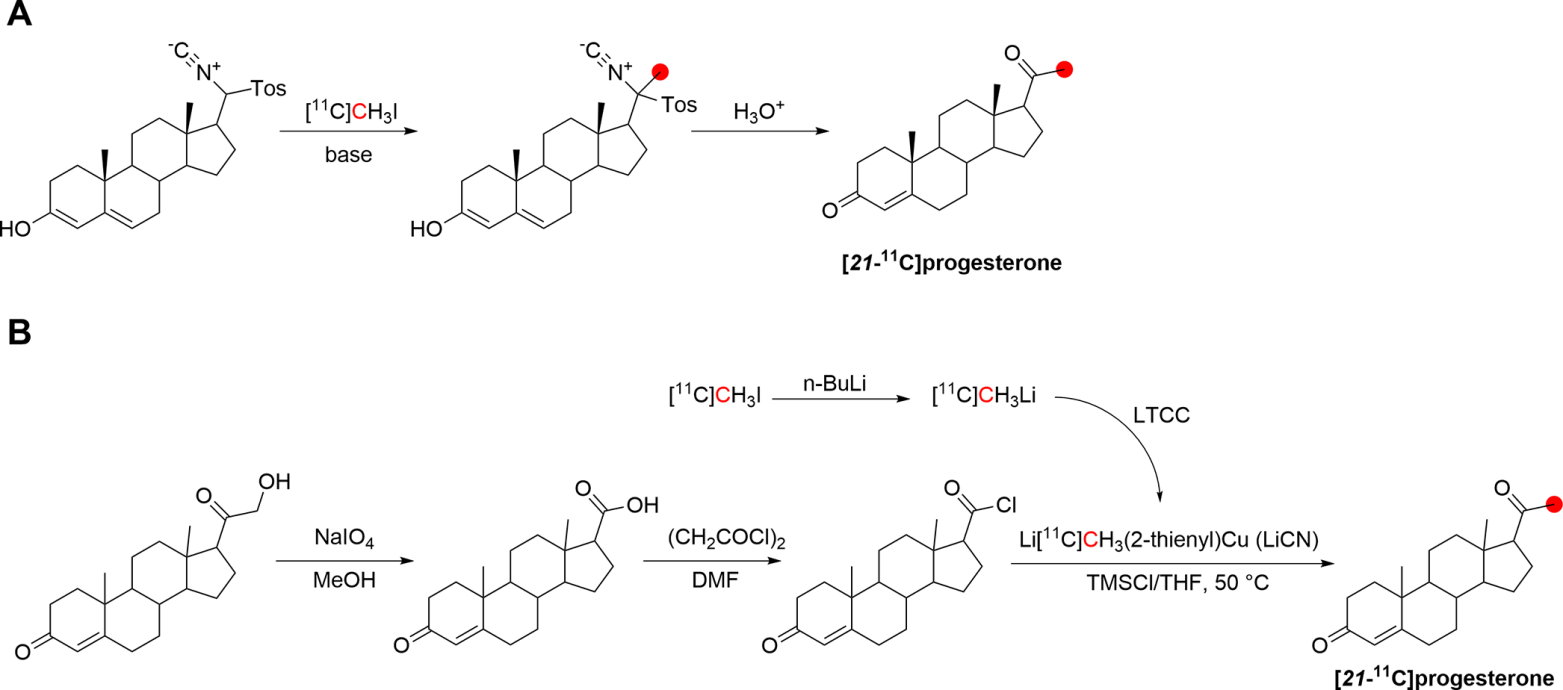
Synthesis of [*21*-^11^C]progesterone. ^11^C radionuclide position is highlighted in red.

Following these hypotheses, the labeling of a carbon-11 analogue of progesterone was proposed as a viable alternative for cancer imaging. Two pathways were developed for the synthesis of [*21*-^11^C]progesterone.^688,689^ The first method employed [^11^C]CH_3_I as a radiolabeling agent and a *p*-toluenesulfonylmethyl isocyanide derivative of progesterone (Figure 147) as the precursor. The precursor was initially dissolved in aqueous NaOH, followed by [^11^C]CH_3_I delivery and the radiolabeling at 100 °C for 15 min. The isocyanide intermediate was hydrolyzed to yield the desired product by adding concentrated HCl and stirring for 5 min at 70 °C. [*21*-^11^C]Progesterone was obtained within 60 min from [^11^C]CH_3_I delivery with an isolated RCY of 13% and *A*_m_ of 0.74 GBq/μmol at 60 min from [^11^C]CH_3_I delivery.^688^

The second radiolabeling pathway proceeded with the aid of [^11^C]methyl(*2*-thienyl)cuprate as a synthon which was synthesized from [^11^C]CH_3_I by initial conversion to [^11^C]CH_3_Li with an excess of *n*-BuLi and subsequent reaction with lithium(*2*-thienyl)cyanocuprate (Figure 147). The precursor *4*-androsten-*3*-one-*5*-ene-*17*-carboxylic acid chloride was synthesized starting from desoxycortisone by oxidation to *4*-androsten-*3*-one-*5*-ene-*17*-carboxylic acid and functionalization to its acyl chloride analogue with oxalyl chloride (Figure 147).^689^ The ^11^C-labeling was then performed *via* cross-coupling of LiCN and *4*-androsten-*3*-one-*5*-ene-*17*-carboxylic acid chloride in THF at 50 °C in the presence of trimethylsilyl chloride (TMSCl) (Figure 147). [*21*-^11^C]Progesterone was obtained with an isolated RCY of 30–35% (based on [^11^C]CH_3_I initial activity) within 35 min from EOB and *A*_m_ of 14 GBq/μmol.^689^

### 8.9 Serotonin

#### 8.9.1. Radiosynthesis

**Figure 148:**
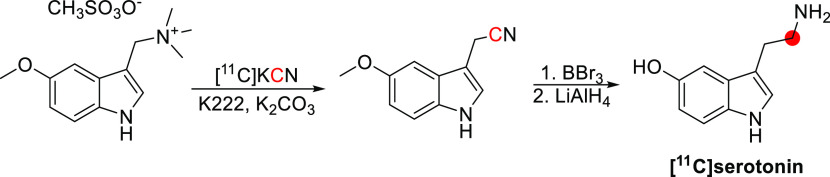
Synthesis of [^11^C]serotonin using [^11^C]KCN. ^11^C radionuclide position is highlighted in red.

The synthesis of [^11^C]serotonin was achieved by the aid of the synthon [^11^C]HCN, which was initially trapped in solution as a potassium salt ([^11^C]KCN) using Kryptofix K222 and K_2_CO_3_. A 5-methoxygramine methyl sulfate precursor was introduced into the reaction mixture and reacts with [^11^C]KCN to yield a [^11^C]*5*-methoxy-*3*-acetonitrile indole. By deprotection with BBr_3_ and reduction with LiAlH_4_, the product [^11^C]serotonin was obtained (Figure 148) with an isolated RCY of 13% and *A*_m_ of 5.1 GBq/μmol within 78 min.^692^

#### 8.9.2 Preclinical Studies

Determination of single-pass extraction was initially tested on anesthetized dogs and rabbits, highlighting a radioactivity uptake in the lung >50% of the total bolus of [^11^C]serotonin injected. The study, however, does not report any data on pulmonary extraction.^690^

#### 8.9.3 Clinical Studies

Determination of single-pass extraction was tested on three healthy volunteers, and pathological modifications were mimicked by infusing imipramine (a serotonin transporter inhibitor, 0.4–0.5 mg/kg iv, 10 min before [^11^C]serotonin administration).^691^ After imipramine infusion, the amount of [^11^C]serotonin extracted by the lungs was significantly lower (−16% at 5 min p.i. of [^11^C]serotonin), confirming the feasibility of using this technique to assess the integrity of the lung endothelium.^691^

### 8.10 Tyramine

#### 8.10.1 Radiosynthesis

**Figure 149:**
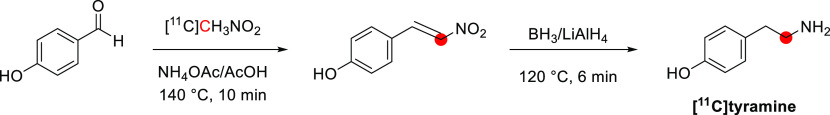
Graphical representation of the radiolabeling strategy for [^11^C]tyramine. ^11^C radionuclide position is highlighted in red.

The synthesis of [^11^C]tyramine was achieved by using [^11^C]CH_3_NO_2_ as a radioactive synthon that reacts with 4-hydroxybenzaldehyde in the presence of ammonium acetate and acetic acid for 10 min at 140 °C.^664^ The resulting [^11^C]nitropropene (Figure 149) was reduced over borane and lithium aluminum hydride at 120 °C for 6 min, returning [^11^C]tyramine with RCY of 8–20% based on the activity of [^11^C]CO_2_, within 45–50 min from EOB and *A*_m_ of 15–37 GBq/μmol at EOS.^664^

## 9 Nucleotides, Nucleosides, and Nucleobases

Nucleotides are the monomeric units of DNA and ribonucleic acid (RNA), consisting of a nucleoside and a phosphate. Nucleosides are glucosamines consisting of a nucleobase and a five-carbon sugar. Nucleobases are simple bases constituting the basic building blocks of nucleic acids, with the five (adenine, cytosine, guanine, thymine, and uracil) being the fundamental units of the genetic code. Adenine forms the nucleoside adenosine when coupled with ribose *via* a *β*-*N*^9^-glycosidic bond, as found in RNA. In DNA, adenine is attached to deoxyribose to form deoxyadenosine. Its phosphorylated analogues AMP, adenosine diphosphate (ADP), and adenosine triphosphate (ATP) act as cellular energy sources, while cAMP is an important second messenger involved in intracellular signal transduction.

Adenosine, ADP, and AMP are endogenous ligands that mediate many physiological processes through binding to the purinergic receptors (P1, P2X, and P2Y).^698^ Thymidine consists of the pyrimidine nucleobase thymine attached to deoxyribose *via* an *N*-glycosidic bond. Thymidine is transported across the cell membrane from the bloodstream by equilibrative nucleoside and concentrative transporters, found in most cell types. Inside the cell, it can either be phosphorylated by thymidine kinase and incorporated into DNA or degraded to thymine by thymidine phosphorylase. Thymine is ultimately catabolized to CO_2_, NH_3_, and *β*-aminoisobutyric acid (*β*-AIB), by reduction, ring-opening, and decarboxylation.^699^ The pyrimidine derivative uracil, initially discovered by Alberto Ascoli in 1900,^700^ is one of the four nucleobases in RNA, while in DNA, it is replaced by its demethylated form, thymine.^701^ Unlike adenine, cytosine, and guanine, thymine is not incorporated into RNA, making thymidine a unique precursor to DNA.

Thymidine was one of the earliest endogenous compounds radiolabeled with carbon-11 due to its potential to measure DNA synthesis and tumor proliferation.^36,702^ To evaluate metabolite kinetics in [^11^C]thymidine PET imaging studies, the initial degradation product, [^11^C]thymine, has also been radiolabeled and studied *in vivo*.^703^ Adenine has been radiolabeled with carbon-11 to evaluate the placental transfer of metabolic substrates.^261,704^ AMP has been radiolabeled and evaluated for its potential as an imaging agent of tumor metabolism.^261,704−707^ Uracil has also been labeled but not evaluated yet (Table 8).

**Table 8:** Carbon-11 Labeled Nucleotides, Nucleosides, and Nucleobases

compd	radiolabeling position	preclinical and clinical studies	synthon	*A*_M_ (GBq/μmol)	RCY	total time (min)	ref
adenine	*4*-, *5*-, and *6*-	rats^261,704^	[^11^C]HCN	nr^a^	8%	60	(708)
adenosine monophosphate	*2*-	mice,^705^ rats^706^	[^11^C]CH_2_O	90	2.4%	34	(705)

thymidine	*methyl*-	mice,^36,703,709^ rabbits,^702^ rat,^710−713^ dogs,^445,709,714^ humans^699,703,715−721^	[^11^C]CH_2_O	3	nr	70	(702)
			[^11^C]CH_3_I	147	75%	39	(722)
			[^11^C]CH_3_ZnI	>50	6%	nr	(723)
	*2*-	nr	[^11^C]CO(NH_2_)_2_	52	14%	40	(724)

thymine	*2*-	humans^703,715−717^	[^11^C]CO(NH_2_)_2_	52	14%	40	(724−726)
			[^11^C]COCl_2_	nr	38%	nr	(727,728)

uracil	*2*-	nr	[^11^C]CO(NH_2_)_2_	nr	75%	nr	(729)

a nr: not reported.

### 9.1 Adenine and Adenosine Monophosphate

#### 9.1.1 Radiosynthesis

**Figure 150:**
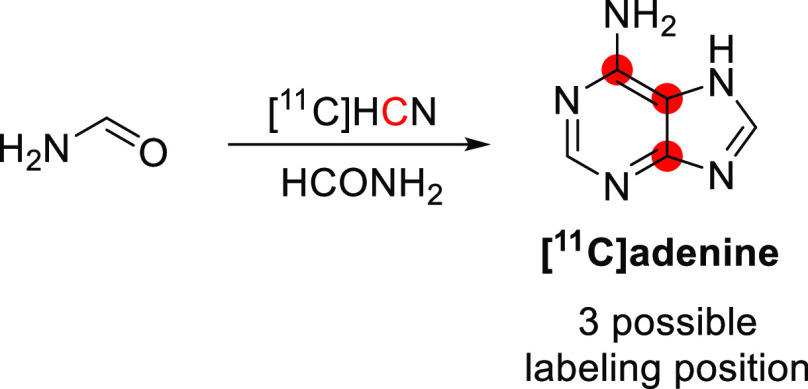
Synthesis of [^11^C]adenine using [^11^C]HCN. ^11^C radionuclide positions are highlighted in red.

The synthesis of [^11^C]adenine was reported in 1983 by Ido *et al.*,^704^*via* reaction of carrier-added [^11^C]HCN with formamide (Figure 150). This procedure is based on a process performed five years ago with [^13^C/^15^N]HCN.^708^ The ^11^C-labeling reaction was conducted in a sealed reaction vessel at 160 °C over 30 min, producing [^11^C]adenine in 6–8% isolated RCY, with an overall synthesis time of 60–70 min, including HPLC purification. As elucidated from the ^13^C/^15^N experiments, the C-4, C-5, and C-6 adenine carbons are derived from HCN. Hence the ^11^C radiolabel is expected to reside in any of these positions, although not in multiple positions within the same molecule due to the high isotopic dilution of ^11^C under standard radiolabeling conditions.

**Figure 151:**
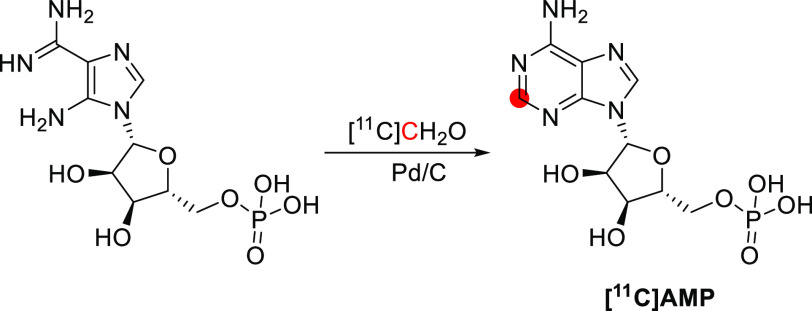
Synthesis of [^11^C]AMP using [^11^C]CH_2_O. ^11^C radionuclide position is highlighted in red.

The nucleotide AMP has been radiolabeled with ^11^C in the C-2 position through cyclization reaction of an acyclic carboxamidine precursor with [^11^C]formaldehyde in the presence of Pd/C (Figure 151).^705^

This one-step reaction proceeded at 80 °C over 10 min, and after HPLC purification and formulation, provided [^11^C]AMP for use in preclinical experiments (synthesis time, 34 min; RCY, 2.4% from [^11^C]CH_2_O; *A*_m_, 90 GBq/μmol).

#### 9.1.2 Preclinical Studies

[^11^C]Adenine has been administered to male and pregnant rats to evaluate the biodistribution, and placental transfer of metabolic substrates following iv injection.^261,704^ High incorporations were observed in the lung, with moderate uptake in the heart, liver, small intestine, and pancreas.^704^ In pregnant rats, the [^11^C]adenosine concentration was much smaller in the fetal organs than in the maternal organs. A fetus-to-placenta ratio of 31% *vs* a placenta-to-blood ratio of 52% (at 30 min) also indicated limited transfer through the placenta. Less than 0.1%ID/g was observed in the maternal brain because of the effect of the BBB. However, a fetal brain-to-maternal brain uptake ratio of 5 was observed, suggesting an immature BBB in the fetal brain.^261^

[^11^C]AMP has been evaluated in mice^705^ and tumor-bearing rats,^706^ for biodistribution and PET studies, respectively. *In vivo*, extracellular adenylates such as AMP are known to undergo rapid dephosphorylation to adenosine, which is then available for cellular uptake *via* nucleoside transporters and subsequent intracellular rephosphorylation to ATP. As such, [^11^C]AMP was investigated for its potential to study normal adenylate metabolism and in pathological conditions such as cancer, where accelerated import/export of adenylates associated with tumor metabolism is expected.

The biodistribution study of [^11^C]AMP in mice showed the highest accumulation of activity in the lungs (30%ID/g), blood (27%ID/g), and heart (18%ID/g), with the lowest in the brain (1%ID/g) (all values 30 min p.i.). The high uptake in blood and perfused organs indicates that [^11^C]AMP participates in the normal adenylate metabolism and delivery cycle. The low brain uptake suggests that the tracer does not cross the intact BBB. The effect of the nucleoside transporter inhibitor dipyridamole (30 mg/kg) on [^11^C]AMP uptake in mice was also investigated. This showed no significant effect on blood activity levels; however, at 60 min p.i., lung uptake was reduced to about 40%.^705^

A baseline *in vitro* experiment was performed to study radiotracer metabolism in human blood. Three adenylate radiometabolites were observed, ATP, ADP, and adenosine. In whole blood, [^11^C]AMP was found to convert to ATP *via* ADP rapidly; at 5 min p.i., the parent was almost undetectable (<1%), with most of the activity as ADP (42%) and AMP (44%). AMP levels increased to ∼60% at 10 min and stabilized afterward. Conversely, in plasma, where red blood cells are absent, [^11^C]AMP was dephosphorylated to adenosine after 5 min. A similar effect was observed in dipyridamole-treated whole blood, where cellular uptake of adenosine was blocked; at 5 min, the major metabolite was adenosine (>70%), with minimal ADP and ATP formation.

In 2006, the potential of [^11^C]AMP as a PET radiotracer for imaging nucleoside transporters in a rat tumor model was explored by Cho *et al.*,^706^ having previously demonstrated that various tumor cell lines had marked uptake of [^3^H]AMP through equilibrative or concentrative nucleoside transporters.^707^ Whole-body images showed intense [^11^C]AMP distribution and uptake to lungs, myocardium, kidney, and brown adipose tissue in both tumor model systems. The authors concluded that there was sufficient tumor uptake *in vivo* to warrant further evaluation of [^11^C]AMP as a radiotracer for tumor nucleoside transporter activity and possibly to assess resistance to nucleoside analogue chemotherapy agents; however, no further studies with this tracer have been reported to date.^706^

### 9.2 Thymidine and Thymine

#### 9.2.1 Radiosynthesis

Thymidine has been labeled with ^11^C, either at the 5-methyl position ([*methyl*-^11^C]thymidine) or in the 2-carbonyl position of the pyrimidine ring ([2-^11^C]thymidine).

**Figure 152:**
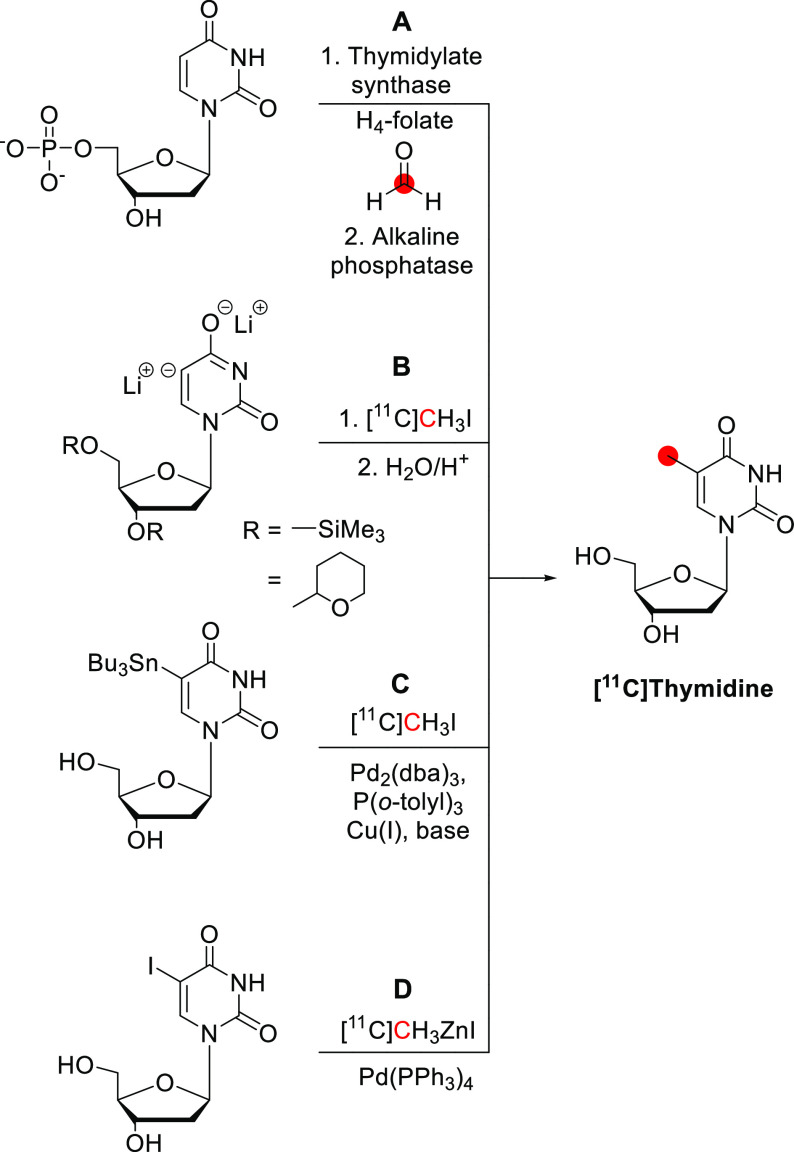
Synthesis of [*methyl*-^11^C]thymidine from different synthons [^11^C]CO_2_, [^11^C]CH_3_I, and [^11^C]CH_3_ZnI. ^11^C radionuclide position is highlighted in red.

[*Methyl*-^11^C]thymidine was first synthesized in 1972 *via* a two-step enzymatic process, beginning with the transfer of [^11^C]CH_2_O to deoxyuridine monophosphate to give [^11^C]thymidine-monophosphate, followed by dephosphorylation^36,702^ (Figure 152A. Time: 70 min, Product activity: 0.15 GBq. *A*_m_: 3 GBq/μmol for [^11^C]CH_2_O at EOB).^702^ To increase yields and *A*_m_ and allow for automation, alternative preparations utilizing [^11^C]CH_3_I were developed and used in clinical tracer productions.^702,730−733^ To increase yields and *A*_m_ and allow for automation, alternative preparations utilizing [^11^C]CH_3_I were developed and used in clinical tracer productions.^731,732,734,735^ In this approach, 5-bromo-2′-deoxyuridine derivatives bearing trimethylsilyl^730,733,736^ or tetrahydropyranyl^730,733,736^ hydroxyl protecting groups are lithiated, then ^11^C-methylated to produce [*methyl*-^11^C]thymidine after protecting group removal (Figure 152B). Time: 30–35 min. Product activity: 0.7–1.1 GBq. RCY: 19%, *A*_m_: 45 GBq/μmol.^733^ More recently, Pd(0)-mediated reactions that do not require protecting groups have been reported to produce [*methyl*-^11^C]thymidine in one step *via* Stille coupling of [^11^C]CH_3_I with *5*-tributylstannyl-*2′*-deoxyuridine^722,737^ (Figure 152C; time, 39 min; product activity of 7.9 GBq, RCY of 75%, *A*_m_ of 86–147 GBq/μmol)^722^ and *via* Negishi coupling of *5*-iodo-*2*-deoxyuridine with the ^11^C-nucleophile [^11^C]ZnCH_3_I (Figure 152D. RCY of 6%, *A*_m_ >50 GBq/μmol).^723^

**Figure 153:**
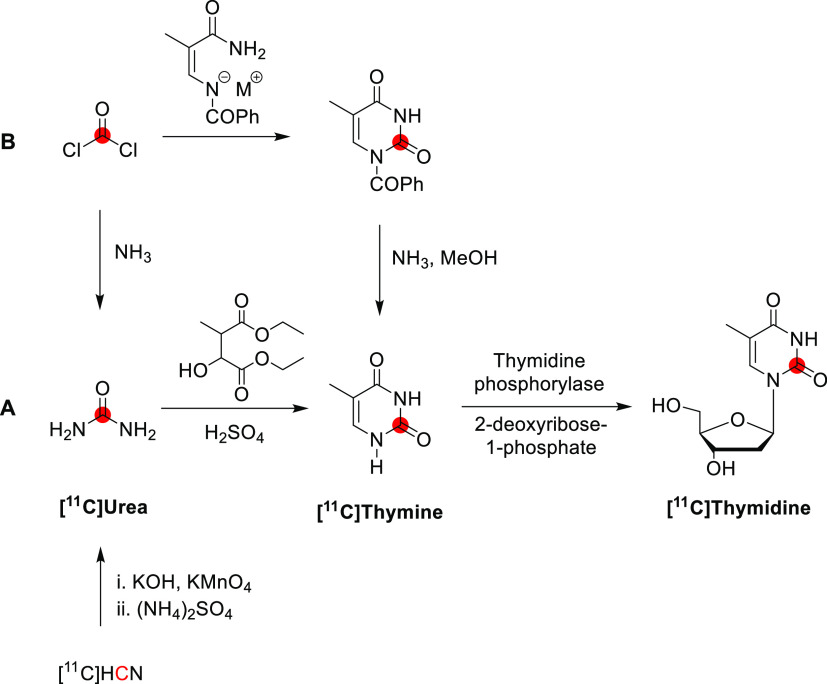
Synthesis of [*2*-^11^C]thymidine from different synthons [^11^C]COCl_2_ and [^11^C]HCN. ^11^C radionuclide position is highlighted in red.

The preparation of [*2*-^11^C]thymidine was first described in 1991 *via* a two-step process, beginning with the cyclo-condensation reaction of (derived from [^11^C]cyanide) and diethyl β-methylmalate is fuming sulfuric acid to produce [*2*-^11^C]thymine, which was subsequently converted to [*2*-^11^C]thymidine *via* enzymatic glycosylation (Figure 153A).^725^ This process has been automated,^726^ modified using alternative cyclization reagents,^738^ and using [^11^C]urea derived from [^11^C]phosgene (production time of 40–50 min, product activity of 1.5–3.3 GBq, RCY of 14%, *A*_m_ of 30–52 GBq/μmol)^724,726^ for applications in clinical tracer production.^724−726,738^

Ohkura, Seki *et al*. reported an alternative approach to the cyclocondensation reaction involving a direct reaction of [^11^C]phosgene with a dinucleophilic thymine precursor.^727,728^ In this process, the potassium salt of *β*-(*N*-benzoylamino)methacrylamide undergoes reaction with [^11^C]phosgene to produce [*2*-^11^C]thymine after amide hydrolysis (Figure 153B). Although the RCY for [*2*-^11^C]thymine formation was lower than that reported *via* [^11^C]urea (24% *vs* 38.5%, from [^11^C]COCl_2_),^724^ it was operationally simple (one-pot process).

#### 9.2.2 Preclinical Studies

[*Methyl*-^11^C]thymidine^36,702,709−712,714^ and [*2*-^11^C]thymidine^703,716,725^ have been studied preclinically *in vivo* in mice,^36,703,709^ rabbits,^702^ rats,^710−713^ and dogs^445,709,714^ to probe metabolism, DNA incorporation, and biodistribution in healthy and disease-model animals. Regardless of radiolabeling position, the tracer was rapidly catabolized *in vivo*. In dog^714^ and rat^711^ studies, less than 30% [*methyl*-^11^C]thymidine was found to remain in plasma at 5 min p.i. Despite this, the majority of activity was found to arise from [*methyl*-^11^C]thymidine incorporated in DNA or as DNA precursors.^712^

A biodistribution study of [*methyl*-^11^C]thymidine in healthy mice observed that primary direct uptake in the liver and organs are known to have high cell proliferation rates, such as the spleen and intestine.^36^ Similar uptake was observed in healthy rats; liver, spleen, and large intestines.^710^ A PET study of [*methyl*-^11^C]thymidine in rats found activity accumulation in the liver and kidneys.^712^

#### 9.2.3 Clinical Studies

In a study of the biokinetics and dosimetry of [*methyl*-^11^C]thymidine, preferential uptake of activity was observed in the liver and kidneys, with less pronounced uptake in the skeletal muscle tissue, heart wall, lungs, and salivary glands.^716^ PET studies using [*2*-^11^C]thymine and [*2*-^11^C]thymidine in healthy volunteers have been performed to validate the kinetic models used to estimate tumor proliferation^703,717^ and to develop and evaluate a system for the measurement of expired [^11^C]CO_2_ during ^11^C PET scans.^715,716^

Numerous PET studies using [*methyl*-^11^C]thymidine,^710,714,718,730,739−743^ and [*2*-^11^C]thymidine,^717,719,744−752^ have been performed to evaluate cell proliferation in human tissue tumors, including lymphoma, head and neck tumors, brain tumors, renal cell carcinoma, lung cancer, and gastrointestinal cancers.

For both labeling positions, radiometabolites account for most plasma radioactivity at just three min post-injection.^718,719^ The radiometabolic profile is influenced by the position of the radiolabel, whereas metabolism of [*methyl*-^11^C]thymidine ultimately generates radiolabeled [^11^C]*β*-AIB, the principal radiometabolite of [*2*-^11^C]thymidine is [^11^C]CO_2_. Because [^11^C]CO_2_ may be rapidly eliminated, the accumulation of labeled metabolites in tissue is minimized, thus favoring [*2*-^11^C]thymidine for cell proliferation studies. However, irrespective of the choice of ^11^C labeling position, the *in vivo* decomposition limits its utility for PET studies.^699,720,721^ To overcome this, radiolabeled thymidine analogues that are resistant to *in vivo* degradation have been developed, notably *3′’*-deoxy-*3′*-[^18^F]fluorothymidine.^753^

### 9.3 Uracil

#### 9.3.1 Radiosynthesis

**Figure 154:**
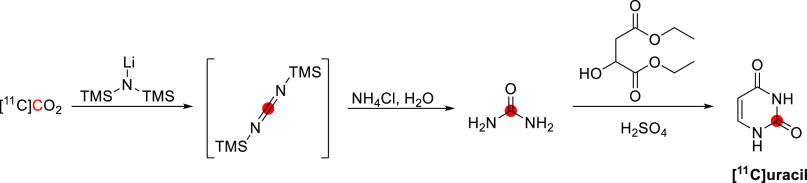
Synthesis of [^11^C]uracil using [^11^C]CO_2_. ^11^C radionuclide position is highlighted in red.

In 1997, Chakraborty *et al*.^729^ reported the synthesis of [^11^C]uracil from [^11^C]urea, based on the cyclocondensation reaction used to radiolabel the 5-methylated uracil derivative, [*2*-^11^C]thymidine.^725^ Here, a simplified means of accessing [^11^C]urea from cyclotron-produced [^11^C]CO_2_ was described, avoiding the previously reported multistep preparations involving the secondary ^11^C-labeling reagents [^11^C]cyanide,^724,754^ or [^11^C]phosgene.^755^ The subsequent conversion of [^11^C]urea to [^11^C]uracil was investigated as a model reaction (Figure 154).

In this process, [^11^C]CO_2_ was bubbled through a solution of lithium bis(trimethylsilyl)amide to produce the intermediate [^11^C]bis(trimethylsilyl)carbodiimide, which was then hydrolyzed with aqueous ammonium chloride.^729^ Following azeotropic drying, anhydrous [^11^C]urea was obtained in 55–70% RCY in 16 min. Condensation of [^11^C]urea and unlabeled urea with diethyl malate in the presence of fuming sulfuric acid produced [^11^C]uracil (carrier-added) with a nonisolated RCY of 40–75%. To date, no biological evaluation of [^11^C]uracil or its nucleoside analogue [^11^C]uridine has been reported.^729^

## 10 Peptides

The use of ^11^C for labeling peptides is, up to date, far more challenging even though peptides and AAs are alike in many ways. Their translation into PET imaging agents has been supported with specialized radiolabeling reactions in very recent years.^756^ However, the application of ^11^C-labeled peptides remains largely unexplored. A challenge for the application might be associated with slow distribution for *in vivo* studies due to rapid metabolism. The only examples of ^11^C-labeled natural peptides were methionine enkephalin (Met-enkephalin)^757,758^ and substance P^759,760^ (Table 9).

**Table 9:** Important Carbon-11 Labeled Peptides

compd	radiolabeling position	preclinical and clinical studies	synthon	*A*_M_ (GBq/μmol)	RCY	total synthesis t (min)	ref
Met5-enkephalin	*S*-*methyl*-	monkeys^757^	[^11^C]CH_3_I	nr	80%	35	(757,759)
substance P	*S*-*methyl*-	nr^a^	[^11^C]CH_3_I	7.4	35%	45	(759,760)

a nr: not reported

Met-enkephalin was initially discovered by Hughes in 1975.^761^ Met-enkephalin is a naturally occurring pentapeptide with the amino acid sequence of Tyr-Gly-Gly-Phe-Met, found in the blood at low concentrations and is present in all parts of the nervous system,^762^ mainly in the adrenal medulla and throughout the CNS.^763^ As Met-enkephalin has low bioavailability, is rapidly metabolized, and has a short half-life, Met-enkephalin, also called opioid growth factor, has an important role in pain regulation by inhibiting the release of neurotransmitters when specific opioid receptors are activated. Met-enkephalin is a potent agonist of the δ-opioid receptor and to a lesser extent, the μ-opioid receptor, with little to no effect on the κ-opioid receptor.^764^

Substance P was initially discovered in 1931 by Ulf von Euler and Gaddum.^765^ It is an undecapeptide member of the tachykinin neuropeptide family with the amino acid sequence of Arg-Pro-Lys-Pro-Gln-Gln-Phe-Phe-Gly-Leu-Met.^766^ It is found in the brain and spinal cord and is associated with inflammatory processes and pain, acting as a neurotransmitter and neuromodulator.^767^ Substance P is widely distributed in the peripheral and CNS of vertebrates.^768^ The endogenous receptor for substance P is the neurokinin 1 receptor.^768,769^

### 10.1 Met5-enkephalin

#### 10.1.1 Radiosynthesis

**Figure 155:**
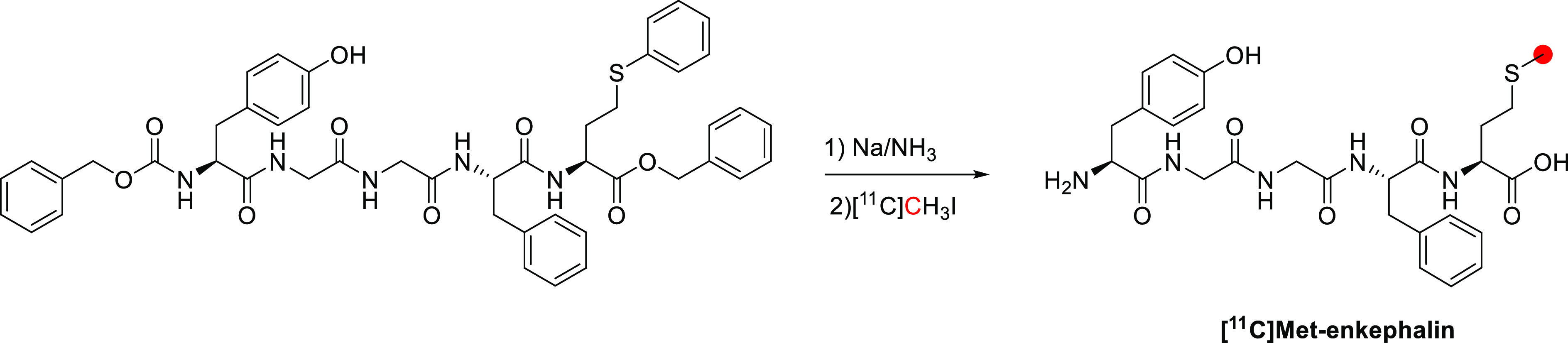
Synthesis of [^11^C]Met-enkephalin using [^11^C]CH_3_I. ^11^C radionuclide position is highlighted in red.

The peptide was radiolabeled with ^11^C by cooling a mixture of protected peptide precursor (Figure 155) and sodium in condensed ammonia to −78 °C. [^11^C]CH_3_I was added to the mixture. The reaction mixture was gently heated, and ammonia was removed using nitrogen gas flow. The resulting solid residue was dissolved in a physiological buffer and purified by HPLC. The isolated RCY, including purification, was between 50–80% and the total synthesis time was between 35–50 min.^757,759^

#### 10.1.2 Preclinical Studies

**Figure 156:**
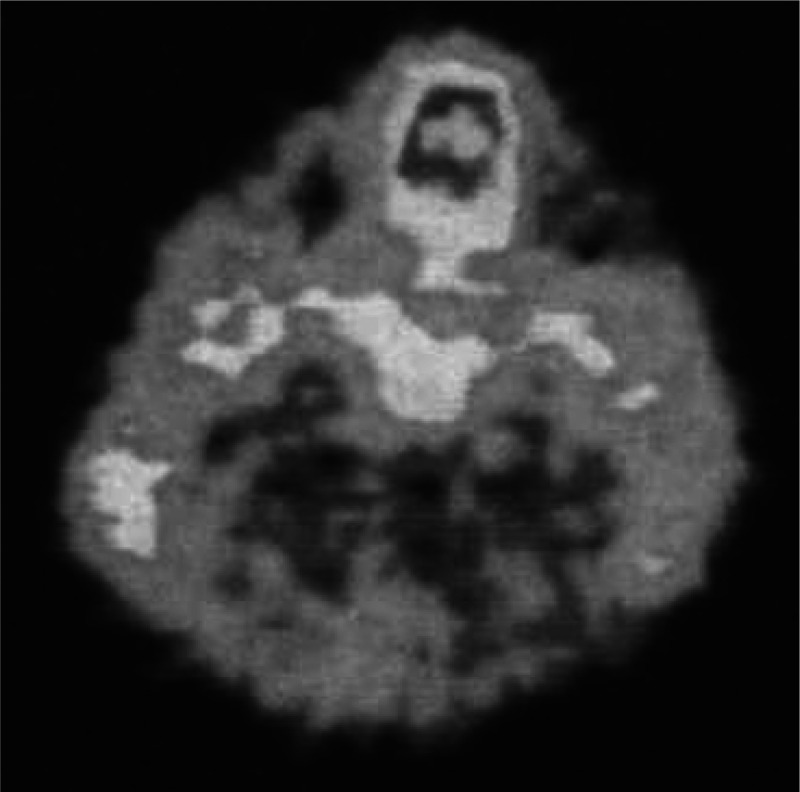
[^11^C]Met-enkephalin PET scan of a rhesus monkey’s head at the pituitary level. Reproduced with permission from ref (757). Copyright 1986 Elsevier.

[^11^C]Met-enkephalin was studied by Hartvig *et al*. in 1986 in two rhesus monkeys (*Macaca mulatta*) after an overnight fast.^757^ An intravenous catheter was inserted in each hind leg of the monkey, one for injection of radioactive dose and the other for blood sampling. After iv administration, the radioactivity, as measured with PET, rapidly reached the head of the monkeys, where it peaked within 1 min p.i. The radioactivity then rapidly declined with half-lives in the range of 2–3.5 min. The authors could not distinguish any localization of the radioactivity within the brain. High activity was observed in the liver and lower in muscle. However, no activity was observed in other tissues. Plasma and urine analysis showed that a significant fraction of the radioactivity was from [^11^C]methionine, whereas the intact [^11^C]Met-enkephalin only constituted a minor proportion of about 1–2%. [^11^C]Methionine was present in significant amounts in the plasma only a few min after administration of the radioactive dose, indicating that [^11^C]Met-enkephalin was hydrolyzed *in vivo*. Increased incorporation of radioactivity (47%, 1 h p.i.) into the plasma protein fraction, probably *via* [^11^C]methionine, was also observed. After the initial blood distribution phase, the radioactivity derived from [^11^C]Met-enkephalin remained at a high level in the pituitary for the whole period of observation, up to 60 min (Figure 156). Therefore, the authors concluded that [^11^C]methionine was probably taken up in the brain, mainly responsible for the high brain uptake.^757^

### 10.2 Substance P

#### 10.2.1 Radiosynthesis

**Figure 157:**
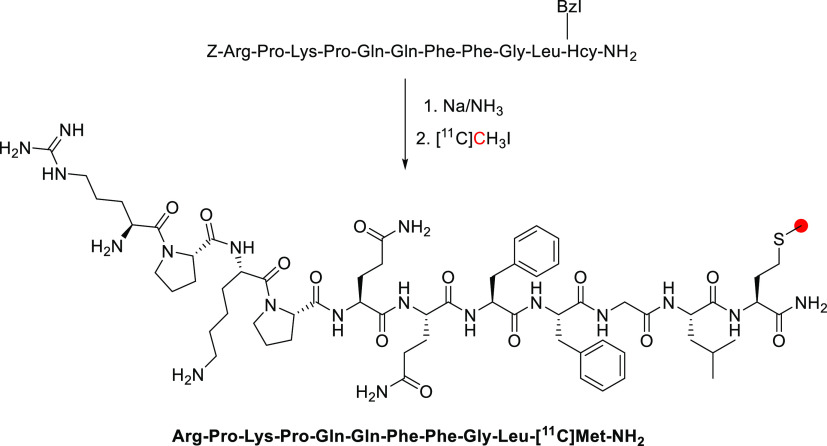
Synthesis of [^11^C]substance P using [^11^C]CH_3_I. ^11^C radionuclide position is highlighted in red.

Substance P was radiolabeled with ^11^C, similar to the preparation of [^11^C]Met-enkephalin described above from the same group (Figure 157).^759,760^ The isolated RCY, including purification, was 35% with at least 98% RPP, and the total synthesis time was between 45–60 min. The *A*_m_ of the labeled peptide was 0.37–7.4 GBq/μmol at the end of the peptide labeling.^760^

## 11 Sugars

The human body uses sugars mainly as an energy source. Glucose, in the human body, acts as the primary form of energy for the cells after being metabolized in the cytoplasm^770^ and is stored as glycogen.^771^ Fructose is mainly found in plants; however, recent reports showed that fructose is produced by the human body, following the polyol metabolic pathway, which consists of two enzymes and allows the isomerization of glucose into fructose.^555^ Galactose is an endogenous sugar and takes part in glucose metabolism. However, its metabolism is independent of glucose in the blood.^772^ Mannose is an endogenous sugar monomer and epimer of glucose. Therefore, it can be produced from glucose and converted to glucose in the human body.^773^ For these reasons, using ^11^C-labeled sugars and their amide derivatives would allow imaging of their metabolism and transport in potentially each part of the body. Furthermore, glucose itself would accurately determine metabolic rates compared to its analogues.^774^ In this chapter, the radiolabeling and application of [^11^C]glucose, [^11^C]fructose, [^11^C]galactose, and [^11^C]mannose are reviewed (Table 10).

**Table 10:** Important Carbon-11 Labeled Sugars

compd	radiolabeling position	preclinical and clinical studies	synthon	*A*_M_(GBq/μmol)	RCY	total time (min)	ref
glucose	*1*-	mice,^775^ dogs,^776−779^ macaques,^780,781^ humans^779,782−794^	[^11^C]NaCN	nr^a^	15%	38	(795)
			[^11^C]NH_4_CN	nr	30%	70	(780,782,796)
			[*1*-^11^C]CH_3_NO_2_	nr	17%	50	(797)

fructose	*1*/*6*-	rats^261^	[^11^C]CH_3_I	nr	15%	70	(560)

galactose	*1*-	nr	[^11^C]CO_2_	nr	nr	40	(798)
			[^11^C]HCN	nr	26%	70	(796)

mannose	*1*-	nr	[^11^C]HCN	nr	50%	46	(796)
			[^11^C]CH_2_NO_2_	nr	30%	50	(799)

a nr: not reported.

### 11.1 Glucose

#### 11.1.1 Radiochemistry

**Figure 158:**
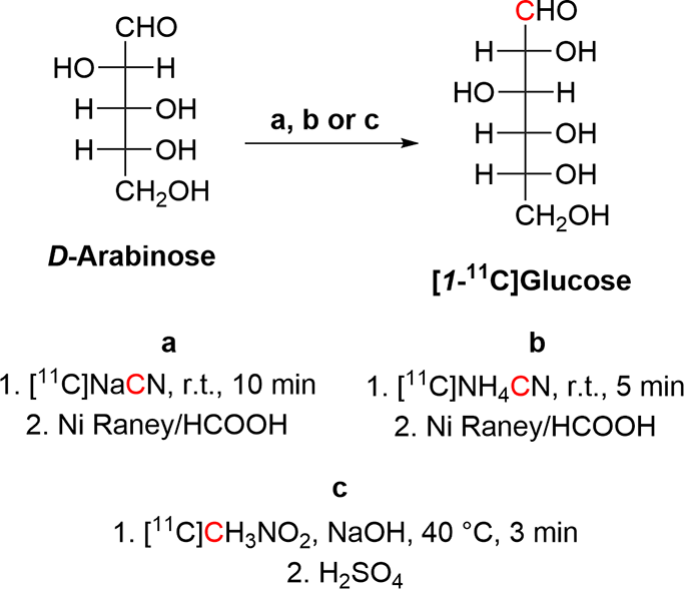
Synthesis of [*1*-^11^C]glucose using [^11^C]NaCN, [^11^C]NH_4_CN, or [^11^C]CH_3_NO_2_. ^11^C radionuclide position is highlighted in red.

Several radiochemical or photosynthetic pathways achieved the radiosynthesis of [^11^C]glucose. Radiochemical preparation was achieved using d-arabinose as starting material, which was reacted with [^11^C]NaCN (rt, 10 min)^796^ or [^11^C]NH_4_CN (rt, 5 min),^780,782,795^ forming an [*1*-^11^C]aldonitrile intermediate, which was subsequently reduced by Raney nickel in formic acid for 6–10 min at 110 °C. [*1*-^11^C]Glucose was obtained with a total synthesis time of 50–70 min and RCYs reaching 30%.^780,782,796^ A shorter reaction time (38 min) was achieved *via* a solid phase-supported reaction while keeping the RCY similar (∼15%).^795^ An alternative radiochemical route used [^11^C]CH_3_NO_2_ as a labeling agent: d-arabinose reacted with [^11^C]CH_3_NO_2_ at 40 °C for 3 min in the presence of NaOH to form a [*1*-^11^C]nitro alcohol (Figure 158). The addition of sulfuric acid returned [*1*-^11^C]glucose with an overall RCY of 14–17% within 50 min.^797^

The photosynthetic procedure was accomplished with either Swiss chard plants,^774,795^ spinach,^800^ or algae.^779^ The plant was exposed to light, and [^11^C]CO_2_ from the target was recirculated into the chamber containing the plant for 6–20 min while maintaining a temperature between rt and 40 °C to allow photosynthesis. This process yielded [*U*-^11^C]glucose (where all carbons have the same probability of being radiolabeled) with a minimum *A*_m_ of 19 GBq/μmol, RCY of 9%, with an overall processing time of 75 min.^775^ Alongside [*U*-^11^C]glucose, photosynthetic methods also produced [^11^C]fructose which, however, was easily removed from the final product by HCl hydroysis^774^ or by HPLC purification.^775^

#### 11.1.2 Preclinical Studies

The ubiquitous consumption of glucose makes [^11^C]glucose a viable probe for imaging the metabolism of a variety of organs. Studies with [*1*-^11^C]glucose and [*U*-^11^C]glucose were also performed to highlight differences in the radiolabeling position.

Initial assessment of the cerebral metabolic rate (CMR_glc_) and BBB glucose flux was done by 48 min scans of macaques after [^11^C]glucose injection.^780^ Analysis of the results returned a net utilization fraction of 0.12.^780^ A more accurate description of [^11^C]glucose biodistribution was then reported after *ex vivo* analysis of male mice.^775^ The highest activity was detected in the brain, where the radiopharmaceutical quickly peaked, plateaued, and stayed constant for 20 min. These studies also highlighted increasing activity as a function of time by the pancreas, while all other organs cleared it.^775^

The ability to differentiate metabolic states with [^11^C]glucose was also studied.^776,777^ Twenty-two mongrel dogs with diverse metabolic states (fasting, hyperinsulinemic, enhanced glycolysis, increased glycogen production) were injected and scanned for 1 h while collecting coronary blood for metabolite analysis.^776,777^ The information retrieved allowed the accuracy of the various metabolic states at the myocardium level.^776,777^ Following studies also revealed a higher accuracy of [^11^C]glucose in myocardial assessment compared to [^18^F]FDG.^778^ No significant difference was detected after comparing [^11^C]glucose with [*U*-^14^C]glucose to determine brain influx and metabolism.^779^

Preclinical studies also focused on determining the most reliable compartmental model to describe glucose influx and metabolism in the brain. While the studies mentioned above related to a three-compartmental model,^801^ the use of a four-compartmental model (where the fourth rate constant considers the ^11^C-metabolite egress) showed to be a more accurate estimation, as confirmed by PET scanning and blood metabolite analysis on four adult male macaques in hypo- and normoglycemic conditions.^781^

#### 11.1.3 Clinical Studies

**Figure 159:**
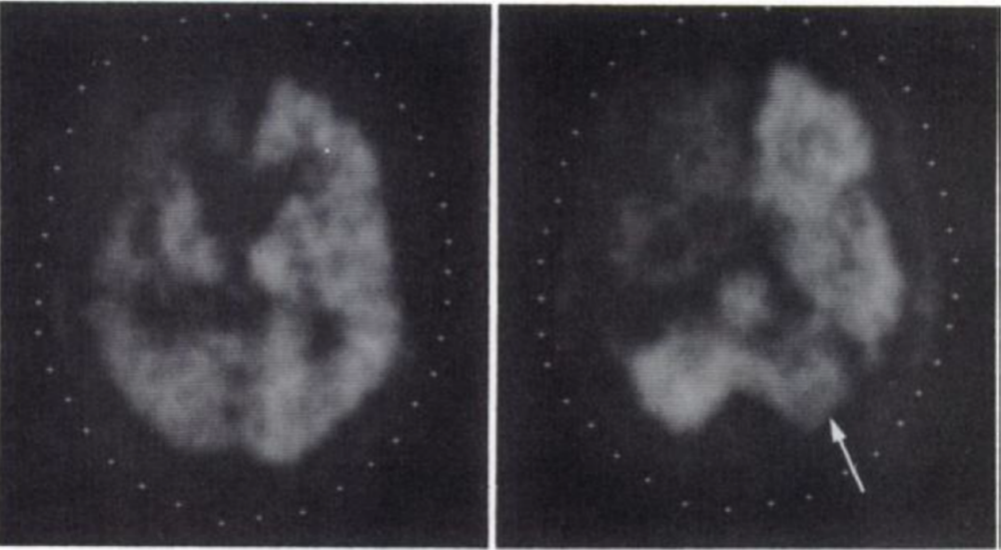
[^11^C]Glucose PET images of a patient suffering brain infarction. Darker areas (right frontal and temporal lobes) indicate a lower [^11^C]glucose metabolism. Reproduced with permission from ref (779). Copyright 1983 Society of Nuclear Medicine. This work is licensed under a Creative Commons Attribution 4.0 International License (https://creativecommons.org/licenses/by/4.0/).

Clinical studies focused on assessing brain glucose metabolism in different metabolic states. Initial assessment of [^11^C]glucose distribution was done on seven healthy volunteers injected with [^11^C]glucose and scanned for 24 min while simultaneously collecting blood samples for metabolite analysis.^783^ The data analysis returned a rCMR_glc_ consistent with known brain metabolic rates.^783^ Moreover, the images acquired during scanning a patient who suffered brain infarction clearly show the differences in metabolic rate between the infarcted and noninfarcted areas (Figure 159).^779^ Radiation dosimetry studies after [^11^C]glucose administration was confirmed to be small and comparable to other clinically-available radiopharmaceuticals, limiting the radiation risks associated with its use.^784^

The effect of insulin-dependent diabetes mellitus was investigated by scanning six and eight nondiabetic patients while being infused with insulin (to maintain the same insulin level as healthy controls).^785^ The results potentially indicate a higher rate of nonoxidative glucose metabolism in diabetic patients.^785^ PET scanning of five healthy male individuals aged 22–40 in hyperglycemic conditions instead highlighted a slight increase in brain glucose metabolism compared to normoglycemia, with the white matter being the most affected part.^786^ A nonlinear relationship between glucose blood levels and brain glucose metabolism was also found.^786^

The ability of [^11^C]glucose to image neuronal activity within different brain regions was then employed to identify aberrant patterns associated with schizophrenia. The hypothesis of a hypofrontality pattern, in which the blood flow in the frontal cortex is decreased, was disproven by Widen *et al*. after studying a cohort of six young schizophrenic patients.^787^ The scanning of a larger cohort (20 patients) could not identify a typical aberrant pattern.^788^ Following studies, however, identified three subtypes of schizophrenia based on glucose metabolism patterns: (i) hypofrontal, with lower activity retention in the frontal cortex (−38% compared to control); (ii) hypoparietal, with a 26% decrease in glucose metabolism in the parietal cortex; and (iii) normal, which did not have any significant changes from control.^789^ To each subtype, a behavioral pattern was associated, with hypofrontal being more blunted and flat, hypoparietal being hallucinating and delusional, and the normal subtype having a mix of both.^789^ Differences in glucose metabolism were also highlighted in depressed patients, with a 35% decrease in overall brain activity retention compared to control subjects.^790^

The detection of other neurological diseases, such as temporal lobe epilepsy, encephalitis, and brain tumors, was also possible with [^11^C]glucose.^791^ The scanning of a 10-year-old encephalitis patient with [*U*-^11^C]glucose, instead, showed a significant white matter low attenuation, indicating a regression in the cortical tissue, which, however, completely disappeared after recovery from the disease.^792^ Regarding the detection of glioma brain tumors, clinical studies were carried out on 40 patients.^793^ The detection of other brain tumors such as astrocytoma (grade II and anaplastic), oligoastrocytoma, or meningiomas, instead, was shown to be limited compared to other clinically-available radiopharmaceuticals (*e.g*., [^11^C]methionine), especially for low-grade tumors.^794^

Besides evaluating different metabolic states and diseases, clinical studies with [^11^C]glucose also focused on disclosing the radiopharmaceutical stability and the formation of metabolites.^782,783^ In particular, [^11^C]CO_2_ egress was determined by blood metabolite analysis on healthy male volunteers.^782^ [*1*-^11^C]Glucose showed to be metabolically more stable than [*U*-^11^C]glucose, having a slower and delayed [^11^C]CO_2_ release.^782^ The slower egress of [*1*-^11^C]glucose also positively impacts distinguishing between white and grey matter in the brain.^783^

### 11.2 Fructose

#### 11.2.1 Radiosynthesis

[^11^C]Fructose production was mainly achieved as a byproduct in the radiolabeling of [^11^C]glucose and proceeded *via* photosynthesis. Light-deprived leaves of Swiss chards were exposed to [^11^C]CO_2_ and subsequently to light to trigger the photosynthetic production of [^11^C]glucose, [^11^C]fructose, and [^11^C]sucrose for 20 min, which were then alcohol extracted from the leaf. The resulting radioactive mixture of saccharides contained 30–32% of [^11^C]fructose. The various products were then separated using ion-exchange resins and specific carriers.^557,558^ This synthetic method was subsequently fully automated.^559^

**Figure 160:**
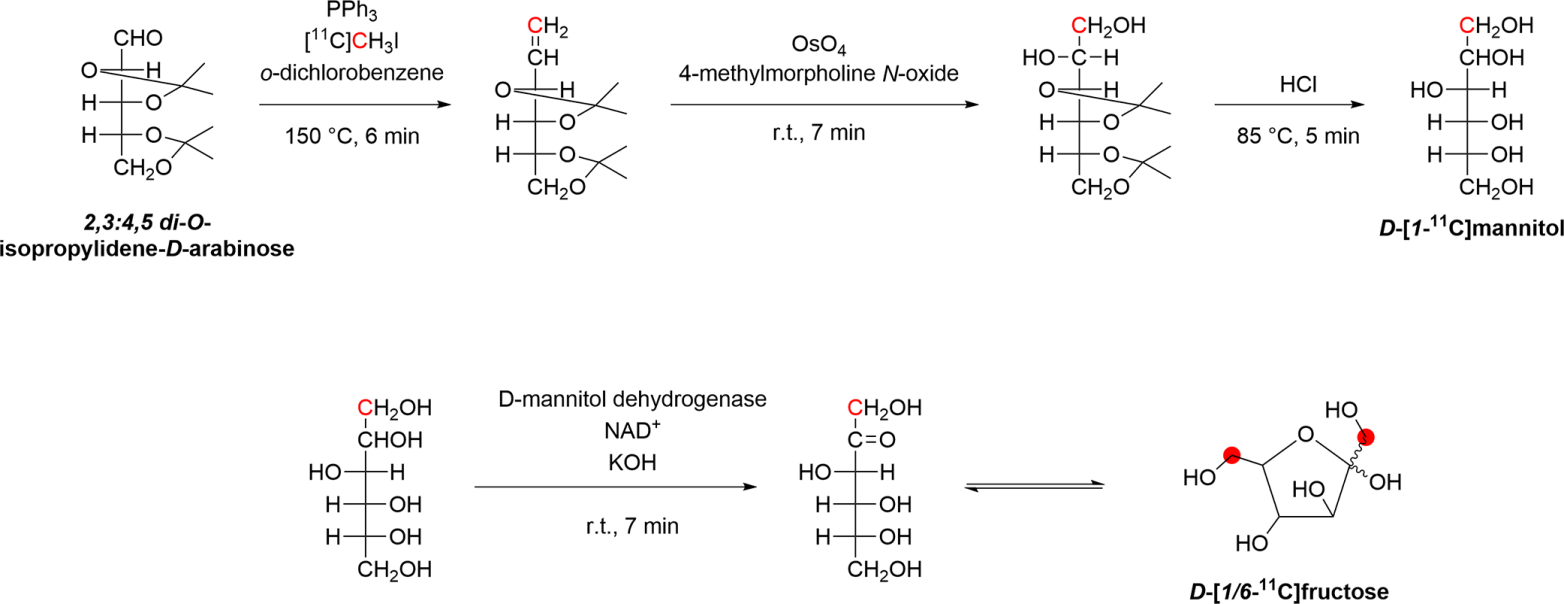
Enzymatic synthesis of *d*-[^11^C]fructose using [^11^C]CH_3_I. ^11^C radionuclide position is highlighted in red.

An alternative radiolabeling pathway was developed to directly produce [^11^C]fructose (Figure 11.4). This pathway required the reaction of [^11^C]CH_3_I with *2*,*4*:*4*,*5* di-*O*-isopropylidene-*d*-arabinose in the presence of triphenylphosphine in *o*-dichlorobenzene, yielding *4*,*4*:5,*6*di-*O*-isopropylidene-*d*-[*1*-^11^C]arabino-hex-*1*-enitol within 6 min and subsequent conversion into *d*-[*1*-^11^C]mannitol by addition of osmium tetroxide and deprotection by 6 M HCl (Figure 160). *d*-[*1*-^11^C]Mannitol was then converted into [^11^C]fructose by enzymatic reaction with d-mannitol dehydrogenase in the presence of NAD^+^ and KOH for 7 min (Figure 160). [^11^C]Fructose was produced with a total processing time of 70 min and with a RCY of 15%.^560^

#### 11.2.2 Preclinical Studies

Clinical studies were carried out using pregnant Wistar rats on their 16–19^th^ day of gestation. The study aimed to disclose the crossing of the placenta of several endogenous compounds, including [^11^C]fructose. A mixture of [^11^C]fructose and [^11^C]glucose (1.85–7.40 MBq) was injected, followed by culling at regular time intervals (1, 5, 10, and 30 min) and tissue analysis. The concentration of ^11^C-sugars in fetal and maternal tissues was comparable and increasing over time, reaching values higher than 1 within 30 min.^261^

### 11.3 Galactose

#### 11.3.1 Radiosynthesis

**Figure 161:**
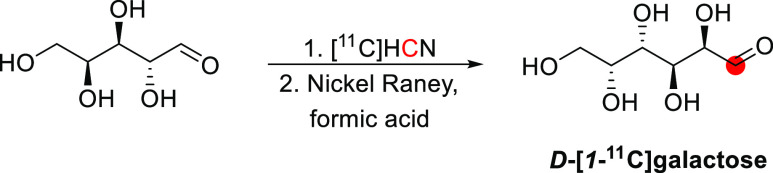
Synthesis of *d*-[*1*-^11^C]galactose using [^11^C]HCN. ^11^C radionuclide position is highlighted in red.

Galactose was only labeled from position 1 and synthesized using natural plants.^798^ The first radiolabeling of galactose with carbon-11 was done as biosynthesis from marine algae (*Girgartina stellata*). Synthesis was started with [^11^C]CO_2_ and completed in 40 min.^798^ Labeled galactose was separated by using LC. Another method which includes the synthesis of *d*-[*1*-^11^C]galactose, starts from [^11^C]HCN and *d*-lyxose, and then continues with the reduction of [*1*-^11^C]aldonitriles by Raney alloy in formic acid (Figure 161).^796^ Total synthesis time was 70 min with a RCY of 26%.

### 11.4 Mannose

#### 11.4.1 Radiosynthesis

**Figure 162:**
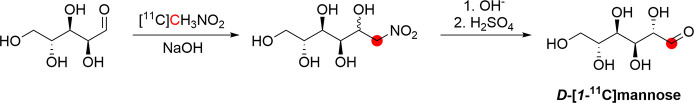
Synthesis of *d*-[*1*-^11^C]mannose using [^11^C]CH_2_NO_2_. ^11^C radionuclide position is highlighted in red.

The first synthesis was reported in 1985 for the *d*-[*1*-^11^C]mannose by starting from [^11^C]HCN in NaCN solution and then reducing d-arabinose with Raney alloy in formic acid made in the same solution.^796^ Final product was purified using HPLC and the total synthesis time without purification was 46 min with a RCY of 40–50%. It was also shown that the yield depends on pH, and in time glucose and mannose interchange affects the yield ratio. *d*-[*1*-^11^C]Mannose was also prepared from [^11^C]nitromethane in 50 min with a RCY of 25–30% (based on [^11^C]nitromethane) (Figure 162).^799^

## 12 Other Compounds

Besides the compounds described below (Table 11), other natural exogenous compounds, less common, have been labeled with carbon-11. Among these, [^11^C]cinnamic acid^802^ (oil of cinnamon), [^11^C]1,3-diphenylurea^803,804^ (a cytokinin found in coconut milk), [^11^C]benzothiazole^153^ (food additive), [^11^C]benzaldehyde^805^ (food additive), [^11^C]octanal^805^ (citrus oil), and [^11^C]heptanoic acid^531^ (additive in cigarettes). However, these compounds have been described only in one or two studies and not evaluated *in vivo*. Thus, they are not discussed in this review.

**Table 11:** Other Carbon-11 Labeled Compounds

compd	radiolabeling position	preclinical and clinical studies	synthon	*A*_M_ (GBq/μmol)	RCY	total time (min)	ref
4-aminobenzoic acid	*carbonyl*-	mice,^850^ rats,^851^ rabbits,^851^ humans^851^	[^11^C]CO_2_	407	14%	nr^a^	(852)
benzoic acid	*carbonyl*-	rabbits,^810,853^ dogs^854^	[^11^C]CO_2_	nr	80%	10	(810,853,855)
cadaverine	*1*-	rats^815^	[^11^C]NaCN	nr	77%	63	(815)
putrescine	*1*-	mice,^856^ rats,^857^ humans^858−860^	[^11^C]HCN	>52	20%	50	(858−860)

choline	*N*-*methyl*-	rats,^817,861^ rabbits,^862,863^ monkeys,^864^ humans^817,862,865−870^	[^11^C]CH_3_I	>133	>98%	25	(862)
			[^11^C]CH_3_OTf	>29.6	85%	15	(871)

daunorubicin	*2*-*acetyl*-	rats^820,821,872^	[^11^C]CH_2_N_2_	1.111	3%	53	(820)
	*4*-*methoxy*-	nr	*l*-[*methyl*-^11^C]methionine	nr	1%	75	(821)

erythromycin lactobionate	*N*-*methyl-*	humans^873^	[^11^C]CH_2_O	nr	12%	42	(822,823)
glycerol	nr	nr	[^11^C]CO_2_	1.08 × 10^–4^	nr	80	(798)
hippuric acid	*carbonyl*-	mice,^874^ rabbits^855^	[^11^C]CO_2_	nr	8%	nr	(874)

lactic acid	*1*-	dogs,^875^ rabbits^876^	[^11^C]KCN	nr	40%	90	(877)
			[^11^C]NaCN	nr	>80%	45	(878)
			[^11^C]HCN	nr	80%	40	(241)
	*2*-	nr	[^11^C]CO(CH_3_)_2_	nr	50%	150	(877)
	*3*-	*rats,^879^* dogs^880^	[^11^C]CO(CH_3_)_2_	nr	50%	150	(877)
			[^11^C]CH_3_I	0.02	26.5%	40	(881)

*N*-acetyl-leukotriene E4	*N*-*acetyl*-	rats,^832,882,883^ pigs,^883^ monkeys^883^	[*1*-^11^C]CH_3_COCl	2	1.3%	50	(883,884)
*N*-methyltaurine	*N*-*methyl*-	nr	[^11^C]CH_3_I	nr	nr	3	(885)
oxalic acid	*carbonyl*-	nr	[^11^C]CN^–^	nr	70.8%	nr	(886)
paclitaxel	*carbonyl*-	nr	[*1*-^11^C]C_6_H_5_COCl	0.0499	7%	38	(887)

phenylethanolamine	*1*-	nr	[^11^C]HCN	130.1	4%	50	(888)
			[^11^C]CH_3_NO_2_	56	50%	40	(685)

phenylpyruvic acid	*3*-	nr	[^11^C]CO_2_	nr	40%	40	(428)

pyruvic acid	*1*-	rats,^889,890^ rabbits,^891^ humans^891^	[^11^C]CO_2_	nr	80%	35	(891)
	*3*-	mice,^892^ pigs^893−897^	[^11^C]CH_3_I	nr	73%	35	(898)

salicylic acid	*1*-	mice,^899^ dogs^531^	[^11^C]CO_2_	23.5	7.3%	nr	(899)

salvinorin A	*acetoxy-*	baboons^813^	[^11^C]CH_3_COCl	27.8	10%	40	(813)
	*O*-*methyl-*	nr	[^11^C]CH_3_I	159.1	72%	nr	(900)

urea	*carbonyl*-	humans^901^	[^11^C]COCl_2_	6.475	35%	nr	(902)
			[^11^C]KOCN	129.5	95%	20	(903)
			[^11^C]CO_2_	nr	70%	16	(729)

uric acid	*6*-/(*carbonyl*)-	rats^904^	[^11^C]COCl_2_	142	36%	30	(904)

a nr: not reported.

Although the compounds below could not fit in any of the other categories described in sections 1–11, they are crucial for humans, and their labeling with carbon-11 has led to relevant outcomes:

- 4-Aminobenzoic acid (PABA) is an intermediate in folate synthesis by fungi, plants, and bacteria, including those found in the human intestinal tract.^806^ In humans, although PABA was considered a “vitamin Bx”, it is no longer recognized as a vitamin because it is generated through the microbiome.^807^ PABA is metabolized in the liver by phase II conjugation *viaN*-acetyltransferase 1 and glycine conjugation. PABA and all its metabolites are characterized by fast renal excretion. Thus, carbon-11 PABA could be used for PET renal imaging and is an excellent candidate for imaging bacterial infections.^808,809^
- Benzoic acid, a natural exogenous compound, is produced as an intermediate in synthesizing secondary metabolites by esterifying with various alcohols. The sodium salt of benzoic acid, sodium benzoate, is a food preservative widely used in food manufacturing.^810^ Benzoate salts and esters are quickly detoxified in the liver by conjugating various biomolecules in the human body. The conjugation of benzoate with glycine generates hippuric acid, quickly excreted by the kidneys. Considering benzoates’ quick metabolism and excretion, using carbon-11 labeled analogues, is hypothesized to quantify hepatic and renal functions.^810^
- Putrescine and cadaverine, natural exogenous compounds, are foul-smelling diamines produced by breaking down amino acids in living and dead organisms. Putrescine is produced by the enzymatic decarboxylation of ornithine-by-ornithine decarboxylase (ODC). It is found in healthy living cells, serving as a precursor to polyamines, spermidine, and spermine. Because these polyamines are protonated under physiological conditions, they can interact with nucleic acids and are involved in cell growth and viability.^811^ While ODC activity and putrescine levels are low in the normal brain; they are elevated in a wide variety of rapidly growing tissues, including primary and metastatic brain tumors. Hence, [^11^C]putrescine was investigated as a potential biomarker of cell division and growth.^812,813^ Ultimately, the diagnostic utility of [^11^C]putrescine was limited by a lack of specificity, with uptake resulting primarily from BBB breakdown. Cadaverine is formed during the putrefaction of animal tissue by bacterial lysine decarboxylase. Although mammalian cells do not possess this enzyme, cadaverine is produced in small quantities by the action of ornithine decarboxylase on intracellular lysine.^814^ [^11^C]Cadaverine has been radiolabeled and studied, along with other aliphatic diamines, to examine the relationship between their molecular structure and *in vivo* biodistribution.^815^
- Choline is a nutrient obtained through dietary intake and endogenous synthesis *via* the hepatic phosphatidylethanolamine *N*-methyltransferase pathway.^816^ Choline plays an essential role as a precursor for synthesizing the neurotransmitter acetylcholine and the two most abundant phospholipids in the brain, phosphatidylcholine and sphingomyelin. It is also implicated in other diverse functions such as lipid transport (lipoproteins), cell-membrane integrity and signaling, and methyl-group metabolism as a significant source of methyl groups in the diet.^816^ Moreover, choline is oxidized to betaine aldehyde,^817^ which is then converted into betaine, a direct methyl group donor in the methionine cycle for the formation of S-adenosylmethionine.^818,819^ The expression of choline kinases is upregulated during carcinogenesis to keep up with the demands of the synthesis of phospholipids in their cellular membranes. Therefore, choline transport is closely associated with cell growth. Hence, [^11^C]choline has been used to detect various cancers.
- Carbon-11 antibiotics could be used as a tracer for a site of infection and as a measure of the tissue distribution of the antibiotic in humans. So far, only one natural antibiotic has been labeled and evaluated *in vivo* for both the above reasons, Erythromycin lactobionate, a macrolide that inhibits protein synthesis. Another compound, daunorubicin, which is classified as an antibiotic but used as a chemotherapeutic compound, has also been labeled.^820−823^ Daunorubicin, an exogenous compound, is an anthracycline antibiotic initially isolated in the 1950s from a new strain of*Streptomyces peucetius*bacteria.^824,825^ However, it is mainly used to treat various types of cancers, especially leukemias, through various mechanisms that include anti-mitotic and anti-cytotoxic activities.^826^
- Glycerol, an endogenous compound, is trivalent alcohol produced by the human white adipose tissue (WAT) when an excess of glucose is present.^827^ Glycerol is usually stored in the body in the adipose tissue in the form of triglycerides and phospholipids, which are metabolized by the liver as a source of energy. Glycerol is also the substrate of hepatic gluconeogenesis.^827^
- Hippuric acid, an endogenous compound, has been a major human metabolite for years, found in urine and formed from benzoic acid and glycine. Hippuric acid can appear in humans as an excretory product from natural or unnatural sources.^828^ Levels of hippuric acid rise with fruit juice, tea, and wine, rich in phenolic compounds.^829^ The phenols are first converted to benzoic acid and then to hippuric acid and excreted in the urine. Hippuric acid is also associated with inborn errors of metabolism such as phenylketonuria, propionic acidemia, and tyrosinemia I.^830^
- Lactic acid, an endogenous compound, is produced by reducing pyruvate in a reversible process catalyzed by l-lactate dehydrogenase, which is predominantly located in the cytosol of human cells. Lactate removal occurs *via* oxidation back to pyruvate, followed by oxidation to CO_2_ to produce energy or gluconeogenesis to produce glucose.^831^ The role of lactic acid as an energy source has prompted preclinical studies using [^11^C]lactic acid to probe *in vivo* lactate metabolism.
- *N*-Acetyl-leukotriene E_4_ is an endogenous metabolite biologically less active than cysteinyl leukotrienes but follows the same elimination pathway.^832^
- *N*-Methyltaurine, a natural exogenous compound mainly found in red algae, is a derivative of endogenous neuroprotector taurine.^833^
- Oxalic acid, an endogenous compound, is synthesized in erythrocytes and the liver through the metabolism of glycine, glyoxylate, and ascorbic acid.^834^ Oxalic acid is a metabolic end-product that is eliminated unchanged and primarily through the kidneys by glomerular filtration and tubular secretion.^835^
- Paclitaxel, a natural exogenous compound, works by interference with the normal function of microtubules during cell division and is a chemotherapy medication used to treat several types of cancer, including ovarian cancer, esophageal cancer, breast cancer, lung cancer, Kaposi’s sarcoma,^836^ cervical cancer, and pancreatic cancer.^837,838^
- Phenylethanolamine, an endogenous compound, acts as a trace amine-associated receptor 1 agonist and a monoaminergic modulator. It is distributed at very low concentrations throughout the central and peripheral nervous systems and has a crucial role in neurotransmission and neuromodulation.^839^
- Phenylpyruvic acid is the α-keto acid of phenylalanine,^840^ present in high levels in the urine of individuals with phenylketonuria due to the lack of phenylalanine hydroxylase.^841^
- Pyruvic acid, an endogenous compound, is essential in most biological pathways and can be converted from alanine to lactic acid by enzymes.^842^
- Salicylic acid is a major metabolite of aspirin, with a mechanism of action still poorly understood.^843^ Aspirin is readily hydrolyzed to salicylic acid in the blood and liver,^844^ which can either be directly excreted (1–31%) or undergo conjugation reactions generating the major metabolite salicyluric acid (20–65%)^845^ and ether and ester glucuronides of salicylic acid (1–42%). Salicylic acid can also be metabolized to *2*,*5*-dihydroxybenzoic acid (*2*,*5*-DHBA; gentisic acid) and *2*,*3*-dihydroxybenzoic acid (*2*,*3*-DHBA; pyrocatechuic acid) *via* the CYP450 enzymes.^844^ Although salicylic acid is a poor inhibitor of cyclooxygenases COX1 and COX2, it suppresses prostaglandin synthesis.^846^
- Salvinorin A is the main active principle of*Salvia divinorum*from the mint family and possesses a chemical structure of a neoclerodane diterpene. It acts as a potent hallucinogen in the body by agonistically binding κ opioid receptors, although its effects only last for a few minutes.^812,813^ Due to the strong perceptive distortion provoked, salvinorin A and dried *Salvia divinorum* leaves quickly became recreational drugs.^812,813^ Biodistribution studies of an ^11^C-labeled analogue of salvinorin A would help understand its exact action mechanism and map κ-opioid receptors in the brain.
- Urea is considered the main nitrogenous waste product of metabolism and is mainly derived from protein catalysis. In the human body, it is excreted through the kidney in urine, and its plasma concentration is considered a marker of renal function.^847^
- Uric acid is a normal component of urine, discovered by Carl Wilhelm Scheele in 1776 in kidney stones. It is the final oxidation product of purine nucleotides metabolism,^848^ and a high blood concentration could lead to gout, formation of ammonium acid urate kidney stones, and diabetes.^849^

### 12.1 *4*-Aminobenzoic Acid

#### 12.1.1 Radiosynthesis

**Figure 163:**
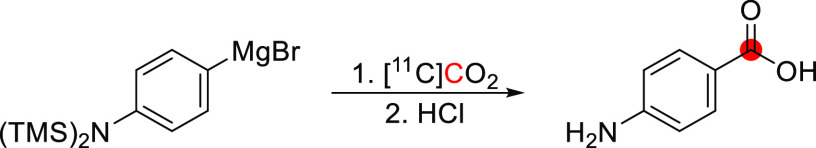
Synthesis of [^11^C]PABA using [^11^C]CO_2_. ^11^C radionuclide position is highlighted in red.

[^11^C]PABA has been prepared by reacting the commercially available Grignard precursor with [^11^C]CO_2_. [^11^C]PABA was isolated with a RCY of 35%, RCP >99%, and *A*_m_ of 30.34 ± 9.55 GBq/μmol at EOS (Figure 163).^850^ Holt *et al*.,^852^ following the method above, developed by Mutch *et al*.,^850^ produced [^11^C]PABA by conforming to cGMP requirements. [^11^C]PABA was produced with a RCY of 14%, high RCP, and *A*_m_ of 407 GBq/μmol as a sterile, pyrogen-free solution suitable for injection.^852^

#### 12.1.2 Preclinical Studies

**Figure 164:**
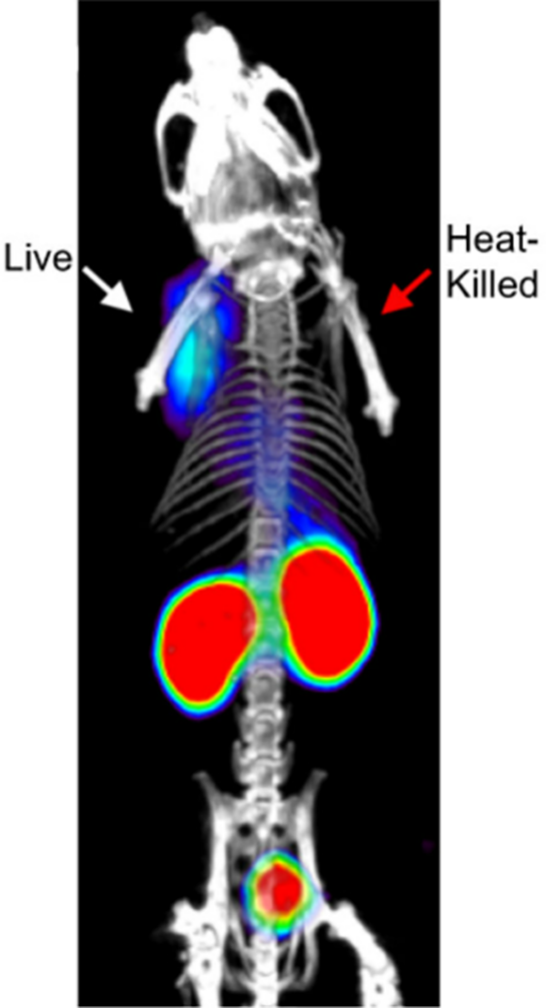
[^11^C]PABA microPET/CT image in a murine myositis model. Live = area inoculated with live *E. coli*; Heat-killed = area inoculated with 10-fold greater (10×) heat-killed bacteria. Reproduced with permission from ref (850). Copyright 2018 American Chemical Society.

[^11^C]PABA has been evaluated in mice,^850^ rats,^850^ and rabbits.^851^ In mice, [^11^C]PABA was evaluated in CBA/J female mice after iv injection to distinguish between infection and sterile inflammation in a murine model of acute bacterial infection. [^11^C]PABA showed high accumulation in the infected left shoulder, with low accumulation, slightly above background, in the heat-killed inoculated right deltoid (Figure 164).^850^

**Figure 165:**
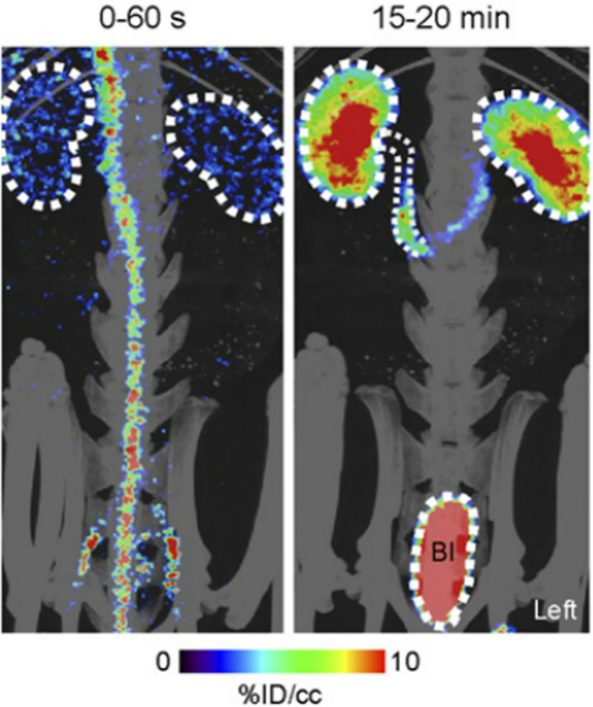
[^11^C]PABA PET/CT maximum-intensity-projection images in healthy Wistar rats. BI = bladder. Reproduced with permission from ref (851). Copyright 2020 Society of Nuclear Medicine and Molecular Imaging. This work is licensed under a Creative Commons Attribution 4.0 International License (https://creativecommons.org/licenses/by/4.0/).

**Figure 166:**
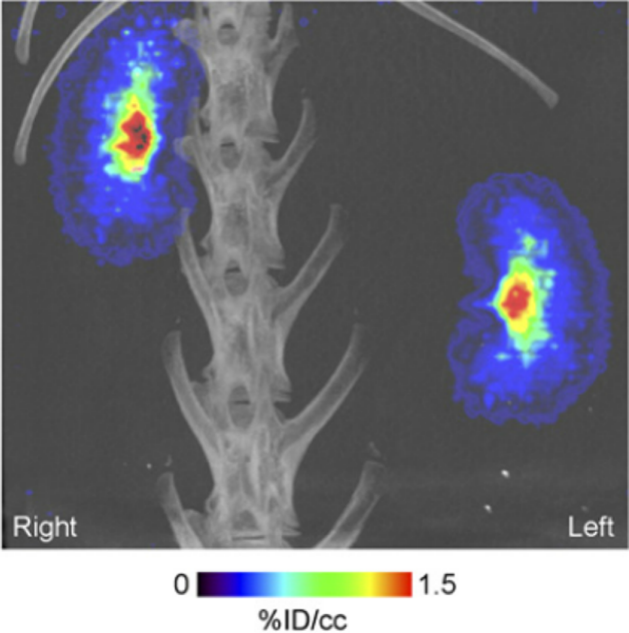
[^11^C]PABA PET/CT renal imaging in healthy rabbits (20 min p.i.). Reproduced with permission from ref (851). Copyright 2020 Society of Nuclear Medicine and Molecular Imaging. This work is licensed under a Creative Commons Attribution 4.0 International License (https://creativecommons.org/licenses/by/4.0/).

[^11^C]PABA in healthy Wistar rats (3 females, 1 male) (Figure 165) and healthy New Zealand white rabbits (2 females) (Figure 166) was evaluated by a dynamic PET demonstrating a fast renal excretion with a very low background signal, rapid and high accumulation in the renal cortex, followed by fast clearance through the pelvicalyceal system.^851^

#### 12.1.3 Clinical Studies

**Figure 167:**
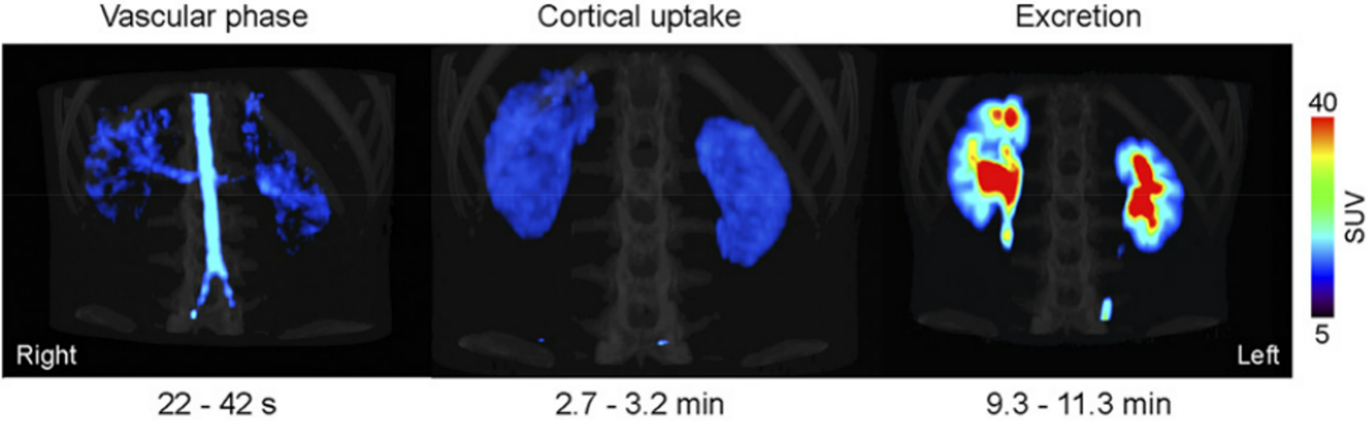
[^11^C]PABA PET/CT renal imaging in a healthy human subject. Reproduced with permission from ref (851). Copyright 2020 Society of Nuclear Medicine and Molecular Imaging. This work is licensed under a Creative Commons Attribution 4.0 International License (https://creativecommons.org/licenses/by/4.0/).

[^11^C]PABA has been evaluated in three healthy volunteers (2 male, 1 female) at least 18 y old (age range, 23–30 y).^851^ [^11^C]PABA PET was safe and well-tolerated, without any adverse or clinically detectable pharmacologic effects. The cortex was delineated on PET, and the activity gradually transited to the medulla and then pelvis with a high spatiotemporal resolution with low radiation exposure (Figure 167).^851^

### 12.2 Benzoic Acid

#### 12.2.1 Radiochemistry

**Figure 168:**
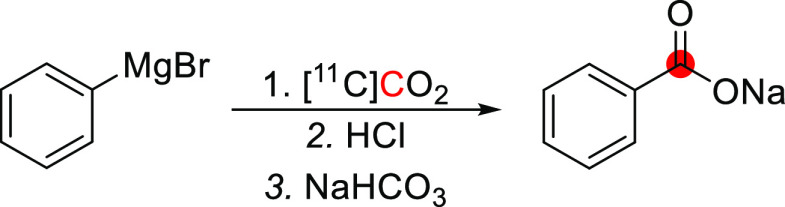
Synthesis of sodium [^11^C]benzoate using [^11^C]CO_2_. ^11^C radionuclide position is highlighted in red.

[^11^C]Sodium benzoate was synthesized by carbonation of phenylmagnesium bromide with [^11^C]CO_2_, followed by acidic hydrolysis with HCl and extraction with sodium bicarbonate (Figure 168).^810,853,855^ The product was obtained within 10–20 min with a RCY of 60–80% at EOB.^810,853,855^ The simplicity made [^11^C]benzoic acid the substrate of choice for carboxylation and carbonylation studies. Several methods have been developed in the past year using [^11^C]benzoic acid and [^11^C]benzoate as pilot compounds, such as fluoride-mediated desilylative methods,^615,905^ copper-catalyzed labeling of arylstannanes,^906^ palladium-catalyzed ^11^C-carbonylation with [^11^C]CO,^907^ and ^11^C-carboxylation of arylboronic esters *via* copper catalysis.^908^

#### 12.2.2 Preclinical

[^11^C]Sodium benzoate was used to evaluate renal function in dogs,^810,853^ and rabbits,^854^ showing a rapid accumulation (within 10 min) and extended retention of the radiopharmaceutical in the kidneys, suggesting the potential use of the tracer to visualize the renal parenchyma.^853−855^ Sodium [^11^C]benzoate was also successfully employed to image a dog’s osteogenic sarcoma.^853^

### 12.3 Cadaverine and Putrescine

#### 12.3.1 Radiosynthesis

**Figure 169:**
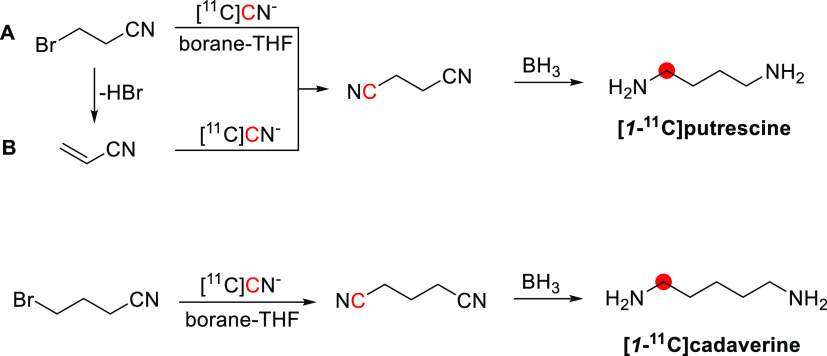
Synthesis of [*1*-^11^C]putrescine and [*1*-^11^C]cadaverine using [^11^C]CN^–^.^11^C radionuclide position is highlighted in red.

[*1*-^11^C]Putrescine was first synthesized by Winstead *et al*. in 1980 in a two-step process involving the reaction of *3*-bromopropionitrile with carrier-added [^11^C]NaCN to form [^11^C]succinonitrile, followed by reduction using borane-THF (Figure 169A).^815^ The product was precipitated as the hydrochloride salt and used without further purification (reaction time, 70 min; RCY, 57% based on [^11^C]NaCN).^815^ [*1*-^11^C]Cadaverine was produced under the same conditions, starting from 3-bromobutyronitrile (reaction time, 63 min; RCY, 77% based on [^11^C]NaCN).

A similar approach was used by Jerabek *et al*. in 1985 to synthesize [^11^C]putrescine under no-carrier-added conditions.^857^ Despite reaction optimization through DMSO as solvent and borane-THF as a more efficient reductant, low RCYs were observed (reaction time of 50–60 min, RCY of 7–13% based on [^11^C]NaCN, *A*_m_ of 2.3 GBq/μmol at EOS).^857^ It was postulated that the reaction proceeds *via* elimination of HBr from the alkyl bromide starting material to form acrylonitrile, which undergoes Michael addition with cyanide to form succinonitrile. Thus, under noncarrier added conditions, [^11^C]cyanide was consumed through reaction with HBr. This led McPherson *et al*. to explore the direct Michael addition of [^11^C]KCN with acrylonitrile (Figure 169B).^856^ This proved successful, with [^11^C]succinonitrile obtained in ∼70% RCY within 5 min.^856^ Subsequent azeotropic drying, reduction using borane-dimethylsulfide, and HPLC purification produced [*1*-^11^C]putrescine, suitable for use in clinical PET studies (reaction time, 50 min; RCY, 20% based on [^11^C]HCN; RCP > 97%, *A*_m_ >52 GBq/μmol at EOS).^858−860^ In 1991, Lambrecht *et al*. reported the use of solid-state support, which enabled the Michael addition to be performed under anhydrous conditions.^909^

#### 12.3.2 Preclinical Studies

The biodistribution of carrier-added carbon-11 labeled diamines of the formula NH_2_(CH_2_)_*n*_NH_2_ (*n* = 4–9) was studied in rats by Winstead *et al*.^815^ For all diamines, activity was found to accumulate in the prostate between 5 and 30 min.^815^ For all diamines, activity accumulated in the prostate between 5 and 30 min. The highest uptake was observed for [*1*-^11^C]putrescine in the prostrate while increasing the chain length by one carbon atom to [*1*-^11^C]cadaverine gave a significant reduction. Significant uptake at 5 min p.i. was observed in the lungs and kidneys, while brain uptake was negligible for [*1*-^11^C]putrescine and [*1*-^11^C]cadaverine. With [*1*-^11^C]cadaverine, the adrenal glands showed a progressive increase in activity until 60 min p.i. Most other tissues showed a progressive decrease over time for all the diamines.

The biodistribution of [*1*-^11^C]putrescine in mice was similar, with most uptake being observed in the liver, kidneys, and small intestine, while little activity was observed in the brain.^856^

Rat biodistribution studies using [*1*-^11^C]putrescine in varying levels of nonradioactive putrescine were performed by Jerabek *et al*.^857^ [*1*-^11^C]Putrescine binding was found to be saturable, with a progressive reduction of uptake being observed in the prostate at 1 h.^857^ In addition, a prostate to nontarget tissue (muscle) ratio of 10.5/1 was observed under baseline conditions, which the administration of unlabeled putrescine reduced.

#### 12.3.3 Clinical Studies

Human PET imaging studies with [*1*-^11^C]putrescine have been performed by researchers at Brookhaven to examine the utility of the tracer for metabolic imaging of brain tumours,^858,859^ and prostatic adenocarcinoma.^860^ In a study on primary and metastatic brain tumors, [*1*-^11^C]putrescine was taken up and retained in tumors, and higher contrast between tumor/normal brain was observed compared with [^11^C]*2*-deoxyglucose.^858^ This was ascribed to putrescine’s inability to cross the normal BBB and its low metabolism in the normal brain compared to glucose but did not ascertain whether or not [*1*-^11^C]putrescine uptake reflects the rate of tumor polyamine biosynthesis and prostatic adenocarcinoma.^860^

**Figure 170:**
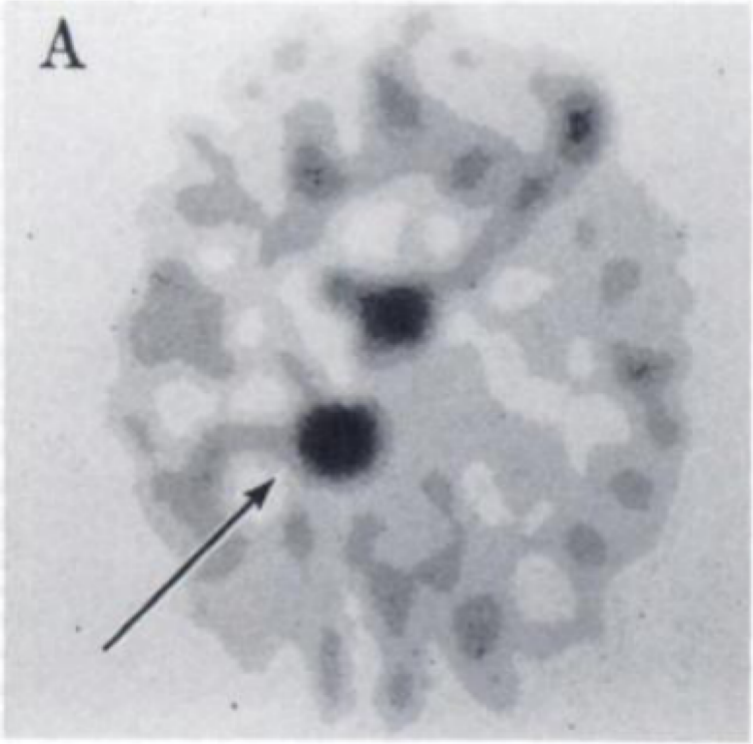
[^11^C]Putrescine PET scan of a patient with cerebellar metastases from lung carcinoma (45 min post-injection). Reproduced with permission from ref (858). Copyright 1987 Society of Nuclear Medicine. This work is licensed under a Creative Commons Attribution 4.0 International License (https://creativecommons.org/licenses/by/4.0/).

In a follow-up publication to address this question, [*1*-^11^C]putrescine was studied in 33 patients with various malignant brain tumors and benign or non-neoplastic lesions (Figure 170).^859^ These studies found that [*1*-^11^C]putrescine uptake was not specific for malignant tumors, with uptake relying on disruption of the BBB.

Similarly, in a study of human prostatic adenocarcinoma, Wang *et al*. observed a significant accumulation of activity in the prostate, bone, and rectum.^860^ However, despite rapid prostate uptake and retention and low uptake in the bladder, the tracer lacked specificity for prostatic adenocarcinoma, with significantly higher prostate uptake in normal controls *versus* cancer patients.

Analysis of the metabolic profile of [*1*-^11^C]putrescine in human plasma has only been reported for one subject, where it was found that by 20 min p.i., only 9% of intact tracer remained, with [^11^C]CO_2_ and nonvolatile metabolites accounting for the remainder of activity.^858^

### 12.4 Choline

#### 12.4.1 Radiosynthesis

**Figure 171:**
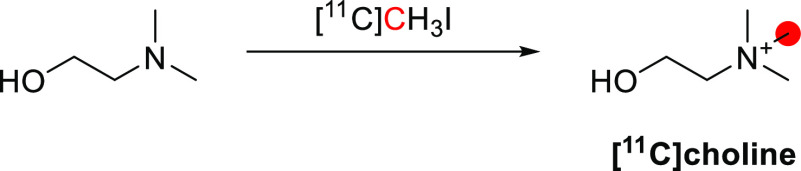
Synthesis of [^11^C]choline using [^11^C]CH_3_I.

In 1985, Rosen *et al*. synthesized [^11^C]choline by reacting [^11^C]CH_3_I with *2*-dimethylaminoethanol, producing [^11^C]choline, a synthesis time of 35 mins, RCY of ∼50%, and *A*_m_ of >11.1 GBq/μmol (Figure 171).^910^ A few years later, this method was improved by Hara *et al*., where [^11^C]choline was reacted with [^11^C]methyl iodide with dimethylaminoethanol at 120 °C for 5 min, producing 11 GBq of [^11^C]choline, a synthesis time of 25 mins and RCY > 98% and *A*_m_ > 133 GBq/μmol.^862^ The same group developed an automated synthesis of [^11^C]choline by reacting [^11^C]CH_3_I with *2*-dimethylaminoethanol at 130 °C for 5 mins, producing 11 GBq of [^11^C]choline, a synthesis time of 20 mins, RCY of 86%, and *A*_m_ of 150 GBq/μmol).^911^ An alternative synthetic route to [^11^C]choline production is the reaction of [^11^C]methyl triflate with *2*-dimethylaminoethanol, obtaining 60–85% RCY (based on [^11^C]CO_2_ with a reaction time of 15–20 min) and *A*_m_ >29.6 GBq/μmol.^871^

[^11^C]Choline was approved in 2012 by the U.S. Food and Drug Administration and the European Medicines Agency in 2018 for clinical use. There was no *A*_m_ release limit for [^11^C]choline. Several synthetic approaches have been described in the literature,^656^ and different automated production methods and modules for clinical production.^911−918^ Excellent yields (RCY > 80% at EOB) are always obtained in a short synthesis time, allowing reliable production of multiple doses and several batches per day.^919^ In addition, Trasis offers commercially available cassette kits and ready-to-use consumables for fully automated preparation of [^11^C]choline on the Trasis *AllinOne* synthesizer, further facilitating GMP implementation. The most simple, efficient, and reliable automated method for [^11^C]choline routine clinical production was described in detail, and the quality control procedures by Hockley, Shao, *et al*.^848,851^ Typical RCYs >60% at EOB in a total synthesis time of 20 min.

#### 12.4.2 Preclinical Studies

**Figure 172:**
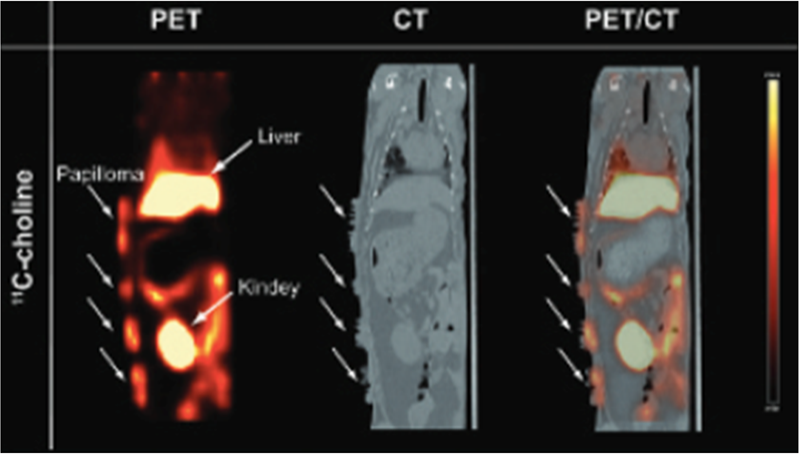
[^11^C]Choline whole-body PET (left), CT (middle), and fused image (right) of a NZW rabbit at 10 months post-infection with papillomavirus (scans made 50 min p.i.). Reproduced with permission from ref (863). Copyright 2014 Decker. This work is licensed under a Creative Commons Attribution 3.0 License (http://www.creativecommons.org/licenses/by/3.0/).

[^11^C]Choline has shown potential for assessing the degree of inflammation in atherosclerotic plaques^920^ to detect cancer (breast,^921^ colorectal,^922^ papillomavirus,^863^ prostate,^922^ and brain cancer^862^) and pulmonary arterial hypertension.^923^ A biodistribution study of [^11^C]choline in normal rats showed high uptake in the kidneys, lungs, liver, and adrenal glands with a gradual decline in the uptake of these organs after 10 min p.i.^861^ A metabolite study was carried out in the plasma and urine of rats, showing that [^11^C]choline represented <50% in plasma after 5 min p.i. Similar results were found in urine, where unmetabolized [^11^C]choline represented about 70% after 10 min p.i. The major metabolite was identified as [^11^C]betaine.^817^ A biodistribution study of [^11^C]choline in normal rabbits showed the highest uptake in the liver, followed by the kidneys and spleen. Other organs such as the brain, lungs, and heart had no visible uptake.^862^ Similar pattern uptake was also observed in rabbits to detect papillomavirus-induced tumors (Figure 172).^863^

The only reported monkey study with [^11^C]choline was performed by Friedland *et al*. in 1983, in which brain imaging and organ biodistribution were studied.^864^ [^11^C]Choline had a rapid brain uptake followed by a rapid decline compared with other organs such as the heart, lung, liver, and kidney. This can be explained by a washout from the intravascular and interstitial fluid spaces as well as the re-entering of [^11^C]choline into circulation after clearance from the blood during the first pass, and distribution to other organs.^864^

#### 12.4.3 Clinical Studies

**Figure 173:**
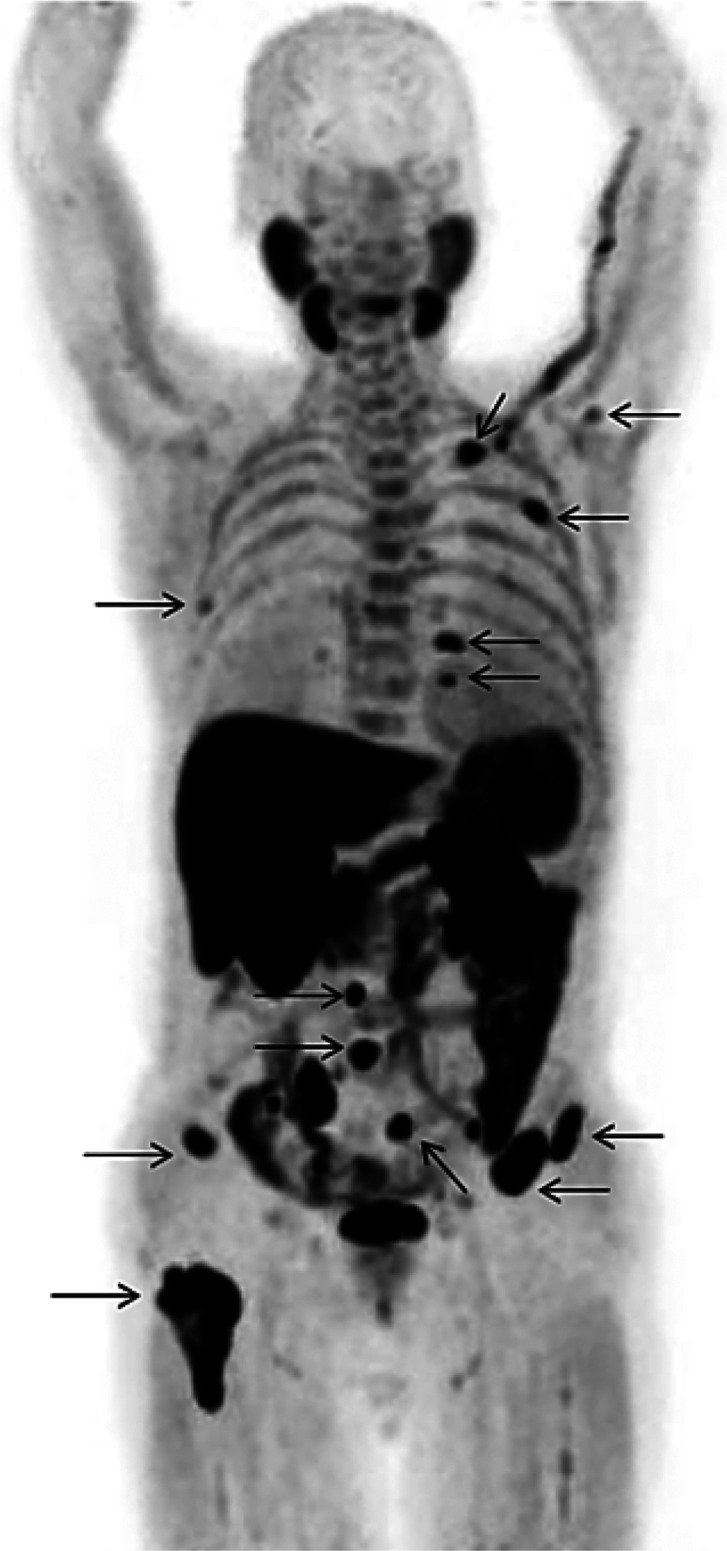
[^11^C]Choline MIP PET image showing several spots of uptake. Reproduced with permission from ref (868). Copyright 2018 Cureus. This work is licensed under a Creative Commons Attribution 3.0 License (http://www.creativecommons.org/licenses/by/3.0/).

Several reported PET studies with [^11^C]choline have been performed over the last few years. Uptake of [^11^C]choline was investigated in a normal 60-year-old man, showing the highest uptake in the kidney, liver, pancreas, small intestine, and spleen.^865^ Similar to the rabbit studies, the uptake in the brain of healthy patients was deficient compared to extracerebral tissue. The pituitary body represented the brain area with the highest uptake of [^11^C]choline.^862^ Choline is highly concentrated in prostate cancer cells;^866^ therefore, [^11^C]choline has been used to evaluate prostate cancer, showing promising results in defining local tumor stage and nodal involvement.^867^ An example is represented in Figure 173, where it is possible to observe several bone metastases using [^11^C]choline. The sensitivity of [^11^C]choline to detect bone metastases was 96.9% compared to 90.3% detected by conventional imaging.^868^

**Figure 174:**
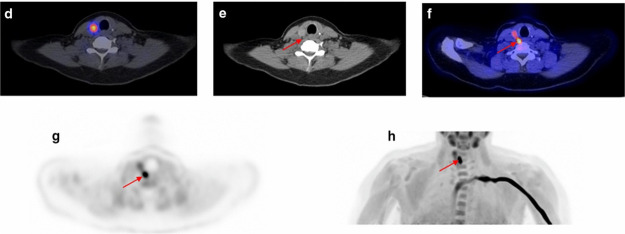
[^11^C]Choline axial PET (d), CT (e), PET/CT (f), axial PET (g), and coronal PET (h) images of a suspected parathyroid adenoma (red arrow). Reproduced with permission from ref (869). Copyright 2021 Springer-Verlag. This work is licensed under a Creative Commons Attribution 4.0 International License (https://creativecommons.org/licenses/by/4.0/).

In recent years, PET/CT imaging with [^11^C]choline has gained interest in detecting and localizing parathyroid adenomas. Noltes *et al*. evaluated the diagnostic performance of [^11^C]choline in patients with primary hyperparathyroidism after negative or discordant first-line imaging (Figure 174).^869^ The sensitivity of [^11^C]choline to localize lesions was 97%, where 37 of the 40 suspected lesions were histologically confirmed as parathyroid adenoma or parathyroid hyperplasia.^869^ Moreover, Parvinian *et al*. evaluated the efficacy of [^11^C]choline in detecting parathyroid adenomas in patients with abnormally high serum calcium and parathyroid hormone levels. All suspected adenomas were [^11^C]choline avid, and a low frequency of incidental thyroid lesions as possible parathyroid adenomas was observed.^870^ The promising results of these studies might lead to more minimally invasive parathyroid procedures.

Metabolite studies were carried out in patients with brain tumors, prostate cancer, and prostate hyperplasia. [^11^C]Choline rapidly declined in arterial plasma within 25 min. This analysis also revealed that the most abundant metabolism was [^11^C]betaine. Another metabolite was also identified but not analyzed.^817^

### 12.5 Daunorubicin

#### 12.5.1 Radiosynthesis

**Figure 175:**
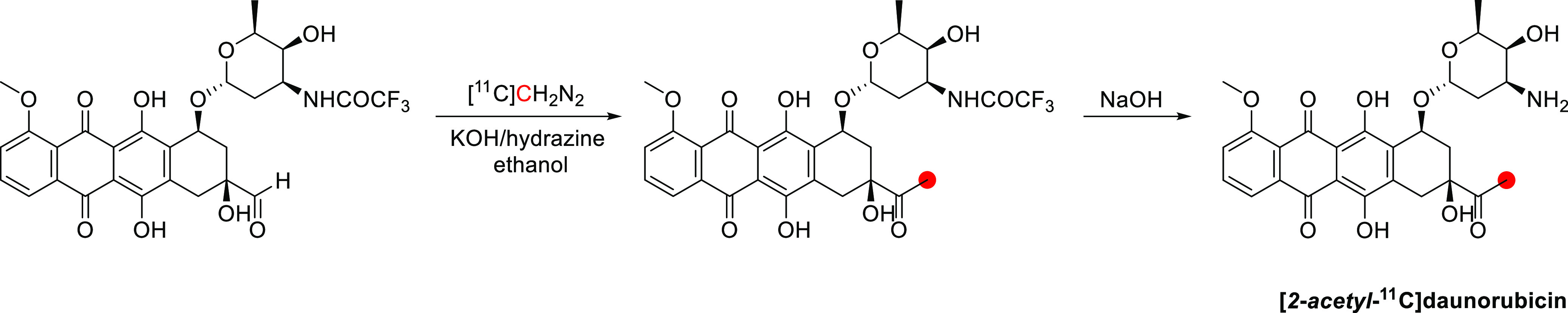
Synthesis of [*2*-*acetyl*-^11^C]daunorubicin using [^11^C]CH_2_N_2_. ^11^C radionuclide position is highlighted in red.

[^11^C]Daunorubicin has been labeled in two positions, 2-*acetyl*-^820^ and 4-*methoxy*-.^821^ [*2*-*acetyl*-^11^C]Daunorubicin was prepared by reaction of [^11^C]CH_2_N_2_ with 9-formyl-trifluoroacetyl-daunorubicin in KOH/hydrazine/ethanol solution for 5 min at 60 °C followed by NaOH hydrolysis (Figure 175). The overall RCY was 3 ± 1%, with a RCP of 99% and *A*_m_ of 0.740–1.111 GBq/μmol with a total synthesis time of 53 min.

**Figure 176:**
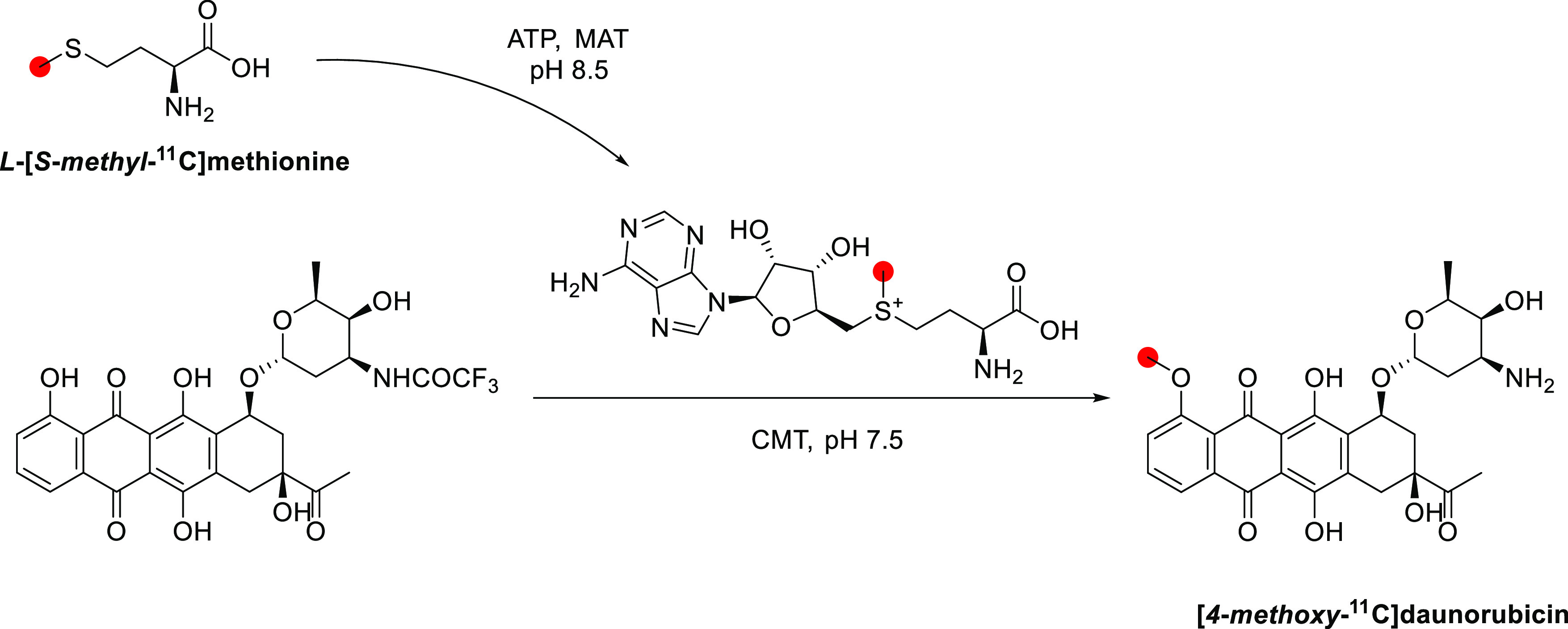
Synthesis of [*4*-*methoxy*-^11^C]daunorubicin using *S*-adenosyl-*l*-[*methyl*-^11^C]methionine. ^11^C radionuclide position is highlighted in red.

[*4*-*Methoxy*-^11^C]daunorubicin has been synthesized using an enzymatic route by the methylation of carminomycin, catalyzed by carminomycin-*4*-*O*-methyltransferase (CMT), to [*4*-*methoxy*-^11^C]daunorubicin by *S*-adenosyl-l-[*methyl*-^11^C]methionine. The latter was initially synthesized from *l*-[*methyl*-^11^C]methionine catalyzed by *l*-methionine-*S*-adenosine transferase (MAT). The final product was isolated within 75 min and with a RCY of 1% (EOB) (Figure 176).^821^

#### 12.5.2 Preclinical Studies

[2-*Acetyl*-^11^C]daunorubicin has been evaluated *in vivo* in healthy^820^ and tumor-bearing rats.^872^ In healthy male Wistar rats, biodistribution studies were performed 60 min after iv injection of the tracer (tracer or pharmacological dosage: 10 mg/kg body weight for [2-*acetyl*-^11^C]daunorubicin) to determine the pharmacokinetics. Differences in uptake are observed in the radioactivity level, between the noncarrier-added and a pharmacological dose of 10 mg/kg body weight, in plasma, liver and urine.^820^

*In vivo* studies in male nude rats bearing tumors bilaterally, a P-gp-negative small cell lung carcinoma (GLC4) and its P-gp-overexpressing subline (GLC4/P-gp) showed a 159% higher level of [2-*acetyl*-^11^C]daunorubicin in GLC4 than in GLC4/P-gp tumors. According to the authors, [*2*-*acetyl*-^11^C]daunorubicin levels showed rapid plasma clearance after the injection, and activity levels showed no significant increase in any other tissue than the GLC4/P-gp tumor; however, no specific data are available.^872^

### 12.6 Erythromycin Lactobionate

#### 12.6.1 Radiosynthesis

**Figure 177:**
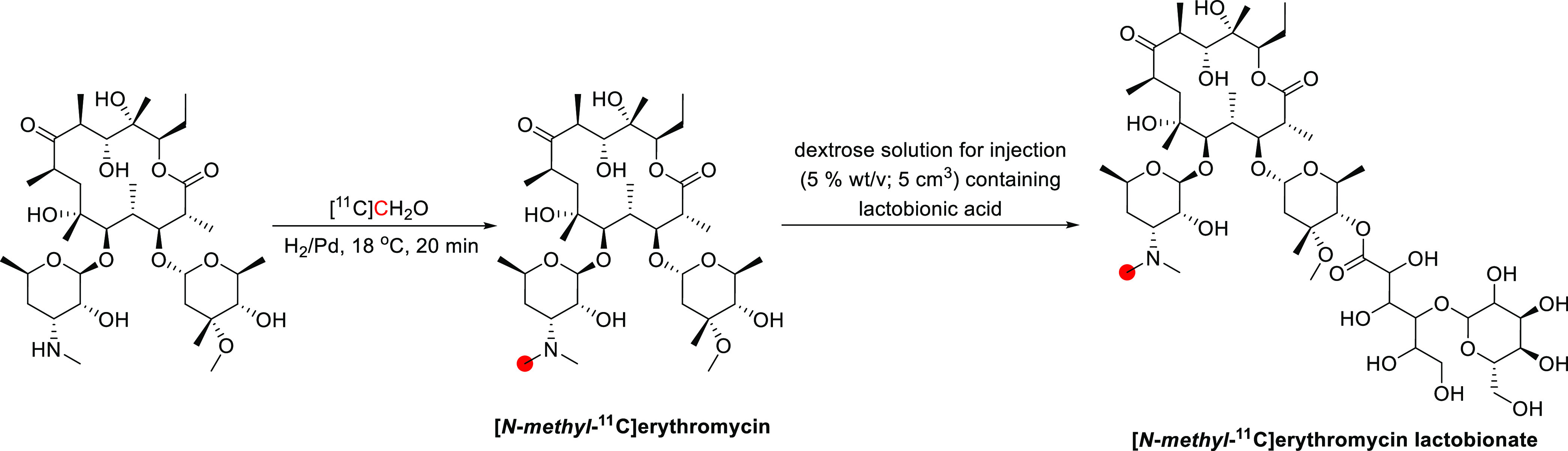
Synthesis of [^11^C]erythromycin lactobionate using [^11^C]CH_2_O. ^11^C radionuclide position is highlighted in red.

[*N*-*Methyl*-^11^C]erythromycin lactobionate has been prepared by reductive alkylation of an *N*-desmethyl-erythromycin using [^11^C]CH_2_O in the presence of H_2_ and Pd on charcoal at 18 °C for 20 min (Figure 177).^822,823^ The purified fraction from the silica column, dissolved in dextrose solution for injection (5% wt/v; 5 cm^3^) containing lactobionic acid and the mixture added to unlabeled erythromycin A (12.8 mg), shaken thoroughly and passed through a sterile filter (0.22 μm). The final product required 42 min from the end of [^11^C]CO_2_ production, with a RCY of 4–12% (based on the [^11^C]CO_2_ activity used at the end of proton irradiation).^822,823^

#### 12.6.2 Clinical Studies

**Figure 178:**
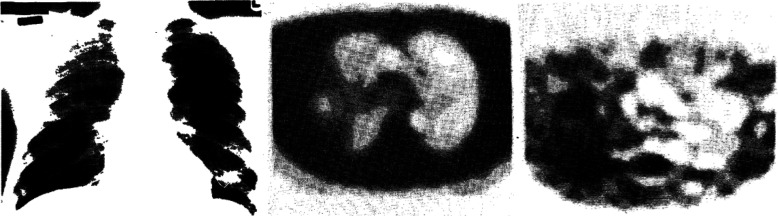
[*N*-*Methyl*-^11^C]erythromycin A lactobionate chest radiograph (left) and tomograms showing regional distribution of extravascular lung density (middle) and extravascular (right) in a patient with upper-lobe pneumonia. Reproduced with permission from ref (873). Copyright 1982 Elsevier.

[*N*-*Methyl*-^11^C]erythromycin lactobionate has been used to determine the pulmonary concentration of the drug in five patients with acute lobar pneumonia in the first few days of their illness. Only one lobe of the lung was infected during lobar pneumonia; thus, the uninfected lung was used as a control. [*N*-*Methyl*-^11^C]erythromycin A lactobionate was administered intravenously in a typical clinical dose of 270 mg. Blood samples and tomographic data were collected every 10 min for 60 min (Figure 178). Lung images were corrected for tracer present in the blood content of this tissue using [^11^C]CO to measure the pulmonary blood volume throughout the experiment with [*N*-*methyl*-^11^C]erythromycin A lactobionate.^873^ [*N*-*Methyl*-^11^C]erythromycin A lactobionate uptake at the pulmonary reached a peak at 10 min after injection and remained at a pharmacologically effective dose throughout the study. Although the infected lung showed an increase in uptake compared to the uninvolved lung, the corrected data proved no statistical difference in the extravascular concentration.^873^

### 12.7 Glycerol

#### 12.7.1 Radiosynthesis

The synthesis of [^11^C]glycerol was achieved by biosynthesis using the alga*Girgartina stellata*.^798^ After being exposed to light, the chamber containing the alga was recirculated with a flow of [^11^C]CO_2_ in N_2_ for 20 min, allowing the synthesis of [^11^C]glycerol (35%) and [^11^C]galactose (65%). The mixture of products was extracted with alcohol and separated by liquid chromatography, returning 2.5 MBq of [^11^C]glycerol with a total processing time of 80–90 min and *A*_m_ of 1.08 × 10^–4^ GBq/μmol.^798^

### 12.8 Hippuric Acid

#### 12.8.1 Radiosynthesis

**Figure 179:**
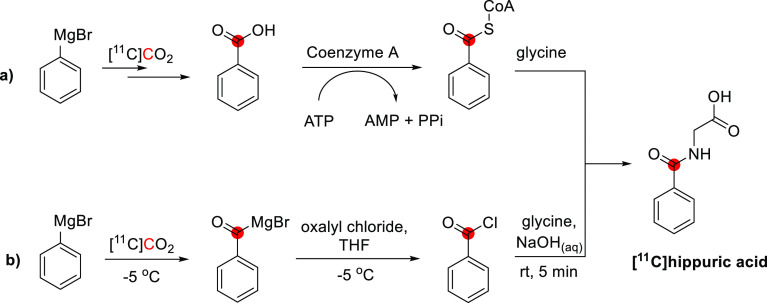
Synthesis of [^11^C]hippuric acid: (a) biosynthetically using rat liver mitochondrial; (b) reaction of glycine with [*carbonyl*-^11^C]benzoyl chloride. ^11^C radionuclide position is highlighted in red.

[^11^C]Hippuric acid was first prepared from carboxylation of phenylmagnesium bromide with [^11^C]CO_2_ to make [^11^C]benzoic acid,and then to attach glycine to this biosynthetically using rat liver mitochondria with a RCY of 1.5–10% and a total synthetic time of 70 min (Figure 179).^855^ [^11^C]Hippuric acid was also prepared by adding an aqueous alkaline solution of glycine to neat [*carbonyl*-^11^C]benzoyl chloride with a RCY of 8 ± 2%, based on [^11^C]CO_2_ (Figure 179).^874^

#### 12.8.2 Preclinical Studies

[^11^C]Hippuric acid has been studied in mice and rabbits after iv injection.^855,874^ In healthy, Mrp_4_^+^ and Oat_3_^+^ mice using PET, Kikuchi *et al*. evaluated the uptake of [^11^C]hippuric acid in the brain and heart after injection of the ester forms using the so-called metabolite extrusion method.^874^ The esters were rapidly hydrolyzed, leading to the corresponding [^11^C]hippuric acid, with no other detected metabolites. The low level of radioactivity observed in the brain at 0.5 min post-injection considerably decreased at 15 min p.i., even in the organic anion transporter knockout mice. Comparable low uptake of [^11^C]hippuric acid into the heart was observed.^874^ In rabbits, [^11^C]hippuric acid was rapidly excreted through the kidneys, and the renograms were comparable to those made with “hippuran”.^855^

### 12.9 Lactic Acid

#### 12.9.1 Radiosynthesis

**Figure 180:**
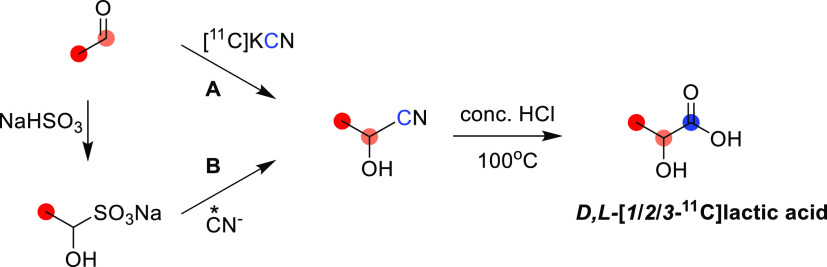
Chemical preparations of [^11^C]lactic acid. ^11^C radionuclide positions are highlighted in red, orange, and blue.

The synthesis of racemic, carrier-added [^11^C]lactic acid was first reported by Cramer and Kistiakowsky in 1941 *via* reaction of KCN with acetaldehyde and subsequent hydrolysis of the intermediate [^11^C]lactonitrile (Figure 180A).^877^ Using [^11^C]KCN gave [^11^C]lactic acid labeled in the 1-position (RCY of 30–40%, synthesis time of 90–120 min), while an isotopomeric mixture of [^11^C]acetaldehyde yielded [^11^C]lactic acid labeled at the *2*-/*3*-position (RCY of 40–50%, synthesis time of 150 min). In 1978 Winstead *et al*. used an *in situ* generated acetaldehyde–bisulfite adduct in reaction with [^11^C]NaCN to generate [*1*-^11^C]lactonitrile, which following hydrolysis, gave [*1*-^11^C]lactic acid (Figure 180B; yield of 280 MBq, RCY of 53.5%, synthesis time of 119 min).^875^ Based on this approach, Drandarov *et al*. developed an automated, no-carrier-added synthesis of enantiopure *d*- and *l*-[*1*-^11^C]lactic acid using chiral ligand exchange chromatography (yield of 2.5 GBq at EOS, RCY >80%, synthesis time of 45 min, RCP >99%, ee >99%).^878^

**Figure 181:**
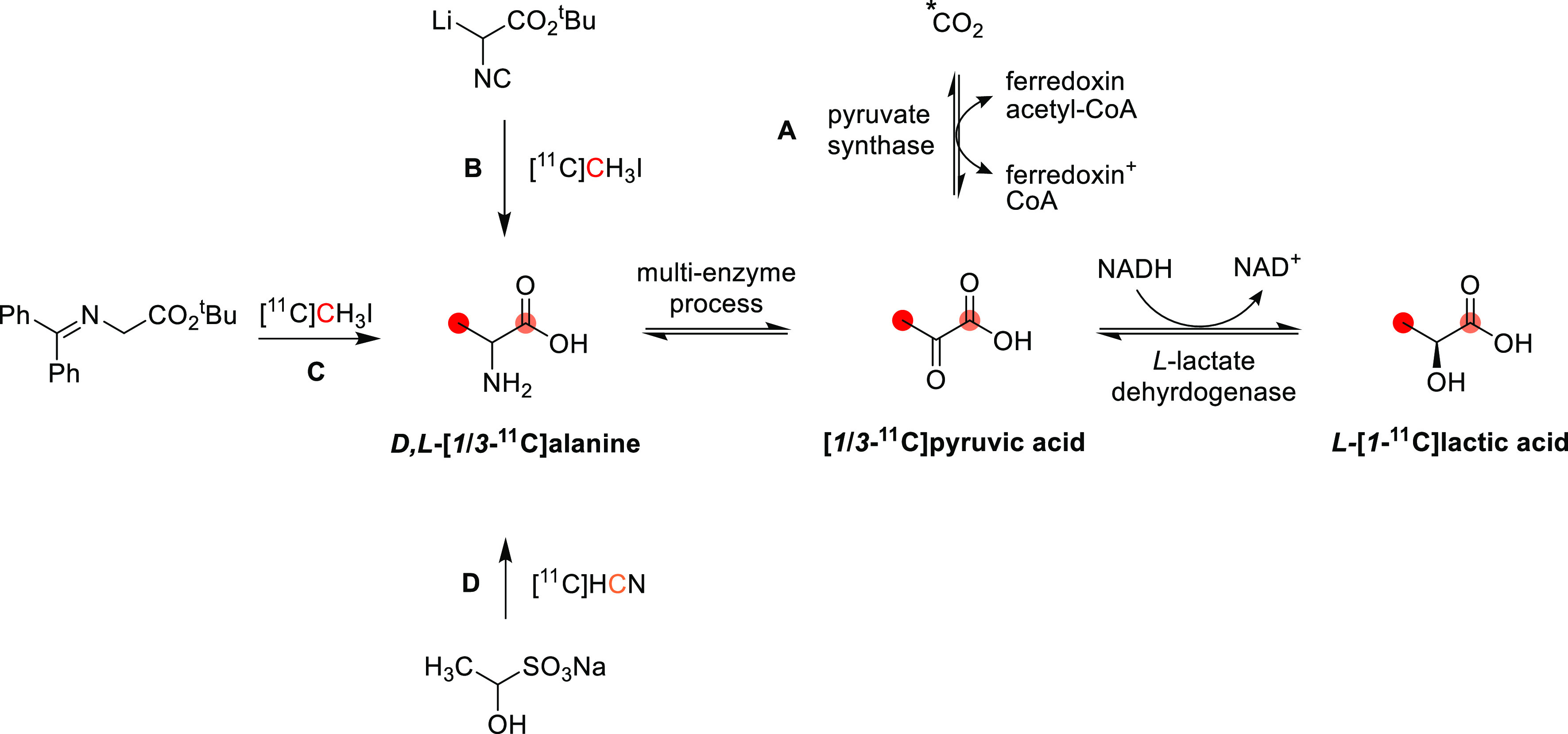
Enzymatic preparations of *L*-[*1*-^11^C]lactic acid using [^11^C]CH_3_I or [^11^C]HCN. ^11^C radionuclide positions are highlighted in red and orange.

Several enzymatic approaches have been used to prepare enantiopure *l*-[*1*-^11^C]lactic acid,^241,924,925^ and *l*-[*4*-^11^C]lactic acid (Figure 181).^879,881,925^ Each preparation proceeds *via* the synthesis of intermediate [^11^C]pyruvic acid and its reduction to *l*-[^11^C]lactic acid using l-lactate dehydrogenase, while the method of forming pyruvic acid varies. In 1980, Cohen *et al.*,^924^ used [^11^C]CO_2_ and acetyl coenzyme A in the presence of pyruvate synthase to access *l*-[*1*-^11^C]pyruvic acid, which was then converted to *l*-[*1*-^11^C]lactic acid (Figure 181A; RCY of 3–5%, synthesis time of 20 min). Alternatively, [^11^C]pyruvic acid can be formed from *d*,*l*-[^11^C]alanine using multicomponent enzyme reactions. *d*,*l*-[^11^C]alanine has been radiolabeled in the *3*-position by ^11^C-methylation of glycine derivatives to ultimately form *l*-[*4*-^11^C]lactic acid (Figure 181B; yield of 220 MBq, RCY of 26.5%, synthesis time of 40 min, *A*_m_ of 0.02 GBq/μmol;^881^Figure 181C; yield of 400 MBq, RCY of 60%, synthesis time of 40 min, RCP >99%;^7^ yield of 1.4 GBq, RCY of 13.4%, activity yield of 2.5%, synthesis time of 49 min, RCP of 97%, ee >99%^879^). Meanwhile, *d*,*l*-[^11^C]alanine can be radiolabeled in the *1*-position *via* a reaction of [^11^C]cyanide with the acetaldehyde-bisulfite adduct to ultimately form *l**-*[*1*-^11^C]lactic acid (Figure 181D; yield of 925 MBq, RCY of 25% (from HCN), synthesis time of 40 min from HCN, RCP >97%, *A*_m_ of 2–3 GBq/μmol;^241^ yield of 320 MBq, RCY of 80% (from HCN), synthesis time of 40 min (from CO_2_), RCP >99%^925^).

#### 12.9.2 Preclinical Studies

**Figure 182:**
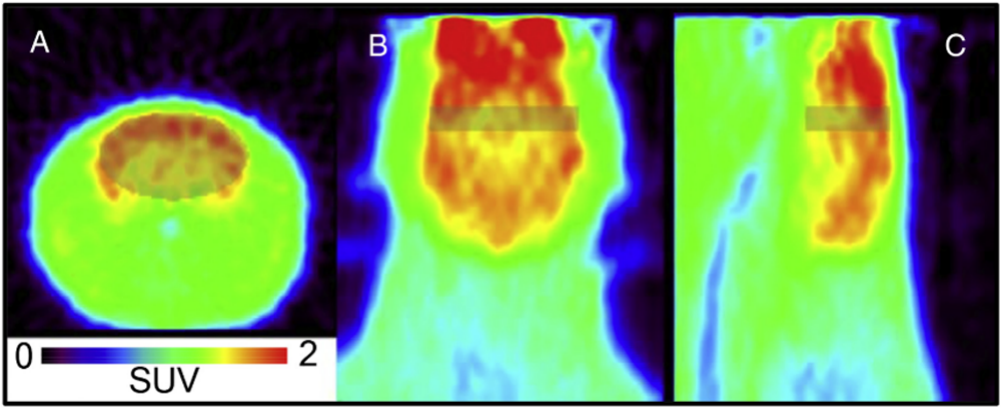
*l*-[*3*-^11^C]Lactate PET images of a rat head in (A) transverse, (B) coronal, and (C) sagittal planes. Reproduced with permission from ref (879). Copyright 2018 Elsevier.

In an early scintigraphic study in 1978, Winstead *et al*. studied the distribution of [*1*-^11^C]lactic acid in a dog, observing the accumulation of activity principally in the pancreas, liver, lungs, heart-blood pool, and kidneys with significant excretion in urine and bile.^875^ In 1979 Cohen *et al*. reported a biodistribution study in rabbits using *l*-[*1*-^11^C]lactic acid and found a seven-fold increase in activity myocardium compared to the lung.^876^ The next *in vivo* study, and first lactate PET study, was performed nearly 30 years later by Herrero *et al*. using *l*-[*4*-^11^C]lactic acid as a tracer for myocardial lactate metabolism in dogs.^880^ Arterial blood metabolism studies found the most abundant ^11^C fraction to be lactate + pyruvate (43%), followed by neutral metabolites (glucose, 31%) and CO_2_ (15%), with basic metabolites (primary alanine) contributing 10% to total arterial ^11^C activity. In 2018, Temma *et al.*, used *l*-[*4*-^11^C]lactic acid for the pharmacokinetic analysis of lactate metabolism in rat brains, and their finding suggests active lactate brain usage *in vivo* (Figure 182).^879^ Rapid metabolic conversion to [^11^C]glucose was observed in arterial blood (79% [^11^C]glucose; 12% *l*-[*4*-^11^C]lactate at 10 min p.i.).

### 12.10 *N*-Acetyl-leukotriene _4_

#### 12.10.1 Radiosynthesis

**Figure 183:**
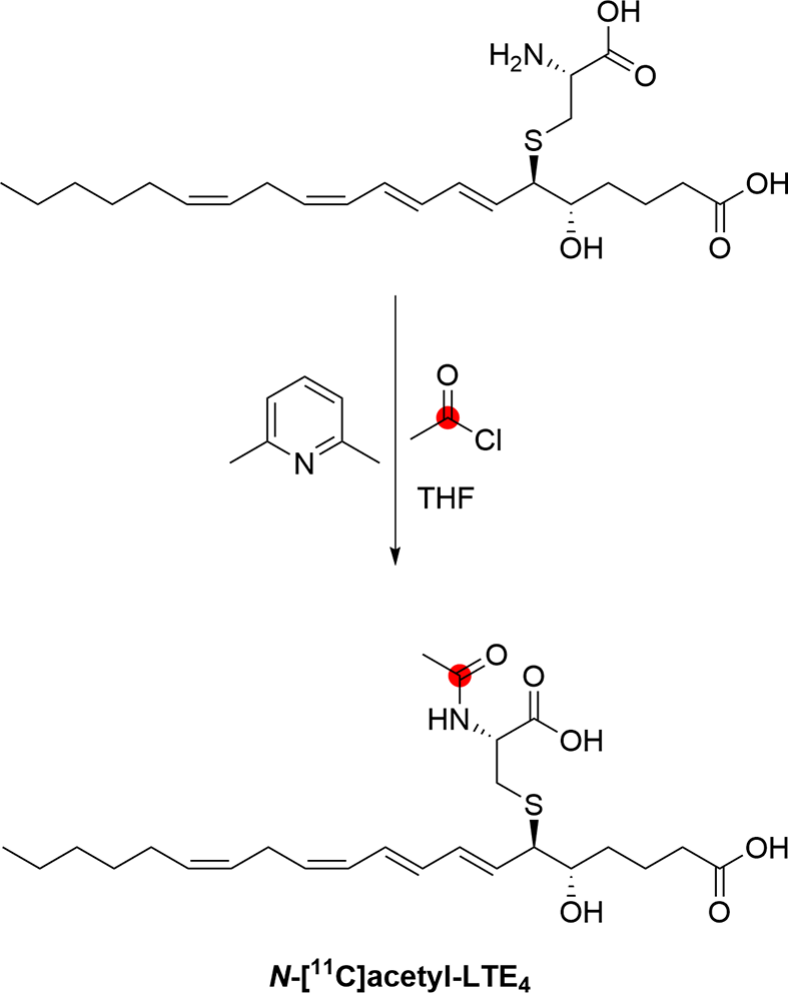
Synthesis of *N*-[^11^C]acetyl-LTE_4_ using [*1*-^11^C]CH_3_COCl. ^11^C radionuclide position is highlighted in red.

^11^C-Labeled *N*-acetyl-LTE_4_ was first labeled from the *N*-acetyl group using [*1*-^11^C]CH_3_COCl (Figure 183).^882^ RCP was >95%, and *A*_m_ was about 2 GBq/μmol. However, there was no mention of total synthesis time or other reaction conditions. In the following years, *N*-[^11^C]acetyl-LTE_4_ was produced from LTE_4_ and [*1*-^11^C]acetyl chloride in THF in 50 min (yield 1.3%, CP and RCP was 95%, the average *A*_m_ was 2 GBq/μmol).^883,884^

#### 12.10.2.1 Preclinical Studies

**Figure 184:**
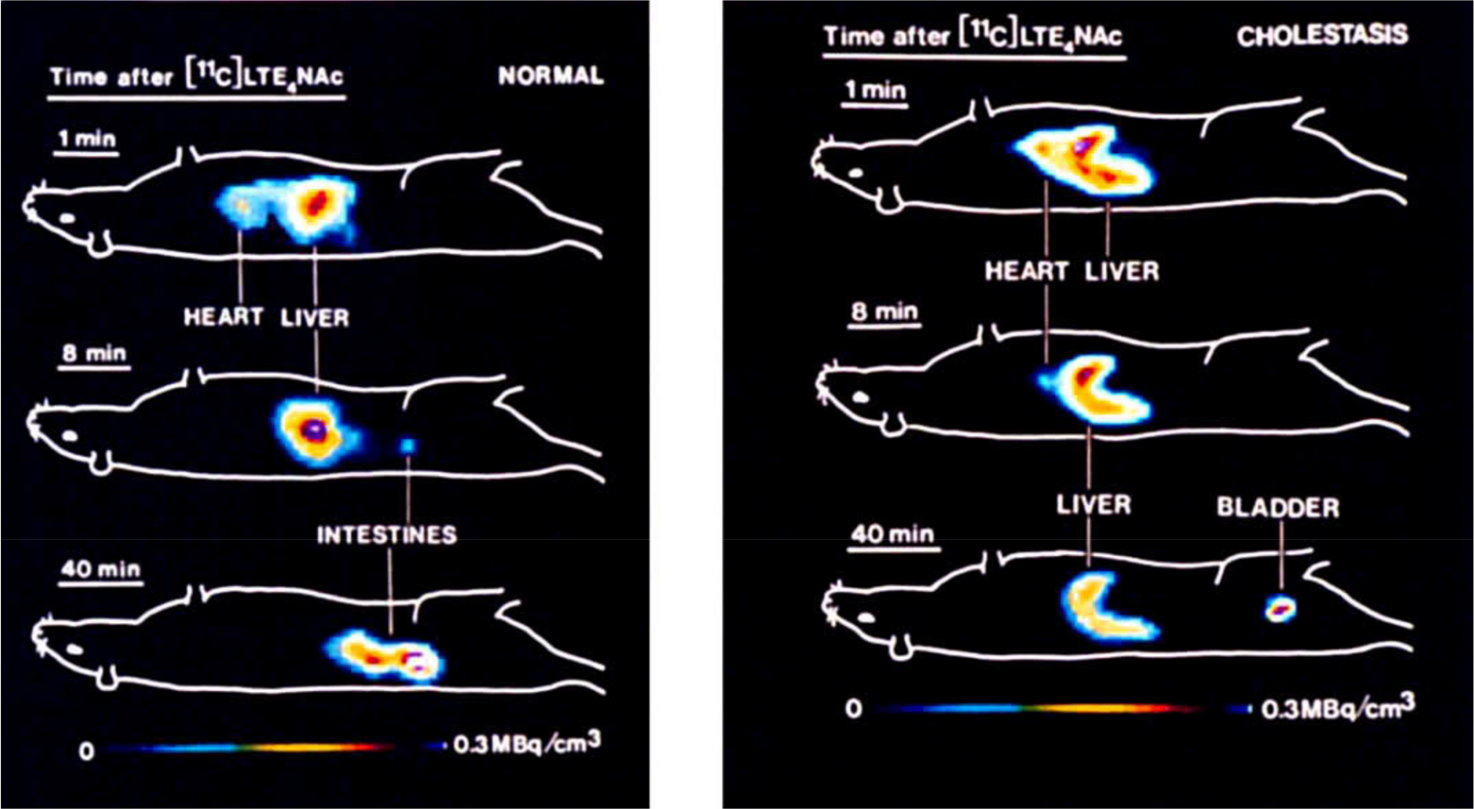
*N*-[^11^C]Acetyl-LTE_4_ pharmacokinetics in a normal rat (left) and a rat with bile duct obstruction-induced cholestasis (right). Reproduced with permission from ref (883). Copyright 2005 John Wiley and Sons.

The first preclinical study was done in 1991 to investigate leukotriene elimination in rats using *N*-[^11^C]acetyl-LTE_4_ from the jugular vein.^882^*N*-[^11^C]Acetyl-LTE_4_ cleared rapidly from the blood, reached its maximum in the liver 4 min after the iv injection and then metabolized through the intestines. In the following year, the same study was repeated to observe *in vivo* elimination of cysteinyl leukotrienes in rats.^832^ Results were consistent with the previous study (iv injection, the dose was 27 MBq/kg). Keppler and his co-workers continued expanding their studies and used *N*-[^11^C]acetyl-LTE_4_ in female Wistar rats, male Hartley guinea pigs (no data was shown), female homozygous transport mutant Wistar rats, and female Cynomolgus monkey.^883^*N*-[^11^C]Acetyl-LTE_4_ was given intravenously and rapid elimination was observed from the blood to the liver, and then excretion into the bile was observed.^883^ As in previous studies, in monkeys, renal excretion into urine was observed, and transit time through the liver was 34 min with 16 min hepatic excretion half-time. These times for normal rats were 17 and 8.5 min, respectively. In mutant and healthy rats with cholestasis due to bile duct obstruction (induced with surgery before injection of *N*-[^11^C]acetyl-LTE_4_), these periods were much more extended than monkeys’ and showed longer organ storage and metabolism. Accumulation of the *N*-[^11^C]acetyl-LTE_4_ was seen in the bladder of mutant rats and rats with cholestasis due to bile duct obstruction instead of the intestines (Figure 184).^883^

### 12.11 *N*-Methyltaurine

#### 12.11.1 Radiosynthesis

**Figure 185:**
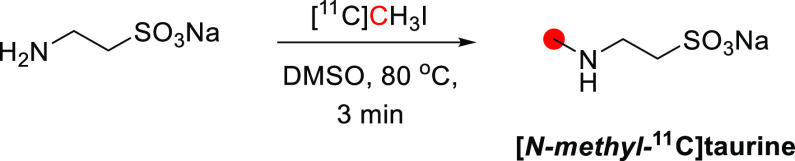
Synthesis of [*N*-*methyl*-^11^C]taurine using [^11^C]CH_3_I. ^11^C radionuclide position is highlighted in red.

Labeling of *N*-methyltaurine has only been performed from the methylamino group to synthesis of [*N*-*methyl*-^11^C]taurine by using [^11^C]CH_3_I (Figure 185).^885,926,927^ The first synthesis was done from the sodium salt of taurine and [^11^C]CH_3_I at 80 °C in 3 min. This synthesis was done to see the biodistribution of conjugated bile acids (cholic acid, chenodeoxycholic acid, deoxycholic acid, ursodeoxycholic acid, lithocholic acid) in pigs, so there is no data available for the activity of [*N*-*methyl*-^11^C]taurine.^885^

### 12.12 Oxalic Acid

#### 12.12.1 Radiosynthesis

**Figure 186:**
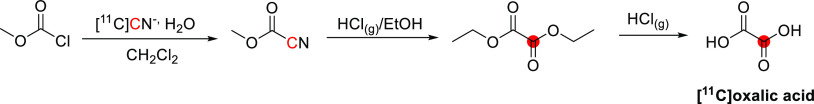
Synthesis of [^11^C]oxalic acid using [^11^C]CN^–^. ^11^C radionuclide position is highlighted in red.

[^11^C]Oxalic acid was first synthesized in 1942 by Nahisky *et al*. from the oxidation of [*1*-^11^C]propionic acid and *α*- and *β*-hydroxypropionic acid.^928^ A few years later, Thorell *et al*. prepared [^11^C]oxalic acid in a three-step procedure. In this process, [^11^C]CN^–^ was reacted with methyl chloroformate to generate the intermediate [^11^C]cyanoformate, and after evaporation of HCl, the [^11^C]oxalic acid was obtained (Figure 186). This reaction produced [^11^C]oxalic acid in a RCY of 70.8%.^886^

### 12.13 Paclitaxel

#### 12.13.1 Radiosynthesis

**Figure 187:**
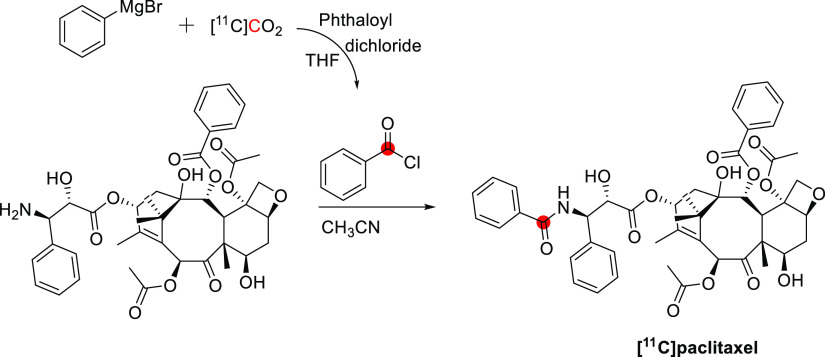
Radiosynthesis of [^11^C]paclitaxel using [*carboxyl*-^11^C]benzoyl chloride. ^11^C radionuclide position is highlighted in red.

[^11^C]Paclitaxel was synthesized by reacting the primary amine precursor of paclitaxel with [*carboxyl*-^11^C]benzoyl chloride (Figure 187). The synthesis, purification, and formulation time was 38 min from EOB with an average *A*_m_ of 0.0499 GBq/μmol at EOS. The average RCY was 7%, with a RCP > 99%.^887^

### 12.14 Phenylethanolamine

#### 12.14.1 Radiosynthesis

**Figure 188:**
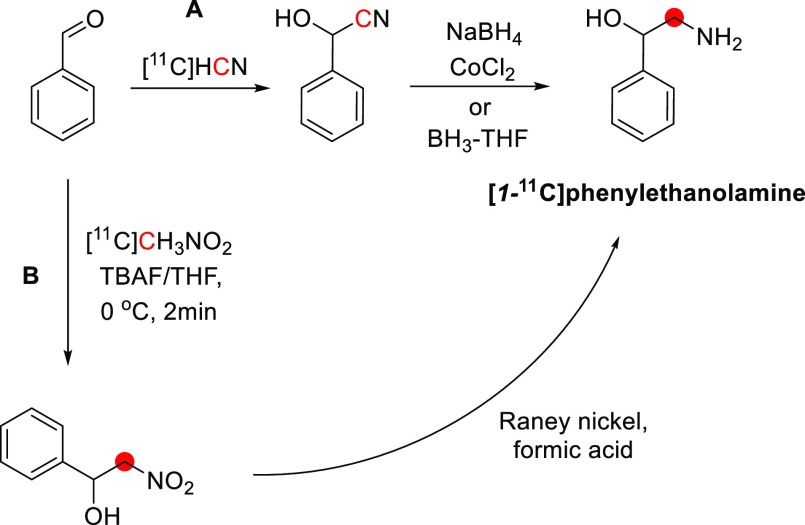
Synthesis of [*1*-^11^C]phenylethanolamine using [^11^C]HCN or [^11^C]CH_3_NO_2_. ^11^C radionuclide position is highlighted in red.

No-carrier-added [*1*-^11^C]phenylethanolamine was prepared in two steps *via* a combined enzymatic and radiochemical synthesis. Free of ammonia hydrogen [^11^C]cyanide was prepared following an established protocol and collected by bubbling through a solution of 0.05 M 50% methanolic acetate buffer (pH 5.4) held at −20 °C.^929^ A mixture of benzaldehyde and salt-free mandelonitrile lyase in CH_2_Cl_2_ was added to this trapping solution and stirred for 10 min at room temperature (20–25°C). The solvent was evaporated, and the [^11^C]mandelonitrile intermediate was used for the next step without further purification. The [^11^C]cyanohydrin intermediate was reduced by applying two different approaches (Figure 188). In the first one, the reduction was carried out using sodium borohydride–cobaltous chloride in methanol at room temperature for 10 min. The final product was purified by a small cation-exchange column (H^+^ form) and then analyzed by analytical HPLC, indicating the presence of two radioactive peaks, one corresponding to [*1*-^11^C]phenylethanolamine and the other corresponding to a [*1*-^11^C]phenethylamine byproduct. When the purification was performed with a preparative HPLC using a reversed-phase column and eluting with a 4:1 mixture of 0.01 N HCl and methanol, no chemical and radiochemical impurities were detected. [*1*-^11^C]Phenylethanolamine hydrochloride was isolated with a RCP of >98%. In the second approach, the [^11^C]cyanohydrin intermediate was reduced to the required amino alcohol functionality with the boran–THF complex at 50 °C for 8 min, and only the [*1*-^11^C]phenylethanolamine hydrochloride was isolated with a RCP of better than 98% (Figure 188A). The two synthetic approaches to [*1*-^11^C]phenylethanolamine gave almost the same overall RCYs of 2–4% at EOS in a total synthesis time of 50–60 min. The *A*_m_ of the product obtained was 130.1 GBq/μmol as determined by UV spectroscopy.^888^

Another method was developed by Nagren *et al*. in 1993. [*1*-^11^C]Phenylethanolamine was prepared by reaction of [^11^C]CH_3_NO_2_ with a mixture of benzaldehyde and TBAF in THF at 0 °C for 2 min, followed by reduction with Raney nickel in formic acid (Figure 188). After semipreparative HPLC purification, [*1*-^11^C]phenylethanolamine was isolated with a RCY of 37–50% (based on [^11^C]CH_3_NO_2_), RCP >98%, and *A*_m_ of 26–56 GBq/μmol within 40–45 min.^685^

### 12.15 Phenylpyruvic Acid

#### 12.15.1 Radiosynthesis

**Figure 189:**
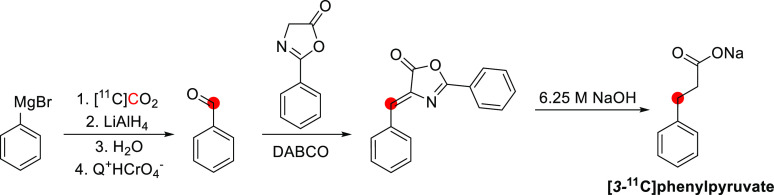
Synthesis of [*3*-^11^C]phenylpyruvate from [^11^C]CO_2_.^428^^11^C radionuclide position is highlighted in red.

The preparation of [*4*-^11^C]phenylpyruvic acid in the 4- position of the side chain was first described in 1986, starting from [^11^C]CO_2_ (Figure 189). In this process, a mixture of 2-phenyl-5-oxazolone in absolute ethanol and *1*,*4*-diazabicyclo[*2*.*2*.*2*]octane (DABCO) in absolute ethanol was added to a solution containing [^11^C]benzaldehyde. NaOH was added to the mixture to convert the condensation product [α-^11^C]*4*-benzylidene-*2*-phenyl-*5*-oxazolone into [*4*-^11^C]phenylpyruvic, in a process lasting 40 min with RCY of 40%.^428^

### 12.16 Pyruvic Acid

#### 12.16.1 Radiosynthesis

**Figure 190:**
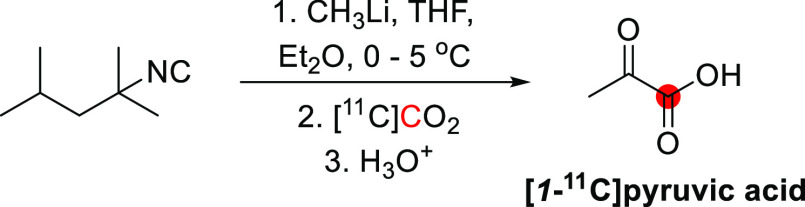
Synthesis of [*1*-^11^C]pyruvic acid using [^11^C]CO_2_. ^11^C radionuclide position is highlighted in red.

The first synthesis of [^11^C]pyruvic acid was done in 1979 from [^11^C]CO_2_, pyruvate-ferredoxin oxidoreductase, and coenzymes.^924^ In 1985, Hara *et al*. synthesized [*1*-^11^C]pyruvate by using a similar method and purified it using sublimation.^891^ Total synthesis was completed in 35 min with a RCY of 80% from [^11^C]CO_2_. [*1*-^11^C]Pyruvic acid was also synthesized by hydrolyzation of carbon-11 labeled *α*-imino acid in 35–40 min (550–9960 MBq)^930^ (Figure 190), and also purification with SEP-PAK C_18_ cartridges, were explicitly studied.^931^ Another enzymatic radiosynthesis was also done by using two different purification methods.^241^ Both methods had the same radiolabeled starter, [^11^C]HCN, and *l*-[*1*-^11^C]alanine was synthesized with [*1*-^11^C]pyruvic acid and purified both *via*d-amino acid oxidase and l-alanine dehydrogenase and *via**d*- and *l*- amino acid oxidases. A similar method from *d*,*l*-[*1*-^11^C]alanine was also investigated, and synthesis was completed in 47 min with 703 MBq activity.^932^

Pyruvic acid was also labeled from position *3*- in different studies. The known first reaction started from [^11^C]CH_3_I to form racemic d,*l*-[*4*-^11^C]alanine and then enzymatically synthesis [4-^11^C]pyruvic acid in 35 min with a RCY of 73% and RCP >99%.^898^ In addition to these methods, [*4*-^11^C]pyruvic acid synthesis was also done with immobilized enzymes in one single column.^933^ For radiolabeling, *d*,l-[*4*-^11^C]alanine was synthesized from [^11^C]CH_3_I, and different enzymes were used for each method. Similar synthesis from *d*,*l*-[*4*-^11^C]alanine, *d*-amino acid oxidase, alanine racemase, and catalase resulted in approximately 52 min with a RCY ∼ 18% and RCP > 99%.^892^ It was also used to synthesize carbon-11 labeled lactic acid as a reactive intermediate.^879,925^

#### 12.16.2 Preclinical Studies

The first body distribution study of [*1*-^11^C]pyruvate was tried on female rabbits with a brain tumor and injected through the ear vein.^891^ According to the results, radioactivity in the tumor had increased in time, and in the liver, it had decreased. Radioactivity accumulation was seen in the urine in the urinary bladder. [*1*-^11^C]pyruvate has been used for the estimation of hemic hypoxia in rats and compared with healthy ones.^889,890^

*In vivo* biodistribution of [*4*-^11^C]pyruvate was studied in two previously healthy mini-pigs that had diabetic hearts in the study.^893^ Another *in vivo* study that used [*4*-^11^C]pyruvate was the characterization of prostate cancer metabolic phenotype in two different prostate cancers.^892^ For this study, male tumor-bearing mice were used, and after 10 min of injection, 30 min PET scans were done.

#### 12.16.3 Clinical Studies

The first clinical study with [*1*-^11^C]pyruvate purified by sublimation was performed on a 51-year-old man with a brain tumor.^891^ [*1*-^11^C]Pyruvate was intravenously injected, and images were collected at 0–5 and 10–15 min p.i. Tumor accumulation was observed in this study. The same research group then tried [*1*-^11^C]pyruvate on 8^894^ and 12 patients.^895^ In the first study, patients were females and males with different brain problems between 38 and 78 years old, and the study aimed to visualize ischemia and infarction in the brain.^894^ [*1*-^11^C]Pyruvate was given intravenously, and a PET scan was started immediately after the injection. In the second study, female and male patients between 40 and 50 years old with different brain tumors.^895^ According to this study, [1-^11^C]pyruvate can be used on local brain tumors using a PET scan. *In vivo* metabolism of [1-^11^C]pyruvate was also investigated for brain tissue distribution in patients with epilepsy and Leigh’s disease and brain and epicranial muscle distribution in mitochondrial encephalomyopathy.^896^ Pyruvate metabolism was also investigated by using [*1*-^11^C]pyruvate.^897^

### 12.17 Salicylic Acid

#### 12.17.1 Radiosynthesis

**Figure 191:**
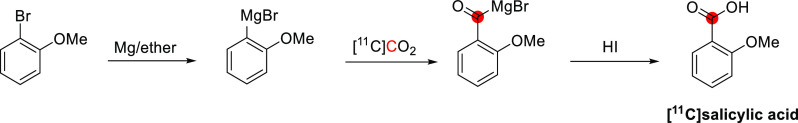
Synthesis of [^11^C]salicylic acid using [^11^C]CO_2_.^899^^11^C radionuclide position is highlighted in red.

[^11^C]Salicylic acid was reported by Sasaki *et al*. through carboxylation of *2*-bromomagnesiumanisole using [^11^C]CO_2_ (Figure 191) and gave [^11^C]salicylic acid with a RCY of 7.3 ± 1.6% and *A*_m_ of 23.5 ± 9.6 GBq/μmol.^899^ An alternative preparation was described by Winstead *et al*., where salicylic acid (cold compound) was produced by carbonating the aryl lithium intermediate obtained from the reaction of *o*-bromophenol with *n*-butyl lithium. Although details were not provided on the radiosynthesis procedure, a RCY of 50% was obtained.

#### 12.17.2 Preclinical Studies

[^11^C]Salicylic acid was studied in mice by Sasaki *et al*. in 1999 to evaluate the production of *2*,*3*-dihydroxybenzoic acid and *2*,*5*-dihydroxybenzoic acid as a result of the reaction of reactive oxygen species with salicylic acid.^899^ Biodistribution studies at 1 min p.i. found the highest uptake in the kidneys and blood, with lower uptake in the liver, lung, and heart.^899^ Studies on dogs showed activity uptake in the heart-blood pool, liver, and upper abdomen at 2–3 min p.i.^531^

### 12.18 Salvinorin A

#### 12.18.1 Radiosynthesis

**Figure 192:**
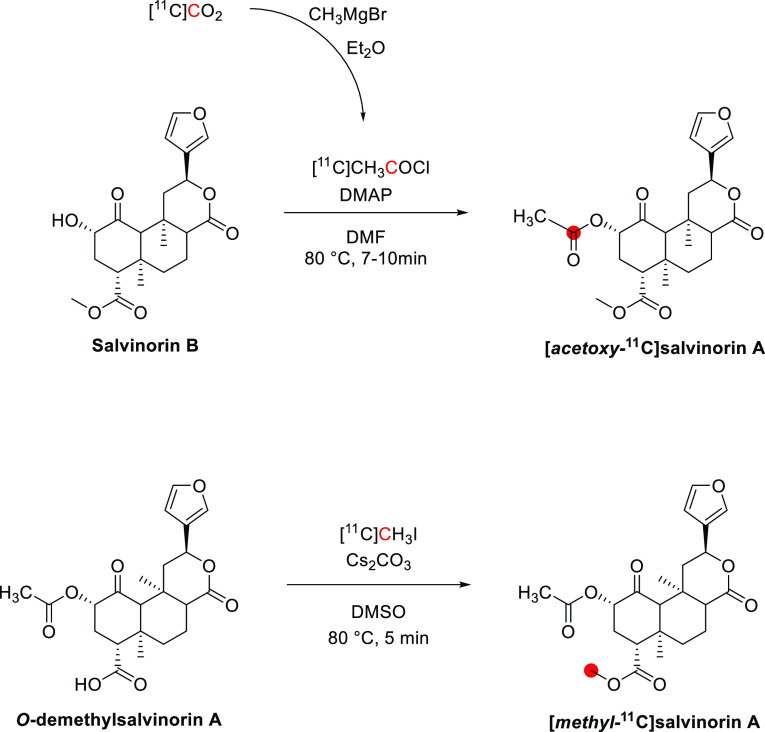
Synthesis of [*acetoxy*-^11^C]salvinorin A using [^11^C]CH_3_COCl and [*methyl*-^11^C]salvinorin A using [^11^C]CH_3_I. ^11^C radionuclide position is highlighted in red.

The production of [^11^C]salvinorin A used [^11^C]CH_3_COCl as a radiolabeling agent, which was prepared by reaction of cyclotron produced [^11^C]CO_2_ with methyl magnesium bromide in diethyl ether. [^11^C]CH_3_COCl was then transferred into a second vial containing a solution of salvinorin B (a deacetylated analogue of salvinorin A, Figure 192) and *4*-dimethylaminopyridine (DMAP) in DMF and reacted for 7–10 min.^813^ [*Acetoxy*-^11^C]salvinorin A was obtained within 40 min with RCY of 3.5–10% (based on the initial activity of [^11^C]CO_2_) and *A*_m_ of 7.4–27.8 GBq/μmol (corrected to EOB).^813^

Alternatively, [^11^C]salvinorin A was radiolabeled using [^11^C]CH_3_I starting from *O*-demethylsalvinorin A (Figure 192).^900^ The reaction was carried out for 5 min at 80 °C in DMSO with the aid of Cs_2_CO_3_. This alternative route yielded [*methyl*-^11^C]salvinorin A with RCY of 72 ± 6% and *A*_m_ of 159.1 ± 44.4 GBq/μmol.

#### 12.18.2 Preclinical Studies

**Figure 193:**
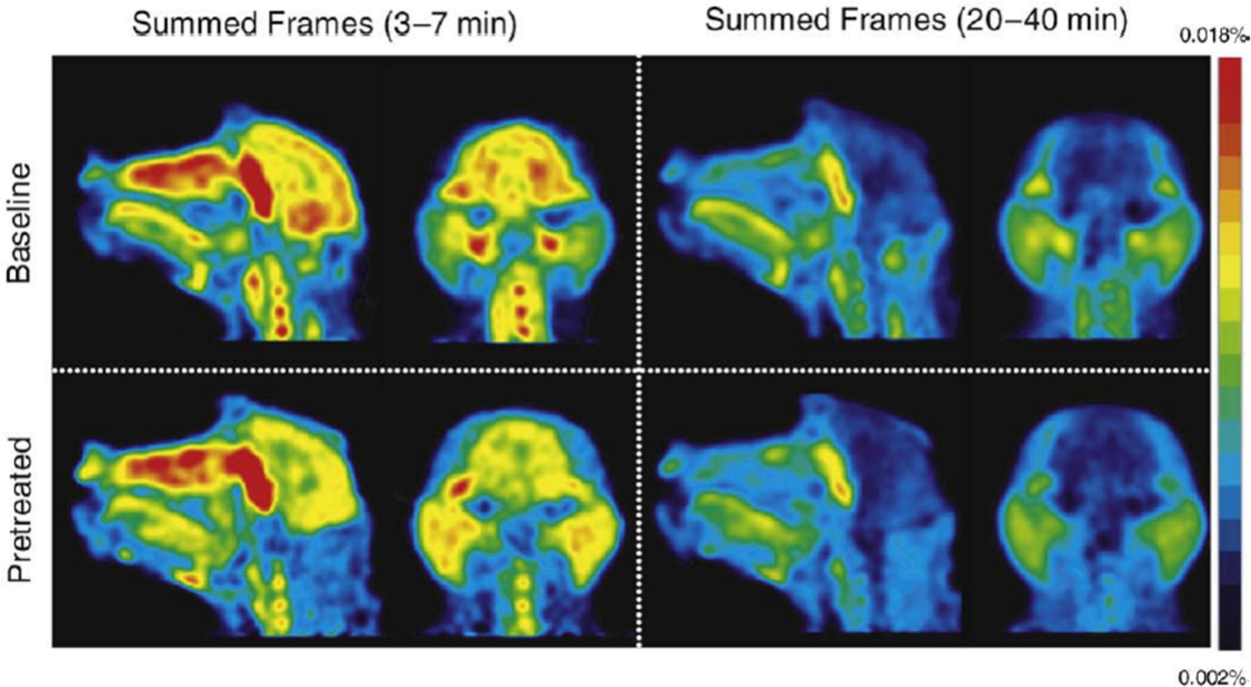
[^11^C]Salvinorin A PET images of*Papio Anubis*baboons with (bottom) and without (top) naloxone pretreatment. Reproduced with permission from ref (813). Copyright 2008 Elsevier.

Pharmacokinetics of [^11^C]salvinorin A was initially assessed on six female*Papio Anubis* baboons on either the brain or the chest. [^11^C]Salvinorin A was injected and scans proceeded for 60 min with and without prior administration of naloxone (15 min before [^11^C]salvinorin A administration, 1.0 mg/kg) for binding specificity assessment.^813^ PET scanning showed that the concentration of [^11^C]salvinorin A rapidly peaks in the brain (3.3% of the administered dose within 40 s) and washes out with a half-life of 8 min, with the most affected areas being in salivary and nasal tracts and the cerebellum, which is in contrast with known κ opioid receptor expression. Moreover, the binding was constant even with naloxone pretreatment (Figure 193), suggesting a high degree of nondisplaceable binding.^813^ Metabolite studies also highlighted the quick metabolism of [^11^C]salvinorin A to [^11^C]salvinorin B.^813,900^

### 12.19 Urea

#### 12.19.1 Radiosynthesis

**Figure 194:**
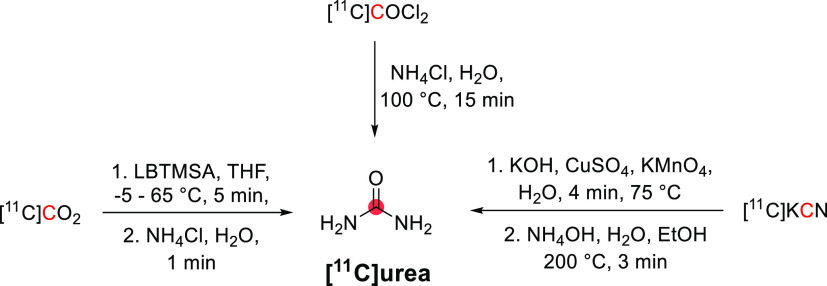
Synthesis of [^11^C]urea from [^11^C]CO_2_, [^11^C]COCl_2_, and [^11^C]KCN. ^11^C radionuclide position is highlighted in red.

In 1980, it was first reported [^11^C]urea’s automated radiosynthesis from [^11^C]COCl_2_ and ammonia aqueous solution reacting at 100 °C for 15 min (Figure 194). The total synthesis takes 30 min, achieving RCY of 15% with respect to [^11^C]CO_2_.^754^ This procedure was further optimized by Bera *et al*. using a continuous conversion of [^11^C]CO_2_ to [^11^C]urea and by maintaining moisture-free conditions with a frequent catalyst replacement.^902^ In this case, 296–370 MBq of [^11^C]urea were produced on-line from 925–1110 MBq of [^11^C]CO_2_, with RCY of 35% and *A*_m_ of 6.475 GBq/μmol.^902^

More recently, [^11^C]urea was prepared as an intermediate in the synthesis of [2-^11^C]thimidine from [^11^C]COCl_2_ by reacting with aqueous ammonia and achieving a RCC of 55% in 20 min.^724^ Furthermore, [^11^C]urea’s radiosynthesis was investigated starting from [^11^C]cyanide to increase RCY and A_m_. Firstly, [^11^C]KCN was converted to [^11^C]KOCN by catalytic oxidation with Cu(OH)_2_ and successively converted to its ammonium salt with ammonium hydroxide to achieve final [^11^C]urea (Figure 194). After HPLC purification, RCY was higher than 85% from no-carrier-added [^11^C]KCN in 20–25 min with *A*_m_ of 129.5 ± 29.6 GBq/μmol at EOB.^903,934^ Further optimizations by increasing the pH and temperature and using an appropriate vessel to reduce urea decomposition provided RCY of 95.0 ± 2.5%, decreasing the total synthesis time to approximately 16 min from EOB.^755^ Lastly, [^11^C]urea was also obtained directly from [^11^C]CO_2_ by reaction with lithium bis(trimethylsilyl)amide (LBTMSA) in THF, followed by a process with aqueous NH_4_Cl solution. In this case, no-carrier-added [^11^C]urea was achieved with a RCY of 55–70% in 16 min from EOB (Figure 12.32).^729^

#### 12.19.2 Clinical Studies

[^11^C]Urea was evaluated as a potential external imaging radiotracer to detect *Helicobacter pylori* colonization in the stomach by locating its specific urease activity. Notably, a 200 mL aqueous solution containing [^11^C]urea with [^99m^Tc]diethylenetriamine pentaacetate ([^99m^Tc]DTPA) (a nonabsorbable marker used as a control for loss of radioactivity from the stomach due to gastric emptying) was orally administered to a patient suffering from esophageal and gastric erosions due to *Helicobacter pylori*’s presence and to healthy negative control. From 5–10 min after administration, radioactivity in the stomach decreases due to gastric emptying and urea metabolism until 20 min, after which it remains constant. Simultaneously, in the breath test in the same time frame, increasing ^11^C activity was registered in the patient due to [^11^C]urea hydrolysis in [^11^C]CO_2_ by *H. pylori*, while ^11^C activity in control subject breath remains at background level.^901^

### 12.20 Uric Acid

#### 12.20.1 Radiosynthesis

**Figure 195:**
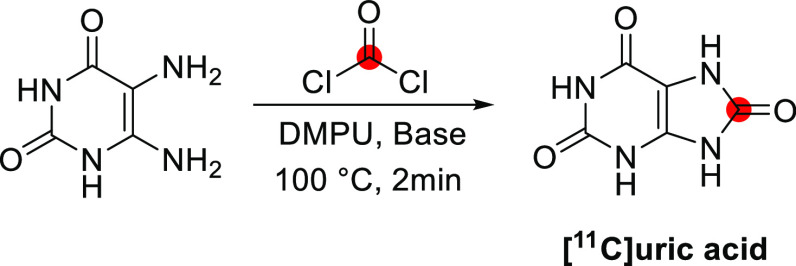
Synthesis of [^11^C]uric acid from [^11^C]COCl_2_ and *5*,*6*-diaminouracil. ^11^C radionuclide position is highlighted in red.

[^11^C]Uric acid was obtained in an efficient and automated synthesis method from the reaction of [^11^C]COCl_2_ and 5,6-diaminouracil in *N*,*N*-dimethylpropyleneurea (DMPU) solution at 100 °C for 2 min (Figure 195). After cooling, the reaction mixture was diluted with phosphate buffer, and the final product was purified using a preparative HPLC system. [^11^C]Uric acid was prepared within 30 min after the EOB with a RCY of 36 ± 6%, *A*_m_ of 89–142 GBq/μmol, and RCP of 98%.^904^

#### 12.20.2 Preclinical Studies

**Figure 196:**
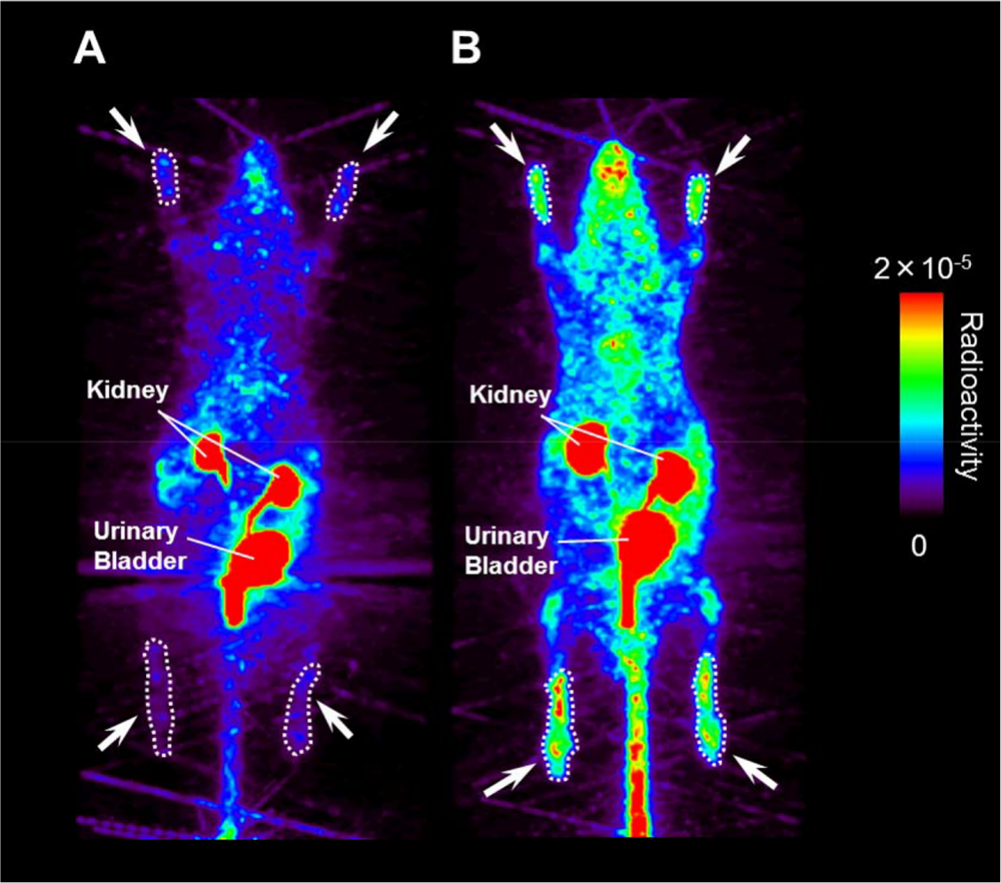
[^11^C]Uric acid whole-body MIP PET image in normal (A) and hyperuremic (B) rats (65–70 min p.i.). Arrows indicate the region of limbs in which a higher accumulation of radioactivity was observed. Reproduced with permission from ref (904). Copyright 2012 Elsevier.

[^11^C]Uric acid has been evaluated in two rats after intravenous bolus injection *via* the tail vein under normal or hyperuricemic conditions (Figure 196). The scans were performed at 65–70 min p.i., in addition to blood sampling *via* the femoral vein. The radiometabolite analysis of blood specimens under normal conditions showed that most of the radioactivity was derived from the metabolite [^11^C]allantoin, whereas, under hyperuricemic conditions, almost all radioactivity was present as [^11^C]uric acid. In addition, the limbs showed a high accumulation of radioactivity under hyperuricemic conditions. Thus, according to Yashio *et al*. [^11^C]uric acid could be a potential tool for diagnosing hyperuricemia, gout, and other urate-related lifestyle diseases.^904^

## 13 Future Perspectives and Conclusions

Since the discovery of carbon-11, many synthetic methods have been developed and applied to incorporate carbon-11 into organic molecules. This manuscript reviews these methods and their subsequent application to the synthesis of carbon-11 labeled endogenous compounds: alcohols, alkaloids, amino acids, enzyme cofactors, and vitamins, endogenous gases, fatty acids, hormones and neurotransmitters, nucleotides, peptides, sugars, and miscellaneous compounds (up until May 2022). A brief description of clinical and preclinical *in vivo* studies is provided, along with a selection of synthetic schemes and images/data from *in vivo* PET imaging studies.

In the review, we show PET imaging can provide pivotal information on how endogenous compounds are trafficked, interact with molecular recognition sites, accumulate in organs, and metabolize in living animals/humans. As discussed herein, the choice of the route of radiotracer administration affects the tracer’s pharmacokinetics and temporal/regional biodistribution (see oral *versus* iv, for [^11^C]biotin and [^11^C]niacin; inhalation *versus* iv, [^11^C]nicotine). In particular, the choice of the iv route, the most common route of PET radiotracer administration, allows the elucidation of the trafficking of endogenous compounds *via* the bloodstream. This does not, however, account for the transport from their site of synthesis (*e.g*., in the brain or gut) into the bloodstream.

Based on the aim to radiolabel autologously endogenous compounds with carbon-11, PET radionuclides such as ^13^N and ^15^O can be employed. However, ^15^O and ^13^N have very short half-lives (2.1 and 10 min, respectively), requiring an unwieldy amount of starting radioactivity for performing the steps from the start of the radiolabelling process to the end of PET imaging. Moreover, their use is also hampered by the limited rapid synthetic approaches available to incorporate ^15^O and ^13^N into endogenous compounds despite some valuable progress being made.^935,936^ Therefore, the most convenient choice for the autologous radiolabelling of endogenous compounds often leads to the use of carbon-11. This also allows the incorporation of the radionuclide in different positions of the organic scaffold, enabling the study of diverging metabolic pathways.

The favorable half-life of carbon-11 also allows the administration of multiple radiotracers to the same subject on the same day, enabling a systems biology approach to multiplex PET imaging. This will become increasingly important with the recent implementation of extremely sensitive “total body PET” scanners, enhancing the feasibility of multitracer studies in individuals at significantly lower radiation doses (also making possible studies in pregnant women and pediatrics).

Over the years, we have seen the evolution of novel, simplified radiosynthetic approaches employing rapid and fully automated methods to increase synthetic robustness and reliability of processing, improve radiochemical yields, minimize synthesis times and losses of radioactivity due to technical handling.

Nowadays, comprehensive regulatory practices (initially intended for regulating commercial drug development at scale) have been applied to the bespoke synthesis of short-lived radiotracers, including stricter regulations for evaluating the toxicology of subpharmacological doses (usually subnanomolar doses) of novel radiotracers applied in PET studies. However, the toxicological profile of endogenous compounds is almost exclusively well documented, removing this costly and time-consuming step in the translational process.

Finally, the variety and flexibility of synthetic approaches using carbon-11 discussed here are matched by the biological complexity of carbon-based life forms. As such, the future of carbon-11 labeled compounds for *in vivo* imaging of biological systems will expand in step with our burgeoning knowledge of in vivo physiology, biochemistry, and pharmacology in health and disease.

## Abbreviations

*2*,*5*-DHBA: *2*,*5*-dihydroxybenzoic
*5*-HT: *5*-hydroxytryptamine
*5*-HTP: *5*-hydroxytryptophan
AA: amino acid
AADC: aromatic l-amino acid decarboxylase
ACC: acetyl-*l*-[*methyl*-^11^C]carnitine
AChE: acetylcholinesterase
AChR: acetylcholine receptor
ACM: [*2*-^11^C]acetyl-*l*-carnitine
ACPC: aminocyclopentanecarboxylic acid
AD: Alzheimer’s disease
ADP: adenosine diphosphate
*A*_m_: molar activity
AMP: Adenosine monophosphate
AMPA: a-amino-*3*-hydroxy-*5*-methyl-*4*-isoxazolepropionic acid
ASD: autism spectrum disorder
ATP: adenosine triphosphate
ATRA: [^11^C]all-*trans*-retinoic acid
BAT: brown adipose tissue
BBB: blood–brain barrier
CAI: carbonic anhydrase
cAMP: cyclic adenosine monophosphate
CAN: [*1*-^11^C]acetyl-*l*-carnitine
CBF: cerebral blood flow
CFS: chronic fatigue syndrome
cGMP: cyclic guanosine monophosphate
COMPDS: compounds
CMR: cerebral metabolic rate
CMT: carminomycin-*4*-*O*-methyltransferase
CNS: central nervous system
CoA: coenzyme A
CPSR: cerebral protein synthesis rate
CRN: *l*-[*methyl*-^11^C]carnitine
CT: computerized tomography
D-*3*-HBD: (*d*)-*β*-hydroxybutyrate dehydrogenase
DABCO: *1*,*4*-diazabicyclo[*2*.*2*.*2*]octane
DBAD: di-*tert*-butyl azodicarboxylate
DBU: *1*,*8*-diazabicyclo[*5*.*4*.*0*]undec-*7*-ene
DCC: *N*,*N*′-dicyclohexylcarbodiimide
DEM: diethyl maleate
DHA: dehydroascorbic acid
DMAP: *4*-dimethylaminopyridine
DMF: dimethylformamide
DMPU: *N*,*N*-dimethylpropyleneurea
DMSO: dimethyl sulfoxide
DNA: deoxyribonucleic acid
DOPA: dihydroxyphenylalanine
DTPA: diethylenetriamine pentaacetate
ee: enantiomeric excess
EOB: end of bombardment
EOD: end of delivery
EOS: end of synthesis
EPI: [^11^C]epinephrine
FA: fatty acid
FDG: fluorodeoxyglucose
GABA: *γ*-aminobutyric acid
Gln: glutamine
GLUT: glucose transport
GMP: Good Manufacturing Practice
Hb: hemoglobin
HDAC: histone deacetylase
HED: [^11^C]hydroxyephedrine
HIAA: hydroxyindoleacetic acid
HPLC: high-pressure liquid chromatography
HTP: hydroxytryptophan
IPr: *1*,*3*-bis(*2*,*6*-diisopropylphenyl)imidazol-*2*-ylidene
iv: intravenously
LAT-1: *L*-type amino acid transporter 1
LBTMSA: lithium bis(trimethylsilyl)amide
LC: liquid chromatography
LTE: leukotriene
MAO: monoamine oxidases
MAT: *l*-methionine-*S*-adenosine transferase
MCT1: monocarboxylate transporter 1
MeCN: acetonitrile
MIP: maximum intensity projection
MOR: μ-opioid receptor
MRI: magnetic resonance imaging
MT: methoxytyramine
*N*,*N*-DMPEA: *N*,*N*-dimethylphenethylamine
ndc: not decay corrected
nr: not reported
nAChRs: nicotinic acetylcholine receptors
NAD: nicotinamide adenine dinucleotide
NADH: nicotinamide adenine dinucleotide
NADP: nicotinamide adenine dinucleotide phosphate
NMDA: *N*-methyl-*d*-aspartate
ODC: ornithine decarboxylase
p.i.: post-injection
P.N.M.T.: phenylethanolamine *N*-methyltransferase
PABA: *4*-aminobenzoic acid
PAT: palmitoyl acyltransferase
pB: binding potential
PBS: phosphate-buffered saline
PBu_3_: tributylphosphine
PDE10A: phosphodiesterase 10A
PET: positron emission tomography
Pgl: protein glycosyltransferase
P-gp: P-glycoprotein
RCC: radiochemical conversion
RCP: radiochemical purity
RCY: radiochemical yield
RNA: ribonucleic acid
ROS: reactive oxygen species
SAM: *S*-adenosylmethionine
SMCT: sodium-coupled monocarboxylate transporters
SMVT: sodium-dependent vitamin transporter
SPE: solid-phase extraction
SVCT1-2: sodium-dependent active transporters
TBAH: tetrabutylammonium hydroxide
TBDMS: *tert*-butyldimethylsilyl
TCA: tricarboxylic acid
THF: tetrahydrofuran
TMSCl: trimethylsilyl chloride
TS: thymidylate synthase
*V*_d_: distribution volume
WAT: white adipose tissue
