# Correction to “Radiosynthesis, Preclinical and Clinical Positron Emission Tomography Studies of Carbon-11 Labeled Endogenous and Natural Exogenous Compounds”

**DOI:** 10.1021/acs.chemrev.3c00470

**Published:** 2023-07-27

**Authors:** Antonio Shegani, Steven Kealey, Federico Luzi, Filippo Basagni, Joana do Mar Machado, Sevban Doğan Ekici, Alessandra Ferocino, Antony D. Gee, Salvatore Bongarzone

The Acknowledgment should be corrected to include the following: Sevban Doğan Ekici sincerely acknowledges the Turkish Ministry of National Education for their sponsorship of her PhD studies. Joana do Mar Machado would like to acknowledge funding from the EPSRC Centre for Doctoral Training in Smart Medical Imaging (EP/S022104/1).

